# Supplementary material for: Stereoselective gridization and polygridization with centrosymmetric molecular packing
Source: Nat Commun. 2020 Apr 9;11:1756. doi: 10.1038/s41467-020-15401-x (PMC7145858; doi:10.1038/s41467-020-15401-x)
Supplement: Supplementary file 1 — Supplementary Information [file 41467_2020_15401_MOESM1_ESM.pdf]

Supplementary Information

Stereoselective Gridization and Polygridization with Centrosymmetric Molecular Packing

Lin et al.

Supplementary Methods

Supplementary Note 1. Characteristics of Friedel-Crafts reactions and Gridization

Supplementary Note 2. The characteristics of the polygridization from DC-F monomers

Supplementary Note 3. Characteristics on the stereoselectivity of DHGs and PDHG-F structures

Supplementary Note 4. The calculation of expansion factor *β* of PDHG-F in solution

Supplementary Note 5. Molecular weight calibration and calculation

Supplementary Note 6. The hydrodynamic radius distribution of PDHG-F chains

Supplementary Note 7. Mechanistic analysis of the gridization process

Supplementary Note 8. The preliminary polygridization kinetics of polygridization

Supplementary Note 9. Molecular dynamic simulation of PDHG-F chain collapse under other DPs.

Supplementary Note 10. NMR spectra for all substrates and products

Supplementary Note 11. Single crystal crystallography

Supplementary References

Supplementary Methods

Materials

Unless otherwise information noted, all reagents were obtained from commercial source and previous reports without purification further. Most of products were purified via column chromatography over silica gel (200-300 mesh) (from Nanjing Wanqing Chemical Instruments Company) and some of them were purified by recrystallization. The synthetic methods of diazafluorenones and diazafluorenol **1a** are referred to the literatures[1,2]. Synthesis of fluorenol **F-1b** is referred to the literature[3].

5-(4-Methoxyphenyl)-5*H*-cyclopenta[2,1-b:3,4-b']dipyridin-5-ol (1b)

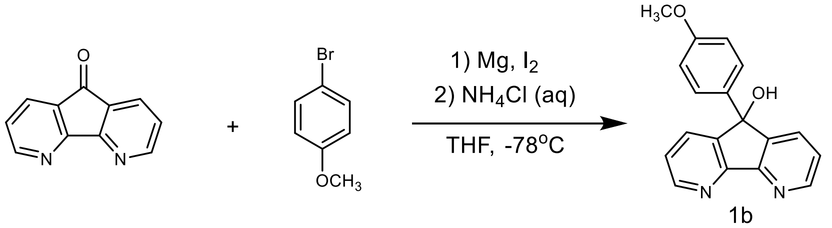

This compound **1b** was synthesized according to the preparation of **1a**[2]. Substrates: Magnesium (480 mg, 19.8 mmol, 3.3 equiv), 1-bromo-4-methoxybenzene (2826 mg, 18 mmol, 3 equiv) and 4,5-diazafluorenone (1.08 mg, 6 mmol, 1 equiv)] and purified by and column chromatography [silica gel, eluent solvents with petroleum: DCM: ethyl acetate = 1:1:1, trimethylamine (2 drops per 100 ml eluent) should be added in whole isolation] followed by washing with DCM and petroleum ether to give **1b** (White solid, 1350 mg, 4.65 mmol. Yield: 78%). ^{1}H NMR (400 MHz, $CDCl_3$): δ (ppm) 8.26 – 8.25 (dd, *J* = 4.9, 1.2 Hz, 2H), 7.61 – 7.59 (dd, *J* = 7.6, 1.2 Hz, 2H), 7.26 – 7.23 (d, *J* = 8.8 Hz, 2H), 7.01 – 6.98 (dd, *J* = 7.6, 4.9 Hz, 2H), 6.77 – 6.75 (d, *J* = 8.8 Hz, 2H), 4.79 (s, 1H), 3.73 (s, 3H). ^{13}C NMR (100 MHz, $CDCl_3$): δ (ppm) 159.1, 156.7, 150.5, 145.8, 133.3, 132.8, 126.6, 123.9, 113.8, 79.2, 55.2. FT-IR (cm^{-1}): 3123, 2995， 2836, 1606, 1593, 1567, 1510, 1464, 1443, 1403, 1303, 1244, 1205, 1167, 1106, 1052, 1035, 984, 948, 923, 839, 806, 773, 747, 692, 670, 629. HRMS (ion: MALDI, FT-ICR-MS): m/z calcd for [$M+H^+$] $C_{18}H_{15}N_2O_2$: 291.1128; found: 291.1126.

3-Bromo-5-(4-methoxyphenyl)-5*H*-cyclopenta[2,1-b:3,4-b']dipyridin-5-ol (1c)

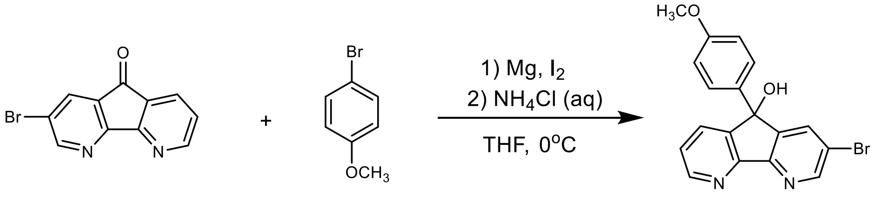

Magnesium (157.5 mg, 6.6 mmol, 3.3 equiv) and a drop of iodine were added to the three-neck flask in the nitrogen atmosphere. Then 1-bromo-4-methoxybenzene (1122 mg, 6 mmol, 3 equiv) and THF (5 mL) were added under nitrogen protection to give a solution containing the Grignard reagent. Then the solution was added into the solution of 2-bromo-4,5-diazafluorenone [520 mg, 2 mmol, 1 equiv, its synthesis is referred to the literature[1]] in tetrahydrofuran (35 ml) under nitrogen protection at 0 °C

to reflux for 20 min. It is noted that the Grignard reagent should be dropped very slowly. Then NH_4Cl solution was added to quench and neutralize the reaction. Then DCM was added for extraction, and the aqueous layer was washed by DCM for three times. The combined organic solution was dried with anhydrous Na_2SO_4. After the filtration, the crude product was collected by evaporating the solvent. The crude product was purified by column chromatography [silica gel, petroleum ether: DCM: ethyl acetate = 2:2:1, trimethylamine (2 drops per 100 ml eluent) should be added in whole isolation] and washing with petroleum ether and DCM to give **1c** (White solid, 494 mg, 1.34 mmol. Yield: 67%). It is noted that higher temperature (30~40 ^{o}C) can lead to byproducts, which reduces the yield of **1c**. ^{1}H NMR (400 MHz, $CDCl_3$): δ (ppm) 8.26 – 8.25 (m, 2H), 7.76 – 7.75 (d, *J* = 2.0 Hz, 1H), 7.64 – 7.62 (dd, *J* = 7.7 Hz, 1.3 Hz, 1H), 7.24 – 7.22 (dd, *J* = 8.8 Hz, 2H), 7.13 – 7.10 (dd, *J* = 7.6 Hz, 4.9 Hz, 1H), 6.81 – 6.79 (d, *J* = 8.8 Hz, 2H), 5.59 (s, 1H). 3.76 (s, 3H). ^{13}C NMR (100 MHz, $CDCl_3$): δ (ppm) 159.3, 155.6, 154.9, 151.5, 150.7, 147.6, 145.9, 135.7, 133.0, 132.5, 126.6, 124.2, 121.4, 114.0, 78.9, 55.3. HRMS (ion: MALDI, FT-ICR-MS): m/z calcd for [M+H^{+}] $C_{18}H_{14}BrN_2O_2$: 369.0233; found: 369.0231.

3,7-Dibromo-5-(4-methoxyphenyl)-5*H*-cyclopenta[2,1-b:3,4-b']dipyridin-5-ol (1d)

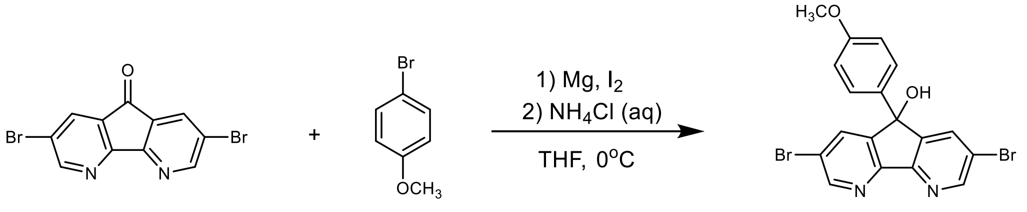

1d

This compound **1d** was synthesized according to the preparation of **1c** [substrates: Magnesium (554.4 mg, 23.1 mmol, 3.3 equiv), 1-bromo-4-methoxybenzene (3927 mg, 21 mmol, 3 equiv) and 2,7-dibromo-4,5-diazafluorenone (2380 mg, 7 mmol, 1 equiv), its synthesis is referred to the literature[1]] and purified by and column chromatography [silica gel, petroleum ether: DCM: ethyl acetate = 5:5:1, trimethylamine (2 drops per 100 ml eluent) should be added in whole isolation] followed by washing with DCM and petroleum ether to give **1d** (White solid, 2252 mg, 5.04 mmol. Yield: 72%). ^{1}H NMR (400 MHz, $CDCl_3$): δ (ppm) 8.48 – 8.47 (d, *J* = 2.0 Hz, 2H), 7.78 – 7.77 (d, *J* = 2.0 Hz, 2H), 7.25 – 7.23 (d, *J* = 8.8 Hz, 2H), 6.85 – 6.83 (d, *J* = 8.8 Hz, 2H), 4.42 (s, 1H), 3.79 (s, 3H). ^{13}C NMR (100 MHz, $CDCl_3$): δ (ppm) 159.6, 154.3, 152.2, 147.1, 135.8, 131.4, 126.5, 121.9, 114.2, 78.9, 55.3. HRMS (ion: MALDI, FT-ICR-MS): m/z calcd for [M+H^{+}] $C_{18}H_{13}Br_2N_2O_2$: 446.9338; found: 446.9336.

2-Bromo-5-(*p*-tolyl)-5*H*-cyclopenta[2,1-b:3,4-b']dipyridin-5-ol (1e)

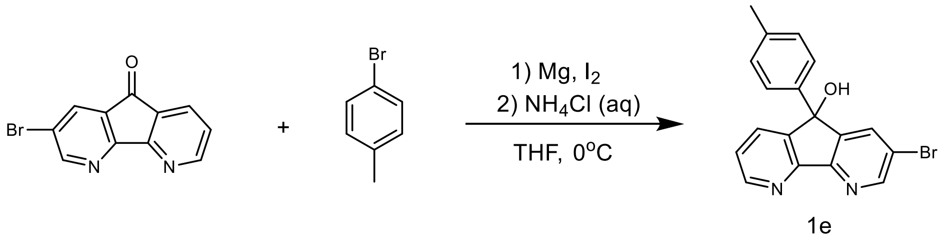

This compound **1e** was synthesized according to the preparation of **1c** [substrates: Magnesium (157.5 mg, 6.6 mmol, 3.3 equiv), 1-bromo-4-methylbenzene (1026 mg, 6 mmol, 3 equiv) and 2-bromo-4,5-diazafluorenone (520 mg, 2 mmol, 1 equiv)] and purified by column chromatography [silica gel, petroleum ether: DCM: ethyl acetate = 2:2:1, trimethylamine (2 drops per 100 ml eluent) should be added in whole isolation] followed by washing with petroleum ether and DCM to give **1e** (White solid, 367 mg, 1.04 mmol. Yield: 52%) ^{1}H NMR (400 MHz, $CDCl_3$): δ (ppm) 8.44 (m, 2H), 7.78 (s, 1H), 7.67 – 7.65 (d, J = 7.7 Hz, 1H), 7.27 (s, 1H), 7.22 – 7.17 (m, 3H), 7.12 – 7.10 (d, J = 7.1 Hz, 2H), 2.32 (s, 3H). ^{13}C NMR (100 MHz, $CDCl_3$): δ (ppm) 155.9, 155.1, 151.8, 151.0, 147.3, 145.6, 137.9, 137.3, 135.8, 133.0, 129.4, 125.2, 124.3, 121.5, 79.3, 21.1. HRMS (ion: MALDI, FT-ICR-MS): m/z calcd for $[M+H^+]$ $C_{18}H_{14}BrN_2O$: 353.0284; found: 353.0281.

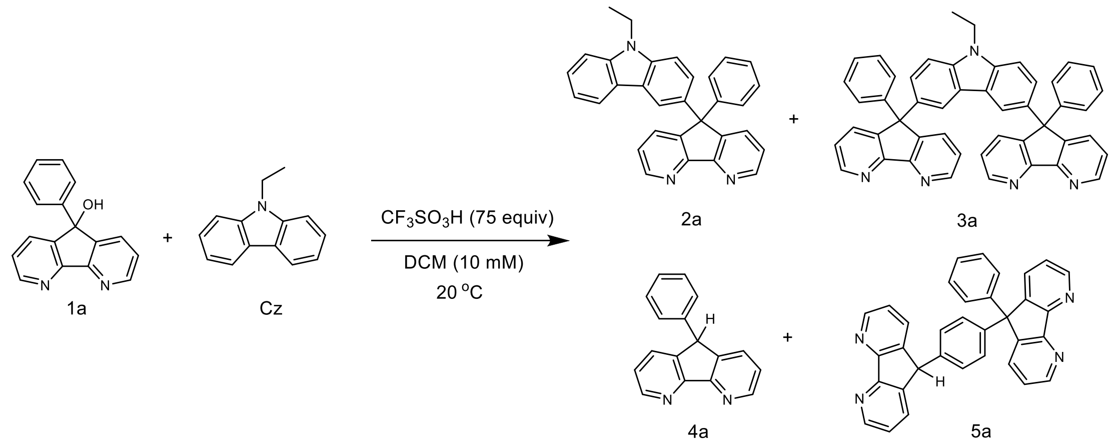

Diazafluorene-based tertiary alcohol **1a** (130 mg, 0.5 mmol, 1 equiv) and 9-ethyl-9*H*-carbazole **Cz** (100 mg, 0.5 mmol, 1 equiv) were added in 50 ml DCM solvent and then the solution was stirred (under 20 °C). Then CF_3SO_3H (3.25 ml, 37.5 mmol, 75 equiv) were added quickly. After the reagent **1a** was completely consumed as monitored by TLC, the excessive solution of potassium hydroxide was added very slowly at the temperature of 0 °C and then extracted with DCM. The mixed organic phase was dried over anhydrous Na_2SO_4 before the organic phase was filtered and concentrated under low pressure. The crude product was purified by column chromatography [silica gel, DCM: ethyl acetate = 2:1, trimethylamine (2 drops per 100 ml eluent) should be added in whole isolation] to give **2a**. The solubility of **3a** is very poor and thus they can be obtained by washing with DCM solvent rather than column chromatography separation. Furthermore, byproduct dehydroxylation **4a** were isolated via column chromatography [silica gel, DCM: ethyl acetate = 1:1, trimethylamine (2 drops per 100 ml eluent) should be added in whole isolation]. It is noted that the polarity

of **4a** is larger than **2a** in thin-layer chromatography. The other byproduct **5a** were isolated through column chromatography [silica gel, DCM: ethyl acetate: methyl alcohol = 30:30:1, trimethylamine (2 drops per 100 ml eluent) should be added in whole isolation] as well. The polarity of **5a** is slightly larger than **3a** in thin-layer chromatography.

5-(9-Ethyl-9*H*-carbazol-3-yl)-5-phenyl-5*H*-cyclopenta[2,1-b:3,4-b']dipyridine (2a)

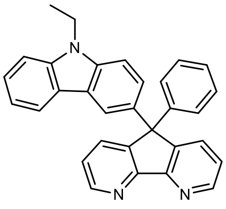

2a

Light yellow solid, 31 mg, 0.07 mmol. Yield: 14%. ^{1}H NMR (400 MHz, $CDCl_3$): δ (ppm) 8.75 – 8.74 (dd, J = 4.8 Hz, 1.5 Hz, 2H), 7.92 – 7.90 (d, J = 7.8 Hz, 1H), 7.86 – 7.83 (m, 3H), 7.46 – 7.42 (t, J = 7.2 Hz, 1H), 7.38 – 7.36 (d, J = 7.5 Hz, 1H), 7.30 – 7.27 (m, 9H), 7.18 – 7.14 (t, J = 7.6 Hz, 1H), 4.35 – 4.30 (q, J = 7.2 Hz, 2H), 1.42 – 1.39 (t, J = 7.2 Hz, 3H). ^{13}C NMR (100 MHz, $CDCl_3$): δ (ppm) 157.6, 150.1, 146.6, 144.5, 140.3, 139.1, 133.9, 128.6, 128.0, 127.4, 126.0, 126.0, 125.8, 123.6, 122.8, 122.5, 120.5, 119.4, 119.0, 108.6, 61.7, 37.6, 13.9. HRMS (DART Positive Ion): m/z calcd for [M+H^{+}] $C_{31}H_{24}N_3$: 438.1965; found: 438.1964.

5,5'-(9-Ethyl-9*H*-carbazole-3,6-diyl)bis(5-phenyl-5*H*-cyclopenta[2,1-b:3,4-b']dipyridine) (3a)

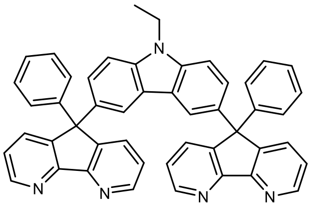

3a

Yellow solid, 99 mg, 0.15 mmol. Yield: 58%. ^{1}H NMR (400 MHz, d_6-DMSO): δ (ppm) 8.85 – 8.84 (d, J = 5.2 Hz, 4H), 8.46 – 8.44 (d, J = 8.0 Hz, 4H), 7.88 (s, 2H), 7.78 – 7.75 (dd, J = 8.0 Hz, 5.2 Hz, 4H), 7.52 – 7.49 (d, J = 8.8 Hz, 2H), 7.30 – 7.27 (m, 6H), 7.21 – 7.17 (m, 6H), 4.44 – 4.43 (m, 123 H, with abnormal integration whose explanation is provided in the spectra section), 1.23 – 1.20 (t, J = 7.1 Hz, 3H). ^{13}C NMR (100 MHz, d_6-DMSO): δ (ppm) 151.1, 148.8, 147.0, 142.8, 140.0, 139.3, 129.4, 128.1, 127.0, 125.9, 122.7, 119.5, 116.3, 110.0, 62.8, 40.5~39.2 (overlapped with the solvent signals), 14.1. HRMS (MALDI, FT-ICR-MS): m/z calcd for [M+H^{+}] $C_{48}H_{34}N_5$: 680.2809; found: 680.2809.

5-Phenyl-5*H*-cyclopenta[2,1-b:3,4-b']dipyridine (4a)

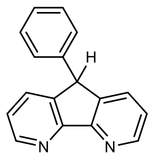

4a

Brown solid. 12 mg, 0.05 mmol. Yield: 10%. ^{1}H NMR (400 MHz, $CDCl_3$): δ (ppm) 8.75 – 8.73 (d, *J* = 4.8 Hz, 2H), 7.67 – 7.65 (d, *J* = 7.7 Hz, 2H), 7.29 – 7.26 (m, 3H), 7.26 – 7.23 (dd, *J* = 7.2 Hz, 4.8 Hz, 2H), 7.08 – 7.05 (dd, *J* = 7.4 Hz, 4.0 Hz, 2H), 5.05 (s, 1H). ^{13}C NMR (100 MHz, $CDCl_3$): δ (ppm) 158.3, 150.0, 142.2, 138.8, 133.1, 129.1, 128.1, 127.6, 123.2, 49.9. HRMS (DART Positive Ion): m/z calcd for [$M+H^+$] $C_{32}H_{25}N_3$: 245.1073; found: 245.1071.

5-(4-(5*H*-cyclopenta[2,1-b:3,4-b']dipyridin-5-yl)phenyl)-5-phenyl-5*H*-cyclopenta[2,1-b:3,4-b']dipyridine (5a)

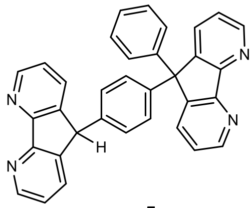

5a

Yellow solid, 7 mg, 0.02 mmol. Yield: 6%. ^{1}H NMR (600 MHz, $CDCl_3$): δ (ppm) 8.74 – 8.72 (m, 4H), 7.74 – 7.73 (d, *J* = 7.1 Hz, 2H), 7.66 – 7.65 (d, *J* = 7.4 Hz, 2H), 7.25 – 7.24 (m, 7H), 7.12 – 7.09 (m, 4H), 6.97 – 6.95 (d, *J* = 7.5 Hz, 2H), 5.02 (s, 1H). ^{13}C NMR (100 MHz, $CDCl_3$): δ (ppm) 158.0, 157.3, 150.2, 149.9, 145.6, 143.2, 143.0, 141.9, 137.9, 133.9, 133.3, 128.7, 128.5, 128.3, 127.7, 127.6, 123.6, 123.4, 61.4, 49.4. HRMS (DART Positive Ion): m/z calcd for [$M+H^+$] $C_{34}H_{23}N_4$: 487.1917; found: 487.1915.

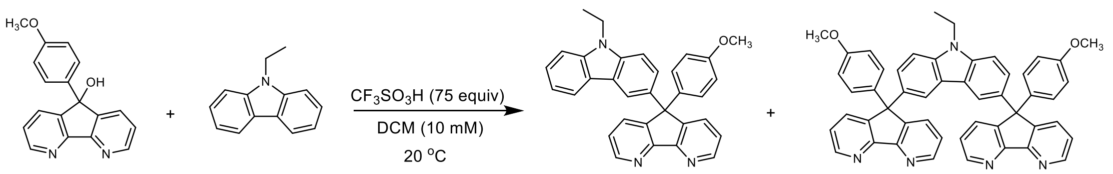

1b Cz 2b 3b

The compounds **2b** and **3b** was synthesized according to the preparation of **2a** and **3a** [substrates: **1b** (200 mg, 0.7 mmol, 1 equiv), **Cz** (195 mg, 0.7 mmol, 1 equiv), CF_3SO_3H (4.75 ml, 52.5 mmol, 75 equiv) and DCM (70 ml)]. It is noted that the solubility of **3b** (White solid) is so poor that the ^{1}H and ^{13}C NMR were not obtained. However, only HRMS (MALDI, FT-ICR-MS, m/z calcd for [$M+H^+$] $C_{50}H_{38}N_5O_2$: 740.3010; found: 740.3010) implied **3b** is likely the target di-DAF-substituted product. It is noted that, when we added CF_3COOH (24 ml) and then the reaction system was stirred in 5 min (before adding CF_3SO_3H), the yield of **2b** is enhanced to 80% but the yield of **3b** is

reduced to 16%. Adding CH_3SO_3H before the addition of CF_3SO_3H, the substrate **1b** transform into **2b** in 62% yield (203 mg, 0.43 mmol) and **3b** in 35% yield (91 mg, 0.12mmol). However, if adding CH_3COOH before the addition of CF_3SO_3H, the reaction did not occur during 30 s (when both yields of **2b** and **3b** are 0%).

5-(9-Ethyl-9*H*-carbazol-3-yl)-5-(4-methoxyphenyl)-5*H*-cyclopenta[2,1-b:3,4-b']dipyridine (2b)

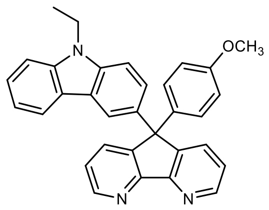

2b

Light yellow solid. ^{1}H NMR (400 MHz, $CDCl_3$): δ (ppm) 8.74 – 8.73 (d, *J* = 5.1 Hz, 2H), 7.91 – 7.89 (d, *J* = 7.8 Hz, 1H), 7.83 – 7.81 (m, 3H), 7.44 – 7.41 (t, *J* = 7.4 Hz, 1H), 7.37 – 7.35 (d, *J* = 8.0 Hz, 1H), 7.29 – 7.26 (m, 2H), 7.19 – 7.13 (m, 3H), 6.80 – 6.78 (d, *J* = 8.7 Hz, 2H), 4.33 – 4.28 (q, *J* = 7.3 Hz, 2H), 3.77 (s, 3H), 1.41 – 1.38 (t, *J* = 7.2 Hz, 3H). ^{13}C NMR (100 MHz, $CDCl_3$): δ (ppm) 158.8, 157.5, 150.0, 146.8, 140.3, 139.1, 136.3, 134.1, 133.8, 129.1, 125.9, 125.7, 123.5, 122.8, 122.5, 120.5, 119.4, 118.9, 113.9, 108.5, 61.1, 55.3, 37.6. FT-IR (cm^{-1}): 3055, 2975, 2930, 2833, 2230, 1602, 1581, 1564, 1508, 1490, 1477, 1470, 1462, 1399, 1345, 1331, 1320, 1287, 1249, 1233, 1179, 1161, 1124, 1113, 1096, 1031, 928, 907, 832, 803, 768, 742, 730, 673, 628. HRMS (DART Positive Ion): m/z calcd for $[M+H^+]$ $C_{32}H_{26}N_3O$: 468.2070; found: 468.2065.

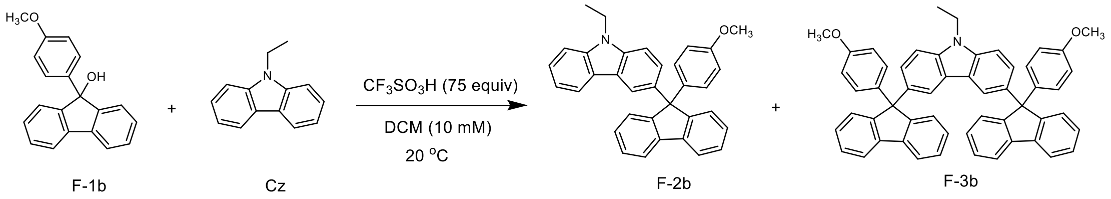

The compounds **F-2b** and **F-3b** was synthesized according to the according to the preparation of **2a** and **3a** [substrates: **F-1b** (200 mg, 0.7 mmol, 1 equiv), **Cz** (195 mg, 0.7 mmol, 1 equiv), CF_3SO_3H (4.75 ml, 52.5 mmol, 75 equiv) and DCM (70 ml)]. They were isolated by column chromatography (silica gel, petroleum ether: DCM = 5:1).

9-Ethyl-3-(9-(4-methoxyphenyl)-9*H*-fluoren-9-yl)-9*H*-carbazole (F-2b)

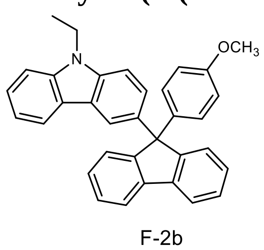

F-2b

White powder, 228 mg, 0.53 mmol. Yield: 75%. ^{1}H NMR (400 MHz, $CDCl_3$): δ (ppm) 7.98 – 7.96 (m, 2H), 7.85 – 7.83 (d, *J* = 7.5 Hz, 2H), 7.55 – 7.53 (d, *J* = 7.5 Hz, 2H), 7.47 – 7.26 (m, 10H), 7.20 – 7.16 (t, *J* = 7.6 Hz, 1H), 6.84 – 6.82 (d, *J* = 8.9 Hz, 2H), 4.33 – 4.28 (q, *J* = 7.2 Hz, 2H), 3.79 (s, 3H), 1.43 – 1.39 (t, *J* = 7.2 Hz, 3H). ^{13}C NMR (100 MHz, $CDCl_3$): δ (ppm) 158.3, 152.3, 140.2, 140.0, 138.8, 138.8, 136.5, 129.3, 127.7, 127.3, 126.3, 125.5, 122.9, 122.6, 120.5, 120.1, 119.7, 118.6, 113.5, 108.4, 108.2, 64.9, 55.2, 37.5, 13.9. HRMS (ion: MALDI, FT-ICR-MS): m/z calcd for [M] $C_{34}H_{27}NO$: 465.2093; found: 465.2087

9-Ethyl-3,6-bis(9-(4-methoxyphenyl)-9*H*-fluoren-9-yl)-9*H*-carbazole (F-3b)

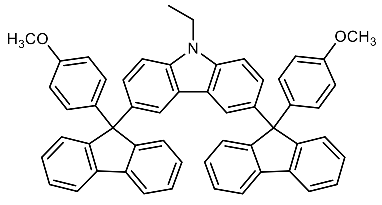

F-3b

White powder, 57 mg, 0.08 mmol. Yield: 22%. ^{1}H NMR (400 MHz, $CDCl_3$): δ (ppm) 7.90 – 7.89 (d, *J* = 1.5 Hz, 2H), 7.81 – 7.79 (d, *J* = 7.5 Hz, 4H), 7.49 – 7.47 (d, *J* = 7.5 Hz, 4H), 7.39 – 7.35 (d, *J* = 7.4, 1.0 Hz, 4H), 7.31 – 7.27 (m, 4H), 7.26 – 7.24 (m, 2H), 7.22 – 7.18 (m, 6H), 6.80 – 6.78 (d, *J* = 8.9 Hz, 4H), 4.25 – 4.20 (q, *J* = 7.2 Hz, 4H), 3.78 (s, 6H), 1.37 – 1.33 (t, *J* = 7.2 Hz, 3H). ^{13}C NMR (100 MHz, $CDCl_3$): δ (ppm) 158.3, 152.4, 140.0, 139.2, 138.9, 136.3, 129.2, 127.7, 127.3, 126.3, 122.7, 120.2, 120.0, 113.6, 108.1, 65.0, 55.2, 37.5, 13.9. HRMS (ion: MALDI, FT-ICR-MS): m/z calcd for [M+H$^+$] $C_{54}H_{41}NO_2$: 735.3137; found: 735.3143

Synthesis of MC derivatives

3-(9-Ethyl-9*H*-carbazol-3-yl)-5-(4-methoxyphenyl)-5*H*-cyclopenta[2,1-b:3,4-b']dipyridin-5-ol (MC1)

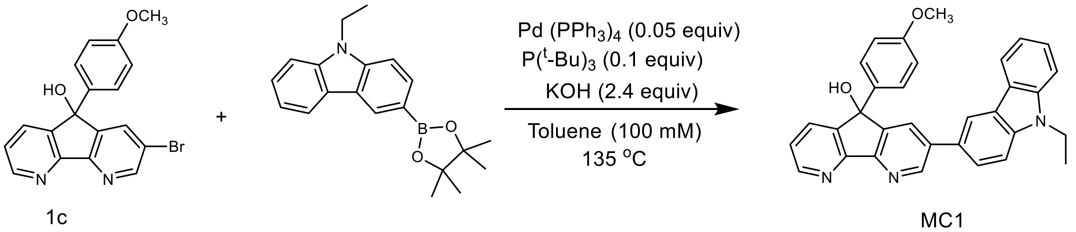

In the nitrogen atmosphere, **1c** (185 mg, 0.5 mmol, 1 equiv) and 9-Ethyl-3-(4,4,5,5-tetramethyl-1,3,2-dioxaborolan-2-yl)-9*H*-carbazole (193 mg, 0.6 mmol, 1.2 equiv), $Pd(PPh_3)_4$ (0.03 g, 0.025 mmol, 0.05 equiv), 0.5 M KOH (2.4 mL, 1.2 mmol, 2.4 equiv), tri-tert-butylphosphine (0.13 ml, 0.05 mmol, 0.1 equiv, 10 wt% in toluene) were mixed in a flask containing with 10 ml of toluene. The mixture was reflux for 3 hours (under about 135 °C), then quenched with water after cooling to room temperature and extracted with DCM. The combined organic solution was dried with anhydrous Na_2SO_4. After filtration the crude product was collected by evaporating the solvent. The crude product was purified by column chromatography [silica gel, petroleum ether:

DCM: tetrahydrofuran = 4:4:1, trimethylamine (2 drops per 100 ml eluent) should be added in whole isolation] and washing with DCM and petroleum ether to give **MC1** (White solid, 126 mg, 0.26 mmol. Yield: 52%). It is noted that the yields of MC1 will be sharply decreased and the substrate **1c** will be massively remained if tri-tert-butylphosphine is not added. Moreover, lower temperature (< 110 ^{o}C) caused to remain **1c** as well. ^{1}H NMR (400 MHz, $CDCl_3$): δ (ppm) 8.81 (s, 1H), 8.45 – 8.43 (d, *J* = 4.6 Hz, 1H), 8.10 – 8.09 (d, *J* = 1.8 Hz, 1H), 8.04 – 8.02 (d, *J* = 7.6 Hz, 1H), 7.94 (d, *J* = 1.4 Hz, 1H), 7.68 – 7.66 (d, *J* = 7.8 Hz, 1H), 7.56 – 7.53 (d, *J* = 8.6, 1.6 Hz, 1H), 7.49 – 7.45 (t, *J* = 7.6 Hz, 1H), 7.37 – 7.33 (m, 3H), 7.24 – 7.21 (m, 2H), 7.10 – 7.06 (dd, *J* = 7.6 Hz, 4.8 Hz, 1H), 6.83 – 6.80 (d, *J* = 8.8 Hz, 2H), 4.16 – 4.10 (m, 2H), 3.745 (s, 3H), 1.34 – 1.31 (t, *J* = 7.2 Hz, 3H). ^{1}H NMR (400 MHz, $CDCl_3$, the protonation of **MC1** by CF_3COOH): δ (ppm) 9.37 (s, 1H), 8.89 (m, 2H), 8.66 – 8.64 (d, *J* = 7.6 Hz, 1H), 8.52 (s, 1H), 8.20 – 8.18 (d, *J* = 7.8 Hz, 1H), 8.11 – 8.09 (m, 1H), 7.84 – 7.82 (d, *J* = 8.9 Hz, 1H), 7.61 – 7.56 (m, 2H), 7.52 – 7.50 (d, *J* = 8.1 Hz, 1H), 7.43 – 7.41 (d, *J* = 8.4 Hz, 2H), 7.37 – 7.33 (t, *J* = 6.2 Hz, 1H), 7.00 – 6.98 (d, *J* = 8.3 Hz, 2H), 4.46 – 4.41 (q, *J* = 6.4 Hz, 2H), 3.87 (s, 3H), 1.51 – 1.48 (t, *J* = 6.7 Hz, 3H). ^{13}C NMR (100 MHz, $CDCl_3$, the protonation of **MC1** by CF_3COOH): δ (ppm) 160.2, 151.3, 150.6, 146.2, 143.7, 143.4, 142.7, 142.1, 141.4, 140.8, 138.5, 129.3, 127.3, 126.5, 124.7, 122.5, 122.1, 120.8, 120.5, 120.0, 115.3, 109.4, 81.0, 55.6, 37.9, 13.4. FT-IR (cm^{-1}): 3182, 3055, 2981, 2938, 2836, 1596, 1555, 1510, 1474, 1453, 1408, 1385, 1380, 1347, 1333, 1299, 1248, 1232, 1168, 1155, 1127, 1106,1088, 1047, 1031, 915, 885, 846, 828, 810, 773, 750, 735, 644. HRMS (ion: MALDI, FT-ICR-MS): m/z calcd for [M+H^{+}] $C_{32}H_{26}N_3O_2$: 484.2020; found: 484.2013.

3-(9-Ethyl-9*H*-carbazol-3-yl)-5-(*p*-tolyl)-5*H*-cyclopenta[2,1-b:3,4-b']dipyridin-5-ol (MC2)

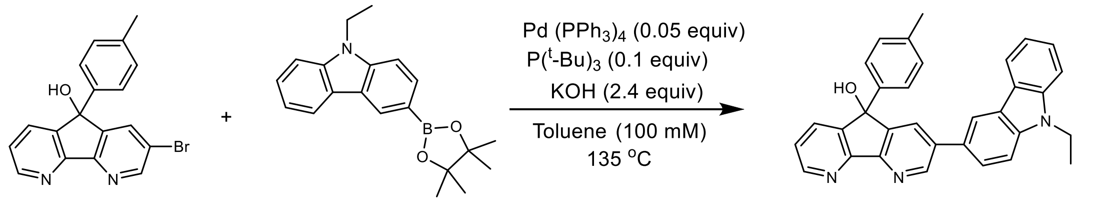

1e MC2

This compound **MC2** was synthesized according to the preparation of **MC1** [substrates: **1e** (180 mg, 0.5 mmol, 1 equiv) and 9-Ethyl-3-(4,4,5,5-tetramethyl-1,3,2-dioxaborolan-2-yl)-9*H*-carbazole (193 mg, 0.6 mmol, 1.2 equiv), $Pd(PPh_3)_4$ (0.03 g, 0.025 mmol, 0.05 equiv), 0.5 M KOH (2.4 mL, 1.2 mmol, 2.4 equiv), tri-tert-butylphosphine (0.13 ml, 0.05 mmol, 0.1 equiv, 10 wt% in toluene)] and purified by column chromatography [silica gel, petroleum ether: DCM: tetrahydrofuran = 4:4:1, trimethylamine (3 drops per 100 ml eluent) should be added in whole isolation] followed by washing with DCM and petroleum ether to give **MC2** (White solid, 88 mg, 0.19 mmol. Yield: 39%). ^{1}H NMR (400 MHz, $CDCl_3$): δ (ppm) 8.82 – 8.81 (d, *J* = 2.0 Hz, 1H), 8.45 – 8.43 (d, *J* = 4.8 Hz, 1H), 8.09 (s, 1H), 8.04 – 8.02 (d, *J* = 7.8 Hz, 1H), 7.94 – 7.93 (d, *J* = 2.0 Hz, 1H), 7.67 – 7.65 (d, *J* = 8.0, 1.6 Hz, 1H), 7.56 – 7.53 (d, *J* = 8.4, 1.6 Hz, 1H), 7.49 – 7.45 (t, *J* = 7.2 Hz, 1H), 7.35 – 7.31 (m, 3H), 7.24 – 7.20 (m,

2H), 7.10 – 7.06 (m, 3H), 4.43 (broad peak, 1H), 4.16 – 4.11 (m, 2H), 2.29 (s, 3H), 1.34 – 1.31 (t, *J* = 7.2 Hz, 1H). ^{1}H NMR (400 MHz, $CDCl_3$, the protonation of **MC2** by CF_3COOH): δ (ppm) 9.37 (d, *J* = 1.2 Hz, 1H), 8.93 (d, *J* = 1.2 Hz, 1H), 8.90 – 8.89 (d, *J* = 5.6 Hz, 1H), 8.71 – 8.69 (d, *J* = 7.6 Hz, 1H), 8.53 (s, 1H), 8.20 – 8.18 (d, *J* = 7.9 Hz, 1H), 8.15 – 8.11 (dd, *J* = 7.8, 5.6 Hz, 1H), 7.84 – 7.81 (dd, *J* = 8.8, 1.7 Hz, 1H), 7.61 – 7.57 (m, 2H), 7.53 – 7.51 (d, *J* = 8.1 Hz, 1H). 7.38 – 7.33 (m, 3H), 7.25 – 7.23 (d, *J* = 7.9 Hz, 2H), 4.49 – 4.43 (q, *J* = 7.3 Hz, 2H), 2.35 (s, 3H), 1.52 – 1.49 (t, *J* = 7.3 Hz, 3H). ^{13}C NMR (100 MHz, $CDCl_3$, the protonation of **MC2** by CF_3COOH): δ (ppm) 151.3, 150.6, 146.4, 143.6, 142.5, 142.2, 141.2, 141.0, 140.9, 138.6, 138.1, 131.5, 130.4, 129.3, 127.3, 124.8, 124.6, 122.5, 121.9, 120.7, 120.5, 120.0, 110.3, 109.3, 81.2, 37.9, 20.5, 13.3. HRMS (ion: MALDI, FT-ICR-MS): m/z calcd for $[M+H^+]$ $C_{32}H_{26}N_3O$: 468.2070; found: 468.2071.

3-Bromo-7-(9-ethyl-9*H*-carbazol-3-yl)-5-(4-methoxyphenyl)-5*H*-cyclopenta[2,1-b:3,4-b']dipyridin-5-ol (MC3)

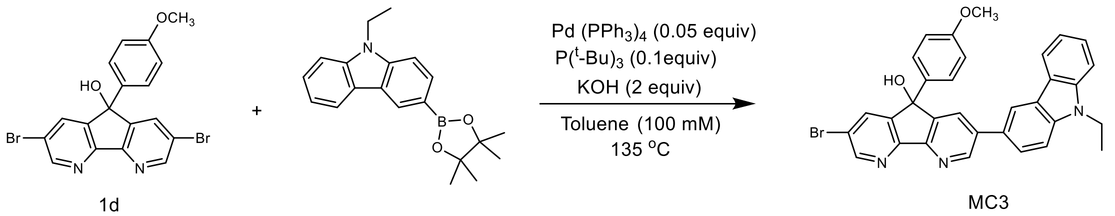

In the nitrogen atmosphere, **1d** (1350 mg, 3 mmol, 1 equiv) and 9-Ethyl-3-(4,4,5,5-tetramethyl-1,3,2-dioxaborolan-2-yl)-9*H*-carbazole (960 mg, 3 mmol, 1 equiv), $Pd(PPh_3)_4$ (180 mg, 0.15 mmol, 0.05 equiv), 2 M KOH (3 mL, 6 mmol, 2 equiv), tri-tert-butylphosphine (0.75 ml, 0.3 mmol, 0.1 equiv, 10 wt% in toluene) were mixed in a flask containing with 10 ml of toluene (under about 135 °C). The mixture was reflux for 3.5 hours, then quenched with water after cooling to room temperature and extracted with DCM. The combined organic solution was dried with anhydrous Na_2SO_4. After the filtration, the crude product was collected by evaporating the solvent. The crude product was purified by column chromatography [silica gel, trimethylamine (2 drops per 100 ml eluent) should be added in whole isolation] to give **MC3** (Eluent: petroleum ether: DCM: tetrahydrofuran = 12:12:1). Yellow solid, 473 mg, 0.84 mmol. Yield: 28%. ^{1}H NMR (400 MHz, $CDCl_3$): δ (ppm) 8.90 – 8.89 (d, *J* = 2.1 Hz, 1H), 8.58 (d, *J* = 2.1 Hz, 1H), 8.11 (d, *J* = 1.3 Hz, 1H), 8.04 – 8.02 (d, *J* = 7.9 Hz, 1H), 7.94 – 7.93 (d, *J* = 1.9 Hz, 1H), 7.81 (d, *J* = 1.9 Hz, 1H), 7.58 – 7.56 (dd, *J* = 8.4, 2.0 Hz, 1H), 7.50 – 7.45 (t, *J* = 7.2 Hz, 1H), 7.37 – 7.34 (m, 3H), 7.24 – 7.21 (m, 2H), 6.84 – 6.82 (d, *J* = 8.8 Hz, 2H), 4.16 – 4.12 (m, 2H), 3.77 (s, 3H), 1.35 – 1.32 (m, 3H). ^{1}H NMR (400 MHz, $CDCl_3$, the protonation of **MC3** via CF_3COOH): δ (ppm) 9.27 (s, 1H), 8.90 (s, 2H), 8.54 (s, 1H), 8.49 (s, 1H), 8.18 – 8.16 (d, *J* = 7.7 Hz, 1H), 7.82 – 7.80 (d, *J* = 8.2 Hz, 1H), 7.61 – 7.56 (m, 2H), 7.53 – 7.51 (d, *J* = 7.6 Hz, 1H), 7.44 – 7.41 (d, *J* = 8.8 Hz, 2H), 7.37 – 7.33 (t, *J* = 7.7 Hz, 1H), 7.03 – 7.01 (d, *J* = 8.8 Hz, 2H), 4.48 – 4.43 (q, *J* =7.3 Hz, 2H), 3.90 (s, 3H), 1.52 – 1.48 (t, *J* = 7.2 Hz, 3H). ^{13}C NMR (100 MHz, $CDCl_3$, the protonation of **MC3** via CF_3COOH): δ (ppm) 160.1, 150.6, 150.3, 147.4, 145.5, 143.9, 143.0, 142.0, 140.9, 140.0, 139.1, 127.8, 127.3, 126.5, 126.0, 124.7, 124.5, 122.5,

120.7, 120.4, 119.8, 115.4, 110.3, 109.3, 80.8, 55.6, 37.8, 13.3. HRMS (ion: MALDI, FT-ICR-MS): m/z calcd for [M+H$^+$] $C_{32}H_{25}BrN_3O_2$: 562.1125; found: 562.1124.

3-(9,9-Dioctyl-*9H*-fluoren-2-yl)-7-(9-ethyl-9*H*-carbazol-3-yl)-5-(4-methoxyphenyl)-5*H*-cyclopenta[2,1-b:3,4-b']dipyridin-5-ol (MC4)

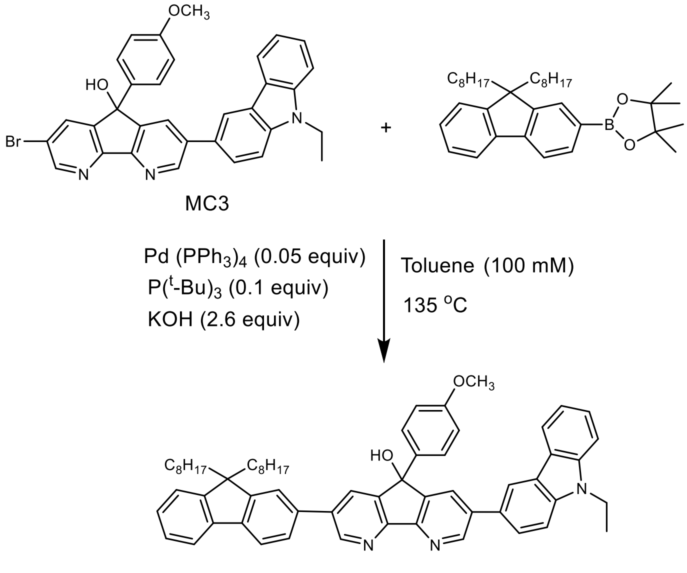

In the nitrogen atmosphere, **MC3** (300 mg, 0.54 mmol, 1 equiv) and 2-(9,9-Dioctyl-9*H*-fluoren-2-yl)-4,4,5,5-tetramethyl-1,3,2-dioxaborolane (360 mg, 0.7 mmol, 1.3 equiv), $Pd(PPh_3)_4$ (30 mg, 0.027 mmol, 0.05 equiv), 2 M KOH (0.7 mL, 1.4 mmol, 2.6 equiv), tri-tert-butylphosphine (0.13 ml, 0.06 mmol, 0.1 equiv, 0.1wt% in toluene) were mixed in a flask containing with 5 ml of toluene. The mixture was reflux for 3 hours (under about 135 ^oC), then quenched with water after cooling to room temperature and extracted with DCM. The combined organic solution was dried with anhydrous Na_2SO_4. After the filtration, the crude product was collected by evaporating the solvent. The crude product was purified by column chromatography [silica gel, eluent: petroleum ether: DCM: ethyl acetate = 4:4:1, trimethylamine (2 drops per 100 ml eluent) should be added in whole isolation] to give **MC4** (Yellow solid, 230 mg, 0.26 mmol. Yield: 49%) ^{1}H NMR (400 MHz, $CDCl_3$): δ (ppm) 8.64 (s, 1H), 8.48 (s, 1H), 7.96 – 7.92 (m, 4H), 7.48 – 7.44 (m, 5H), 7.35 – 7.32 (m, 4H), 7.29 – 7.27 (d, J = 8.4 Hz, 1H), 7.21 – 7.16 (m, 2H), 7.01 – 6.99 (d, J = 7.6 Hz, 1H), 6.94 – 6.92 (d, J = 8.5 Hz, 1H), 6.85 – 6.83 (d, J = 8.9 Hz, 2H), 3.93 – 3.82 (m, 2H), 3.74 (s, 3H), 2.00 – 1.93 (q, J = 8.0 Hz, 4H), 1.21 – 0.98 (m, 27H), 0.83 – 0.79 (t, J = 7.1 Hz, 3H), 0.78 – 0.74 (t, J = 7.1 Hz, 3H), 0.67 – 0.61 (m, 4H). ^{13}C NMR (100 MHz, $CDCl_3$): δ (ppm) 159.1, 155.2, 151.8, 151.0, 149.4, 149.3, 146.5, 141.1, 140.3, 140.1, 139.5, 137.8, 137.2, 136.0, 133.6, 130.6, 127.6, 127.3, 126.9, 126.8, 126.0, 125.9, 124.5, 123.4, 123.0, 122.7, 121.1, 120.5, 120.1, 119.9, 119.1, 118.6, 113.9, 108.6, 108.4, 79.2, 55.2, 40.3, 37.2, 31.8, 30.1, 29.2, 24.0, 22.6, 14.1, 13.7. MALDI-Tof-MS: m/z calcd for [M+H$^+$] $C_{61}H_{65}N_3O_2$: 872.515; found: 872.528.

3-(3',6'-Bis(octyloxy)spiro[fluorene-9,9'-xanthen]-2-yl)-7-(9-ethyl-9*H*-carbazol-3-yl)-5-(4-methoxyphenyl)-5*H*-cyclopenta[2,1-b:3,4-b']dipyridin-5-ol (MC5)

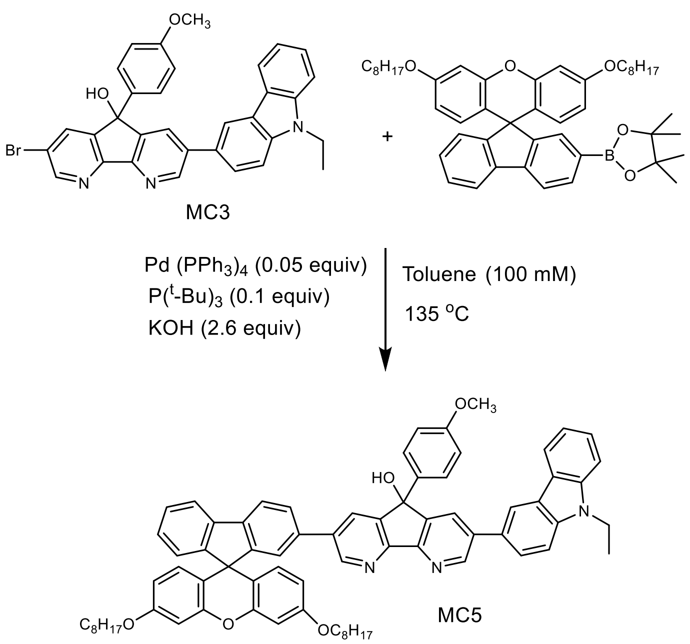

In the nitrogen atmosphere, **MC3** (300 mg, 0.54 mmol, 1 equiv) and 2-(3',6'-Bis(octyloxy)spiro[fluorene-9,9'-xanthen]-2-yl)-4,4,5,5-tetramethyl-1,3,2-dioxaborolane (460 mg, 0.65 mmol, 1.2 equiv), $Pd(PPh_3)_4$ (30 mg, 0.027 mmol, 0.05 equiv), 2 M KOH (0.7 mL, 1.4 mmol, 2.6 equiv), tri-tert-butylphosphine (0.13 ml, 0.06 mmol, 0.1 equiv, 0.1wt% in toluene) were mixed in a flask containing with 5 ml of toluene. The mixture was reflux for 3 hours (under about 135 °C), then quenched with water after cooling to room temperature and extracted with DCM. The combined organic solution was dried with anhydrous Na_2SO_4. After the filtration, the crude product was collected by evaporating the solvent. The crude product was purified by column chromatography [silica gel, eluent: petroleum ether: DCM: ethyl acetate = 4:4:1, trimethylamine (2 drops per 100 ml eluent) should be added in whole isolation] to give **MC5** (Yellow solid, 250 mg, 0.23 mmol. Yield: 43%) ^{1}H NMR (400 MHz, $CDCl_3$): δ (ppm) 8.59 (d, *J* = 1.2 Hz, 1H), 8.27 (d, *J* = 1.2 Hz, 1H), 7.97 – 7.95 (d, *J* = 8.0 Hz, 1H), 7.93 (s, 1H), 7.85 – 7.84 (d, *J* = 1.7 Hz, 1H), 7.74 (d, *J* = 1.7 Hz, 1H), 7.57 – 7.55 (d, *J* = 7.6 Hz, 1H), 7.47 – 7.44 (t, *J* = 7.2 Hz, 2H), 7.35 – 7.32 (d, *J* = 8.9 Hz, 4H), 7.30 – 7.27 (m, 2H), 7.22 – 7.17 (m, 3H), 7.11 – 7.09 (d, *J* = 7.6 Hz, 1H), 6.99 – 6.97 (d, *J* = 8.5 Hz, 1H), 6.78 – 6.76 (d, *J* = 8.9 Hz, 2H), 6.73 – 6.72 (m, 2H), 6.31 – 6.28 (dd, *J* = 8.8, 2.3 Hz, 1H), 6.23 – 6.20 (d, *J* = 8.7 Hz, 1H), 6.18 – 6.15 (m, 2H), 5.81 (s, 1H), 3.92 – 3.84 (m, 6H), 3.71 (s, 3H), 1.77 – 1.71 (m, 4H), 1.45 – 1.29 (m, 24H), 1.19 – 1.15 (t, *J* = 7.2 Hz, 3H), 0.92 – 0.86 (m, 7H). ^{13}C NMR (100 MHz, $CDCl_3$): δ (ppm) 159.0, 156.3, 155.4, 154.1, 152.0, 149.4, 149.2, 146.7, 146.2, 140.1, 140.0, 139.5, 138.3, 138.0, 137.7, 137.4, 136.5, 133.3, 130.6, 130.4, 128.7, 128.6, 128.5, 127.6, 126.9, 126.8, 125.9, 125.6, 124.4, 124.1, 123.3, 122.7, 120.5, 120.2, 119.9, 119.1, 118.6, 116.0, 113.9, 111.1, 110.9, 108.5, 101.9, 101.8, 79.1, 68.1, 55.2, 53.6, 37.2, 31.9, 29.4, 29.2, 26.1, 22.7, 14.2, 13.6. MALDI-ToF-MS: m/z calcd for $[M+H^+]$ $C_{73}H_{71}N_3O_5$: 1070.547; found: 1070.567.

Gridization from A_1B_1-typed MC1 derivatives

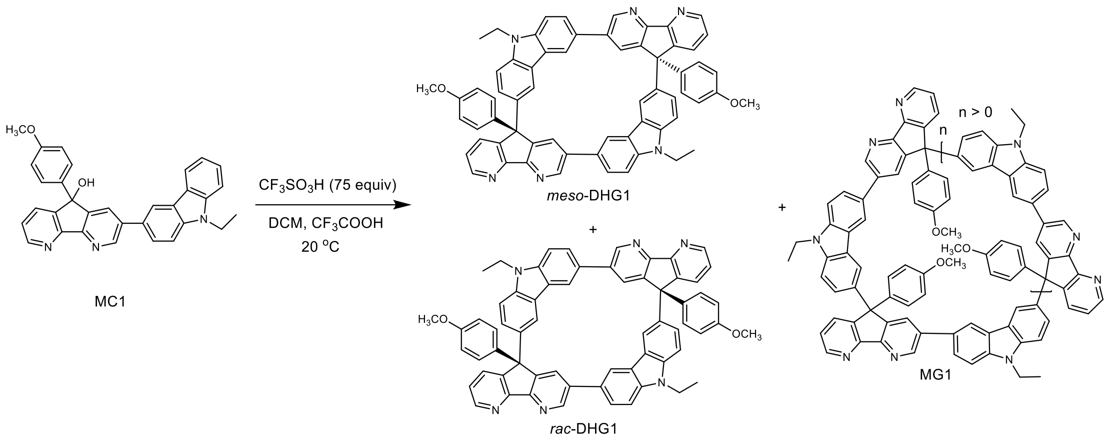

MC1 (140 mg, 0.3 mmol, 1 equiv) was added in 22.5 ml DCM and 7.5 ml CF_3COOH mixed solvents and then the solution was stirred (under 20 ^oC). Then CF_3SO_3H (2 ml, 22.8 mmol, 75 equiv) were added quickly. After 1 min, the excessive solution of potassium hydroxide was added very slowly at the temperature of 0 ^oC and then extracted with DCM. The mixed organic phase was dried over anhydrous Na_2SO_4, and then the organic phase was filtered and concentrated under low pressure. The crude product was purified by column chromatography [silica gel, trimethylamine (2 drops per 100 ml eluent) should be added in whole isolation] to give ***meso*-DHG1** (Eluent: DCM: ethyl acetate = 2:1), ***rac*-DHG1** (Eluent: DCM: ethyl acetate = 2:1) and the byproducts **MG1** (oligomer cycles, a series of homolog, Eluent: DCM: methyl alcohol = 10:1). It is noted that the trimethylamine is mixed with MG1 and is very difficult to remove.

***meso*-DHG1**

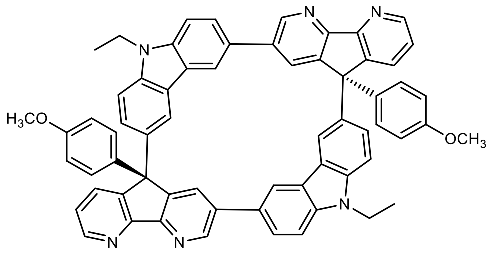

meso-DHG1

White solid, 97 mg, 0.10 mmol. Yield: 69%. ^1H NMR (400 MHz, $CDCl_3$): δ (ppm) 8.96 – 8.95 (d, *J* = 1.8 Hz, 2H), 8.80 – 8.78 (dd, *J* = 5.0 Hz, 1.3 Hz, 2H), 8.60 (d, *J* = 2.1 Hz, 2H), 8.30 – 8.29 (m, 4H), 8.01 – 7.98 (dd, *J* = 7.8 Hz, 1.4 Hz, 2H), 7.74 – 7.72 (dd, *J* = 8.2 Hz, 2.2 Hz, 2H), 7.63 – 7.60 (dd, *J* = 8.6 Hz, 1.7 Hz, 2H), 7.52 – 7.50 (d, *J* = 9.0 Hz, 2H), 7.39 – 7.36 (m, 4H), 6.79 – 6.77 (d, *J* = 9.0 Hz, 4H), 6.63 – 6.61 (d, *J* = 9.0 Hz, 4H), 4.44 – 4.38 (q, *J* = 7.2 Hz, 4H), 3.65 (s, 6H), 1.49 – 1.45 (q, *J* = 7.2 Hz, 6H). Because of its extremely poor solubility, its ^{13}C NMR cannot be obtained easily. Fortunately, its solubility is increased if adding CF_3COOH in $CDCl_3$. ^1H NMR (400 MHz, $CDCl_3$, the protonation of ***meso*-DHG1** via CF_3COOH): δ (ppm) 9.15 (s, 2H), 9.04 – 9.01 (m, 4H), 8.66 – 8.65 (d, *J* = 1.4 Hz, 2H), 8.35 – 8.31 (m, 4H), 7.81 – 7.79 (d, *J* = 9.5 Hz, 2H), 7.70 – 7.67 (d, *J* = 8.9 Hz, 2H), 7.59 – 7.57 (d, *J* = 8.9 Hz, 2H), 7.45 – 7.42 (dd, *J* = 8.9, 1.3 Hz, 2H), 6.93 – 6.91 (d, *J* = 9.1 Hz, 4H), 6.86 – 6.84 (d, *J*

= 9.0 Hz, 4H). 4.51 – 4.45 (q, *J* = 7.2 Hz, 4H), 3.80 (s, 6H), 1.52 – 1.49 (t, *J* = 7.4 Hz, 6H). ^{13}C NMR (100 MHz, $CDCl_3$, the protonation of ***meso*-DHG1** via CF_3COOH): δ (ppm) 159.5, 152.5, 152.0, 145.1, 143.4, 142.3, 142.1, 141.7, 141.0, 140.0, 131.1, 128.7, 128.6, 127.3, 126.0, 124.5, 123.5, 123.3, 121.1, 120.2, 115.4, 110.7, 110.3, 63.6, 55.4, 38.1, 13.0. FT-IR (cm^{-1}): 3050, 2967, 2929, 2873, 1602, 1582, 1553, 1510, 1482, 1456, 1408, 1381, 1347, 1310, 1294, 1248, 1236, 1183, 1157, 1130, 1098, 1087, 1026, 880, 852, 828, 813, 786, 749, 636, 621. HRMS (ion: MALDI, FT-ICR-MS): m/z calculated: for [$M+H^+$] $C_{64}H_{47}N_6O_2$: 931.3755; found: 931.3751.

***rac*-DHG1**

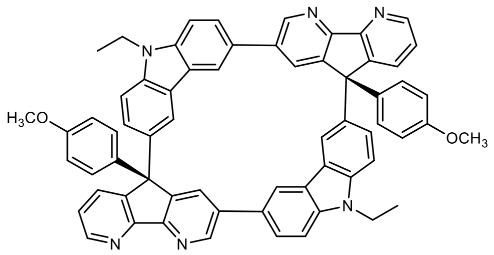

rac-DHG1

Yellow-White solid, 11 mg, 0.012 mmol. Yield: 8%. ^{1}H NMR (400 MHz, $CDCl_3$): δ (ppm) 8.97 (s, 2H), 8.78 – 8.77 (d, *J* = 4.8 Hz, 2H), 8.62 (s, 2H), 8.44 (m, 4H), 7.96 – 7.94 (d, *J* = 7.5 Hz, 2H), 7.70 – 7.60 (broad peak, 2H), 7.46 – 7.44 (d, *J* = 7.2 Hz, 2H), 7.37 – 7.34 (dd, *J* = 7.2, 5.2 Hz, 2H), 7.26 – 7.15 (m, 4H), 7.06 – 7.04 (d, *J* = 9.0 Hz, 4H), 6.76 – 6.74 (d, *J* = 8.7 Hz, 4H), 4.30 – 4.20 (broad peak, 4H), 3.72 (s, 6H), 1.25 (m, 6H). ^{13}C NMR (100 MHz, $CDCl_3$): δ (ppm) 158.7, 147.7, 146.4, 142.7 140.5, 139.8, 137.6, 136.1, 134.4, 132.6, 131.5, 129.0, 125.4, 123.5, 122.9, 121.5, 118.5, 114.0, 109.1, 108.2, 60.7, 55.2, 37.7, 13.7. FT-IR (cm^{-1}): 2957, 2925, 2868, 2853, 1728, 1606, 1580, 1508, 1484, 1455, 1409, 1375, 1345, 1294, 1262, 1233, 1178, 1158, 1101, 1083, 1024, 803, 746, 633. HRMS (ion: MALDI, FT-ICR-MS): m/z calculated: for [$M+H^+$] $C_{64}H_{47}N_6O_2$: 931.3755; found: 931.3751.

MG1 (a series of macrogrid homologs)

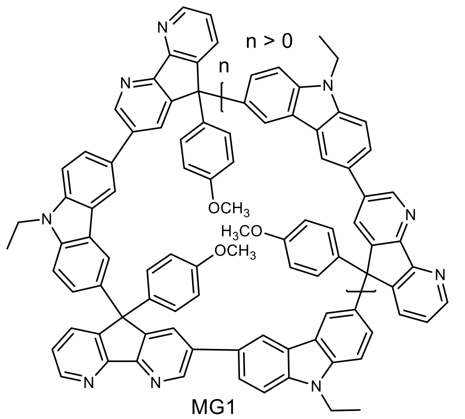

MG1

Yellow-orange powder, 28 mg, Yield: 20%. ^{1}H NMR (400 MHz, $CDCl_3$): δ (ppm) 9.01 – 8.96 (2.00 H), 8.72 – 8.59 (2.03 H), 8.15 – 8.09 (1.38 H), 8.06 – 8.02 (1.74 H), 7.96 – 7.88 (1.20 H), 7.86 – 7.81 (1.79 H), 7.58 – 7.51 (2.44 H), 7.44 – 7.33 (4.36 H),

7.19 – 7.16 (1.50 H), 6.80 – 6.70 (2.16 H), 4.33 – 4.22 (3.55 H), 3.75 – 3.66 (5.31 H), 1.50 (4.43 H),. The above integration was relative ratio. ^{13}C NMR (100 MHz, $CDCl_3$): δ (ppm), 158.9, 157.2, 155.4, 149.9, 149.0, 149.0, 147.1, 147.0, 140.2, 139.6, 137.7, 136.0, 134.7, 133.8, 131.9, 129.0, 128.8, 127.3, 125.6, 123.3, 122.7, 119.5, 119.2, 114.0, 109.2, 109.0, 61.0, 55.2, 37.7, 13.8. **This compound was mixed with trimethylamine as well (extremely difficult to be removed by solvent washing and reduced pressure distillation), which contained its ethyl signals at 3.11 – 3.05 (q, *J* = 7.2 Hz) and 1.40 – 1.37 (t, *J* = 7.3 Hz) ppm in ^{1}H NMR as well as the 46.0, 8.6 ppm in ^{13}C NMR spectra. These signals were also observed in other MG-based derivatives.** FT-IR (cm^{-1}): 2978, 2937, 1605, 1584, 1509, 1479, 1444, 1399, 1384, 1291, 1260, 1170, 1161, 1098, 1072, 1032, 851, 806, 748, 638. **The C-H bands in the range of 2750~2400 cm^{-1} was assigned to triethylamine.**

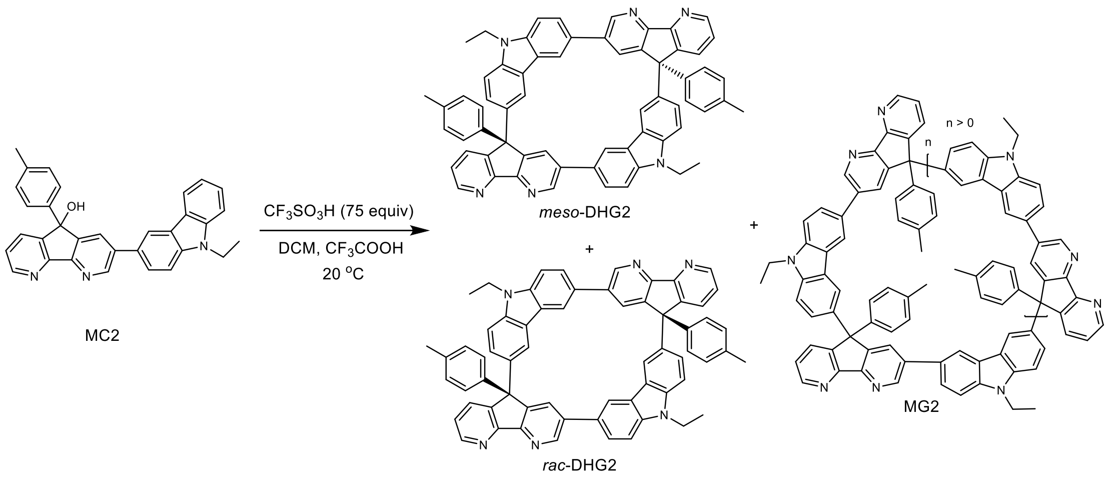

MC2 (140 mg, 0.3 mmol, 1 equiv) was added in 22.5 ml DCM solvent and 7.5 ml CF_3COOH mixed solvents and then the solution was stirred (under 20 °C). Then CF_3SO_3H (2 ml, 22.8 mmol, 75 equiv) were added quickly. After the reagent **MC2** was completely consumed (in 1 minute) as monitored by TLC, the excessive solution of potassium hydroxide was added very slowly at the temperature of 0 °C and then extracted with DCM. The mixed organic phase was dried over anhydrous Na_2SO_4 and then this organic phase was filtered and concentrated under low pressure. The crude product was purified by column chromatography [silica gel, trimethylamine (2 drops per 100 ml eluent) should be added in whole isolation] to give ***meso*-DHG2** (Eluent: DCM: ethyl acetate = 2:1), ***rac*-DHG2** (Eluent: DCM: ethyl acetate = 2:1) and the byproducts **MG2** (oligomer cycles, a series of homolog, Eluent: DCM: methyl alcohol = 10:1). It is noted that no linear irregular oligomers were observed.

***meso*-DHG2**

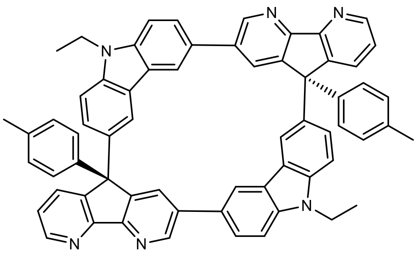

meso-DHG2

White powder, 20 mg, 0.022mmol. Yield: 15%. ^{1}H NMR (400 MHz, $CDCl_3$): δ (ppm) 8.95 – 8.94 (d, *J* = 1.8 Hz, 2H), 8.80 – 8.78 (dd, *J* = 4.8, 1.3 Hz, 2H), 8.58 (d, *J* = 1.6 Hz, 2H), 8.29 (d, *J* = 1.8 Hz, 2H), 8.27 (d, *J* = 1.4 Hz, 2H), 8.01 – 7.98 (dd, *J* = 7.7, 1.3 Hz, 2H), 7.74 – 7.71 (dd, *J* = 8.4, 1.6 Hz, 2H), 7.62 – 7.59 (dd, *J* = 8.7, 1.8 Hz, 2H), 7.52 – 7.49 (dd, *J* = 8.5 Hz, 2H), 7.38 – 7.35 (m, 4H), 6.91 – 6.89 (d, *J* = 8.2 Hz, 4H), 6.78 – 6.75 (d, *J* = 8.3 Hz, 4H), 4.43 – 4.39 (m, 4H), 2.19 (s, 6H), 1,48 – 1.45 (t, *J* = 7.0 Hz, 6H). Because of its extremely poor solubility, its ^{13}C NMR cannot be obtained easily. Fortunately, its solubility is increased if adding CF_3COOH in $CDCl_3$. ^{1}H NMR (400 MHz, $CDCl_3$, the protonation of ***meso*-DHG2** via CF_3COOH): δ (ppm) 9.30 – 9.25 (2H), 9.08 – 8.85 (6H), 8.40 – 8.25 (6H), 7.88 – 7.82 (2H), 7.74 – 7.67 (2H), 7.57 – 7.52 (2H), 7.45 – 7.37 (2H), 7.13 – 7.05 (4H), 6.90 – 6.83 (4H), 4.46 (4H), 2.25 – 2.24 (6H), 1.52 – 1.47 (m, 6H). These are broad peaks. ^{13}C NMR (100 MHz, $CDCl_3$, the protonation of ***meso*-DHG2** via CF_3COOH): δ (ppm) 152.4, 151.8, 147.3, 145.5, 145.0, 143.5, 142.5, 142.1, 142.0, 141.4, 140.9, 140.2, 135.7, 135.5, 132.9, 130.6, 130.4, 128.5, 127.8, 127.4, 127.0, 126.8, 125.9, 125.7, 124.4, 123.3, 123.2, 120.9, 120.1, 117.3, 115.3, 113.5, 111.6, 110.6, 110.2, 63.8, 38.0, 20.5, 20.0, 13.0, 12.9. HRMS (ion: MALDI, FT-ICR-MS): m/z calculated: for $[M+H^+]$ $C_{64}H_{47}N_4$: 899.3857; found: 899.3851.

***rac*-DHG2**

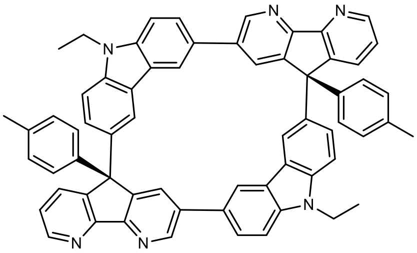

rac-DHG2

Yellow-white powder, 46 mg, 0.051mmol. Yield: 26%. ^{1}H NMR (400 MHz, $CDCl_3$): δ (ppm) 9.14 (s, 2H), 8.76 – 8.75 (d, *J* = 4.8 Hz, 2H), 8.58 (d, *J* = 1.5 Hz, 2H), 8.51 (s, 2H), 8.47 (s, 2H), 8.01 – 7.99 (d, *J* = 7.9 Hz, 2H), 7.84 – 7.82 (d, *J* = 7.6 Hz, 2H), 7.43 – 7.40 (dd, *J* = 7.4 Hz, 4.6 Hz, 4H), 7.34 – 7.31 (d, *J* = 7.6 Hz, 2H), 7.09 – 7.03 (m, 8H), 4.30 (broad peak, 4H), 2.29 (s, 3H), 2.27 (s, 3H). 1.40 – 1.35 (m, 6H). ^{13}C NMR (100 MHz, $CDCl_3$): δ (ppm) 149.7, 147.3, 146.8, 146.2, 142.8, 140.5, 139.8, 137.2, 135.9, 135.8, 134.6, 132.9, 131.3, 129.6, 127.7, 125.5, 125.2, 124.4, 123.6, 123.3, 123.1, 121.6, 118.1, 109.0, 108.1, 61.2, 37.5, 34.3, 30.3, 29.7, 21.1, 13.6. HRMS (ion: MALDI, FT-ICR-MS): m/z calc for $[M+H^+]$ $C_{64}H_{47}N_4$: 899.3857; found: 899.3848.

MG2 (a series of macrogrid homologs)

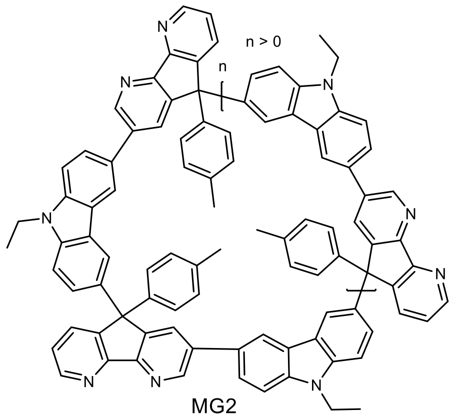

MG2

Yellow powder, 66 mg. Yield: 47%. ^{1}H NMR (400 MHz, $CDCl_3$): δ (ppm) 9.05 – 8.85 (1.74H), 8.73 – 8.57 (2.00H), 8.20 – 8.05 (3.49H), 7.96 – 7.83 (3.78H), 7.60 – 7.52 (2.43H), 7.43 – 7.35 (3.53H), 7.19 – 7.12 (6.13H), 7.09 – 6.97 (5.91H), 4,34 – 4.21 (4.46H), 2.36 – 2.15 (7.60H), 1.24 (8.09 H). The above integration was relative ratio. ^{13}C NMR (100 MHz, $CDCl_3$): δ (ppm) 157.0, 155.2, 149.7, 148.9, 147.1, 147.0, 141.1, 140.2, 139.6, 139.0, 137.8, 137.2, 134.5, 134.1, 134.0, 133.4, 132.1, 129.4, 128.7, 127.8, 126.4, 125.6, 123.5, 123.4, 123.2, 122.7, 122.1, 119.5, 119.2, 118.9, 109.3, 109.0, 46.2, 37.8, 30.3, 24.9, 20.9, 13.8.

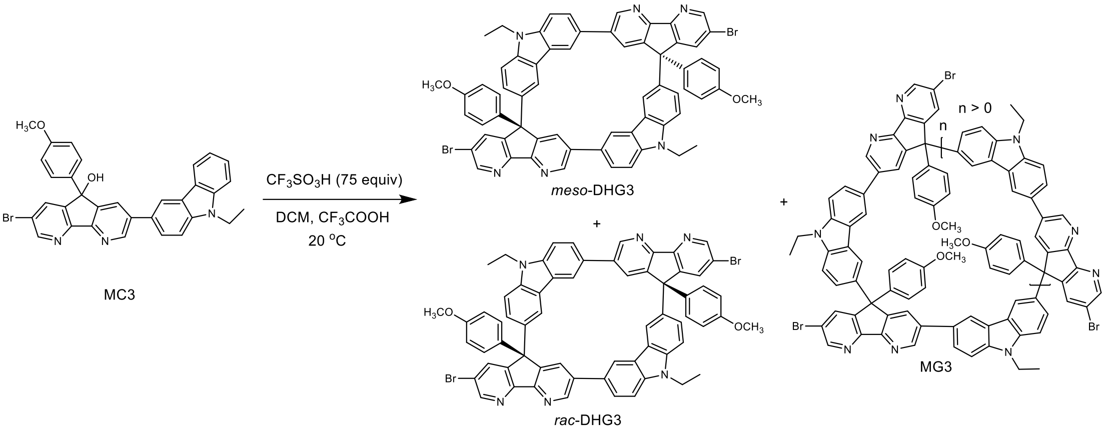

MC3 (170 mg, 0.3 mol, 1 equiv) was added in 20 ml DCM solvent and 7 ml CF_3COOH mixed solvents and then the solution was stirred (under 20 °C). Then CF_3SO_3H (2 ml, 22.8 mmol, 75 equiv) were added quickly. After the reagent **MC3** was completely consumed (in 1 minute) as monitored by TLC, the excessive solution of potassium hydroxide was added very slowly at the temperature of 0 °C and then extracted with DCM. The mixed organic phase was dried over anhydrous Na_2SO_4 and then the organic phase was filtered and concentrated under low pressure. The crude product was purified by column chromatography [silica gel, trimethylamine (2 drops per 100 ml eluent) should be added in whole isolation] to give ***meso*-DHG3** (Eluent: DCM: ethyl acetate = 3:1), ***rac*-DHG3** (Eluent: DCM: ethyl acetate = 2:1) and the

byproducts **MG3** (oligomer cycles, a series of homolog, Eluent: DCM: methyl alcohol = 10:1).

***meso*-DHG3**

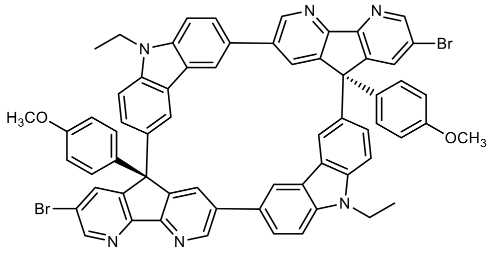

meso-DHG3

White powder, 99 mg, 0.09 mmol. Yield: 61%. ^{1}H NMR (400 MHz, $CDCl_3$): δ (ppm) 8.95 (d, *J* = 2.0 Hz, 2H), 8.85 – 8.84 (d, *J* = 2.1 Hz, 2H), 8.54 (d, *J* = 1.8 Hz, 2H), 8.27 – 8.26 (m, 4H), 8.10 – 8.09 (d, *J* = 2.0 Hz, 2H), 7.74 – 7.72 (dd, *J* = 8.4 Hz, 1.8 Hz, 2H), 7.58 – 7.55 (dd, *J* = 8.7 Hz, 2.0 Hz, 2H), 7.53 – 7.51 (d, *J* = 8.8 Hz, 2H), 7.43 – 7.41 (d, *J* = 8.4 Hz, 2H), 6.79 – 6.77 (d, *J* = 9.0 Hz, 4H), 6.65 – 6.63 (d, *J* = 9.0 Hz, 4H). 4.45 – 4.39 (q, *J* = 7.2 Hz, 4H), 3.67 (s, 6H), 1.50 – 1.46 (t, *J* = 7.2 Hz, 6H). Because of its extremely poor solubility, its ^{13}C NMR cannot be obtained easily. Fortunately, its solubility is increased if adding CF_3COOH in $CDCl_3$. ^{1}H NMR (400 MHz, $CDCl_3$, the protonation of ***meso*-DHG3** via CF_3COOH): δ (ppm) 9.19 (s, 2H), 9.08 – 9.05 (m, 4H), 8.71 (s, 2H), 8.39 – 8.34 (m, 4H), 7.85 – 7.83 (d, *J* = 7.6 Hz, 2H), 7.74 – 7.72 (d, *J* = 8.3 Hz, 2H), 7.63 – 7.61 (d, *J* = 8.4 Hz, 2H), 7.49 – 7.47 (d, *J* = 7.3 Hz, 2H), 6.96 – 6.94 (d, *J* = 8.8 Hz, 4H), 6.90 – 6.87 (d, *J* = 8.5 Hz, 4H), 4.55 – 4.48 (m, 4H), 3.83 (s, 6H), 1.53 (m, 6H). ^{13}C NMR (100 MHz, $CDCl_3$, the protonation of ***meso*-DHG3** via CF_3COOH): δ (ppm) 159.5, 152.5, 152.0, 145.8, 145.1, 143.4, 142.6, 142.3, 142.2, 141.7, 141.0, 140.0, 131.1, 128.7, 128.6, 126.0, 125.9, 124.5, 123.3, 121.1, 120.2, 115.4, 110.7, 110.3, 63.6, 55.4, 38.1, 13.0. HRMS (ion: MALDI, FT-ICR-MS): m/z calcd for $[M+H^+]$ $C_{64}H_{45}Br_2N_6O_2$: 1087.1965; found: 1087.1977.

***rac*-DHG3**

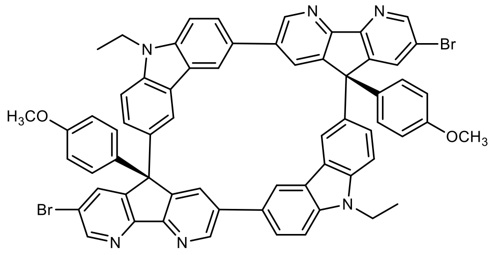

rac-DHG3

Yellow-white powder, 13 mg, 0.012 mmol. Yield: 8%. ^{1}H NMR (400 MHz, $CDCl_3$): δ (ppm) 9.02 (s, 2H), 8.82 – 8.81 (d, *J* = 2.0 Hz, 2H), 8.59 – 8.58 (d, *J* = 1.5 Hz, 2H), 8.44 – 8.43 (d, *J* = 1.5 Hz, 2H), 8.41 (d, *J* = 1.8 Hz, 2H), 8.05 – 8.04 (d, *J* = 2.0 Hz, 2H), 7.71 – 7.69 (d, *J* = 9.0 Hz, 2H), 7.44 – 7.41 (dd, *J* = 8.6 Hz, 1.7 Hz, 2H), 7.31 – 7.27 (m, 4H), 7.04 – 7.02 (d, *J* = 8.9 Hz, 4H), 6.78 – 6.76 (d, *J* = 8.9 Hz, 4H), 4.22 – 4.17 (q, *J* = 7.2 Hz, 4H), 3.73 (s, 6H), 1.34 – 1.30 (m, 6H). ^{13}C NMR (100 MHz, $CDCl_3$): δ (ppm) 159.3, 151.8, 151.5, 149.0, 144.6, 143.7, 143.0, 142.3, 142.2, 140.9, 139.7, 132.1, 128.7, 127.9, 126.1, 125.8, 125.6, 124.5, 123.4, 123.3, 120.9, 120.2, 115.4,

110.6, 110.1, 100.0, 63.1, 55.5, 38.1, 13.1. HRMS (ion: MALDI, FT-ICR-MS): m/z calcd for [M+H$^+$] $C_{64}H_{45}Br_2N_6O_2$: 1087.1965; found: 1087.1964.

MG3 (a series of macrocyclic homologs)

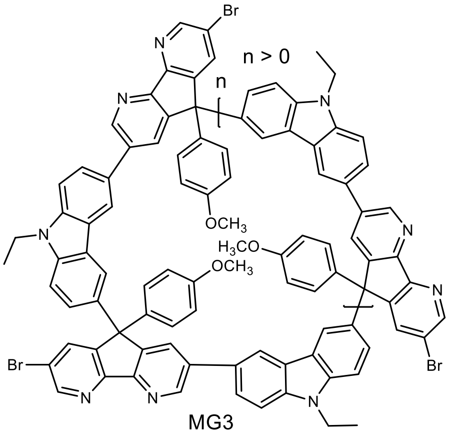

MG3

Yellow-brown powder, 51 mg. Yield: 30%. ^{1}H NMR (400 MHz, $CDCl_3$): δ (ppm) 9.10 – 9.00 (2.00 H), 8.85 – 8.75 (1.91 H), 8.25 – 7.90 (7.25 H), 7.70 - 7.55 (2.56 H), 7.50 – 7.28 (5.49 H), 7.25 – 7.10 (1.96 H), 6.90 – 6.75 (3.17 H), 4.40 – 4.20 (3.74 H), 3.86 – 3.75 (6.12 H). The above integration was relative ratio. ^{13}C NMR (100 MHz, $CDCl_3$): δ (ppm) 159.1, 156.0, 154.4, 151.2, 149.6, 148.6, 147.1, 140.3, 139.7, 138.0, 136.3, 134.1, 131.8, 130.6, 129.1, 128.9, 127.3, 125.7, 123.2, 122.9, 119.7, 119.4, 114.2, 113.5, 109.3, 109.2, 100.0, 61.1, 55.3, 37.8, 32.0, 29.7, 29.1, 24.9, 13.8.

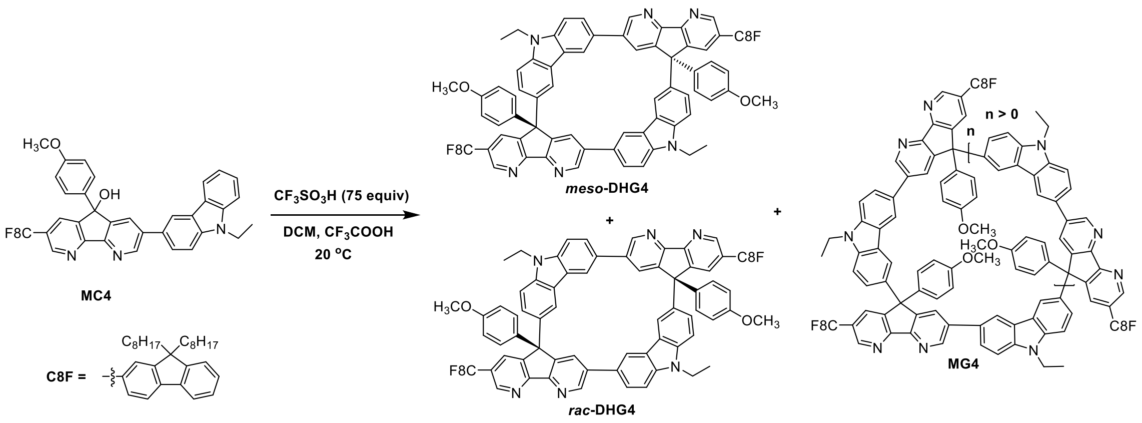

MC4 (200 mg, 0.23 mmol, 1 equiv) was added in 15 ml DCM solvent and 5 ml CF_3COOH mixed solvents and then the solution was stirred (under 20 ^{o}C). Then CF_3SO_3H (1.5 ml, 17.5 mmol, 75 equiv) were added quickly. After the reagent **MC4** was completely consumed (in 1 minute) as monitored by TLC, the excessive solution of potassium hydroxide was added very slowly at the temperature of 0 ^{o}C and then extracted with DCM. The mixed organic phase was dried over anhydrous Na_2SO_4 and then the organic phase was filtered and concentrated under low pressure. The crude product was purified by column chromatography [silica gel, trimethylamine (2 drops per 100 ml eluent) should be added in whole isolation] to give ***meso*-DHG4** (Eluent: petroleum ether: DCM: ethyl acetate = 5:5:1.), ***rac*-DHG4** (Eluent: petroleum ether: DCM: ethyl acetate = 2:2:1) and the byproducts **MG4** (oligomer cycles, a series of

homolog, Eluent: DCM: methyl alcohol = 10:1). Even so, the product ***rac*-DHG4** is impure and they are mixed with **MG4**. As a result, we further isolated them via HPLC.

***meso*-DHG4**

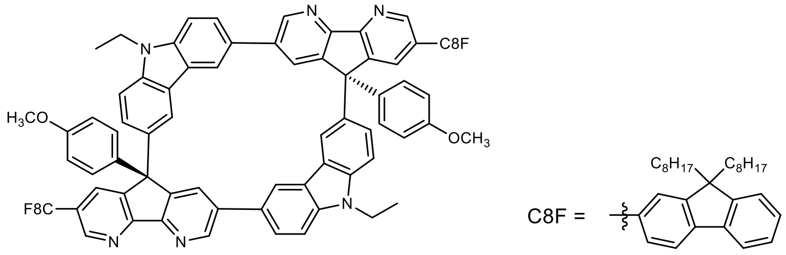

meso-DHG4

Yellow-white powder, 139 mg, 0.082 mmol. Yield: 71%. ^{1}H NMR (600 MHz, $CDCl_3$): δ (ppm) 9.13 – 9.12 (d, *J* = 1.8 Hz, 2H), 9.01 (d, *J* = 1.9 Hz, 2H), 8.65 (d, *J* = 1.6 Hz, 2H), 8.33 (m, 4H), 8.24 (d, *J* = 1.9 Hz, 2H), 7.85 – 7.83 (d, *J* = 8.4 Hz, 2H), 7.79 – 7.76 (m, 6H), 7.68 – 7.67 (m, 4H), 7.55 – 7.54 (d, *J* = 8.4 Hz, 2H), 7.46 – 7.44 (d, *J* = 8.8 Hz, 2H), 7.41 – 7.36 (m, 6H), 6.94 – 6.92 (d, *J* = 9.1 Hz, 4H), 6.70 – 6.68 (d, *J* = 9.1 Hz, 4H), 4.45 – 4.42 (q, *J* = 7.2 Hz, 4H), 3.68 (s, 6H), 2.08 – 2.06 (m, 8H), 1.51 – 1.48 (t, *J* = 7.2 Hz, 6H), 1.22 – 1.10 (m, 44H), 0.82 – 0.80 (m, 16H). ^{13}C NMR (100 MHz, $CDCl_3$): δ (ppm) 158.6, 156.6, 155.8, 151.9, 151.1, 149.7, 149.2, 147.1, 146.5, 141.4, 140.4, 139.7, 138.1, 137.4, 136.8, 136.6, 133.1, 132.5, 132.2, 130.1, 129.1, 127.5, 126.9, 126.6, 125.9, 123.3, 123.1, 123.0, 121.5, 121.0, 120.6, 120.3, 120.0, 113.9, 108.9, 108.5, 60.5, 55.2, 40.4, 37.9, 31.8, 30.0, 29.3, 23.9, 22.6, 14.1, 13.9. FT-IR (cm^{-1}): 3028, 2924, 2855, 1625, 1600, 1578, 1544, 1506, 1485, 1465, 1453, 1440, 1414, 1376, 1348, 1290, 1252, 1236, 1217, 1180, 1167, 1133, 1113, 1037, 1021, 1004, 948, 884, 874, 841, 831, 811, 799, 743, 727, 635, 620. MALDI-ToF-MS: m/z calcd for [M] $C_{122}H_{126}N_6O_2$: 1706.994; found: 1706.937.

***rac*-DHG4**

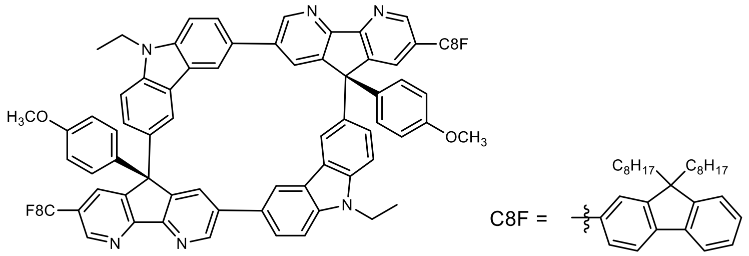

rac-DHG4

Yellow powder, 16 mg, 0.01 mmol. Yield: 8%. ^{1}H NMR (600 MHz, $CDCl_3$): δ (ppm). 9.17 (d, *J* = 1.7 Hz, 2H), 9.07 (d, *J* = 1.8 Hz, 2H), 8.68 (d, *J* = 1.6 Hz, 2H), 8.52 (s, 2H), 8.46 (d, *J* = 1.5 Hz, 2H), 8.17 – 8.16 (d, *J* = 1.9 Hz, 2H), 7.87 – 7.85 (dd, *J* = 8.4, 1.1 Hz, 2H), 7.82 – 7.80 (d, *J* = 8.4 Hz, 2H), 7.76 – 7.75 (d, *J* = 7.5 Hz, 2H), 7.64 – 7.63 (m, 4H), 7.59 – 7.57 (dd, *J* = 8.4, 1.3 Hz, 2H), 7.45 – 7.43 (m, 2H), 7.39 – 7.32 (m, 8H), 7.16 – 7.14 (d, *J* = 9.0 Hz, 4H), 6.81 – 6.79 (d, *J* = 9.1 Hz, 4H), 4.36 – 4.31 (q, *J* = 7.2 Hz, 4H), 3.74 (s, 6H), 2.04 – 2.02 (m, 8H), 1.43 – 1.41 (m, 6H), 1.15 – 1.10 (m, 60H). ^{13}C NMR (100 MHz, $CDCl_3$): δ (ppm). 158.8, 158.0, 156.3, 155.6, 155.0, 154.0, 151.9, 151.8, 151.1, 149.5, 149.1, 148.0, 147.4, 147.3, 146.5, 141.4, 140.6,

140.4, 139.9, 137.7, 136.7, 136.6, 136.3, 135.8, 132.6, 132.2, 131.8, 131.7, 129.9, 129.2, 129.1, 127.4, 126.9, 126.4, 125.0, 123.6, 123.5, 121.5, 120.2, 119.9, 118.8, 114.2, 114.1, 109.2, 108.4, 60.8, 55.3, 55.2, 40.3, 31.9, 31.7, 30.3, 30.0, 29.4, 29.2, 23.9, 22.7, 22.6, 14.1, 14.0, 13.8. FT-IR (cm^{-1}): 2960, 2923, 2873, 2853, 1732, 1667, 1604, 1506, 1486, 1463, 1443, 1378, 1294, 1262, 1180, 1097, 1083, 1024, 971, 802, 741, 668. MALDI-ToF-MS: m/z calcd for [M] $C_{122}H_{126}N_6O_2$: 1706.994; found: 1706.973.

MG4 (a series of macrogrid homologs)

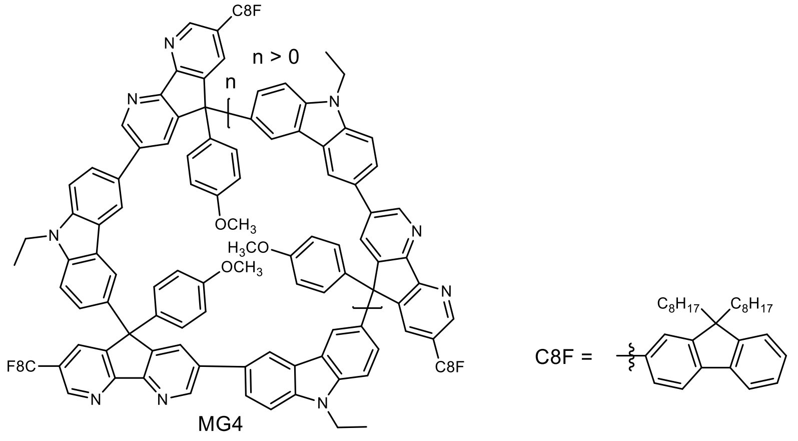

Orange powder, 36 mg. Yield: 18%. ^1H NMR (600 MHz, $CDCl_3$): δ (ppm) 9.16 – 8.98 (4.00 H), 8.30 – 8.00 (7.32 H), 7.80 – 7.67 (m, 6.12 H), 7.60 – 7.53 (5.63 H), 7.45 – 7.30 (14.81 H), 6.99 – 6.75 (4.38 H), 4.40 – 4.20 (4.08 H), 3.85 – 3.65 (6.76 H), 2.10 – 1.96 (13.46 H). 1.44 – 1.27 (20.26H), 1.20 – 1.00 (45.79H). The above integration was relative ratio. ^{13}C NMR (100 MHz, $CDCl_3$): δ (ppm) 158.9, 156.1, 151.8, 151.0, 149.1, 147.3, 141.3, 140.3, 139.6, 136.7, 131.7, 129.3, 127.4, 126.9, 126.8, 126.4, 123.0, 121.4, 120.2, 119.9, 119.3, 114.1, 109.0, 70.6, 61.2, 55.3, 40.3, 37.8, 32.0, 31.7, 30.0, 29.7, 29.4, 29.2, 23.8, 22.7, 22.6, 14.2, 14.1, 13.9, 13.8. FT-IR (cm^{-1}): 2924, 2850, 1603, 1581, 1509, 1478, 1465, 1456, 1441, 1417, 1383, 1375, 1348, 1292, 1250, 1214, 1175, 1156, 1130, 1101, 1088, 1034, 880, 804, 739.

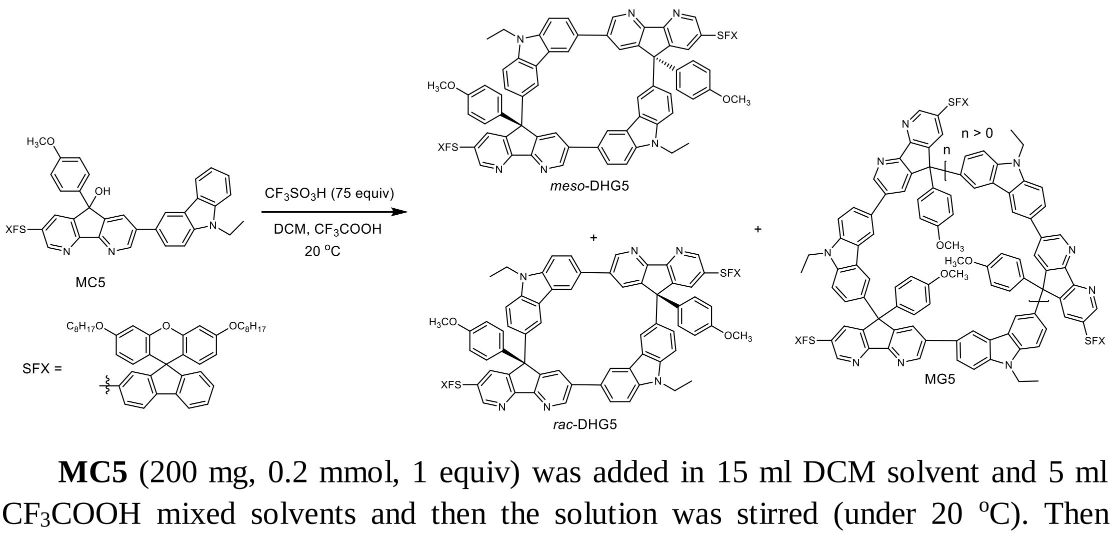

MC5 (200 mg, 0.2 mmol, 1 equiv) was added in 15 ml DCM solvent and 5 ml CF_3COOH mixed solvents and then the solution was stirred (under 20 ^oC). Then

CF_3SO_3H (1.3 ml, 15 mmol, 75 equiv) were added quickly. After the reagent **MC5** was completely consumed (1 minute) as monitored by TLC, the excessive solution of potassium hydroxide was added very slowly at the temperature of 0 °C and then extracted with DCM. The mixed organic phase was dried over anhydrous Na_2SO_4 and then the organic phase was filtered and concentrated under low pressure. The crude product was purified by column chromatography [silica gel, trimethylamine (2 drops per 100 ml eluent) should be added in whole isolation] to give ***meso*-DHG5** (Eluent: petroleum ether: DCM: ethyl acetate = 5:5:1.), ***rac*-DHG5** (Eluent: petroleum ether: DCM: ethyl acetate = 2:2:1, **but it was evidently mixed with other macrocyclic byproduct, thus we did not obtained its NMR spectra**. The yield is roughly evaluated to be 4%) and the byproducts **MG5** (oligomer cycles, a series of homolog, Eluent: DCM: methyl alcohol = 10:1). It is noted that no linear irregular oligomers were observed.

***meso*-DHG5**

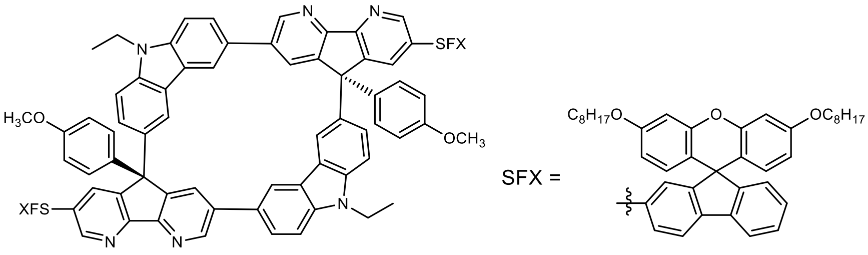

meso-DHG5

Yellow powder, 78 mg, 0.037 mmol. Yield: 37%. ^{1}H NMR (400 MHz, $CDCl_3$): δ (ppm) 8.94 – 8.93 (d, J = 1.8 Hz, 2H), 8.90 (d, J = 1.9 Hz, 2H), 8.57 (d, J = 1.2 Hz, 2H), 8.27 (d, J = 1.2 Hz, 2H), 8.25 (d, J = 1.7 Hz, 2H), 8.05 (d, J = 1.9 Hz, 2H), 7.91 – 7.89 (d, J = 8.0 Hz, 2H), 7.83 – 7.81 (d, J = 7.7 Hz, 2H), 7.74 – 7.71 (dd, J = 8.4, 1.3 Hz, 2H), 7.67 – 7.62 (m, 4H), 7.52 – 7.49 (d, J = 8.5 Hz, 2H), 7.46 (s, 2H), 7.40 – 7.36 (m, 4H), 7.25 – 7.22 (t, J = 7.2 Hz, 2H), 7.17 – 7.15 (d, J = 7.2 Hz, 2H), 6.99 (s, 4H), 6.83 – 6.80 (d, J = 8.9 Hz, 4H), 6.77 – 6.76 (m, J = 2.7 Hz, 4H), 6.63 – 6.61 (d, J = 9.0 Hz, 4H), 6.42 – 6.33 (m, 8H). 5.02 (s, 2H), 4.43 – 4.38 (q, J = 7.2 Hz, 4H), 3.96 – 3.93 (t, J = 6.5 Hz, 8H), 3.65 (s, 6H), 1.38 – 1.26 (m, 58H). ^{13}C NMR (100 MHz, $CDCl_3$): δ (ppm) 159.1, 158.5, 156.7, 156.4, 155.7, 152.1, 149.7, 149.2, 147.1, 146.4, 144.3, 140.4, 140.1, 139.6, 138.5, 138.3, 138.1, 137.3, 136.0, 135.8, 133.1, 132.5, 131.9, 130.1, 129.1, 128.7, 128.3, 127.8, 127.5, 125.5, 124.5, 120.9, 120.6, 120.5, 120.1, 116.3, 113.9, 111.2, 108.9, 108.5, 101.8, 100.0, 68.2, 60.4, 55.2, 53.7, 37.9, 34.3, 31.8, 31.5, 30.3, 29.7, 29.2, 26.1, 22.7, 14.1, 13.9. FT-IR (cm^{-1}): 3039, 2924, 2855, 1630, 1610, 1568, 1547, 1497, 1467, 1441, 1414, 1374, 1342, 1305, 1292, 1249, 1180, 1116, 1104, 1033, 1025, 1002, 880, 856, 828, 806, 782, 750, 727, 698, 634, 620. MALDI-ToF-MS: m/z calcd for [M] $C_{146}H_{138}N_6O_8$: 2103.058; found: 2102.784.

MG5 (a series of macrogrid homologs)

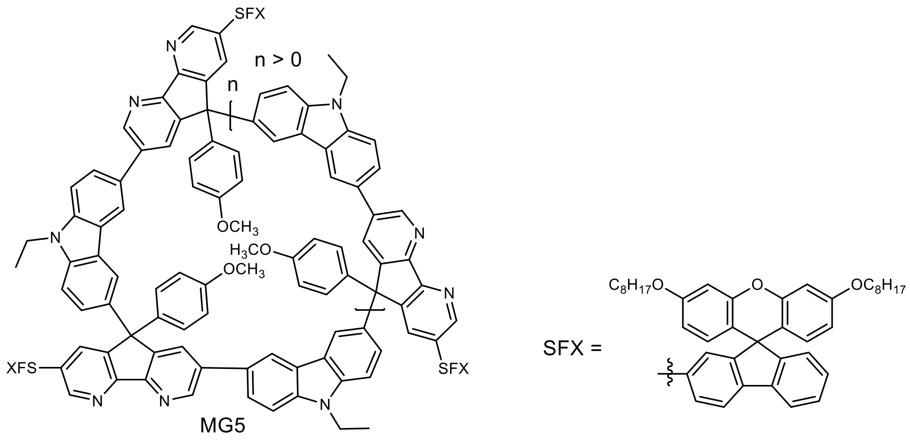

Yellow powder, 100 mg. Yield: 50%. ^{1}H NMR (400 MHz, $CDCl_3$): δ (ppm) 9.00 – 8.88 (4.00H), 8.60 – 8.56 (2.14H), 8.29 – 8.24 (2.28H), 8.06 – 8.04 (2.08H), 7.92 – 7.86 (2.41H), 7.83 – 7.78 (1.98H), 7.71 – 7.68 (1.84H), 7.65 – 7.62 (1.95H), 7.53 – 7.44 (4.04H), 7.41 – 7.31 (3.63H), 7.23 – 7.12 (2.41H), 6.89 – 6.71 (6.51H), 6.61 – 6.58 (1.60H), 6.45 – 6.27 (4.15H), 4.39 – 4.33 (4.01H), 3.93 – 3.90 (4.54H), 3.62 – 3.61 (3.06H), 3.28 – 3.22 (m, 2.56H), 1.76 – 1.72 (4.53H), 1.38 – 1.24 (58.52H). The above integration was relative ratio. ^{13}C NMR (100 MHz, $CDCl_3$): δ (ppm) 171.2, 170.1, 159.1, 159.0, 158.5, 156.6, 156.4, 156.1, 155.6, 152.2, 152.1, 149.6, 149.0, 147.2, 146.4, 140.4, 140.1, 139.6, 138.5, 138.2, 137.2, 136.1, 132.0, 131.9, 131.8, 129.0, 128.7, 128.6, 128.4, 127.8, 127.7, 127.4, 127.3, 125.8, 125.7, 124.4, 123.2, 120.5, 120.1, 119.2, 116.4, 116.2, 114.1, 113.9, 111.1, 110.9, 109.0, 108.9, 108.7, 108.6, 101.8, 68.2, 60.4, 55.1, 34.5, 31.8, 29.7, 29.2, 26.0, 23.3, 22.6, 14.8, 14.1. FT-IR (cm^{-1}): 3035, 2923, 2852, 1659, 1628, 1606, 1569, 1495, 1441, 1414, 1375, 1342, 1291, 1253, 1174, 1102, 1033, 1025, 879, 830, 804, 749, 725, 636.

Preparation of A_2B_2-typed DC monomers

DC-F

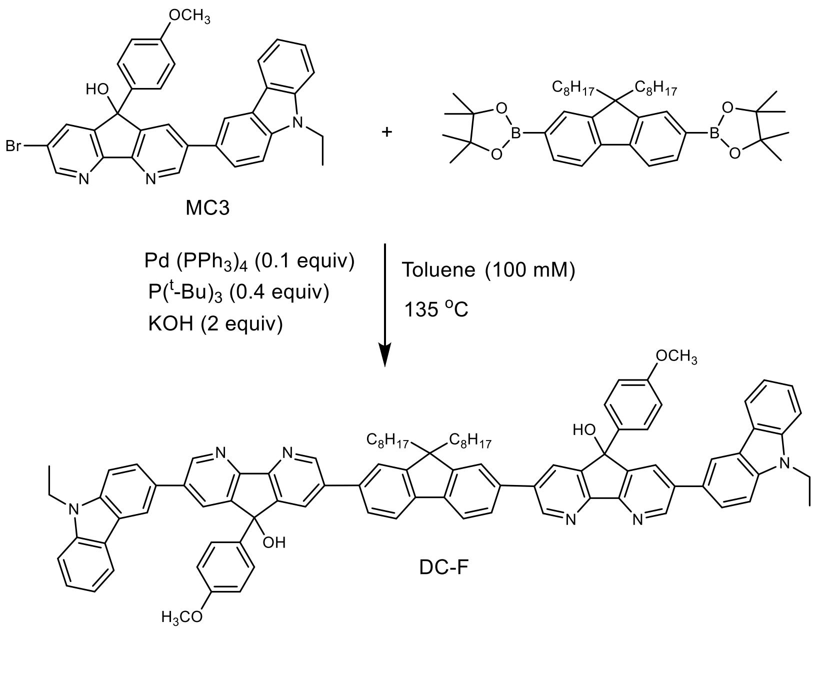

In the nitrogen atmosphere, **MC3** (480 mg, 0.85 mmol, 1 equiv) and 2,2'-(9,9-Dioctyl-9*H*-fluorene-2,7-diyl)bis(4,4,5,5-tetramethyl-1,3,2-dioxaborolane) (247 mg, 0.38 mmol, 0.45 equiv), $Pd(PPh_3)_4$ (50mg, 0.05 mmol, 0.05 equiv), 2 M KOH (0.85 mL, 1.7 mmol, 2 equiv), tri-tert-butylphosphine (0.21 ml, 0.3 mmol, 0.1 equiv, 0.1wt% in toluene) were mixed in a flask containing with 10 ml of toulene. The mixture was reflux for 3 hours (under about 135 ^{o}C), then quenched with water after cooling to room temperature and extracted with DCM. The combined organic solution was dried with anhydrous Na_2SO_4. After the filtration, the crude product was collected by evaporating the solvent. The crude product was purified by column chromatography (silica gel, DCM: ethyl acetate = 4:1, trimethylamine (2 drops per 100 ml eluent) should be added in whole isolation) to give **DC-F** (Yellow powder, 276 mg, 0.204 mmol. Yield: 48%). ^{1}H NMR (400 MHz, $CDCl_3$): δ (ppm) 8.72 – 8.70 (d, *J* = 4.9 Hz, 2H), 8.54 (s, 1H), 8.32 (s, 1H), 8.03 – 7.92 (m, 6H), 7.87 – 7.84 (d, *J* = 8.8 Hz, 1H), 7.79 (s, 1H), 7.48 – 7.40 (m, 10H), 7.36 – 7.30 (m, 4H), 7.21 – 7.15 (m, 4H), 7.06 (broad peak, 2H), 6.86 – 6.84 (d, *J* = 8.8 Hz, 2H), 6.80 – 6.78 (d, *J* = 8.8 Hz, 2H), 3.97 (broad peak, 2H), 3.75 – 3.64 (m, 10H), 1.45 – 1.35 (m, 6H), 1.32 – 1.20 (m, 12H), 1.18 – 1.02 (m, 16H), 1.00 – 0.92 (m, 8H). ^{1}H NMR (400 MHz, $CDCl_3$, the protonation of **DC-F** via CF_3COOH): δ (ppm) 9.37 – 9.35 (m, 4H), 8.93 (s, 4H), 8.56 (s, 2H), 8.22 – 8.20 (d, *J* = 7.6 Hz, 2H), 8.07 – 8.05 (d, *J* = 7.7 Hz, 2H), 7.87 – 7.83 (m, 6H), 7.62 – 7.53 (m, 12H), 7.39 – 7.35 (t, *J* = 6.9 Hz, 2H), 7.07 – 7.05 (d, *J* = 8.8 Hz, 4H), 4.49 – 4.44 (q, *J* = 7.2 Hz, 4H), 3.91 (s, 6H), 2.23 (m, 4H), 1.53 – 1.50 (t, *J* = 7.1 Hz, 6H), 1.15 – 1.10 (m, 24H), 0.80 – 0.77 (m, 6H). ^{13}C NMR (100 MHz, $CDCl_3$, the protonation of **DC-F** via CF_3COOH): δ (ppm) 160.2, 154.0, 151.4, 151.0, 145.9, 144.0, 143.3, 142.1, 141.1, 140.9, 140.8, 139.8, 138.6, 138.2, 132.1, 127.7, 127.3, 127.1, 126.6, 124.8, 124.6, 122.5, 122.4, 122.0, 121.7, 120.7, 120.5, 120.0, 115.4, 110.3, 109.4, 81.0, 56.5, 55.6, 39.9, 37.9, 31.5, 29.6, 29.1, 29.0, 23.8, 22.3, 13.4, 13.3. HRMS (ion: MALDI, FT-ICR-MS): m/z calcd for $[M+H^+]$ $C_{93}H_{89}N_6O_4$: 1353.6940; found: 1353.6950.

DC-SFX

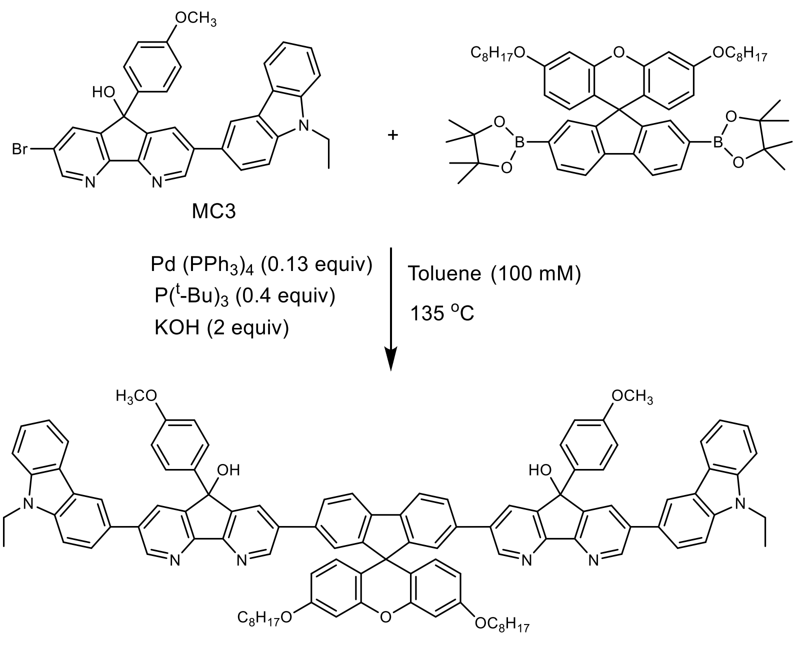

In the nitrogen atmosphere, **MC3** (300 mg, 0.54 mmol, 1 equiv) and 2,2'-(3',6'-Bis(octyloxy)spiro[fluorene-9,9'-xanthene]-2,7-diyl)bis(4,4,5,5-tetramethyl-1,3,2-dioxaborolane) (200 mg, 0.24 mmol, 0.45 equiv), $Pd(PPh_3)_4$ (31 mg, 0.03 mmol, 0.05 equiv), 2 M KOH (0.54 mL, 1.08 mmol, 2 equiv), tri-tert-butylphosphine (0.14 ml, 0.05 mmol, 0.1 equiv, 0.1wt% in toluene) were mixed in a flask containing with 5 ml of toulene. The mixture was reflux for 3 hours (under about 135 ^{o}C), then quenched with water after cooling to room temperature and extracted with DCM. The combined organic solution was dried with anhydrous Na_2SO_4. After the filtration, the crude product was collected by evaporating the solvent. The crude product was purified by column chromatography (silica gel, DCM: ethyl acetate = 4:1, trimethylamine (2 drops per 100 ml eluent) should be added in whole isolation) to give **DC-SFX** (Yellow powder, 230 mg, 0.15 mmol. Yield: 56%). As almost all of the exhibited signals are broad peaks, we only reported its integration. ^{1}H NMR (400 MHz, $CDCl_3$): δ (ppm) 8,65 – 8.61 (4H), 8.31 – 8.23 (2H), 8.01 – 7.90 (8H), 7.81 – 7.76 (2H), 7.58 – 7.43 (6H), 7.41 – 7.33 (6H), 7.21 – 7.18 (4H), 6.91 – 6.73 (8H), 6.30 – 6.04 (4H), 4.00 – 3.84 (8H), 3.74 – 3.72 (6H), 2.80 – 2.70 (2H), 1.82 – 1.72 (4H), 1.45 – 1.18 (30H), 0.91 (8H). ^{13}C NMR (100 MHz, $CDCl_3$): δ (ppm) 162.5, 159.2, 159.1, 157.1, 155.6, 155.4, 154.1, 153.9, 151.9, 149.4, 149.1, 146.8, 146.3, 140.1, 139.4, 137.8, 137.5, 136.5, 133.4, 133.3, 131.9, 130.8, 130.7, 130.5, 128.8, 128.4, 128.2, 127.6, 127.2, 126.8, 125.8, 124.5, 124.4, 123.3, 122.7, 120.5, 119.0, 118.6, 118.5, 115.5, 115.2, 114.9, 113.9, 111.7, 111.2, 110.2, 109.8, 108.4, 102.0, 101.9, 79.1, 68.0, 55.2, 37.1, 31.9, 29.4, 29.3, 26.1, 22.7, 14.1, 13.6. MALDI-ToF-MS: m/z calcd for [M] $C_{105}H_{94}N_6O_7$: 1550.705; found: 1550.878.

Nano-linkage by polygridization

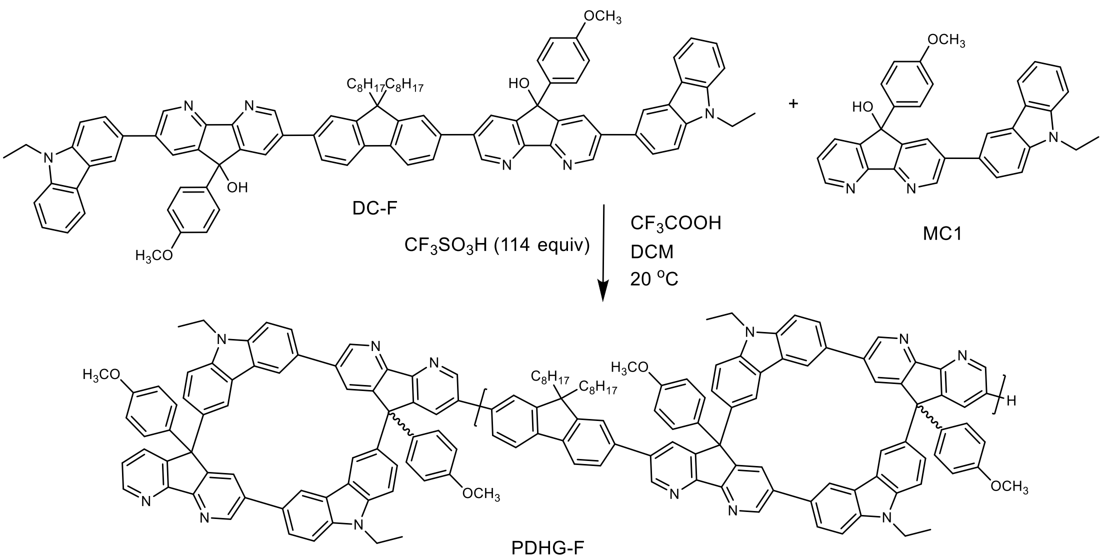

PDHG-F

DC-F (52 mg, 0.04 mmol, 1 equiv) was added in 1.5 ml DCM and 0.5 ml TFA solvent, then the solution was stirred (under 20 °C). Then CF_3SO_3H (0.40 ml, 4.55 mmol, 114 equiv) were added quickly. **6~25** seconds later, we added **MC1** (5~10 mg, 0.01~0.021 mmol, 0.26~0.52 equiv) quickly to terminate the intermediates. Then in 1 min, the excessive solution of potassium hydroxide was added slowly at the temperature of 0 °C and then extracted with DCM. **It is noted that the solvent should be added in less amount (high concentration of DC-F) to ensure the instantly complete contact between polymeric intermediates and MC1, which determining the success of this polygridization.** **In addition, adding the highly excessive amount of MC1 (2 equiv of DC-F monomers) leads to introducing the byproduct MG1 derived from MC1 end-capping reagent.** The mixed organic phase was dried over anhydrous Na_2SO_4 before organic phase was filtered and concentrated under low pressure. After filtration the crude product was collected by evaporating the solvent. The crude product was purified by Soxhlet extraction using tetrahydrofuran and acetone mixing solvents.

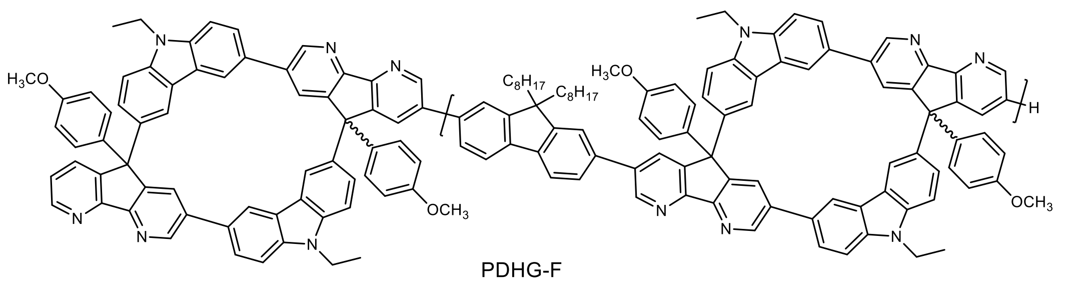

PDHG-F

PDHG-F. **(**The orange bulk solid, 60% yields after the Soxhlet extraction). ^1H NMR (600 MHz, $CDCl_3$): δ (ppm) 9.17 – 8.98 (4.00H), 8.80 (0.23H), 8.71 (0.16H), 8.65 – 8.61 (1.40H), 8.55 (0.16H), 8.46 (0.16H), 8.32 (2.87H), 8.24 – 8.21 (1.41H), 8.15 – 8.11 (1.08H), 8.03 – 7.99 (1.04H), 7.80~7.86 (0.88H), 7.80 – 7.50 (9.89H), 7.45 – 7.35 (5.12H), 7.16 (0.31H), 6.92 – 6.60 (7.66H), 4.45 – 4.30 (3.77H), 3.85 – 3.60 (6.83H), 2.14 – 2.05 (3.83H), 1.58 – 1.43 (18.17H), 1.30 – 1.20 (25.04H), 1.18 – 1.10 (22.09H), 0.89 – 0.76 (31.65H). ^{13}C NMR (100 MHz, $CDCl_3$): δ (ppm) 158.6, 157.9,

156.7, 156.0, 155.8, 152.3, 150.1, 149.6, 149.2, 146.5, 146.4, 140.7, 140.4, 140.1, 139.7, 139.6, 138.3, 137.4, 137.3, 136.4, 134.5, 133.2, 132.5, 132.0, 131.9, 130.1, 129.1, 129.0, 126.7, 126.5, 126.0, 123.3, 123.1, 121.7, 121.0, 120.6, 113.9, 113.8, 109.1, 108.9, 108.5, 100.0, 99.6, 60.4, 55.7, 55.2, 40.3, 37.9, 37.7, 31.7, 30.0, 29.3, 24.0, 22.7, 22.6, 14.1, 13.9. FT-IR (cm^{-1}): 2952, 2923, 2853, 1602, 1578, 1509, 1479, 1438, 1374, 1350, 1294, 1249, 1215, 1179, 1157, 1129, 1032, 880, 815, 804, 749, 702, 694, 634, 621. It is noted that the integration in the spectrum only represented the integration ratio rather than the fact number of hydrogen atoms.

CP-F

If without CF3COOH-additive, 100% insoluble **CP-F** (red solid) were obtained. FT-IR (cm^{-1}): 3044, 2930, 2850, 1630, 1604, 1581, 1510, 1483, 1441, 1378, 1346, 1290, 1251, 1178, 1159, 1132, 1088, 1027, 952, 880, 818, 805, 749, 697, 635.

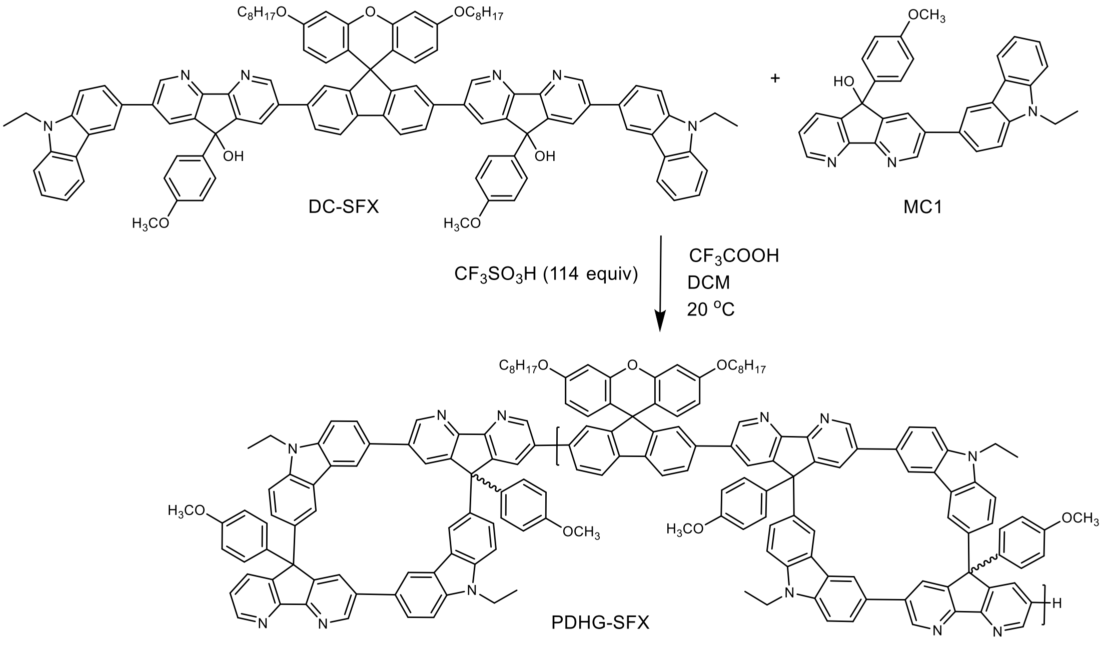

DC-SFX (45 mg, 0.03 mmol, 1 equiv) was added in 1 ml DCM and 0.30 ml TFA solvent, then the solution was stirred (under 20 °C). Then CF_3SO_3H (0.20 ml, 3.41 mmol, 114 equiv) were added quickly. 10 seconds later, we added **MC1** (4~8 mg, 0.008~0.016 mmol, 0.26~0.52 equiv) quickly to terminate the reaction. Then after 1 min, the excessive solution of potassium hydroxide was added slowly at the temperature of 0 °C and then extracted with DCM. The mixed organic phase was dried over anhydrous Na_2SO_4 and then the organic phase was filtered and concentrated under low pressure. After the filtration, the crude product was collected by evaporating the solvent. The crude product was purified by Soxhlet extraction using tetrahydrofuran and acetone mixing solvents.

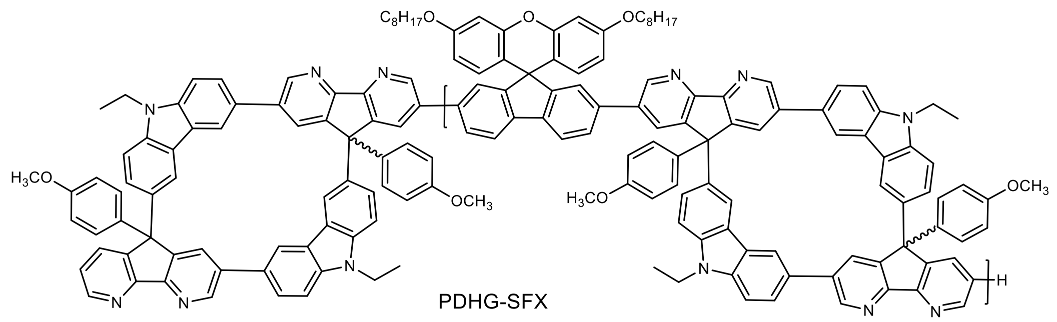

PDHG-SFX. Orange compact film, 22 mg. Yield: 50% (after the Soxhlet extraction). ^{1}H NMR (400 MHz, $CDCl_3$): δ (ppm) 9.15 – 8.88 (4.00H), 8.80 – 8.79 (0.38H), 8.65 – 8.56 (1.99H), 8.29 – 8.26 (2.21H), 8.12 – 7.85 (4.70H), 7.77 – 7.64 (3.95H), 7.50 – 7.46 (1.83H), 7.41 – 7.35 (2.95H), 6.85 – 6.70 (4.56H), 6.63 – 6.55 (1.80H), 6.44 – 6.32 (2.00H), 4.45 – 4.27 (3.81H), 3.97 – 3.91 (2.21H), 3.74 – 3.60 (10.13H), 2.30 – 2.10 (2.78H), 1.57 – 1.25 (31.78H). ^{13}C NMR (100 MHz, $CDCl_3$): δ (ppm) 159.2, 158.9, 158.5, 157.8, 157.3, 157.0, 156.8, 156.0, 155.6, 152.2, 152.1, 150.1, 149.6, 149.1, 147.2, 147.0, 146.8, 146.4, 140.4, 139.6, 138.2, 137.4, 135.9, 134.5, 133.2, 131.9, 130.0, 129.2, 129.1, 129.0, 128.7, 127.3, 126.6, 125.9, 124.4, 123.3, 123.0, 122.7, 120.9, 120.8, 120.6, 119.2, 115.9, 114.1, 113.9, 113.8, 111.4, 111.3, 109.2, 109.1, 108.9, 108.8, 108.6, 108.4, 107.2, 101.9, 68.2, 61.1, 60.4, 55.2, 37.9, 37.7, 31.8, 30.9, 29.4, 29.2, 26.1, 22.7, 14.1, 13.9. FT-IR (cm^{-1}): 3038, 2924, 2850, 1628, 1605, 1508, 1482, 1440, 1410, 1376, 1346, 1293, 1250, 1218, 1176, 1130, 1108, 1101, 1032, 1004, 875, 802, 749, 726, 636, 620. It is noted that the integration in the spectrum only represented the integration ratio rather than the fact number of hydrogen atoms.

CP-SFX. Red insoluble solid. 11 mg. Yield: `~25% yields. FT-IR (cm^{-1}): 2935, 2846, 1679, 1630, 1609, 1509, 1483, 1441, 1288, 1257, 1178, 1035, 831, 806, 768, 749, 653, 643.

Theoretical calculations and simulations

Method for the vibrational simulation of *meso*-DHG1 and *rac*-DHG1

The simulation method of vibration modes of **DHG1** backbones was detailed. For both ***meso*-DHG1** and ***rac*-DHG1**, the geometric optimization was performed under the calculation method of RB3LYP and the basis set of 6-31G (D). On this basis, the vibrational simulation was performed under the method of RB3LYP and the basic set of 6-31G(D). The scaling factor 0.9614 is referred to the literature[4]. The output files for ***meso*-DHG1** and ***rac*-DHG1** are provided as the **Supplementary Data 1** and **2** respectively.

The calculation of cohesive energy density of PDHG-F chains.

The amorphous structure consisting of 5 chains of **PDHG-F** (each chain exhibits the *DP* of 9, approximate to the experimental results) was constructed via the "Amorphous cell" modules in the Material Studio 2016 software. The initial density is set as 0.2 g cm^{-3}. Then, the geometry optimization (in Forcite plus modules) was carried out based on the SMART algorithum (that is the cascade algorithum of the Steepest

Descent, ABNR and Quasi-Newton methods, in sequence), an energy convergence tolerance of 0.0001 kcal mol^{-1}, a force convergence tolerance of 0.005 kcal mol^{-1} $Å^{-1}$, the maxium number of iterations of 200000. In addition, we used COMPASS force field[5] in which the summation method of Electrostatic terms was set as the Ewald (a buffer width of 0.5 Å) and the summation method of van der Waals terms was used as the Group based (a cutoff distance of 15.5 Å). The MD simulation after the geometry optimization was firstly performed via the Forcite plus modules based on the NPT ensemble, 298 K, 10^{-4} GPa, a total time of 1100 ps with a time step of 1.5 fs, COMPASS force field, Group-based summation of van de Waals terms (cutoff: 15.5 Å) and Ewald-summation of electrostatic terms. In order to obtain the statistic conformations in equilibrium state, then we carried out the NVT dynamics based on the 298 K, a total simulation time of 3000 ps with a time step of 1.5 fs. The actual conformational state was obtained every 5000 steps. For each conformations, we calculated the cohesive energy density (*CED*) using the equation $CED = E_{\text{inter}} / V$, where E_{inter} is defined as the total energy of intermolecular interactions (in this case, the intermolecular attractive force should be stronger than intermolecular repulsion) and V is equal to the volume of amorphous cell. The cohesive energy density table of **PDHG-F** chains is provided as the **Supplementary Data 3**.

The calculation of cohesive energy density of $CHCl_3$ solvent.

We constructed the amorphous structures of $CHCl_3$ solvent molecules via the "Amorphous cell" modules (the density was set as the 1.48 g cm^{-3} that is the consistent with the real density, and the number of $CHCl_3$ molecules was set as 30). Then, such system was subjected to the NVT ensemble based on the 298 K, a total time of 600 ps with a time step of 1.5 fs, COMPASS force field, Group-based summation of van de Waals terms (cutoff: 15.5 Å) and Ewald-summation of electrostatic terms. The actual conformation during the 300~600 ps of the dynamic procedure was saved every 4000 steps to calculate the cohesive energy density using the equation $CED = E_{\text{inter}} / V$, where E_{inter} is defined as the total energy of intermolecular interactions (in this case, the intermolecular attractive force should be stronger than intermolecular repulsion) and V is equal to the volume of amorphous cell. The cohesive energy density table of $CHCl_3$ solvent is provided as the **Supplementary Data 4**.

The calculation of cohesive energy density of tetrahydrofuran solvent.

The amorphous structures of tetrahydrofuran solvent molecules was constructed via the "Amorphous cell" modules (the density was set as the 0.89 g cm^{-3} that is the consistent with the real density, and the number of tetrahydrofuran molecules was set as 30). Then, such system was subjected to the NVT ensemble based on the 298 K, a total time of 600 ps with a time step of 1.5 fs, COMPASS force field, Group-based summation of van de Waals terms (cutoff: 15.5 Å) and Ewald-summation of electrostatic terms. The actual conformation during the 300~600 ps of the dynamic procedure was saved every 4000 steps to calculate the cohesive energy density using the equation $CED = E_{\text{inter}} / V$, where E_{inter} is defined as the total energy of intermolecular interactions (in this case, the intermolecular attractive force should be stronger than

intermolecular repulsion) and *V* is equal to the volume of amorphous cell. The cohesive energy density table of tetrahydrofuran solvent is provided as the **Supplementary Data 5**.

The chain collapse simulation of *meso*-PDHG-F (full *meso*-configurational PDHG-F chains) and *rac*-PDHG-F (full *rac*-configurational PDHG-F chains).

The simulation of chain collapse for other chain length such as *DP* = 7, 9 and 18 were detailed. All **PDHG-F** chains were constructed in the expanded conformations where the dihedral angle between adjacent repeat units are approximate to 180°. The dynamics of chain collapse were carried out under the conditions of NVT ensemble (298 K) and the COMPASS forcefield (the summation method of van de Waals term and the electrostatic term are both Group-based in which the cutoff distance were set as 12.5 Å). For *DP* = 7, the total times of dynamics were set as 600 ps for ***meso*-PDHG-F** with the time step of 1.5 ps. For ***rac*-PDHG-F** (*DP* = 7), the total times of dynamics were set as 2000 ps with the time step of 1.5 ps. For *DP* = 9, the total times of dynamics were set as 500 ps for ***meso*-PDHG-F** with the time step of 1.5 ps. For ***rac*-PDHG-F** (*DP* = 9), the total times of dynamics were set as 500 ps with the time step of 1.5 ps. For *DP* = 18, the total times of dynamics were set as 800 ps for ***meso*-PDHG-F** with the time step of 1.5 ps. For ***rac*-PDHG-F** (*DP* = 18), the total times of dynamics were set as 2500 ps with the time step of 1.5 ps. The trajectory files of collapsed process are provided as **Supplementary Data 6** (***meso*-PDHG-F**, *DP* = 7), **Supplementary Data 7** (***rac*-PDHG-F**, *DP* = 7), **Supplementary Data 8** (***meso*-PDHG-F**, *DP* = 9), **Supplementary Data 9** (***rac*-PDHG-F**, *DP* = 9), **Supplementary Data 10** (***meso*-PDHG-F**, *DP* = 18), **Supplementary Data 11** (***rac*-PDHG-F**, *DP* = 18).

Supplementary Note 1. Characteristics of Friedel-Crafts reactions and Gridization

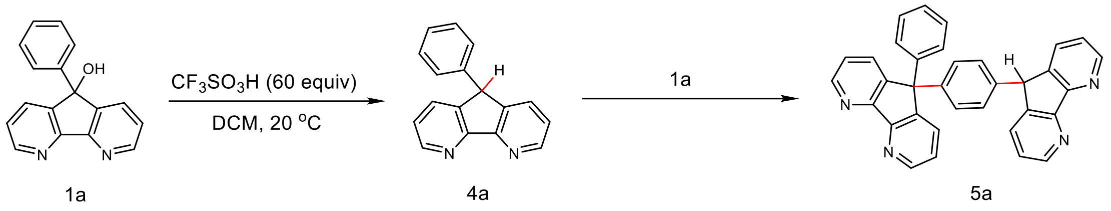

Supplementary Figure 1. Byproducts in Friedel-Crafts reactions. The dehydroxylation was observed in fluorenols' Friedel-Crafts reactions as well in the previous work[6]. The plausible mechanism involves intermolecularly cationic transfer[7]. On this basis, **4a** could react with **1a** to generate **5a**.

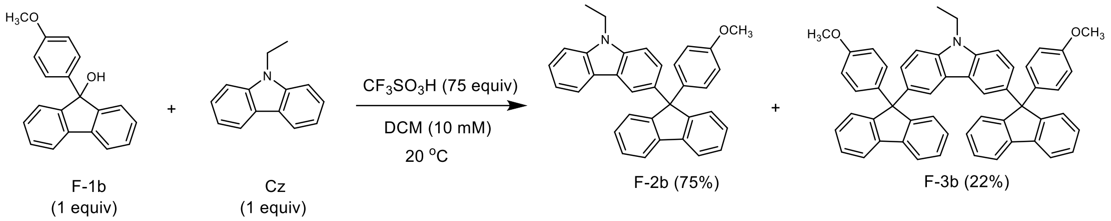

Supplementary Figure 2. Fluorene-based Friedel-Crafts reactions. The conditions are the same as the reactions of **1a** and **1b**, generating **F-2b** in 75% yield and **F-3b** in 22% yield. The evidently lower **F-3b** yield suggests the reaction acceleration of **F-2b** is sharply diminished.

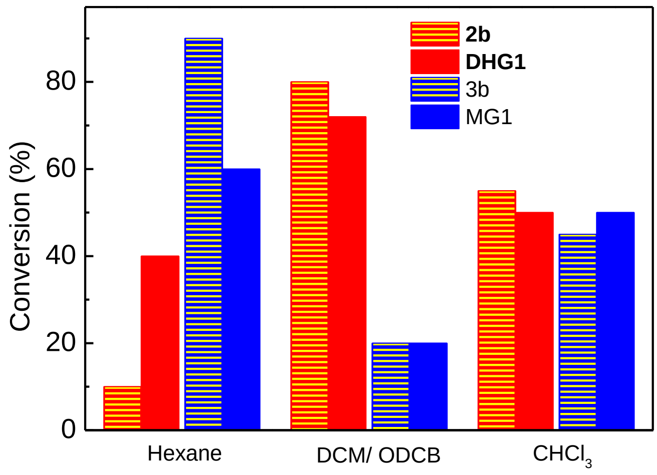

Supplementary Figure 3. Solvent effects in Friedel-Crafts of 1b and gridization of MC1 based on the same 10 mM concentration and CF_3COOH-additive. The serious aggregation in hexane is consistent with the generation of dominant **3b** and **MG1** respectively. For the Friedel-Crafts reactions, the height of red and yellow

column corresponds to the yield of **2b**; the height of blue and yellow column represents the yield of **3b**. For the gridization reactions, the heights of pure red and blue column correspond to the yields of **DHG1** and **MG1** respectively.

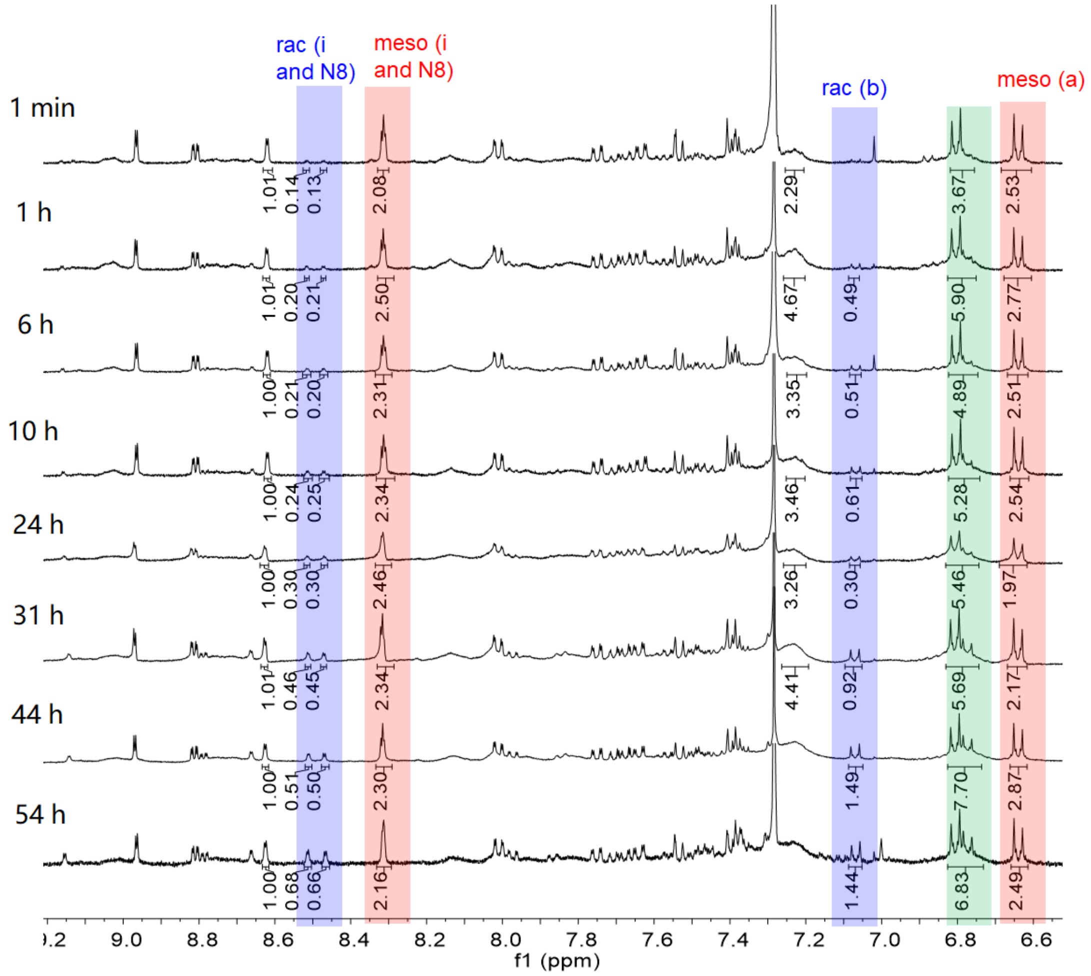

Supplementary Figure 4. The ^{1}H NMR spectra of the MC1 gridization systems after reacting specific times. The blue region indicates the signals that belong to *rac*-configuration while the red region shows the peaks of *meso*-configuration. The green region indicates the signals consisting of ***meso*-DHG1** (b site), ***rac*-DHG1** (a site) and **MG1** (a site).

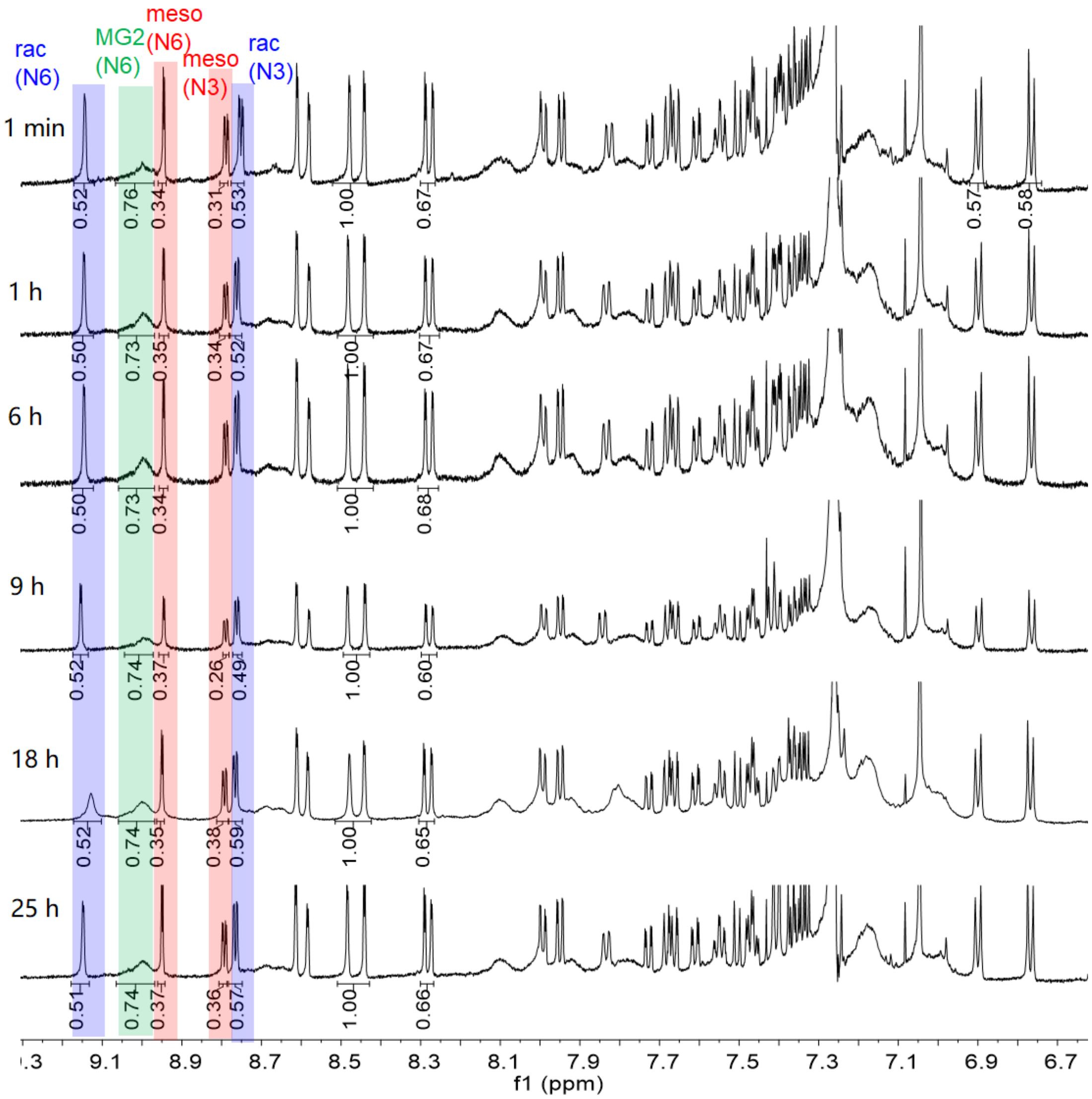

Supplementary Figure 5. The ^{1}H NMR spectra of the MC2 gridization systems after reacting specific times. The blue region indicates the signals that belong to *rac*-configuration while the red region shows the peaks of *meso*-configuration. The green region indicates the **MG2** region.

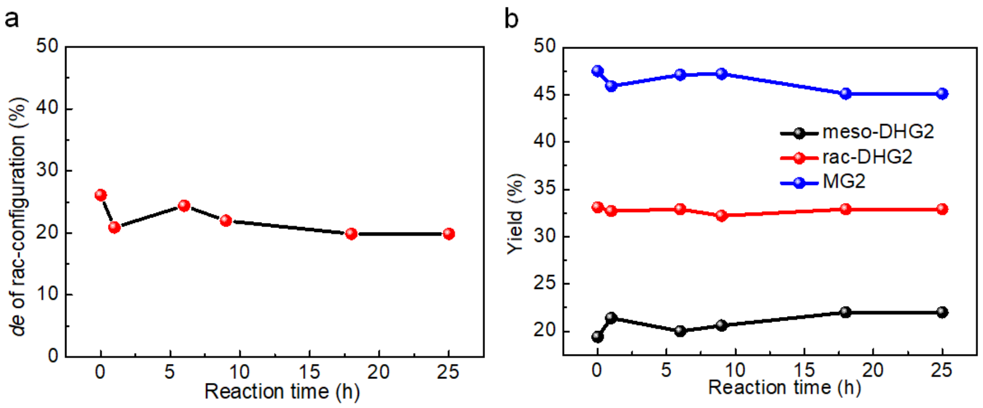

Supplementary Figure 6. The reaction results of the MC2 gridization systems. a The *de* value (red dots) of *rac*-configuration. **b** The yields of corresponding products involving ***meso*-DHG2** (black line and dots), ***rac*-DHG2** (red line and dots) and **MG2** (blue line and dots).

Supplementary Note 2. The characteristics of the polygridization from DC-F monomers

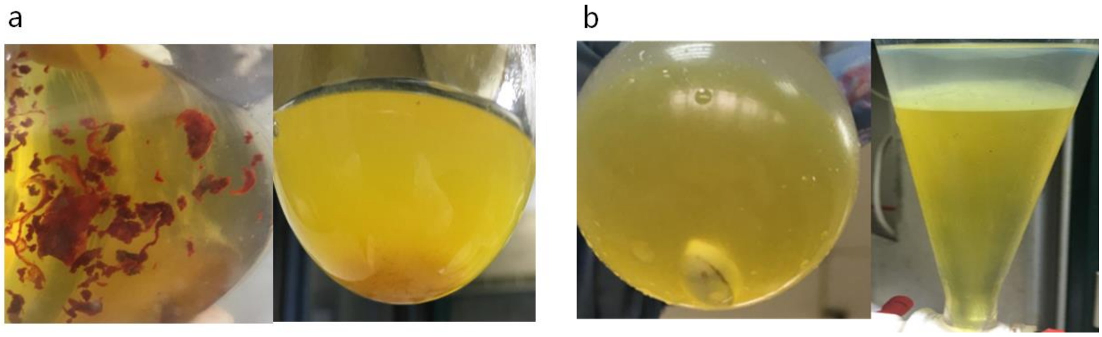

Supplementary Figure 7. The reacting solution after quenching the polygridiation. a) exhibits the cross-linked **CP-F**. **a** If the concentration is large (>10 mM), the **CP-F** is red bulk solid with elastic property (swelling in DCM or $CHCl_3$ solution). However, the lower concentration (1 mM) caused to tiny yellow particles. **b** The reacting solution without **CP-F** (in 15~60 mM concentration).

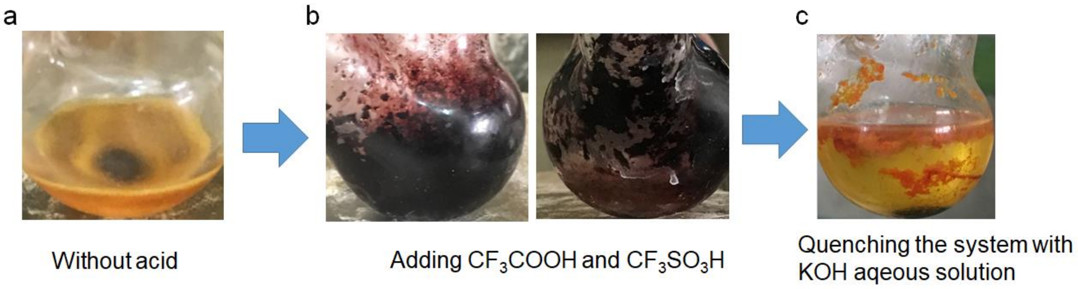

Supplementary Figure 8. The reaction solution state of PDHG-F after adding the CF_3COOH and CF_3SO_3H. **a** In the absence of these acids, the solution is transparent. **b** If adding CF_3COOH and CF_3SO_3H, the solution become black. After 15 min, the insoluble sediments were generated. The yields of sediments were gradually increased if elongating the reaction time. **c** Using KOH aqeous solution to quench such system, the red insoluble cross-linking polymers were observed.

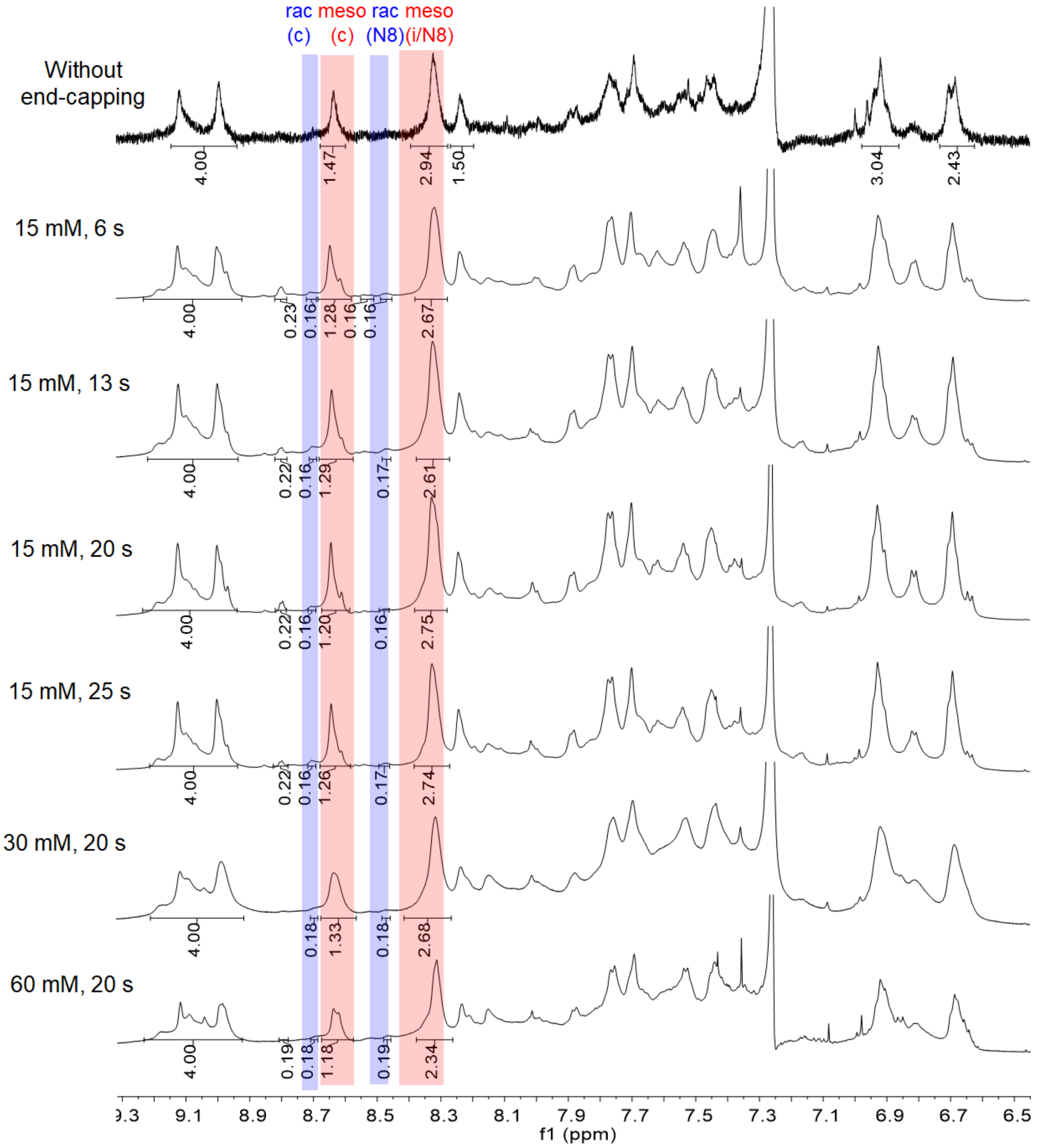

Supplementary Figure 9. The ^{1}H NMR spectra of PDHG-F from various conditions. The blue region indicates the signals that belong to *rac*-configuration while the red region shows the peaks of *meso*-configuration. The **PDHG-F** spectrum without end-capping of **MC1** is provided as well to demonstrate the dominant *meso*-selectivity of polygridization.

Supplementary Note 3. Characteristics on the stereoselectivity of DHGs and PDHG-F structures

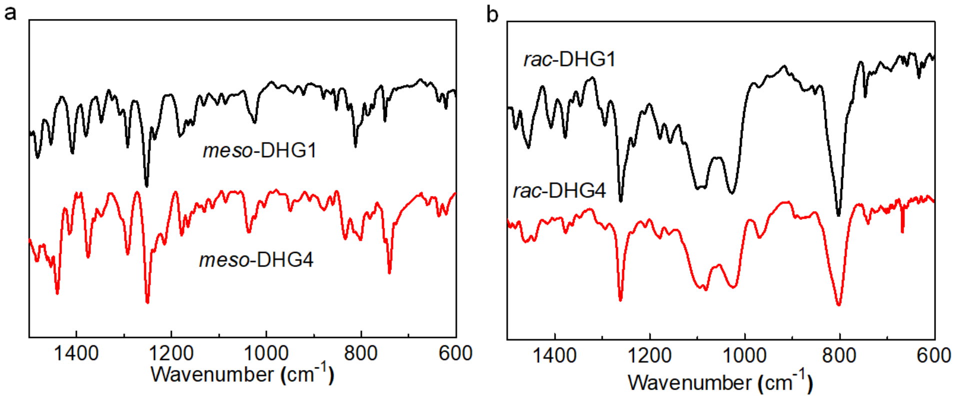

Supplementary Figure 10. The FT-IR spectra for *meso*- and *rac*-configurational DHG products in the range of 1500~600 cm^{-1}. a The FT-IR spectra of ***meso*-DHG1** (black line) and ***meso*-DHG4** (red line). **b** The FT-IR spectra of ***rac*-DHG1** (black line) and ***rac*-DHG4** (red line).

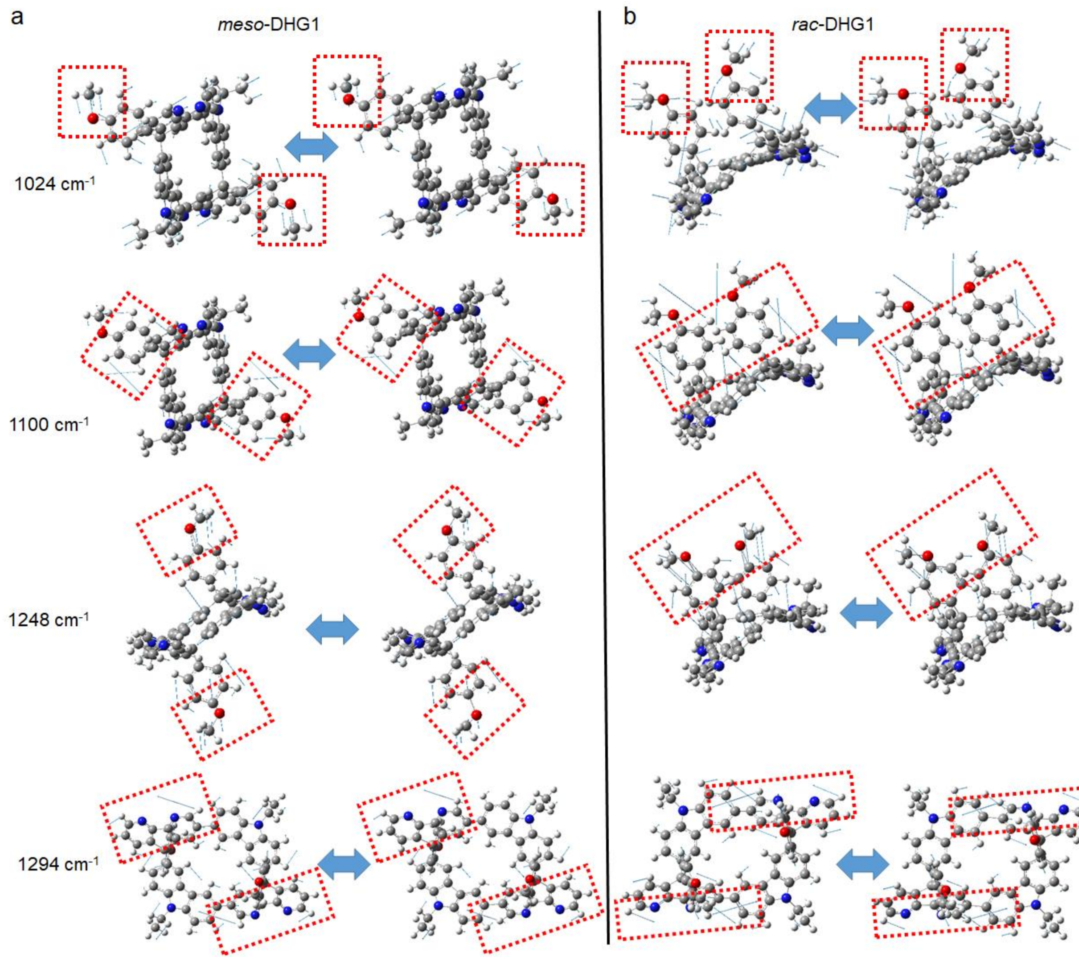

Supplementary Figure 11. The vibrational mode of DHG1 products. The vibrational features (associated with stereoregular backbones) are shown in red dashed boxes. **a** The vibrational simulation of ***meso*-DHG1**. **b** The vibrational simulation of ***rac*-DHG1**.

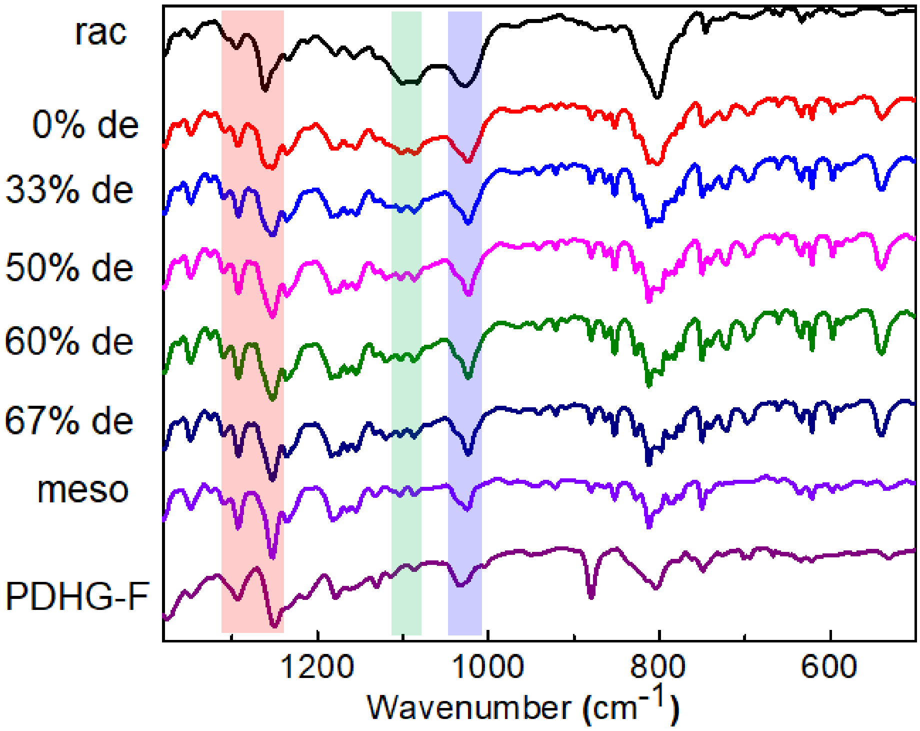

Supplementary Figure 12. The FT-IR spectra of the doping systems where *meso*-DHG1 and *rac*-DHG1 are mixed in specific ratio (0% *de* = 1:1 of ***meso*-DHG1**: ***rac*-DHG1**; 33% *de* = 2:1; 50% *de* = 3:1; 60% *de* = 4:1; 67% *de* = 5:1). The red region ranges from 1294 to 1248 cm^{-1}, which exhibits stronger band intensity in ***meso*-DHG**s. The green region exhibits the band at 1100~1085 cm^{-1}, which is more powerful in ***rac*-DHG**s. The blue region includes the vibrational absorption at 1030~1024 cm^{-1}, which is stronger in ***rac*-DHG**s.

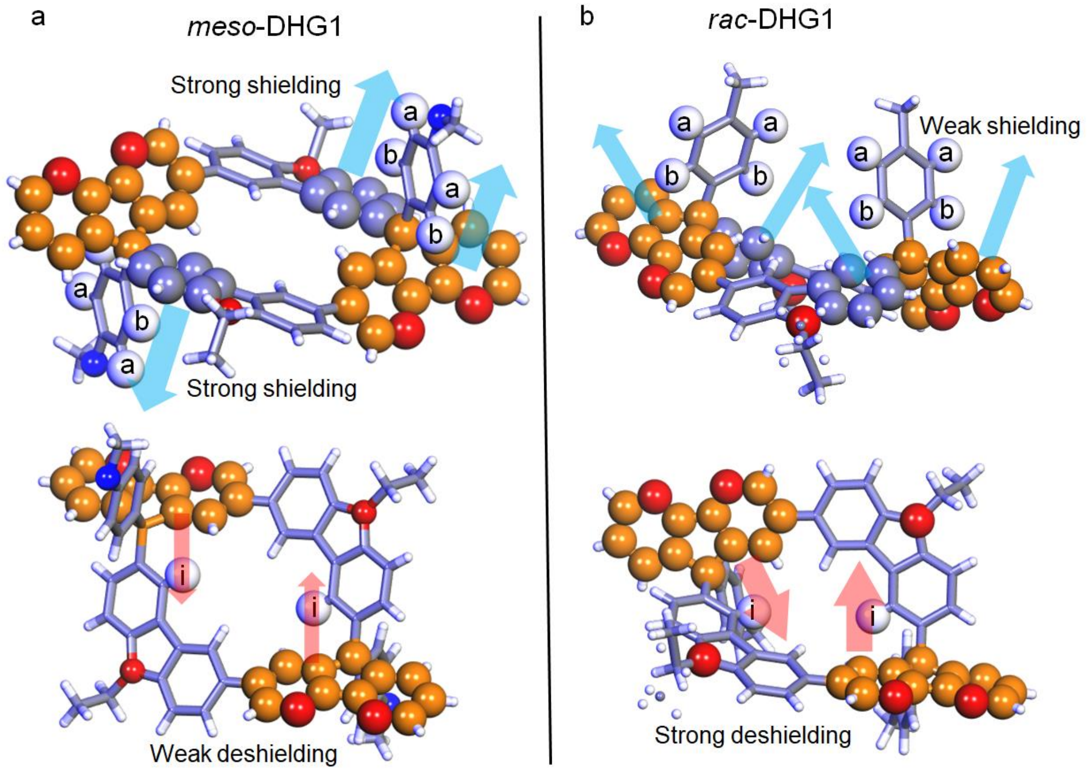

Supplementary Figure 13. The shielding effect (blue arrows) and deshielding effect (red arrows) analysis on DHG1. a These effect on ***meso*-DHG1**. **b** These effect on ***rac*-DHG1**. The orange-bead segments are diazafluorene moieties.

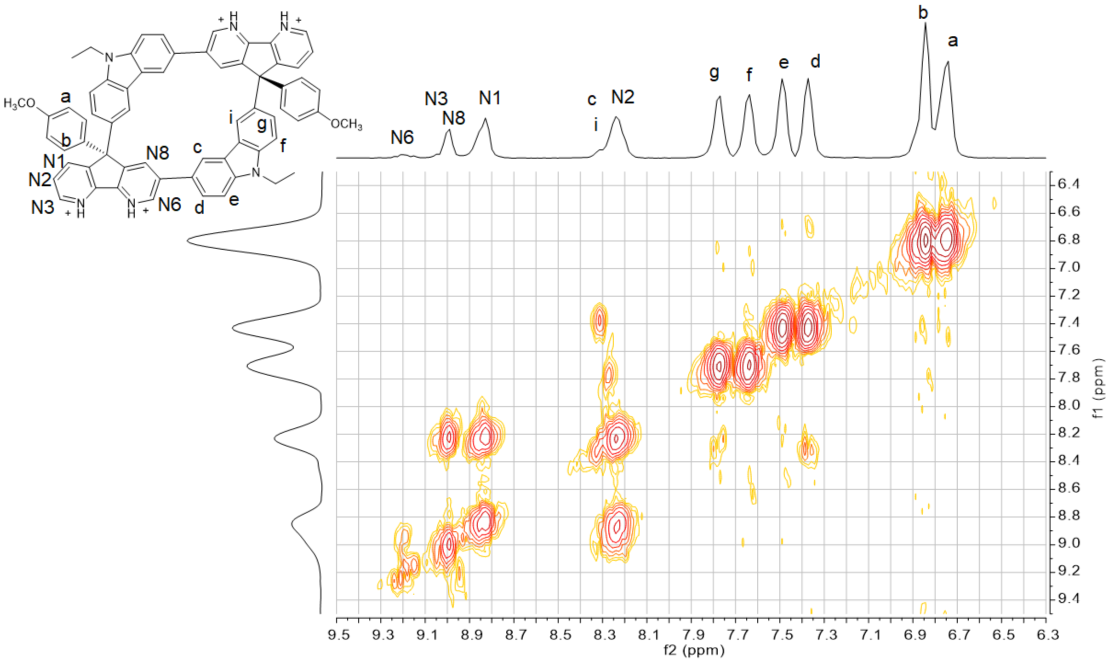

Supplementary Figure 14. The ^{1}H-^{1}H COSY spectra of *meso*-DHG1 (protonated) in $CDCl_3$-CF_3COOH solution.

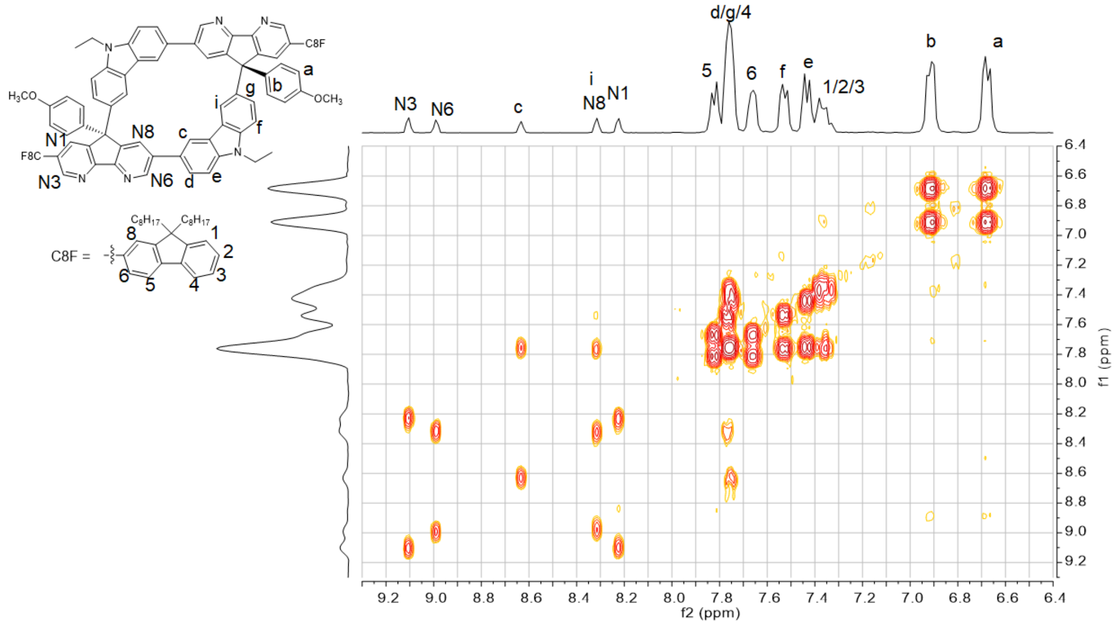

Supplementary Figure 15. The ^{1}H-^{1}H COSY spectra of *meso*-DHG4 was represented in $CDCl_3$ solution.

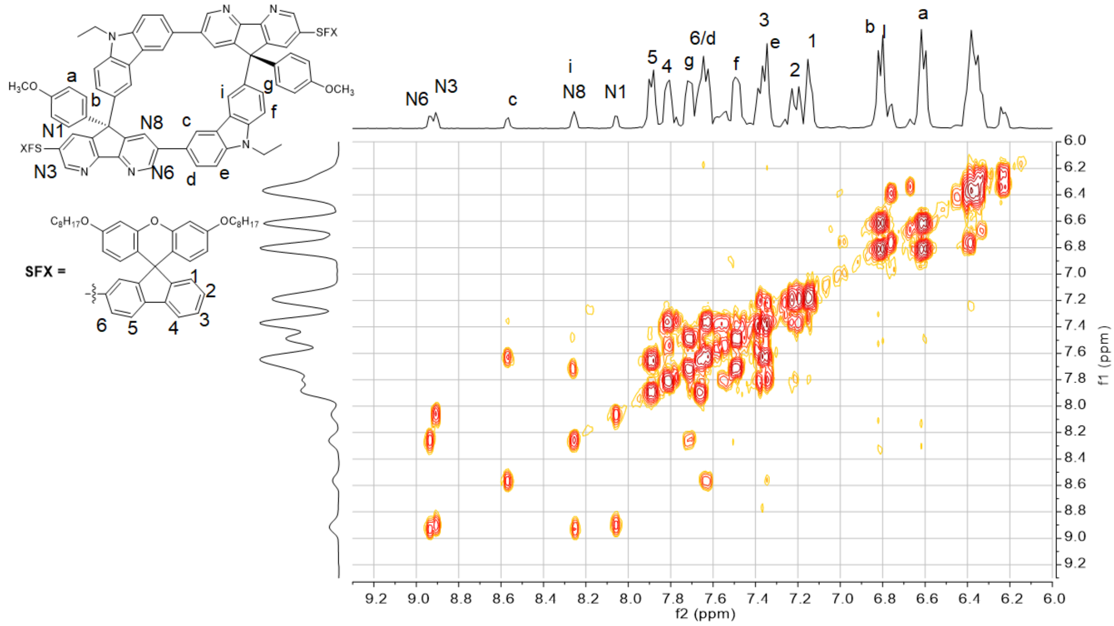

Supplementary Figure 16. The ^{1}H-^{1}H COSY spectra of *meso*-DHG5 was represented in $CDCl_3$ solution.

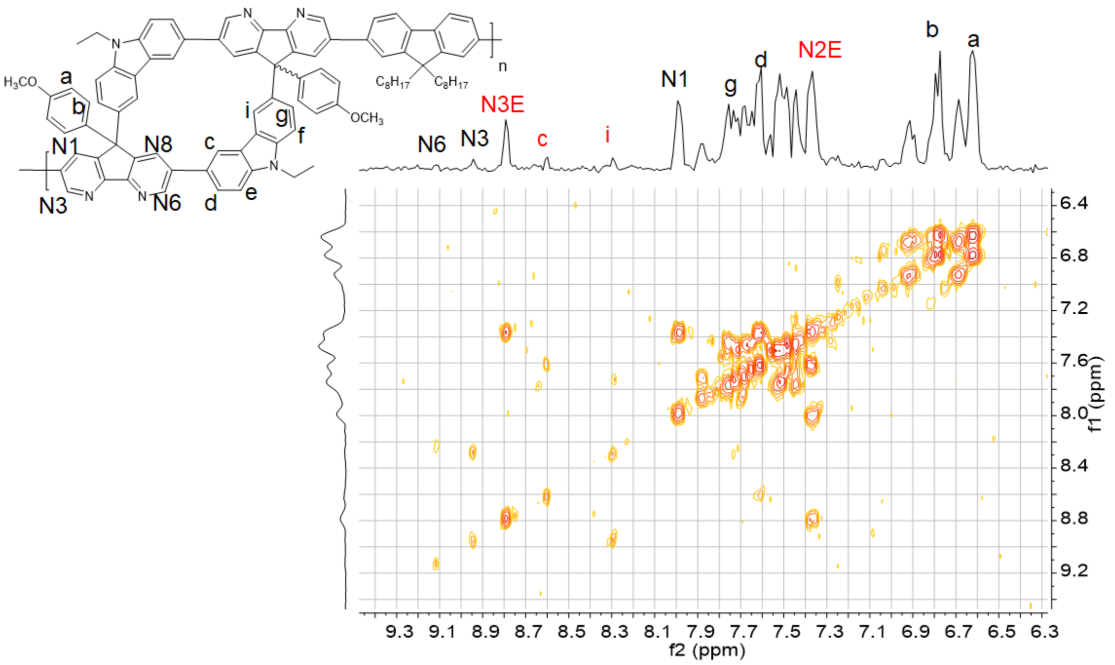

Supplementary Figure 17. The ^{1}H-^{1}H COSY spectra of PDHG-F in lower average molecular weight. The **N3E** and **N2E** signals represent the corresponding positions **N3** and **N2** both at the terminal **DHG**s that does not link C8F groups. The cross-peak (8.60, 7.60) demonstrates the c site at 8.60 ppm (the signal at 7.60 ppm is d site) while cross-peak (8.30, 7.72) indicates the i-signals at 8.30 ppm.

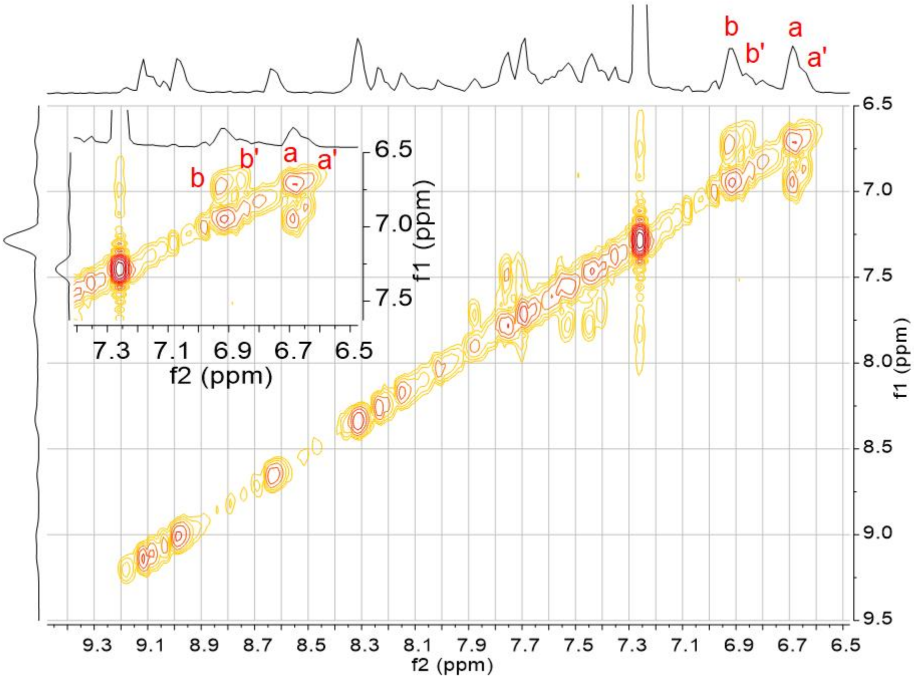

Supplementary Figure 18. The ^{1}H-^{1}H COSY spectra of PDHG-F in high molecular weight. The spin-coupling correlation of a and b (6.70, 6.95) is consistent with the ***meso*-DHG4**; the spin-coupling correlation of a' and b' (6.85, 6.65) is identical to the ***meso*-DHG1**. The *rac* features are not observed.

Supplementary Note 4. The calculation of expansion factor β of PDHG-F in solution

To testify the validity of stereoselective demonstration relying on c/i/N8 signals, we studied the chain conformation of **PDHG-F** via the molecular dynamic simulation. For the relatively good solvent $CHCl_3$, the solvent molecules enable to interact with the **PDHG-F** chain and amplify the excluded volume effect to expand the chain backbones. To demonstrate such point, we referred to the equation **ES1**[8] : $C(1/x_s - 2\chi)N^{1/2} = \beta^5 - \beta^3$, where C is the length ratio of the repeat units to Kuhn segments (defined as the equivalent chains that consist of rigid segments via the linkage of flexible covalent bonds) and N is the number of Kuhn length. x_s is defined as the volume ratio of the solvent molecule to the repeat units. The volume of $CHCl_3$ (V_C) is calculated to be 70.30 Å^3; the volume of tetrahydrofuran (V_T) is computed to be 79.55 Å^3; the volume of the repeat units of **PDHG-F** is evaluated to be 1261.86 Å^3. As a result, we accomplished $x_s = 0.056$ for $CHCl_3$ solvent and 0.063 for the tetrahydrofuran solvent respectively. β is defined as the expansion factor in which $\beta > 1$ corresponds to the expanded chains while $0 < \beta < 1$ belongs to the collapsed chains. Commonly, β ranges from 0.33 to 0.7 if the chains are collapsed. [9,10] According to the equation **ES1**, β is determined by the Flory-Huggins interaction parameter χ where high χ indicates the poor compatibility between the solvent and the polymer chains while low χ suggests such good compatibility. In this case, high χ leads to dominantly intramolecular interactions between polymeric segments, which induces the chain collapses. On the contrary, low χ gives rise to dominant interactions between the solvent molecules and the repeat units, which enhances the excluded volume effect and supports the chain expansion or stretching. The χ can be calculated from the equation **ES2**[11]: $\chi = V(\delta_P - \delta_S)^2 / kT$, where V is the monomeric molar volume (1261.86 Å^3 for **PDHG-F**); k is the Boltzmann constant; T is the temperature (298 K). As a result, χ is influenced by the term $(\delta_P - \delta_S)^2$ in which δ_P and δ_S are the solubility parameters of the polymer chains and the solvent molecules respectively. During the molecular dynamic calculation, the solubility parameters are divided into two-dimensional parameters in terms of van de Waals force ($\delta_{P\text{-}vdW}$ and $\delta_{S\text{-}vdW}$) and electrostatic force ($\delta_{P\text{-}E}$ and $\delta_{S\text{-}E}$) according to the calculation method of the COMPASS forcefield. [5] In this case, $(\delta_P - \delta_S)^2$ should be transformed to $(\delta_{P\text{-}vdW} - \delta_{S\text{-}vdW})^2 + 0.25\ (\delta_{P\text{-}E} - \delta_{S\text{-}E})^2$ accodring to the reference[12]. According to the equation $\delta = (CED)^{1/2}$ where CED is the cohesive energy density (involving $\delta_{vdW} = (CED_{vdW})^{1/2}$, $\delta_E = (CED_E)^{1/2}$, referring to the literature[13], we calculated the CED values of the **PDHG-F** chains, the $CHCl_3$ solvent and the tetrahydrofuran solvent (As is shown in **Supplementary Table 1**).

In the light of **Supplementary Table 1**, the term $(\delta_P - \delta_S)^2$ of the **PDHG-F** and $CHCl_3$ pair (**3.49**) is relatively lower than the **PDHG-F** and tetrahydrofuran pair (**4.48**). According to the equations **ES1**, the term $(1/x_s - 2\chi)$ is **8.99** for $CHCl_3$ solution and **4.35** for the tetrahydrofuran. Considering $C > 0$ and $N > 0$, $\beta > 1$ is absolutely satisfied in this case because of the term $\beta^5 - \beta^3 = \beta^3\ (\beta + 1)(\beta - 1) > 0$. Moreover, if we further consider $C \approx 1/9$ and $N \approx 1$ for the rigid rod-like conformation of **PDHG-F** (DP = 9), we will evaluate $\beta \approx 1.242$ for $CHCl_3$ solution and $\beta \approx 1.152$ for tetrahydrofuran solution. Therefore, the **PDHG-F** chains are expanded both in $CHCl_3$ (to more degree)

or tetrahydrofuran solutions. In this case, the chemical shifts of major hydrogen atoms on the **PDHG-F** chains should be the same as (or much approximate to) the ***meso*-DHG4** or ***rac*-DHG4** (**DHG4** as the repeat unit).

Supplementary Table 1. The solubility parameters of the **PDHG-F**, $CHCl_3$ and the tetrahydrofuran.

Compound	CED_{vdw} (J m^{-3})	CED_E (J m^{-3})	δ_{vdw} (cal cm^{-3})$^{1/2}$	δ_E (cal cm^{-3})$^{1/2}$	$(\delta_P - \delta_S)^2$ (cal cm^{-3})
**PDHG-F**	1.987×10^8	1.016×10^7	6.89	1.56	
$CHCl_3$	3.204×10^8	6.517×10^6	8.75	1.23	**3.49**
tetrahydrofuran	3.357×10^8	2.690×10^7	8.95	2.53	**4.48**

It is noted that 1 cal = 4.187 J. All *CED* and δ are the average values in which the standard errors are lower than 0.015 (cal cm^{-3})$^{1/2}$.

Supplementary Note 5. Molecular weight calibration and calculation

The fundamental principle of GPC calibration[14,15] is derived from Mark-Houwink equation (**ES3**):

$$\eta = KM^{\alpha} \qquad \textbf{ES3}$$

In the equation **ES3**, the exponent α is represented as Mark-Houwink constant which is associated with molecular conformation and solvents. K is represented as the constant that is related to polymer species, solvent environment, and temperature. The single polymeric size is linked to hydrodynamic volume V_h, which is proportional to ηM according to Einstein equation[14] (**ES4**):

$$V_h = \frac{\eta M}{2.5\ N_A} \qquad \textbf{ES4}$$

The elution volume is defined[16] by equation **ES5**:

$$\lg M = A_1 + B_1\ V \qquad \textbf{ES5}$$

In the equation **ES5**, A_1 and B_1 are constant. The dependence of molecular weight M on elution volume V can be uncovered by injecting several reference standards which possess extremely narrow molecular weight distribution (For example, its polydispersity index should be lower than 1.05). As the elution time is related to the elution volume, the equation **ES5** transforms into equation **ES6**:

$$\lg M = -kt + b \qquad \textbf{ES6}$$

In the equation **ES6**, k and b are constant ($k > 0$ and $b > 0$). To obtain k and b, we introduced the relationship of accurate exact mass in each *DP* (M_{LP}) to the corresponding elution times (t). **Supplementary Table 2** exhibits these values. Therefore, the new calibration equation of **PDHG-F** was shown below:

$$\lg M_{LP} = -0.128\ t + 6.329 \qquad \textbf{ES7}$$

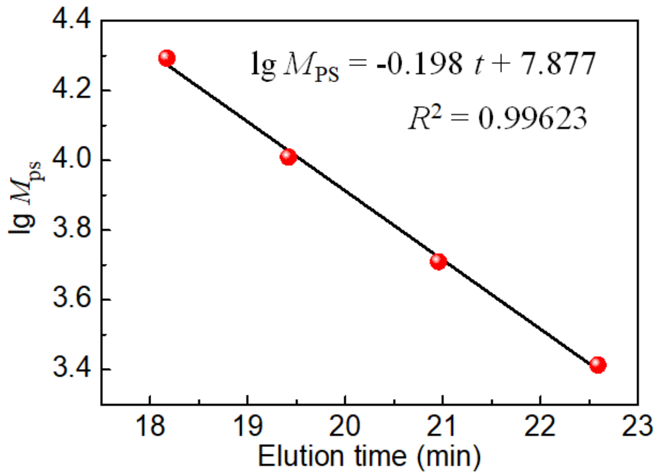

Supplementary Figure 19. The molecular weight calibration curve of the polystyrene standard. We used its relationship during the elution time 18~23 min because such calibration curve will become bend during 16~18 min and 23~24 min. The bend curve will lead to evident error on the slope that is relevant to the calculation

of α_{LP}, Thus, we selected the linear region during 18~23 min that is the scope of DP = 3~6 for **PDHG-F**.

The polystyrene calibration (M_{PS} is represented as the molecular weight of polystyrene, depicted in **Supplementary Figure 19**) is shown in equation **ES8**:

$$\lg M_{PS} = -0.198t + 7.877 \qquad \textbf{ES8}$$

As the hydrodynamic volume V_h of **PDHG-F** should be equivalent to polystyrene (**PS**) at any elution time, we formulated the equation **ES9**:

$$\eta_{PS} M_{PS} = \eta_{LP} M_{LP} \qquad \textbf{ES9}$$

Combining with Mark-Houwink equation, we obtain the equation **ES10**[17] in which K_{PS} and K_{LP} are the constant K for polystyrene and **PDHG-F** respectively; α_{PS} and α_{LP} are the Mark-Houwink constants for polystyrene and **PDHG-F** respectively:

$$\lg M_{LP} = \frac{\alpha_{PS} + 1}{\alpha_{LP} + 1} \lg M_{PS} + \frac{1}{\alpha_{LP} + 1} \lg \frac{K_{PS}}{K_{LP}} \qquad \textbf{ES10}$$

Using **ES8,** we discovered the ingenious relationship between α_{PS} and α_{LP} in equation **ES11**, where k_{PS} and k_{LP} are the slope of calibration equation (like the form **ES6**) for polystyrene and **PDHG-F** respectively:

$$\frac{\alpha_{PS} + 1}{\alpha_{LP} + 1} = \frac{k_{LP}}{k_{PS}} = 0.65 \qquad \textbf{ES11}$$

Supplementary Table 2. The molecular weight calibration of oligomers from dimer to hexamer for PDHG-F systems.

DP	[a] M_n/M_w (*PDI*)	Exact mass (M)	lg M	Elution time t (minute)
2	2130/2155 (1.012)	2248	3.3516	23.223
3	3996/4022 (1.006)	3564	3.55194	21.572
4	5877/5922 (1.008)	4880	3.6886	20.511
5	8154/8215 (1.008)	6196	3.79225	19.777
6	12721/13326 (1.05)	7512	3.87587	19.129

[a] The values of M_n, M_w and *PDI* were obtained from GPC using polystyrene as standard.

We used α_{ps} = 0.714 according to the literature and thus calculated α_{LP} = 1.651 (in the tetrahydrofuran). According to the definition of number-average molecular weight (M_n, **ES12**) and weight-average molecular weight (M_w, **ES13**, Noted that $\int_0^\infty P(M)dM = \mathbf{1}$, $P(M)$ represents the polymeric fraction for the specific *DP*):

$$M_n = \frac{\sum N_i M_i}{\sum N_i} = \int_0^{\infty} p(M) M dM \qquad \textbf{ES12}$$

$$M_w = \frac{\sum N_i M_i^2}{\sum N_i M_i} = \frac{\int_0^{\infty} p(M) M^2 dM}{M_n} \qquad \textbf{ES13}$$

The elution time *t* that corresponds to each specific *DP* (7~12) was evaluated via the extrapolation of the **PDHG-F** calibration equation (shown in **Supplementary Table 3**)

Supplementary Table 3. The calculation of retention time for DP = 7~12.

DP	molar mass (Da)	Elution time (minute)	*DP*	molar mass (Da)	Elution time (minute)
7	8828	18.65	10	12776	17.36
8	10144	18.16	11	14092	17.04
9	11460	17.74	12	15408	16.73

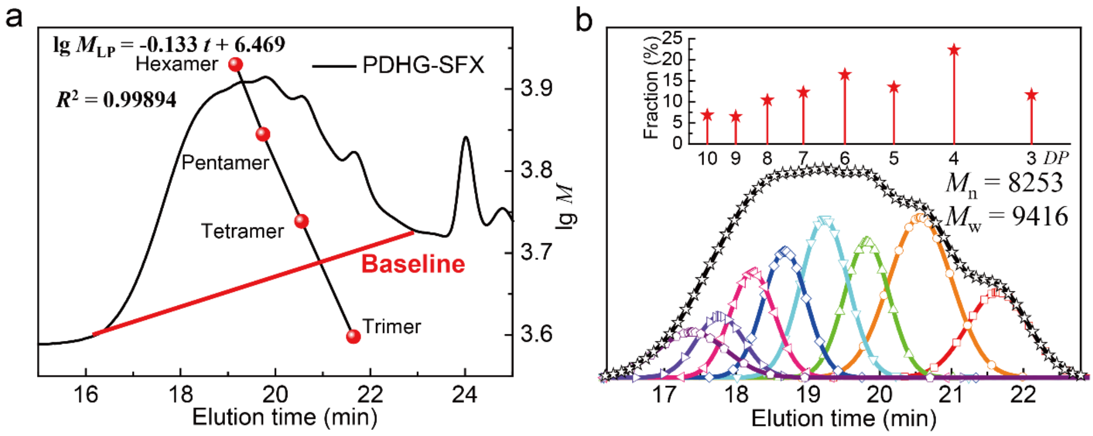

Supplementary Figure 20. The GPC spectra of PDHG-SFX including its calibration. a The calibration equation of **PDHG-SFX** is lg *M* = -0.133 *t* + 6.469. The elution times on *DP* = 3, 4, 5 and 6 (shown in red dots) are extremely approximate to corresponding *DP* in **PDHG-F** systems because the hydrodynamic volume mode of **PDHG-SFX** is the same as that of **PDHG-F**. Because of the Mark-Houwink exponent α = 1.552 (in the tetrahydrofuran solvent), the **PDHG-SFX** was demonstrated as the rod-like chain as well. **b** The DP_n was calculated as 5.85 from GPC analysis and as 5.76 from ^{1}H NMR spectra. The total elution peaks (black line and stars) are divided into a series of gauss peaks (in red, orange, green, cyan, blue, pink, violet and purple lines) with well-defined degree of polymerization 3~10, in terms of the extrapolation of this calibration. The height of red star columns represents the proportion of each *DP*.

Supplementary Note 6. The hydrodynamic radius distribution of PDHG-F chains

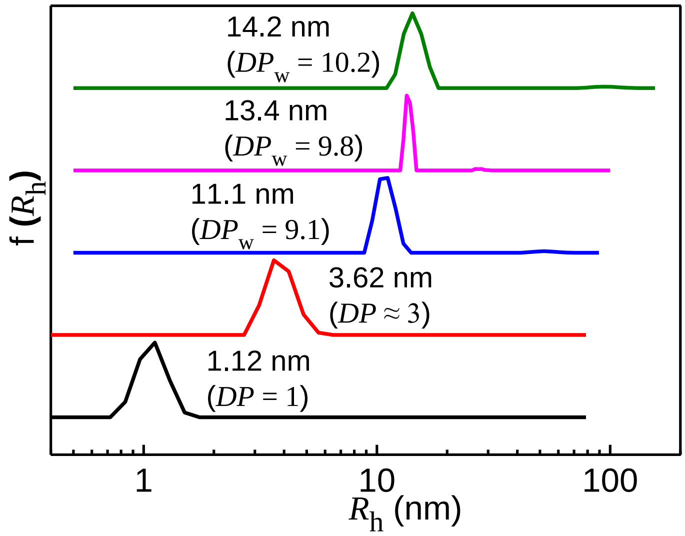

Supplementary Figure 21. The hydrodynamic radius (R_h) distribution of **PDHG-F** with different *DP* values. For higher **PDHG-F** samples, *DP* is replaced by the weight-average of molecular weight (DP_w).

Supplementary Note 7. Mechanistic analysis of the gridization process

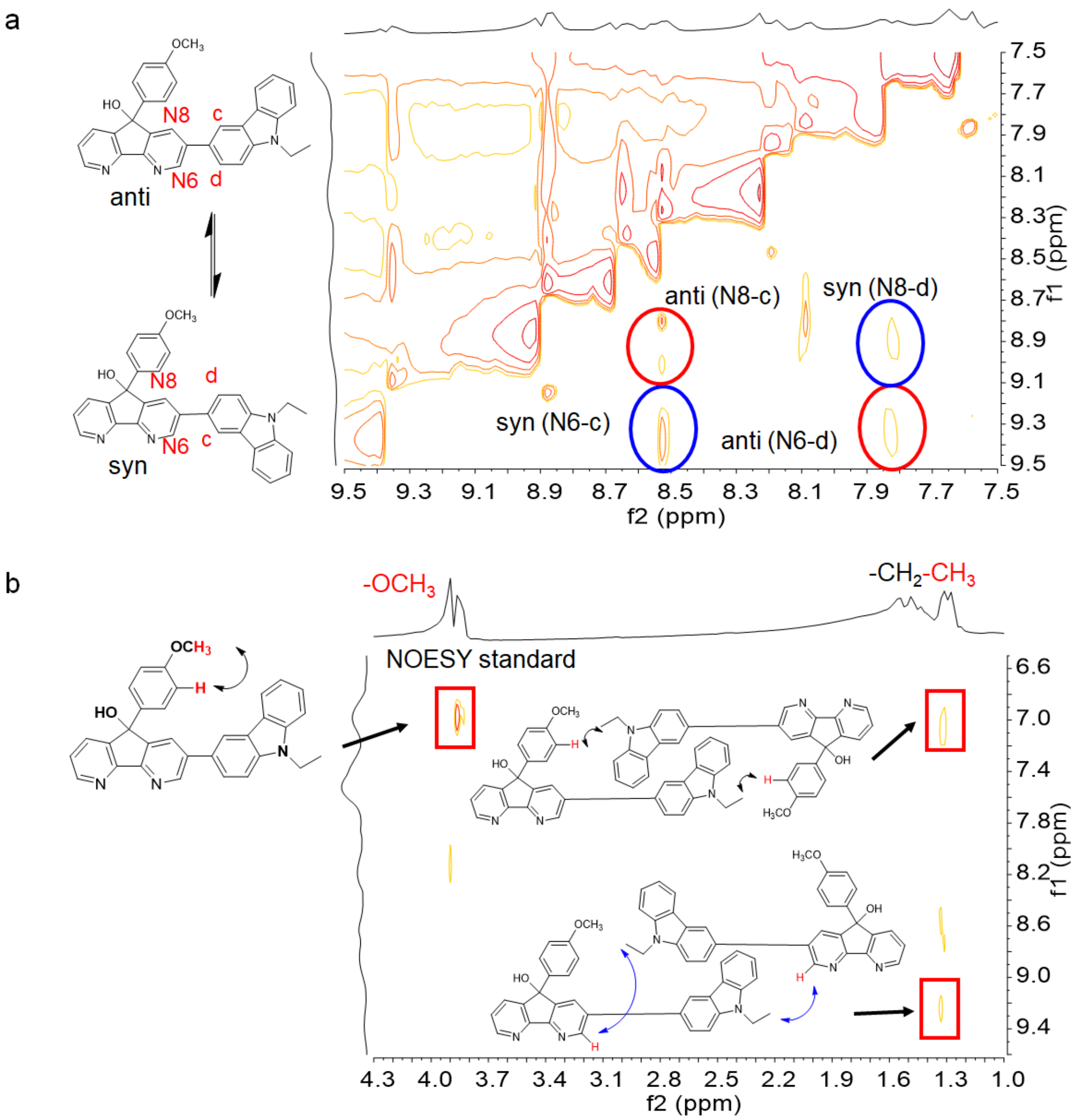

Supplementary Figure 22. The Nuclear Overhauser Effect spectroscopy (NOESY) of protonated MC1. The mixed solvents involve CF_3COOH and $CDCl_3$ in 1:3 volume ratio. **a** In the range of (7.5~9.5, 7.5~9.5). The cross-peaks of (N6, d) and (N8, c) in red circles are consistent with the *anti*-conformation while (N6, c) and (N8, d) in blue circles are in agreement with the *syn*-conformation. **b** In the range of (4.3~1.0, 9.6~6.6). The cross-peak (3.90, 7.10) serves as the standard assigning to the space correlation of two hydrogen atoms between methoxyl and a-protons (intramolecular cases, shown in a black bend arrow). The anti-parallel stacking hypothesis is confirmed by the space correlation [cross-peak (1.30, 7.00)] between the methyl group (on the Cz moiety) and the a-proton (on the methoxybenzenyl group), as well as another correlation [cross-peak (1.30, 9.30)] between the methyl group (on the Cz moiety) and the N6-protons (on the DAF groups). Both space correlations are depicted in blue bend arrows. The cross-peaks are enclosed in the red boxes.

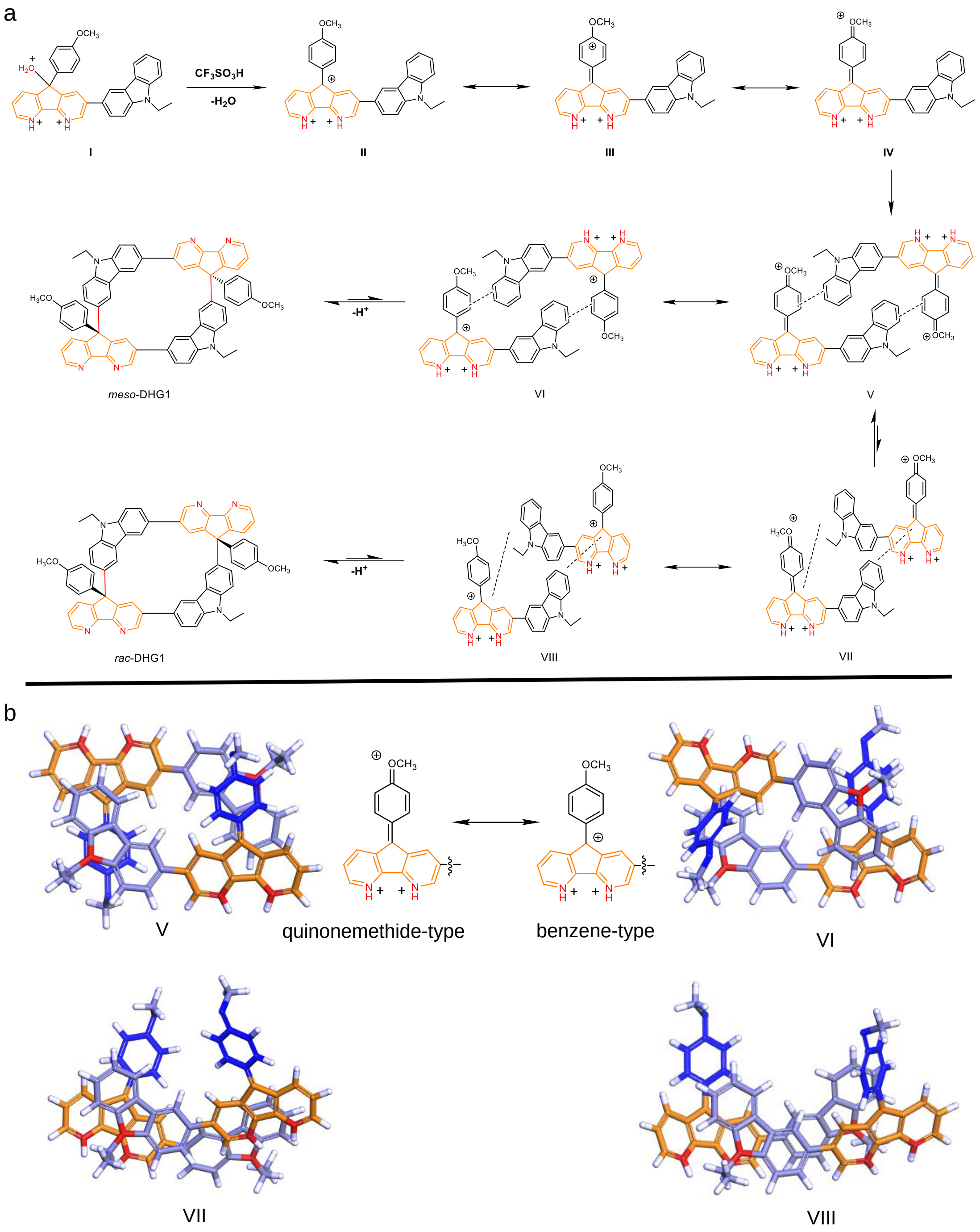

Supplementary Figure 23. The plausible gridization mechanism of MC1 that involves the carbocationic delocalization step. **a** The reaction pathways. **b** The ball-and-stick models of the likely intermediate V, VI, VII and VIII. The methoxybenzene moieties are marked in blue to stand out the stacking mode in the plausible dimer packing process. The tricationic species II easily undergoes the migration of the carbocation (III)[18] to obtain the quinonemethide-type intermediates IV[19] that performs structural planarization between the diazafluorene group and the benzoid moiety (fixed by double bonds). In this case, the cationic methoxybenzyl group probably interacts with the electron-donating carbazole moiety (via pi-pi stacking) and powerfully strengthens the centrosymmetric molecular packing V, which is favorable to *meso*-

selectivity. By contrast, the rotation of methoxybenzyl groups (VI) is unfavorable to the π-π stacking between two **MC1** backbones. For the asymmetrically packing mode (VIII) that generates ***rac*-DHGs**, the quinonemethide-type intermediates IV likely make negligible contributions to enhancing molecular packing (VII), which is unable to efficiently improve *rac*-selectivity. Hence, the quinonemethide-type structures are seemingly favorable to centrosymmetric packing that enhances the *meso*-selectivity, which is confirmed by the *rac*-selectivity of tolyl-based **MC2** substrates without quinonemethide-type species. Under these gridization conditions, the molar ratio of the water (released from MC substrates): CF_3COOH: CF_3SO_3H is about 1:300:75. The 0.3% amount of water should not obviously lower the acidity of the media that is maintained by highly excessive equivalents of strong acids.

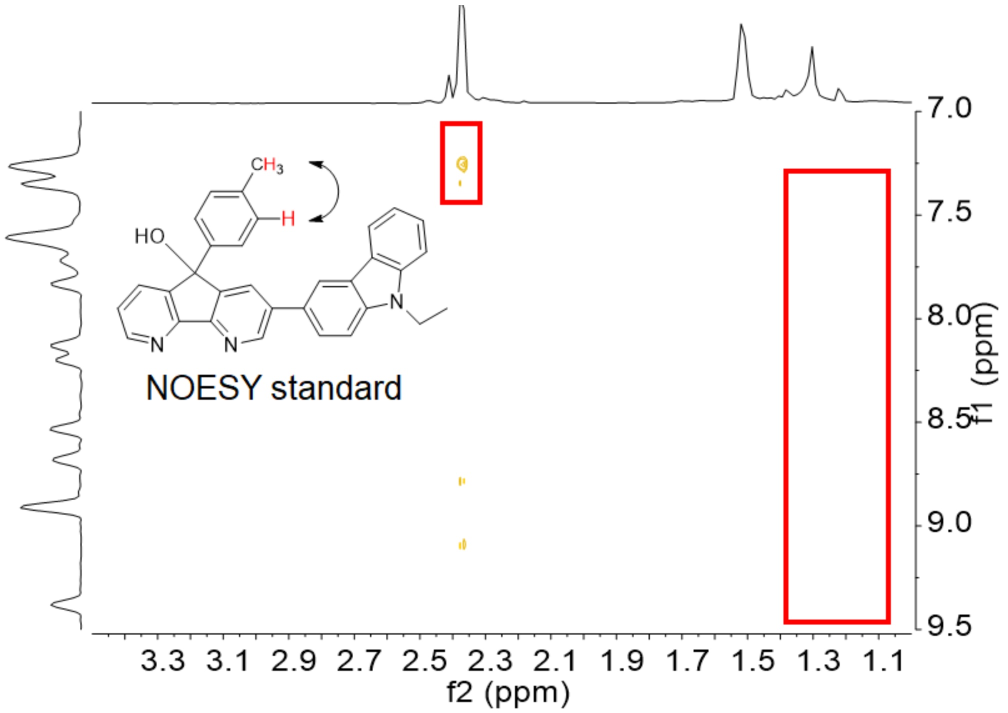

Supplementary Figure 24. The Nuclear Overhauser Effect spectroscopy (NOESY) of protonated MC2 in the range of (3.5~1.0, 9.5~7.0). The cross-peak (2.37, 7.25) serves as the standard assigning to the intramolecular space correlation of two hydrogen atoms between a-protons and methyl groups on tolyl segments. However, no other intermolecular space correlations are observed like protonated **MC1** (in the red boxes), which indicates that no strong intermolecular packing exists in the solution.

Supplementary Note 8. The preliminary polygridization kinetics of polygridization

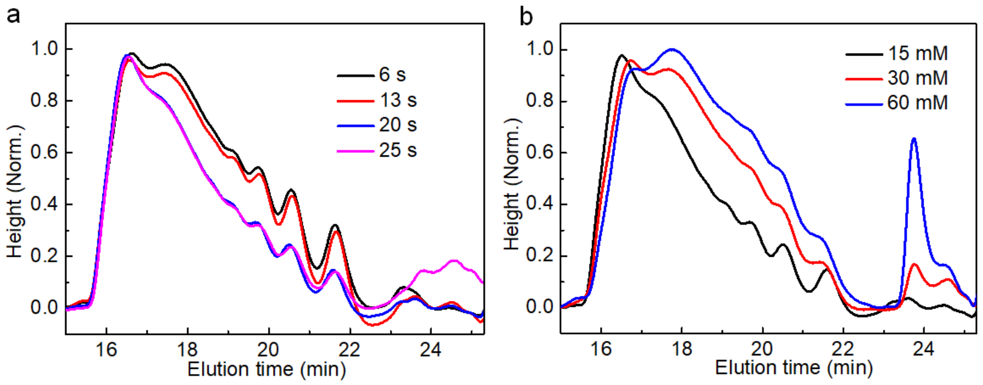

Supplementary Figure 25. The GPC spectra of PDHG-F produced from polygridization under the various conditions. a Under the various reaction times (fixed 15 mM as the monomer concentration). **b** Under the various monomer concentrations (fixed 20 s as the reaction time).

According to **Supplementary Figure 25** and **Supplementary Figure 26**, we tentatively found out the primarily kinetic laws of the polygridization controlled by supramolecular packing. As the polygridization performed in the bimolecular process, we use the second-order reaction rate expression **ES14**: $-\mathrm{d}c/\mathrm{d}t = kc^2$, where the c is defined as the concentration of the **DC-F** monomer; k is the reaction rate constant; and t is the reaction time. If the reaction rate constant is unchangeable during the polygridization process, we will obtain the equation **ES15**: $DP_\mathrm{n} = c_0kt + constant$, where c_0 is the initial concentration of **DC-F** monomers[20]. In this case, c_0 is proportional to DP_n. However, increasing c_0 from 15 to 60 mM leads to decreasing the $\mathrm{DP_n}$ from 9.2 to 8.0, which is contradictory to **ES15**. These results indicate that k should be decreased along with increasing c_0. It is noted that increasing the monomer concentration enables to increase the supramolecular load of acid, which reduces the self-assembly efficiency[21]. As a result, k should be related to the supramolecular efficiency that is reciprocal to the monomer concentration: $k \sim 1/c_0^{a}$, where the exponent a is slightly higher than 1. To further confirm this point, we observed the dependence of reaction times on DP_n. We found that DP_n is increased sharply in the beginning stage (0~6 s, the average polymerization rate of 1.4 DP s^{-1}) while the following stage (6~25 s after the initiation) evidently lowered the polymerization rate to about 0.04 DP s^{-1} on average. Commonly, increasing the reaction time should enhance the DP_n proportionally, like the typical cases[22]. As a consequence, we deduced that the reaction rate constant is high in the beginning stage but becomes much lower in the following polygridization stage, which is linked to the supramolecular packing of **DC-F** monomers in the beginning stage of the polygridization. Therefore, the reaction rate constant should be controlled by the supramolecular assembly efficiency.

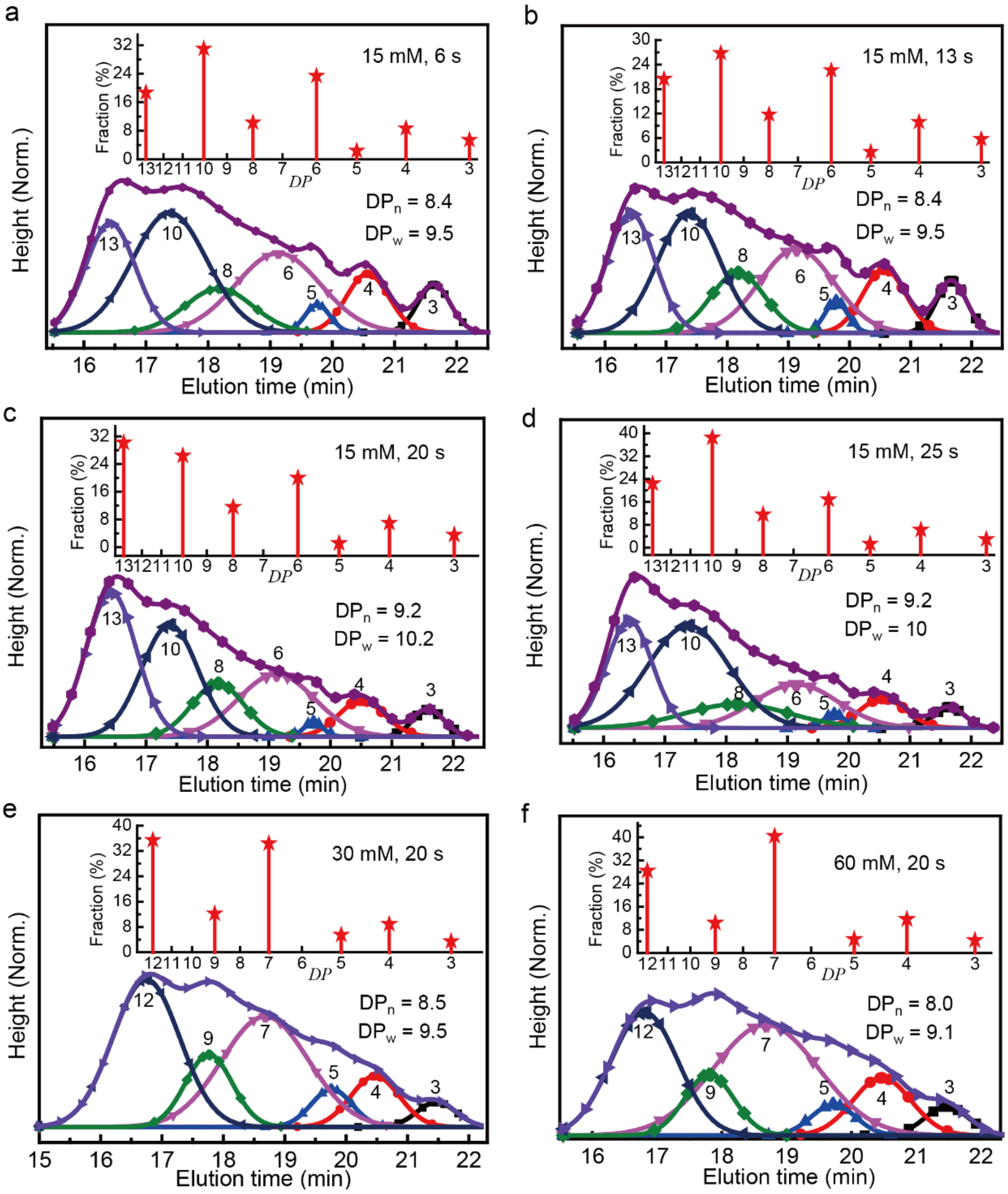

Supplementary Figure 26. The molecular weight of PDHG-F produced from polygridization under the various conditions. The calculation method is similarly based on the **PDHG-F** calibration. **a** the **PDHG-F** samples from the polygridization under 15 mM of monomer concentration and 6 s of reaction times. **b** the **PDHG-F** samples from the polygridization under 15 mM of monomer concentration and 13 s of reaction times. **c** the **PDHG-F** samples from the polygridization under 15 mM of monomer concentration and 20 s of reaction times. **d** the **PDHG-F** samples from the polygridization under 15 mM of monomer concentration and 25 s of reaction times. **e** the **PDHG-F** samples from the polygridization under 30 mM of monomer concentration and 20 s of reaction times. **f** the **PDHG-F** samples from the polygridization under 60 mM of monomer concentration and 20 s of reaction times. For all six figures, the broad elution peak (violet lines) is divided into a series of gauss peaks

(in black, red, blue, magenta, olive, navy and purple lines) with well-defined degree of polymerization, in terms of the extrapolation of this calibration. The height of red star columns represents the proportion of each degree of polymerization *DP*.

Supplementary Note 9. Molecular dynamic simulation of PDHG-F chain collapse under other *DPs*.

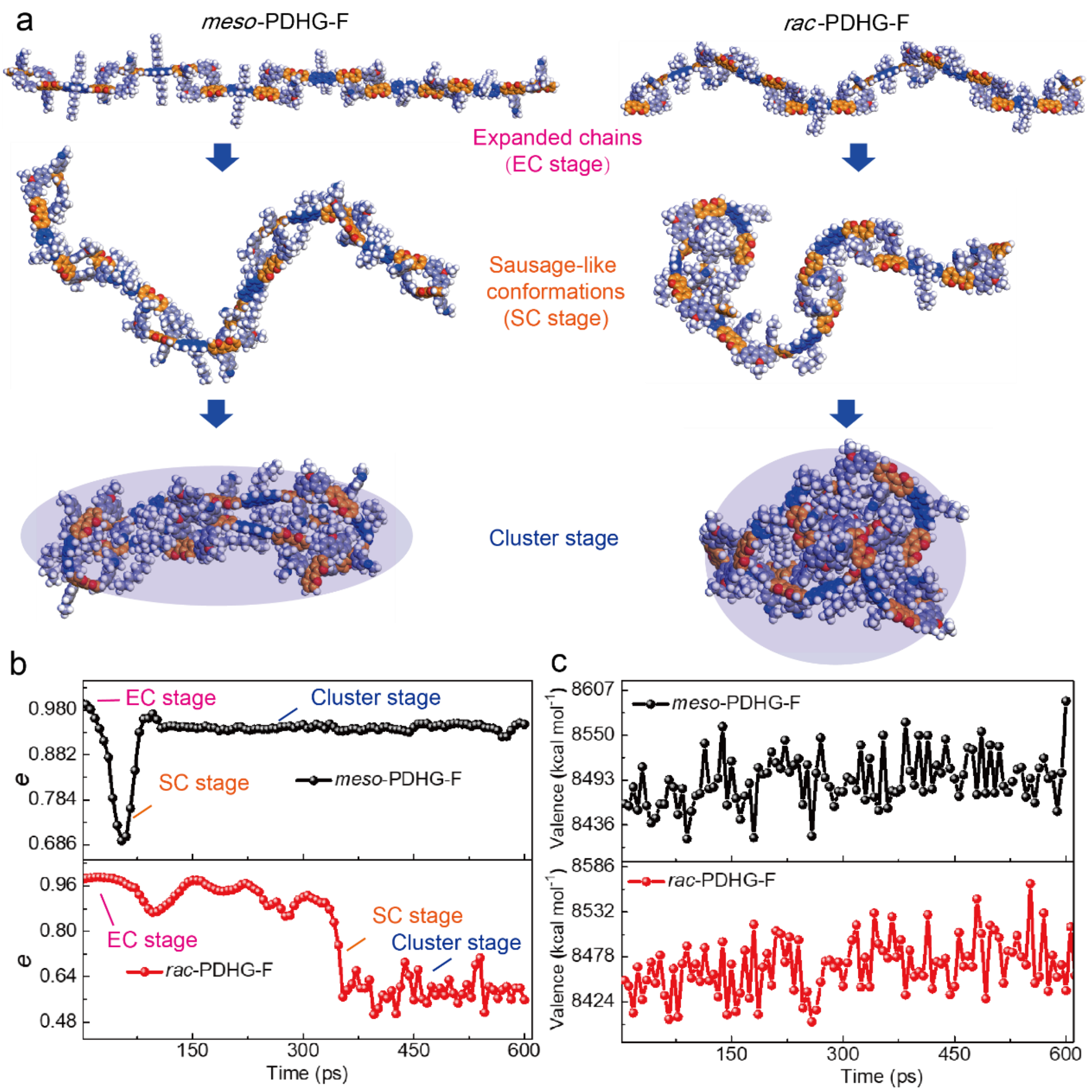

Supplementary Figure 27. The molecular dynamic simulation of the single PDHG-F (*DP* = 7) chain collapse under the vacuum conditions (298 K). a The transformed conformations during the collapsed process. **b** The eccentricity of the ellipsoid model (*e*) for quantitatively evaluating the conformational anisotropy. Black line and dots represent the *e* of ***meso*-PDHG-F**; red line and dots show the *e* of ***rac*-PDHG-F**. **c** The covalent energy (Valence) of the main-chain during the collapse. Black line and dots represent the valence of ***meso*-PDHG-F**; red line and dots show the valence of ***rac*-PDHG-F**.

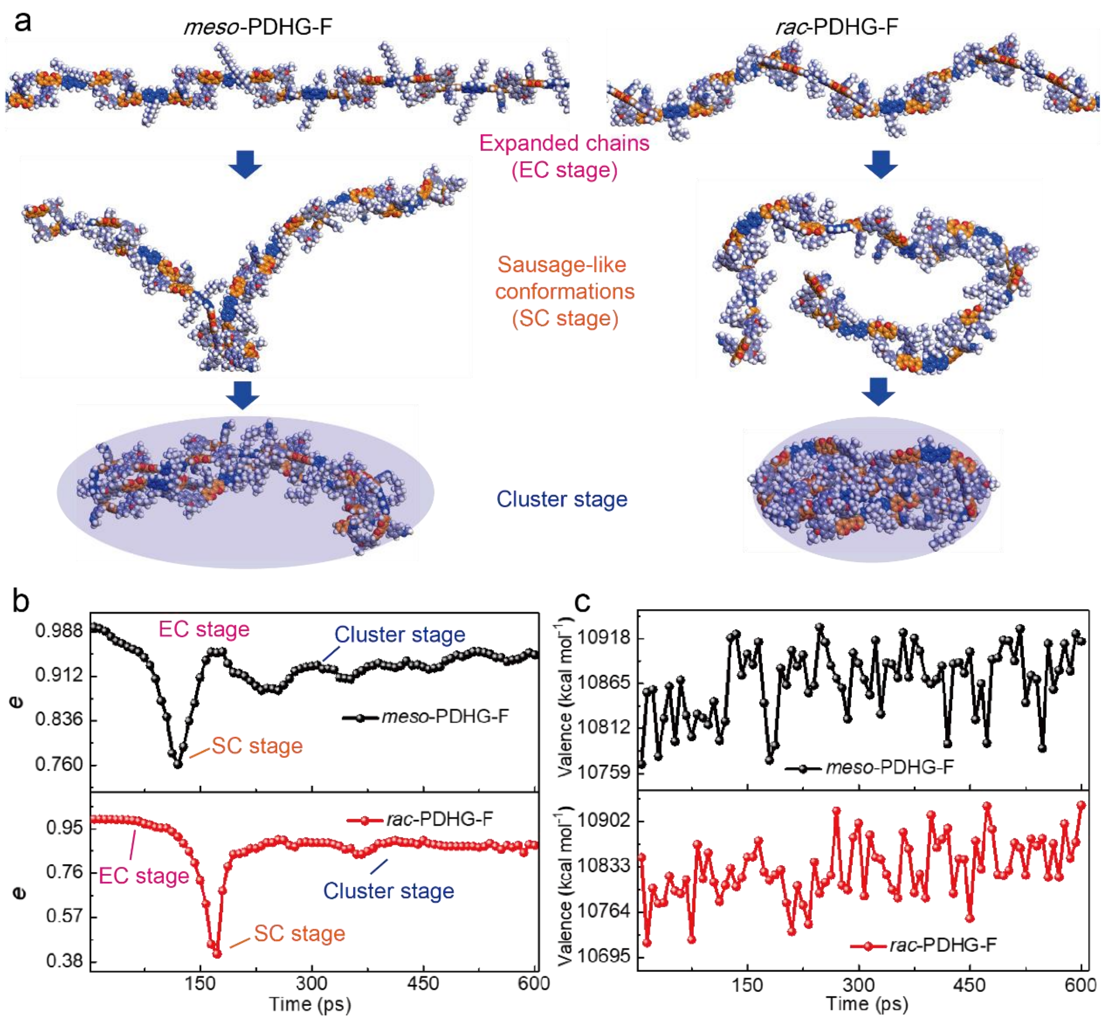

Supplementary Figure 28. The molecular dynamic simulation of the single PDHG-F (*DP* = 9) chain collapse under the vacuum conditions (298 K). a The transformed conformations during the collapsed process. **b** The eccentricity of the ellipsoid model (*e*) for quantitatively evaluating the conformational anisotropy. Black line and dots represent the *e* of ***meso*-PDHG-F**; red line and dots show the *e* of ***rac*-PDHG-F**. **c** The covalent energy (Valence) of the main-chain during the collapse. Black line and dots represent the valence of ***meso*-PDHG-F**; red line and dots show the valence of ***rac*-PDHG-F**.

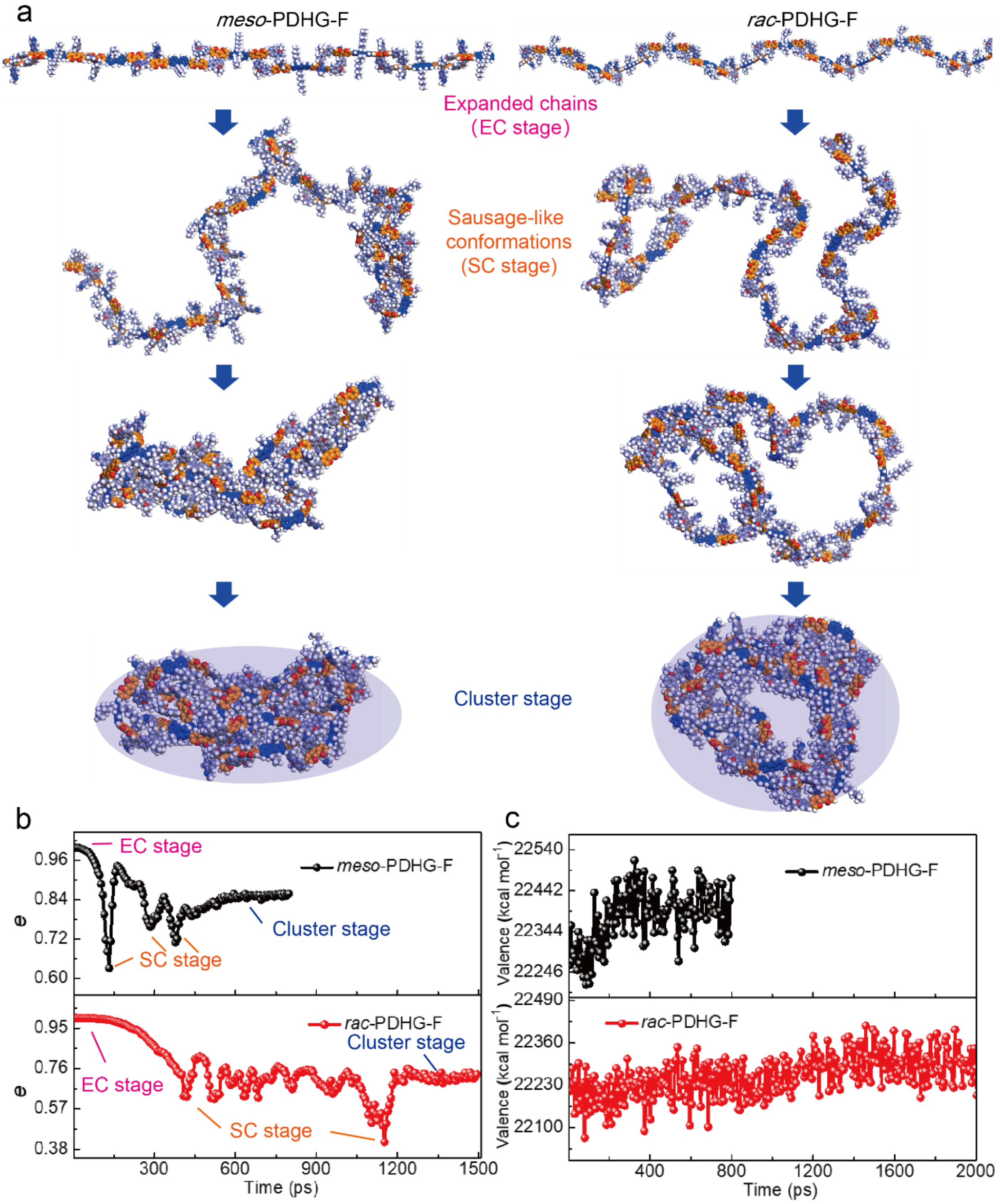

Supplementary Figure 29. The molecular dynamic simulation of the single PDHG-F (*DP* = 18) chain collapse under the vacuum conditions (298 K). a The transformed conformations during the collapsed process. **b** The eccentricity of the ellipsoid model (*e*) for quantitatively evaluating the conformational anisotropy. Black line and dots represent the *e* of ***meso*-PDHG-F**; red line and dots show the *e* of ***rac*-PDHG-F**. **c** The covalent energy (Valence) of the main-chain during the collapse. Black line and dots represent the valence of ***meso*-PDHG-F**; red line and dots show the valence of ***rac*-PDHG-F**.

As the entropic contribution (related to the flexibility of the main-chain conformations) is significant to influence the behaviors of chain collapse[10], we

simulated chain collapse of both ***meso*-PDHG-F** and ***rac*-PDHG-F** respectively under other *DPs* (*DP* = 7 in **Supplementary Figure 27**; *DP* = 9 in **Supplementary Figure 28**; *DP* = 18 in **Supplementary Figure 29**). During the collapsed process of ***meso*-PDHG-F** chains, it is initiated by chain folding based on the *anti*-to-*syn* conformational transitions of **DHG**-edges. Then, the contact of intramolecular segments driven by van de Waals force occurs toward the formation of the cluster. Deeply, increasing the chain length (by increasing the *DP*) enables to increase the number of the folding point (1~2 for *DP* = 7 and 9; 2 for *DP* = 13 and 6 for *DP* = 18) and thus decreased the average eccentricity of their ellipsoid models (in full collapsed equilibrium state) from 0.95 (*DP* = 7), 0.94 (*DP* = 9), 0.91 (*DP* = 13) to 0.85 (*DP* = 18). As a result, increasing the entropic driving force (via increasing the chain length) leads to decreasing the structural anisotropy of the collapsed backbones, which is consistent with the collapse of the traditional polymers[10]. However, all collapsed conformations belong to rod-like shape with higher anisotropy than that of ***rac*-PDHG-F**, independent of the chain length. For ***rac*-PDHG-F**, the similar cyclic skeletons derived from the conformational rotation between ***rac*-DHG** units and fluorene groups are all observed in *DP* = 7, 9 and 18, which are confirmed by the drastically fluctuating eccentricity of the ellipsoid models (*e* = 0.4~0.8). More interestingly, the full colllpased state of ***rac*-PDHG-F** is dependent on the DP where the short chains (*DP* = 7 and 9) transform into compact coil-like conformations (**Supplementary Figure 27** and **Supplementary Figure 28**) but the long chains (*DP* = 13 and 18) lead to toroid conformations. These results suggest that the formation of toroid collapsed conformation requires enough entropic driving force. Moreover, whatever the coil-like or toroid backbones, ***rac*-PDHG-F** displays higher structural isotropy than ***meso*-PDHG-F**. In addition, when *DP* = 7, the collapsed ***rac*-DHG-F** chains have lower covalent energy, 9.4 kcal mol^{-1} per repreating units lower than that of ***meso*-PDHG-F**. For other chain length, ***rac*-PDHG-F** exhibits lower covalent energy as well, with corresponding differences (per repeat unit) involving 3.1 kcal mol^{-1} (*DP* = 9), 7.3 kcal mol^{-1} (*DP* = 13) and 10.8 kcal mol^{-1} (*DP* = 18). These results suggest that ***meso*-PDHG-F** increased the ability to prevent the chain collapse, which indicates higher rigidity. In all, even with entropic effects, the collapsed ***meso*-PDHG-F** still display the rod-like conformation with higher structural anisotropy and more unstably collapsed conformations than those of ***rac*-PDHG-F**.

Supplementary Note 10. NMR spectra for all substrates and products

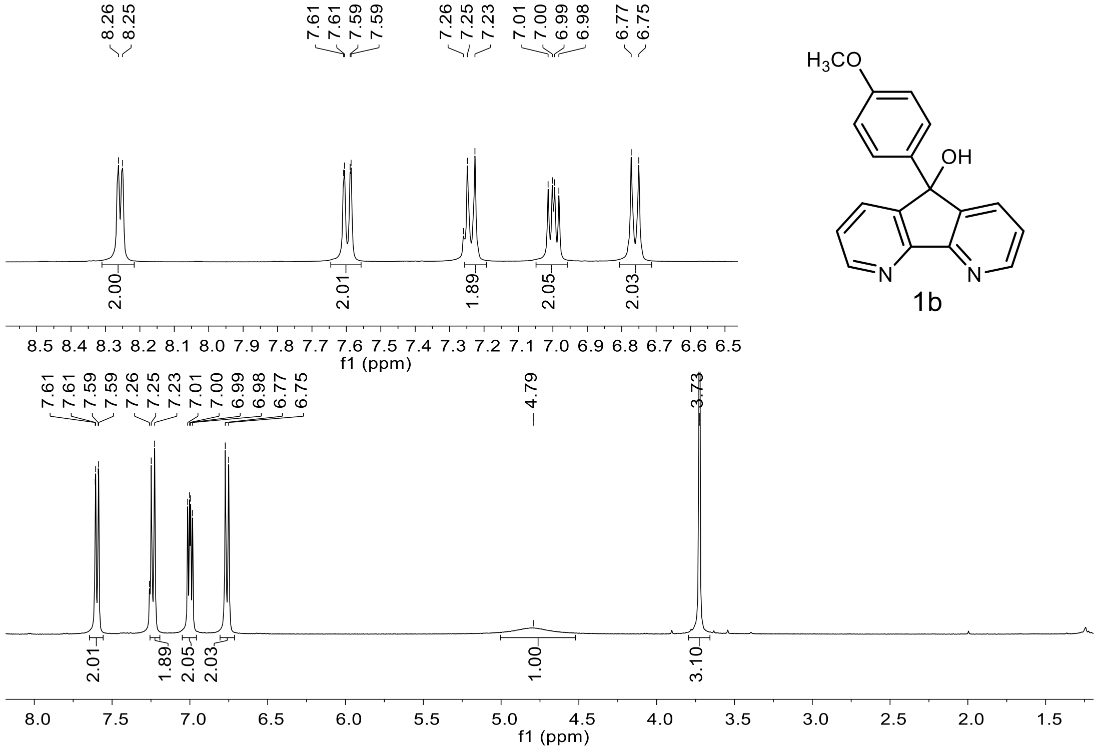

Supplementary Figure 30. ^{1}H NMR spectra for **1b** in $CDCl_3$

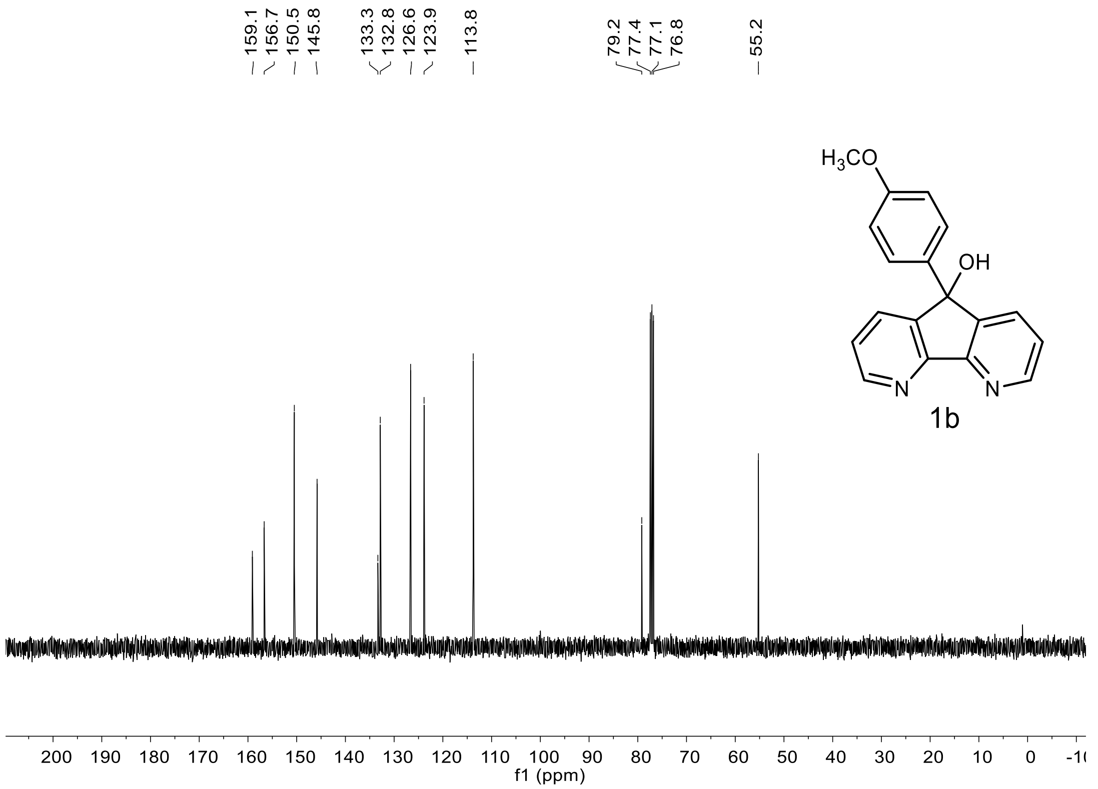

Supplementary Figure 31. ^{13}C NMR spectra for **1b** in $CDCl_3$

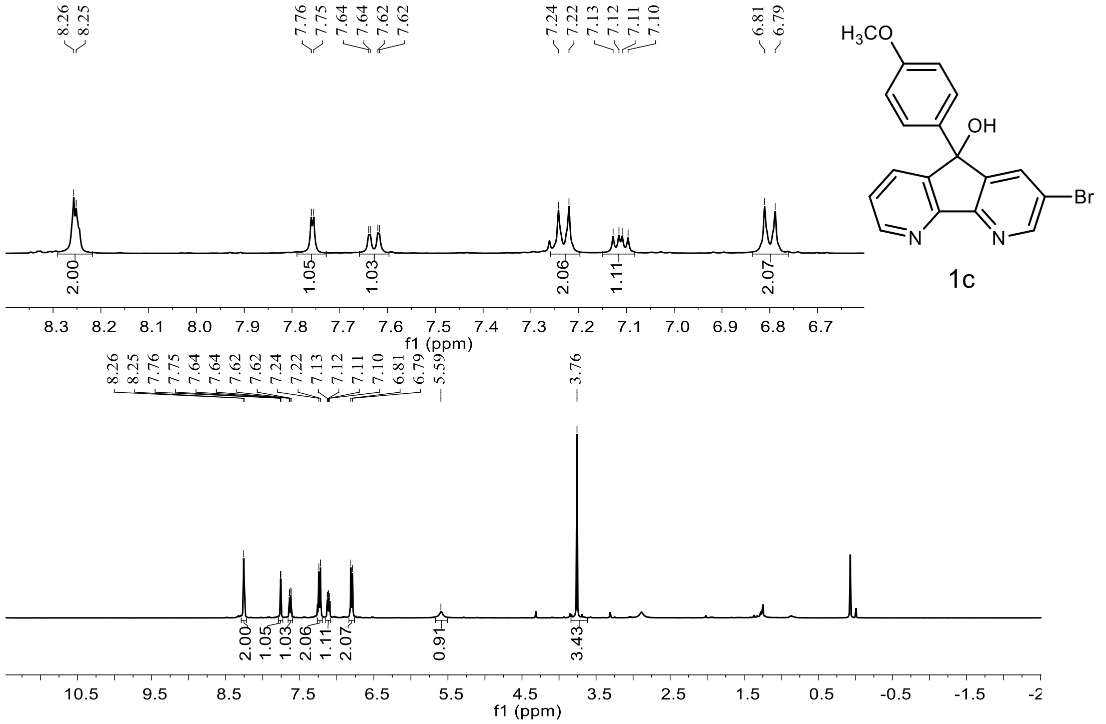

Supplementary Figure 32. ^{1}H NMR spectra for **1c** in $CDCl_3$

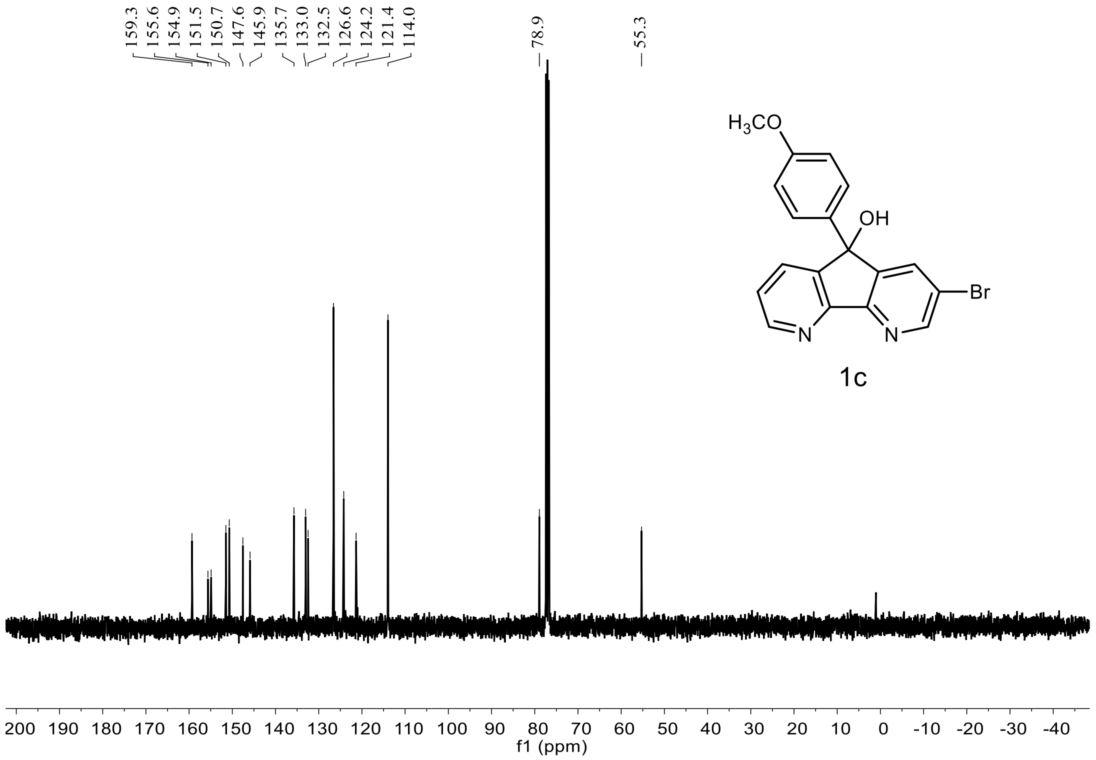

Supplementary Figure 33. ^{13}C NMR spectra for **1c** in $CDCl_3$

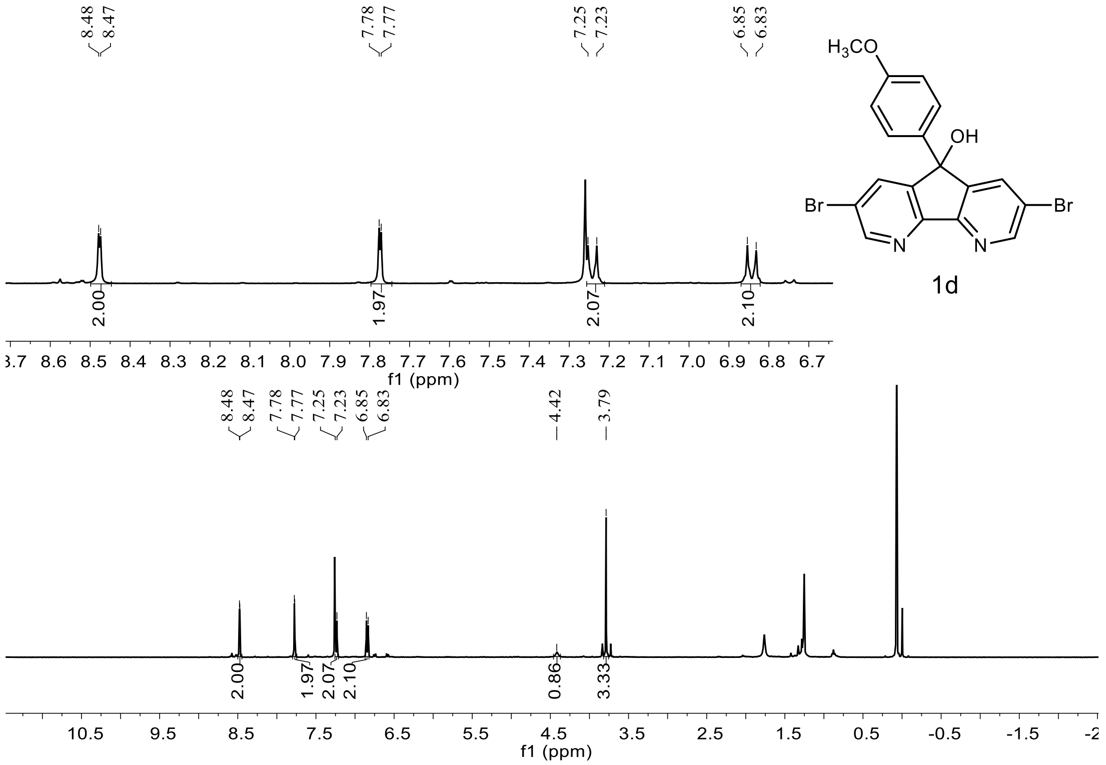

Supplementary Figure 34. ^{1}H NMR spectra for **1d** in $CDCl_3$

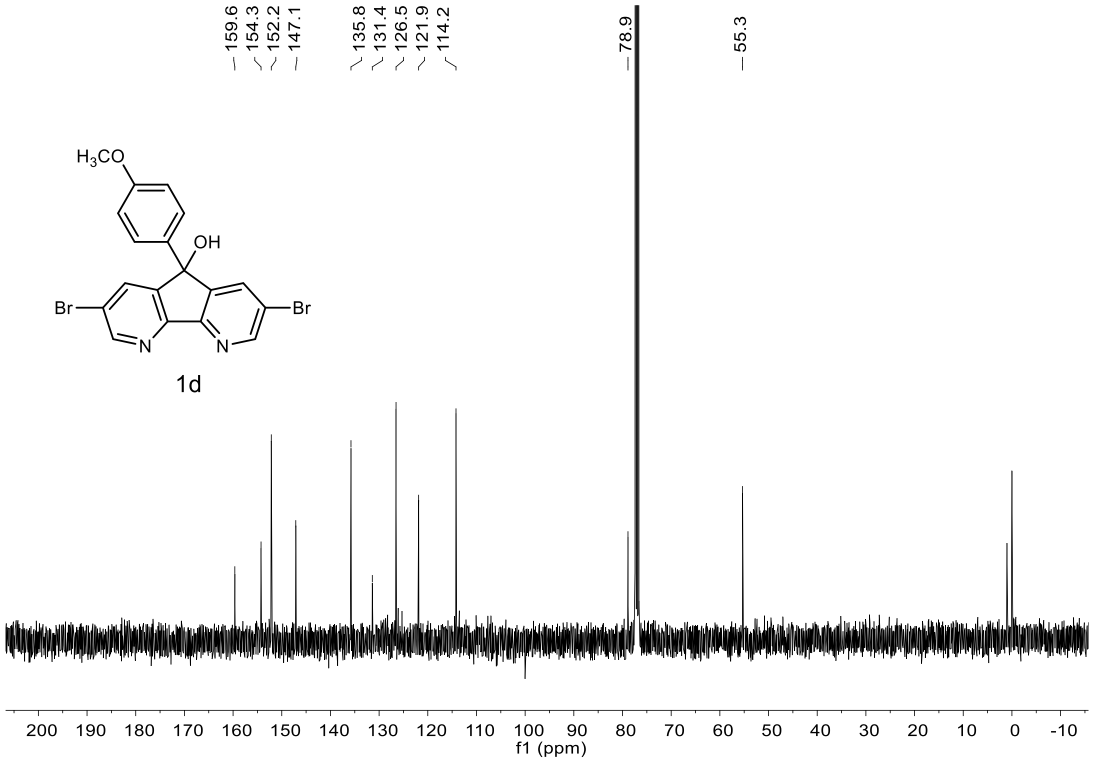

Supplementary Figure 35. ^{13}C NMR spectra for **1d** in $CDCl_3$

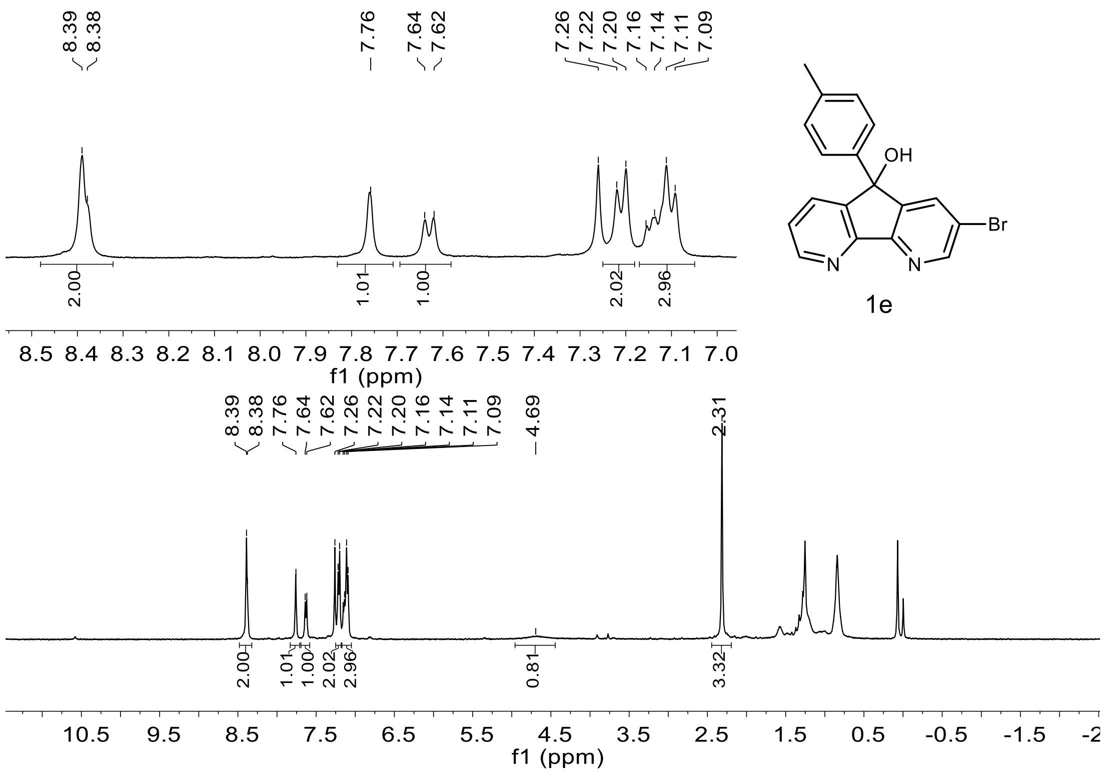

Supplementary Figure 36. ^{1}H NMR spectra for **1e** in $CDCl_3$

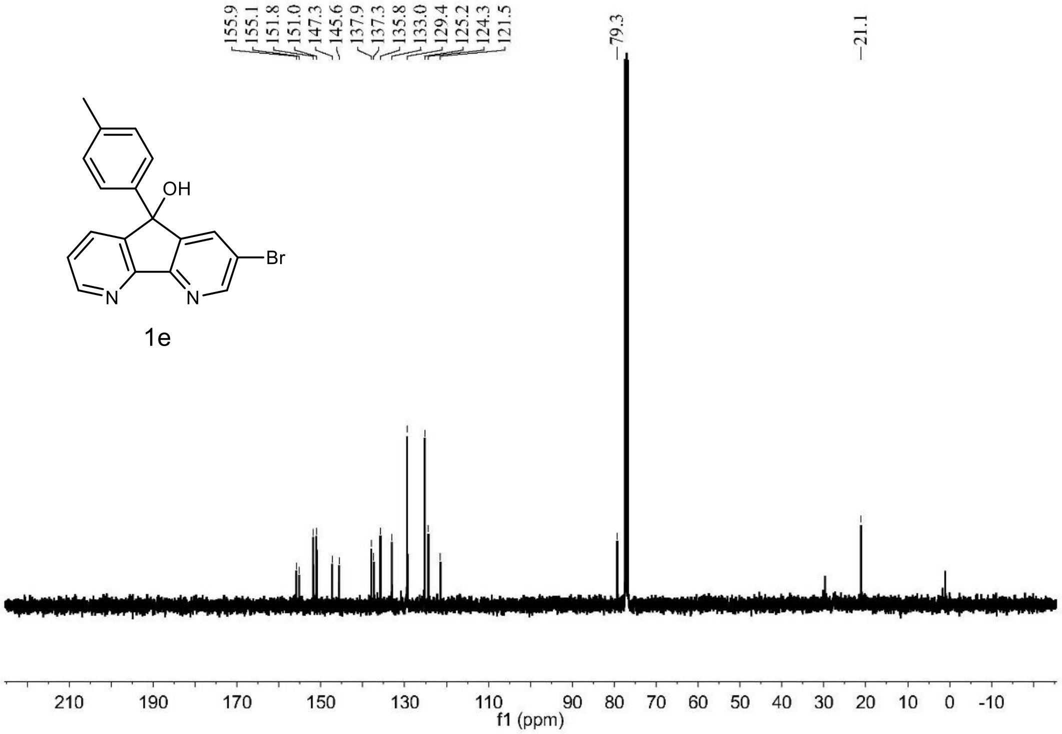

Supplementary Figure 37. ^{13}C NMR spectra for **1e** in $CDCl_3$

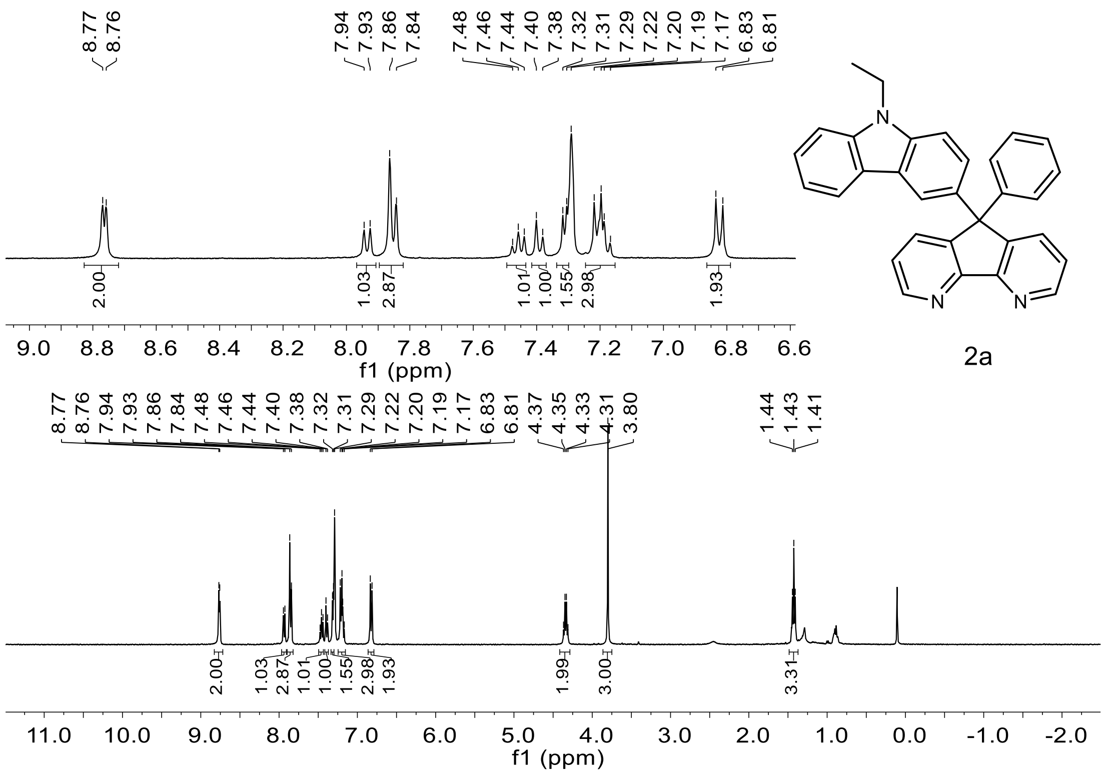

Supplementary Figure 38. ^{1}H NMR spectra for **2a** in $CDCl_3$

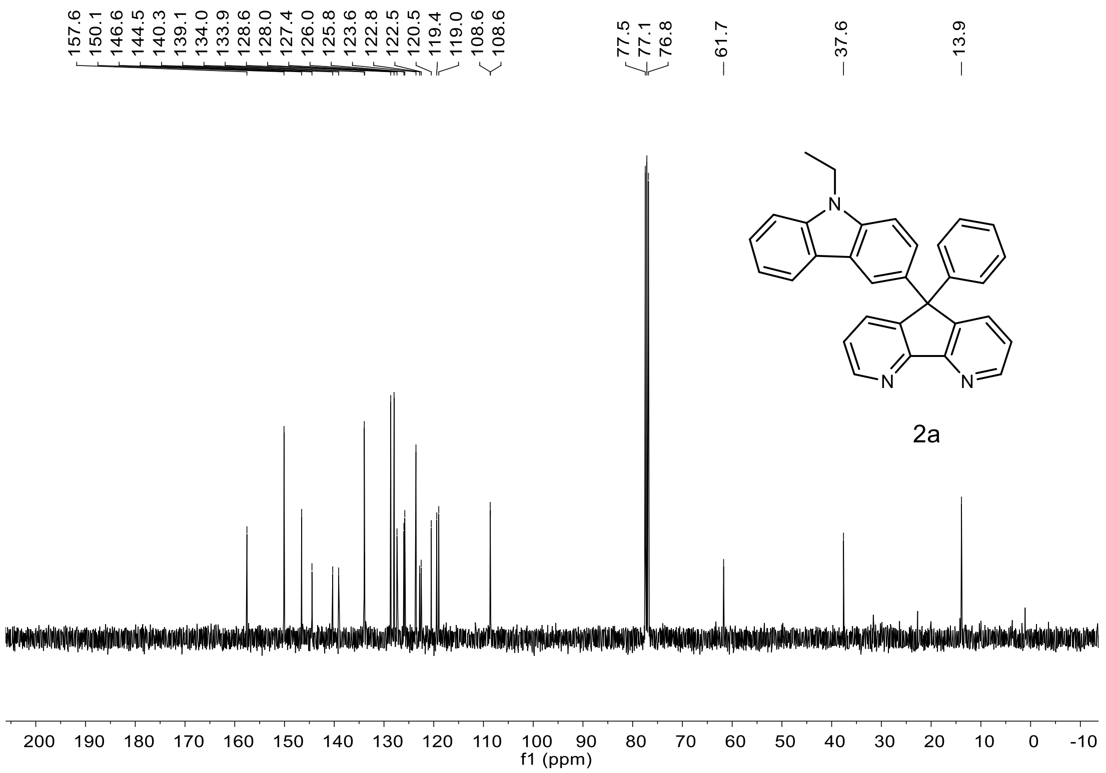

Supplementary Figure 39. ^{13}C NMR spectra for **2a** in $CDCl_3$

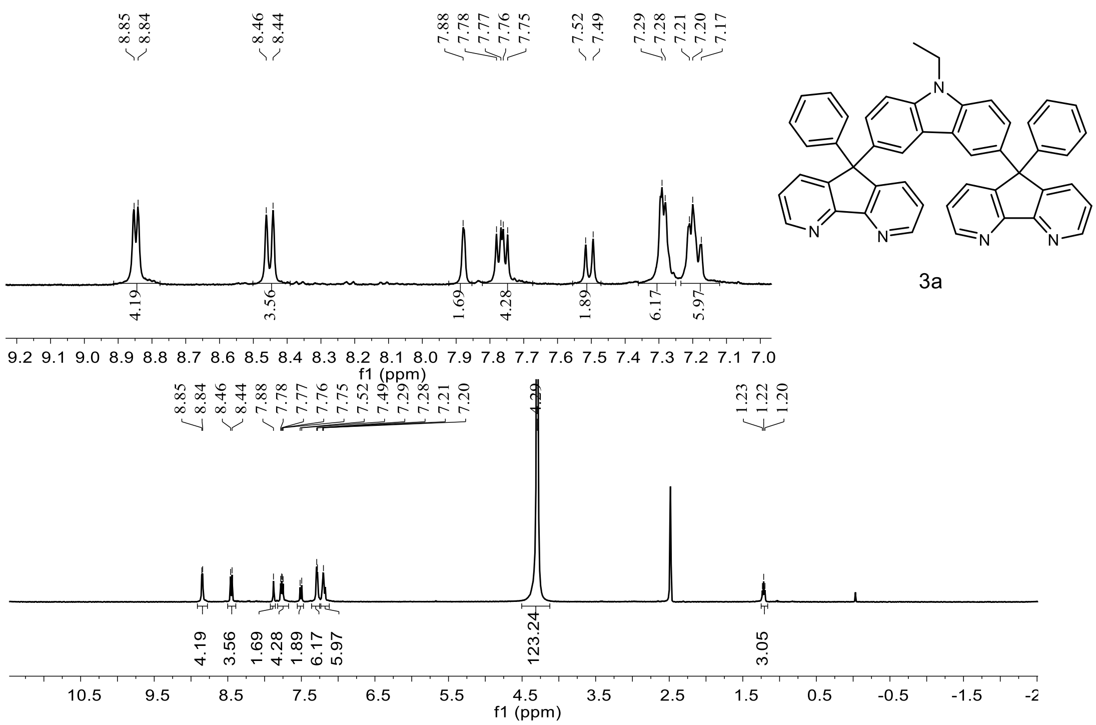

Supplementary Figure 40. ^{1}H NMR spectra for **3a** in d_6 –DMSO

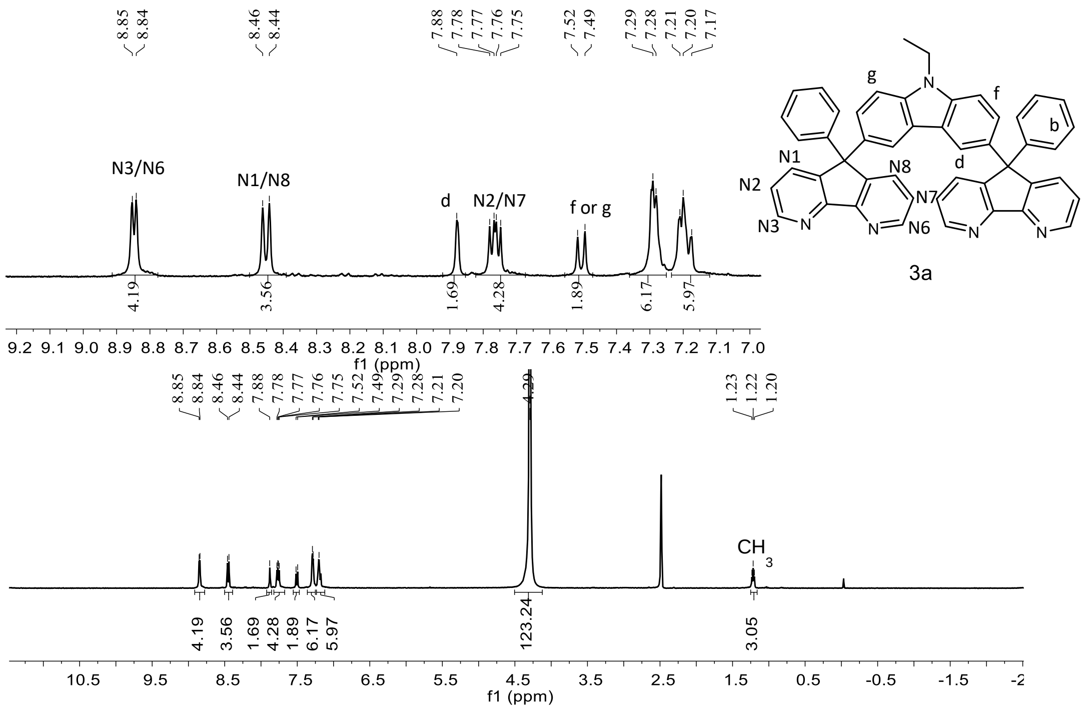

Supplementary Figure 41. The analysis of ^{1}H NMR spectra for 3a. It is noted that the solubility of **3a** is extremely poor (even using CF_3COOH) and they are slightly soluble in DMSO. Even so, the white insoluble powder are precipitated during the ^{1}H NMR characterization which could interfere the results. The hydrogen signals of methylene on the carbazole can be only assigned to be at 4.29 ppm but with drastically large integration. Such signal is a singlet peak. Thus, the methylene signal can be overlapped with this peak. However, we can assign other peaks that testify this structure.

For example, the 8.85 ppm peak with 5.2 Hz of coupling constant is assigned to N3/N6 positions of DAF groups. The 8.46~8.44 ppm peak (J = 8.0 Hz) is assigned to N1/N8-sites on the DAF groups. The single signal at 7.88 ppm is merely assigned to be d-sites on the carbazole group because no other hydrogen atoms belong to the single signal. The dd peak of the signal at the 7.78 ~ 7.75 ppm is assigned to N2/N7-sites on the DAF groups because their two coupling constant 5.2 Hz and 8.0 Hz are corresponding to the coupling of N3-N2 (or N6-N7) and N2-N1 (or N7-N8). The doublet peak at 7.62 ~ 7.59 ppm is assigned to f or g-sites at the carbazole group. The teiplet signal at the 1.23 ~ 1.20 ppm is assigned to the methyl group on the carbazole segment. What's more, the integration ratio of N3/N6 to i (meanwhile, N3/N6 to f or g) is equal to 2. Similarly, the integration ratio of N3/N6 to methyl group is equal to 4:3 instead of 2:3. Therefore, the 3a structure is the disubstitution of DAF on the ethyl-carbazole in the Friedel-Crafts reaction.

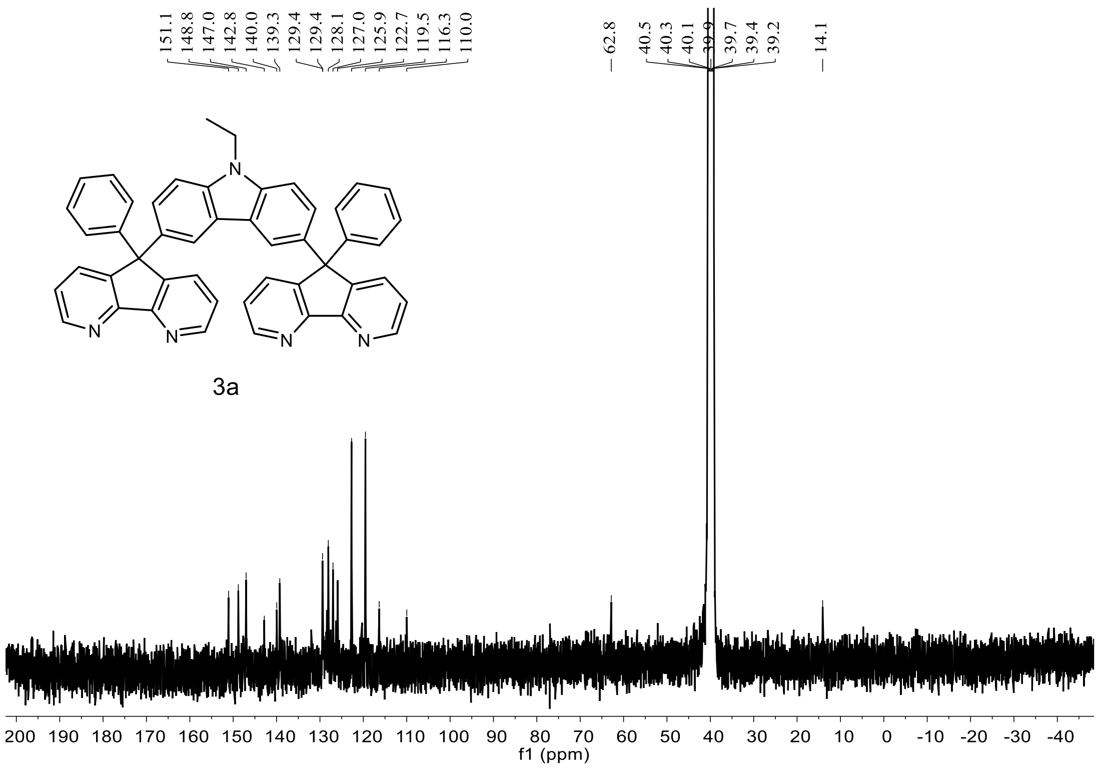

Supplementary Figure 42. ^{13}C NMR spectra for 3a in d_6 –DMSO. Analogously, due to extremely poor solubility, the ^{13}C NMR spectra is not good. However, based on the deduction of the 1H NMR spectra, we considered that the signal ranging from 40.3 to 39.2 ppm should be assigned to the methylene group (at the carbazole segment) that is overlapped by the solvent DMSO (39.52 ppm). Considering such carbon signal is at 37.6 ppm for **2a** (in $CDCl_3$ solvent), this signal is indeed likely to be distributed at the 40.3 ~ 39.2 ppm.

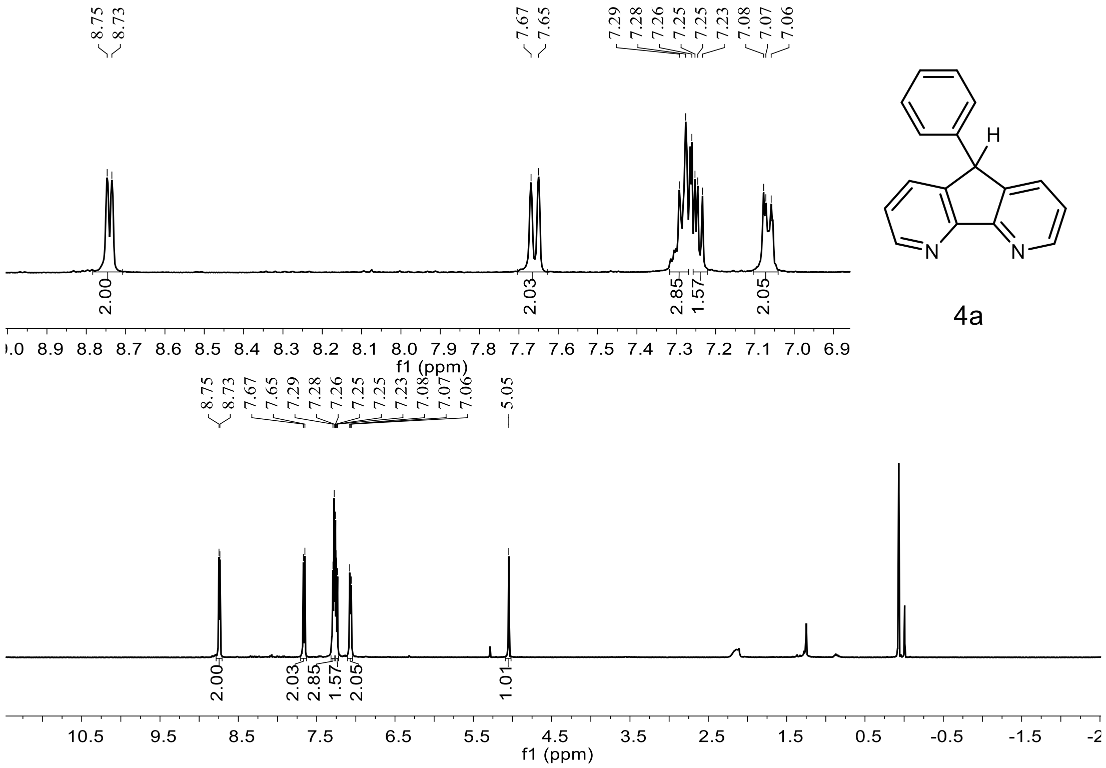

Supplementary Figure 43. ^{1}H NMR spectra for **4a** in $CDCl_3$

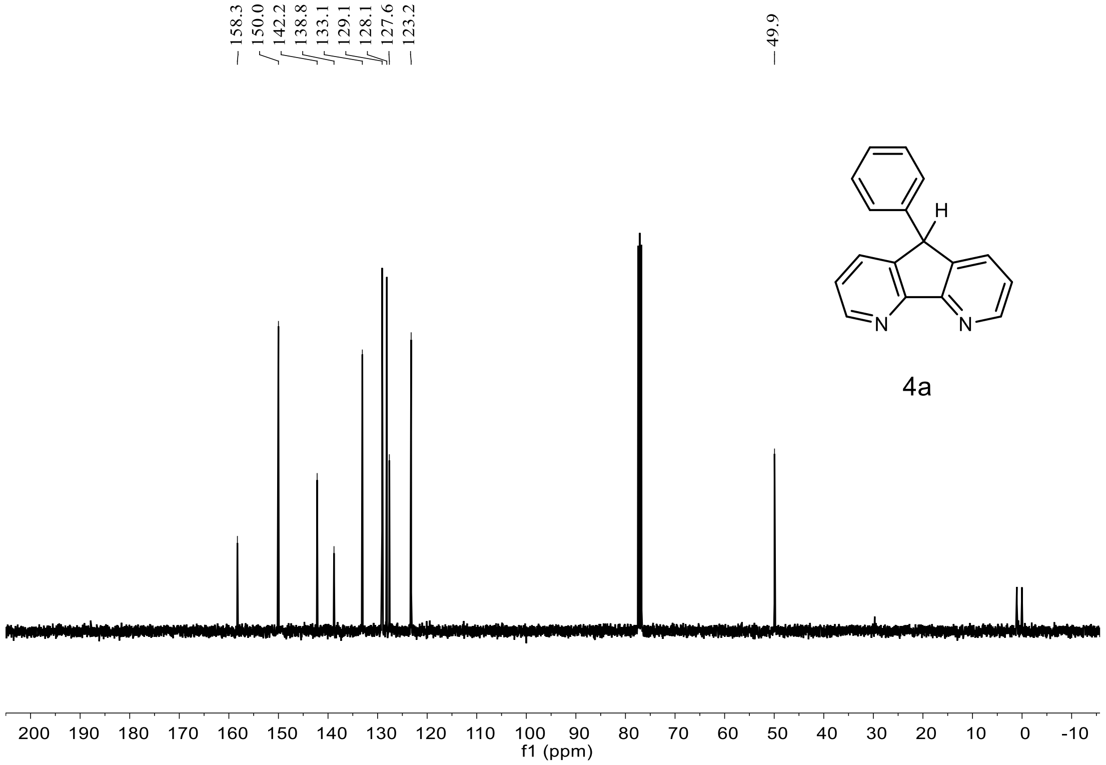

Supplementary Figure 44. ^{13}C NMR spectra for **4a** in $CDCl_3$

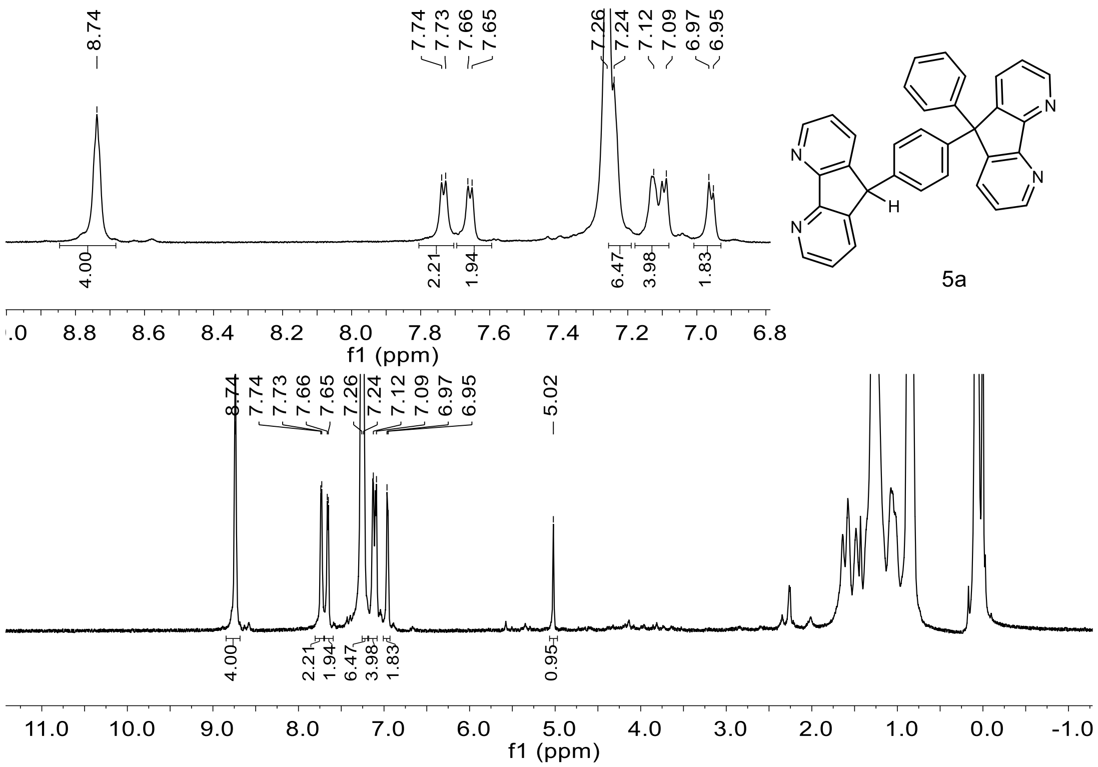

Supplementary Figure 45. ^{1}H NMR spectra for **5a** in $CDCl_3$

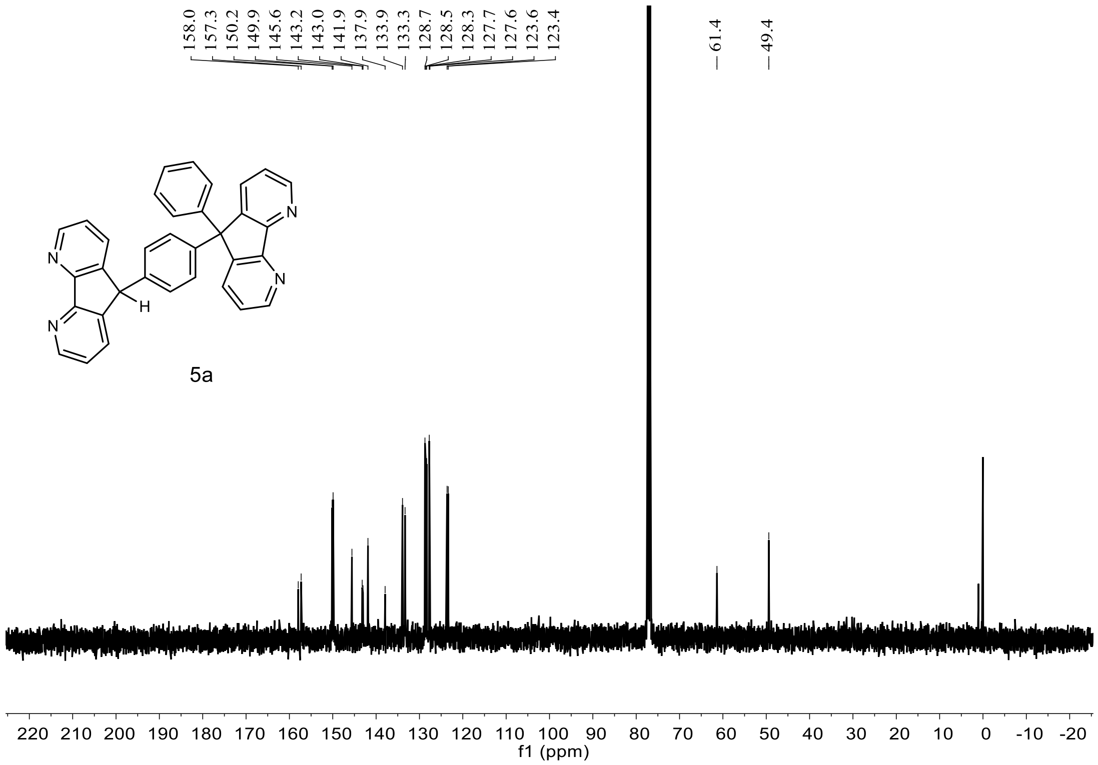

Supplementary Figure 46. ^{13}C NMR spectra for **5a** in $CDCl_3$

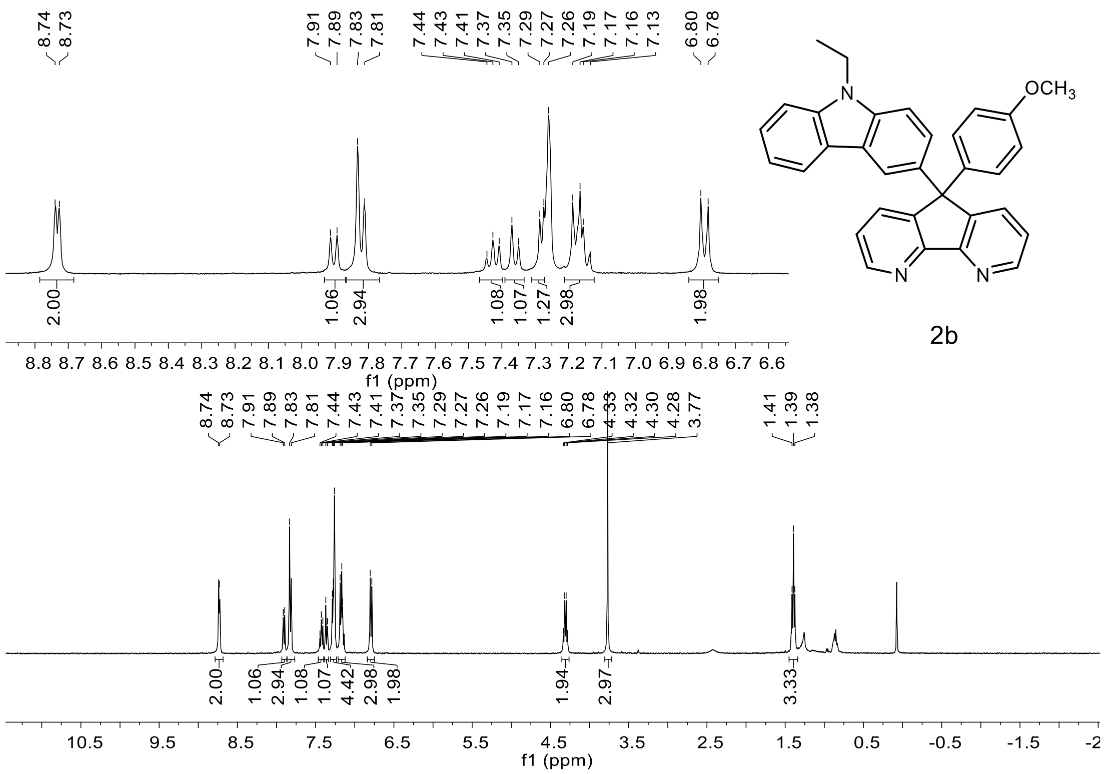

Supplementary Figure 47. ^{1}H NMR spectra for **2b** in $CDCl_3$

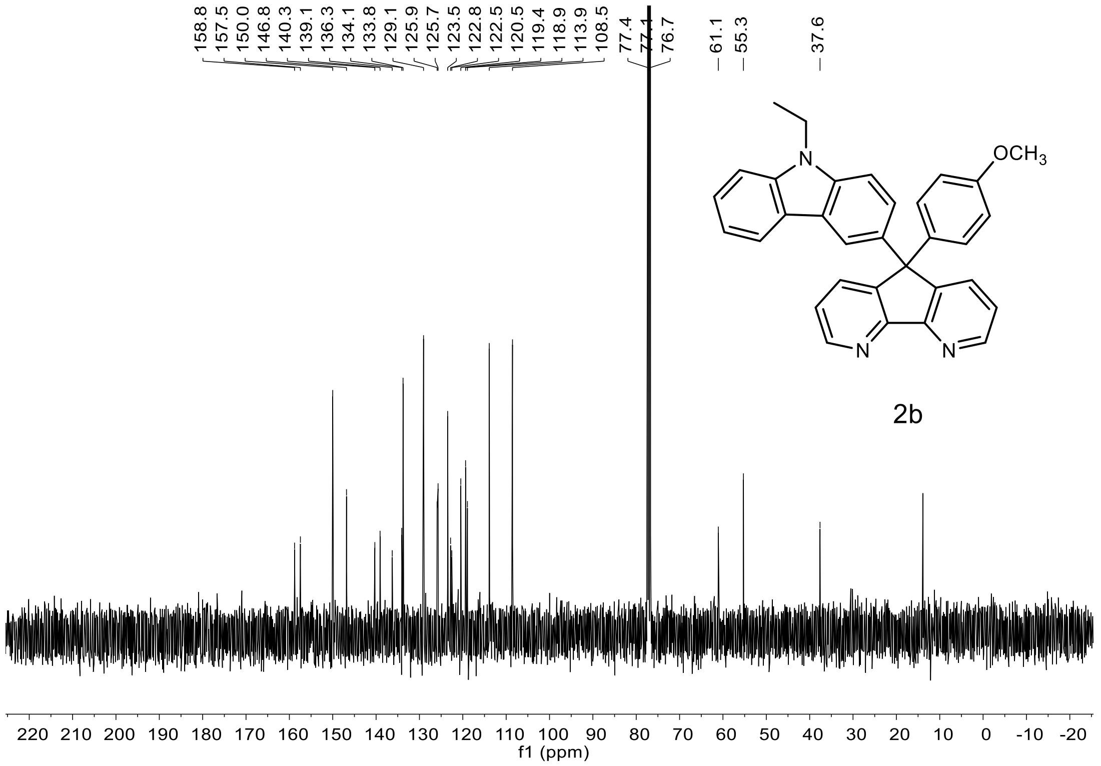

Supplementary Figure 48. ^{13}C NMR spectra for **2b** in $CDCl_3$

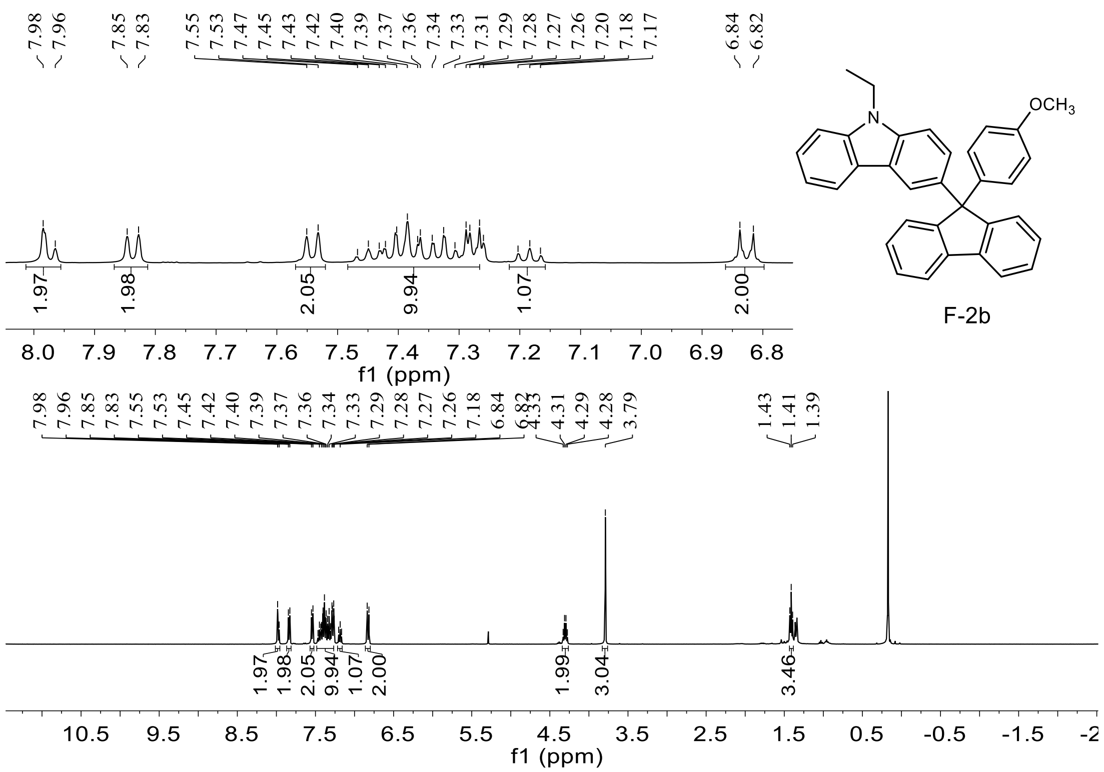

Supplementary Figure 49. ^{1}H NMR spectra for **F-2b** in $CDCl_3$

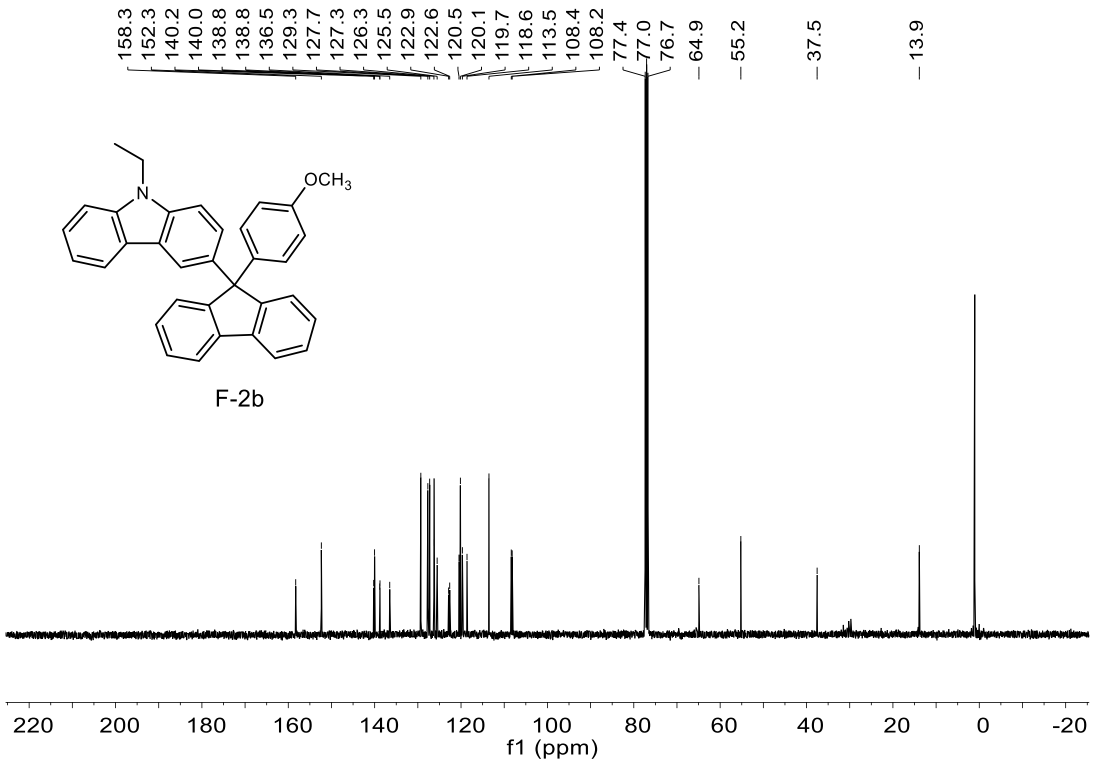

Supplementary Figure 50. ^{1}H NMR spectra for **F-2b** in $CDCl_3$

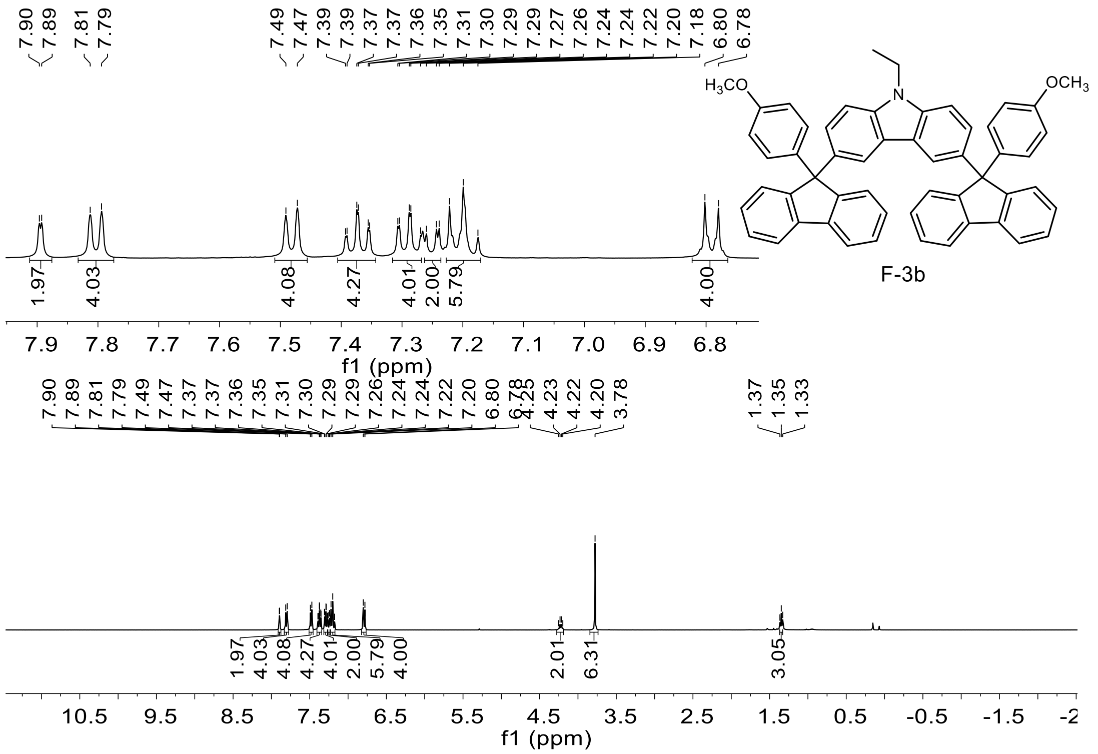

Supplementary Figure 51. ^{1}H NMR spectra for **F-3b** in $CDCl_3$

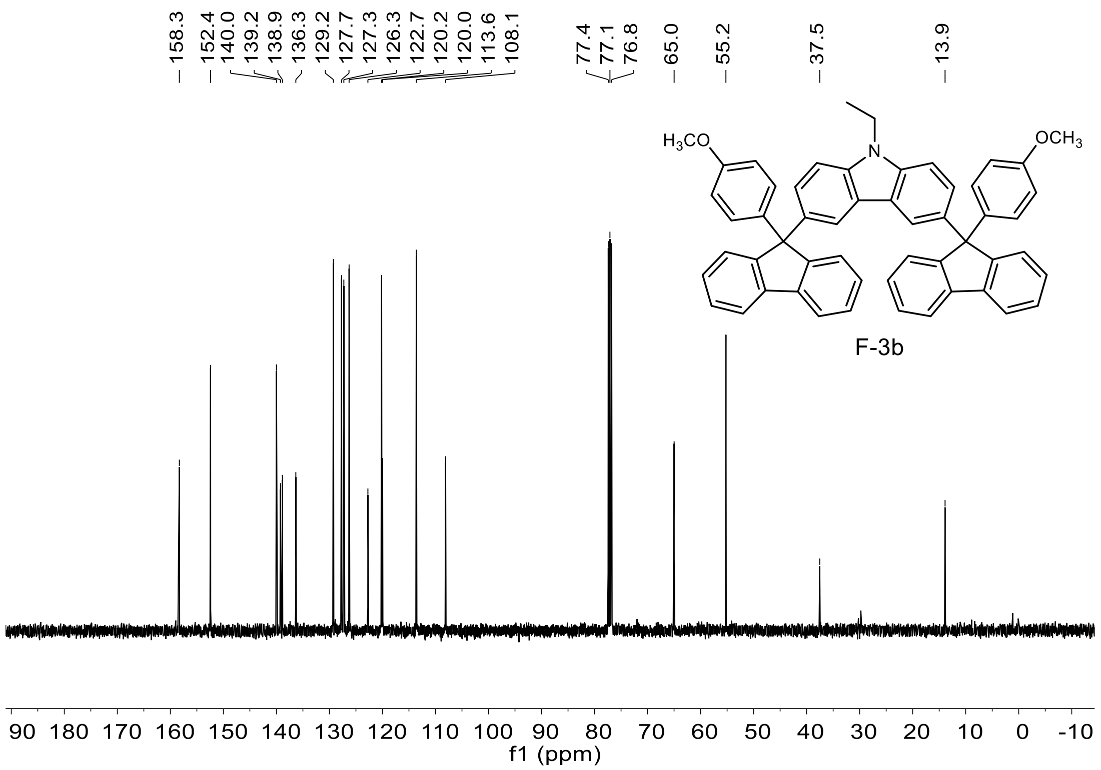

Supplementary Figure 52. ^{1}H NMR spectra for **F-3b** in $CDCl_3$

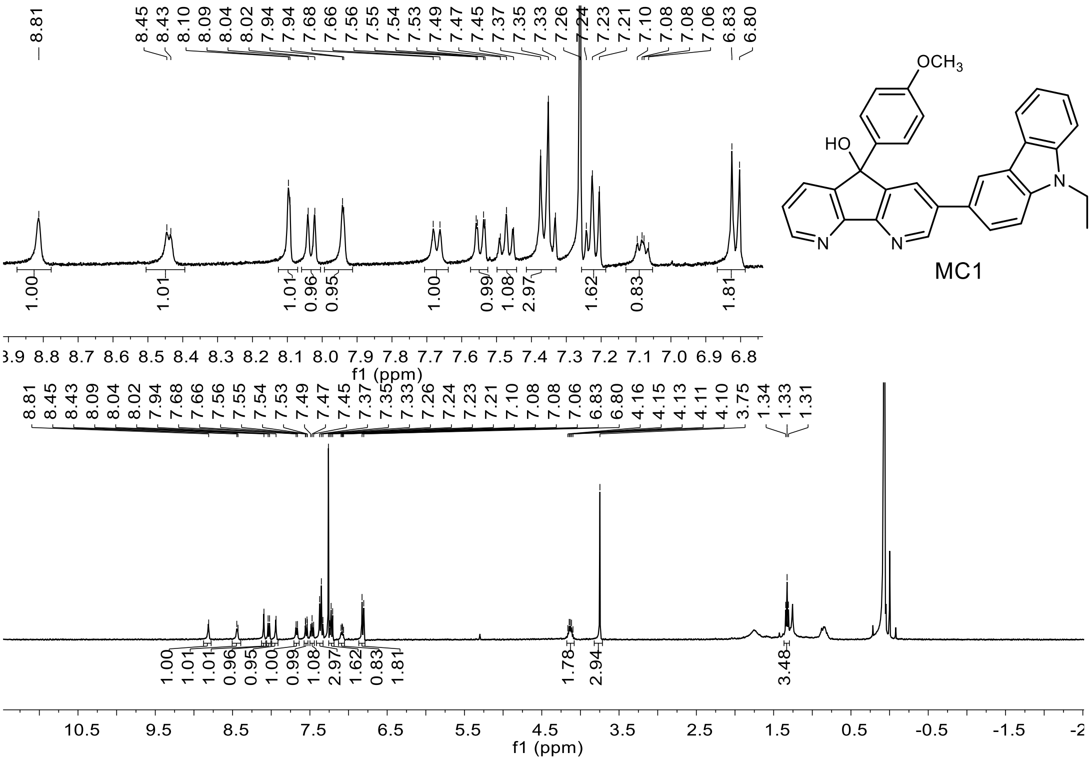

Supplementary Figure 53. ^{1}H NMR spectra for **MC1** in $CDCl_3$

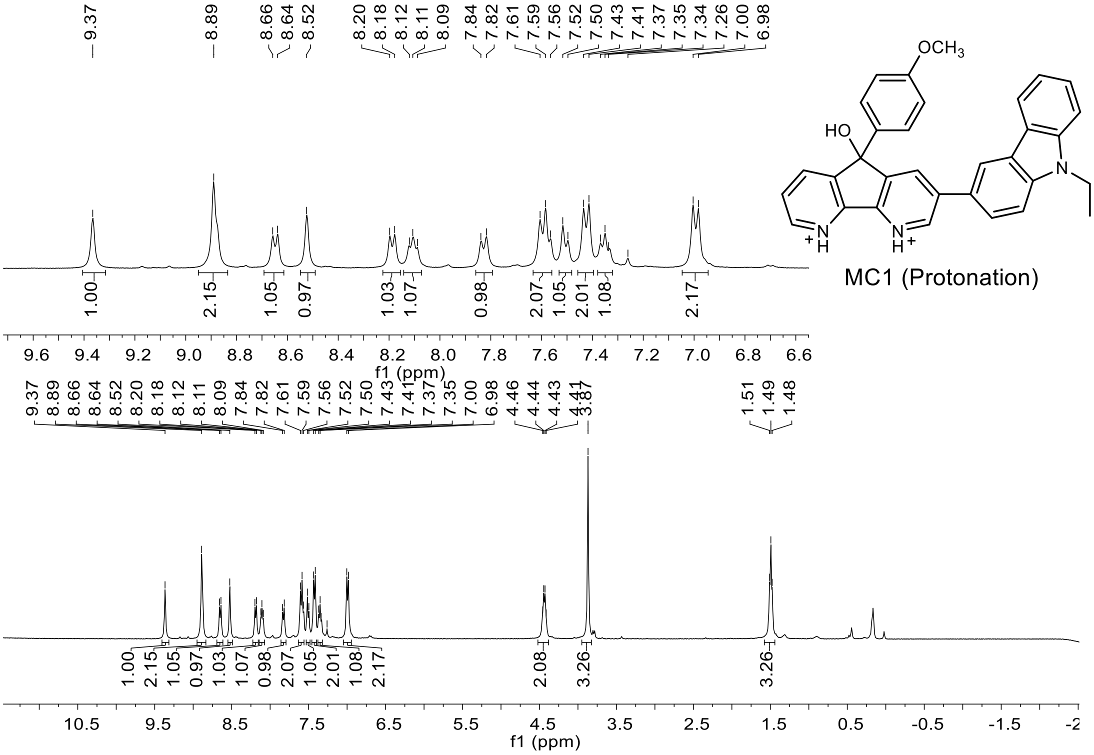

Supplementary Figure 54. ^{1}H NMR spectra for protonated **MC1** in $CDCl_3$ and CF_3COOH mixed solvents. The signal of CF_3COOH is located at 12.0 ppm.

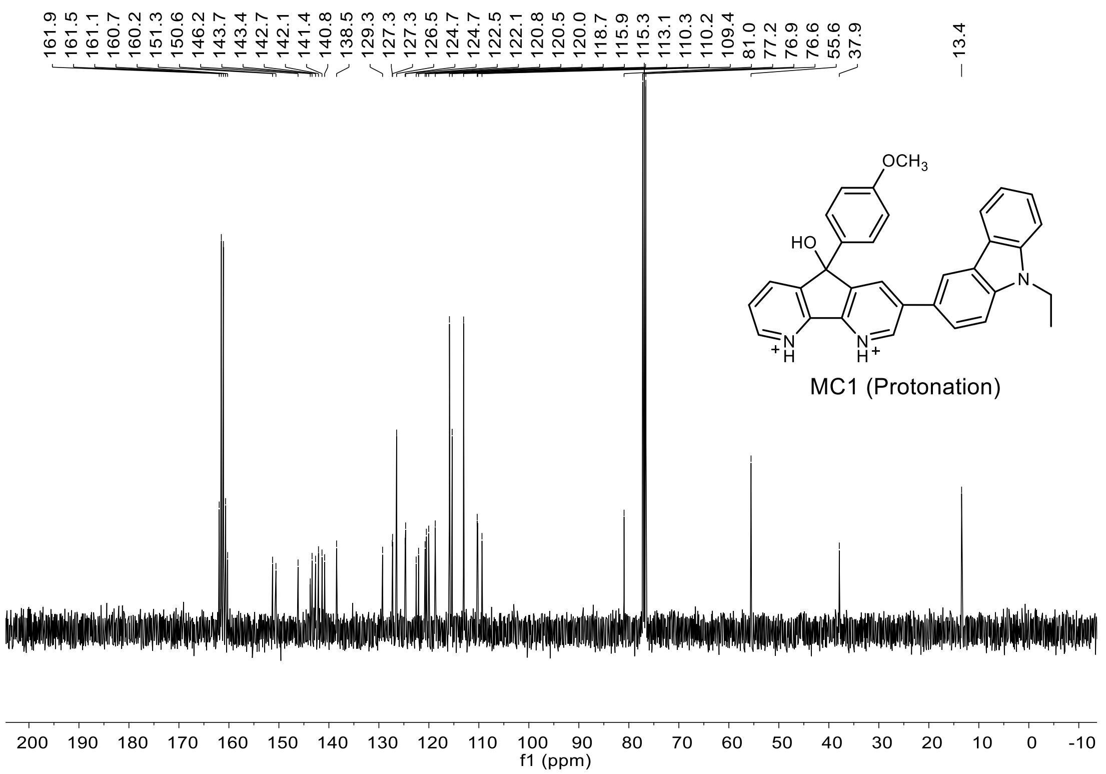

Supplementary Figure 55. ^{13}C NMR spectra for protonated **MC1** in $CDCl_3$ and CF_3COOH mixed solvents. The signals at 161.9, 161.5, 161.1, 160.7, 118.7, 115.9, 113.1 and 110.3 ppm are assigned to additional CF_3COOH.

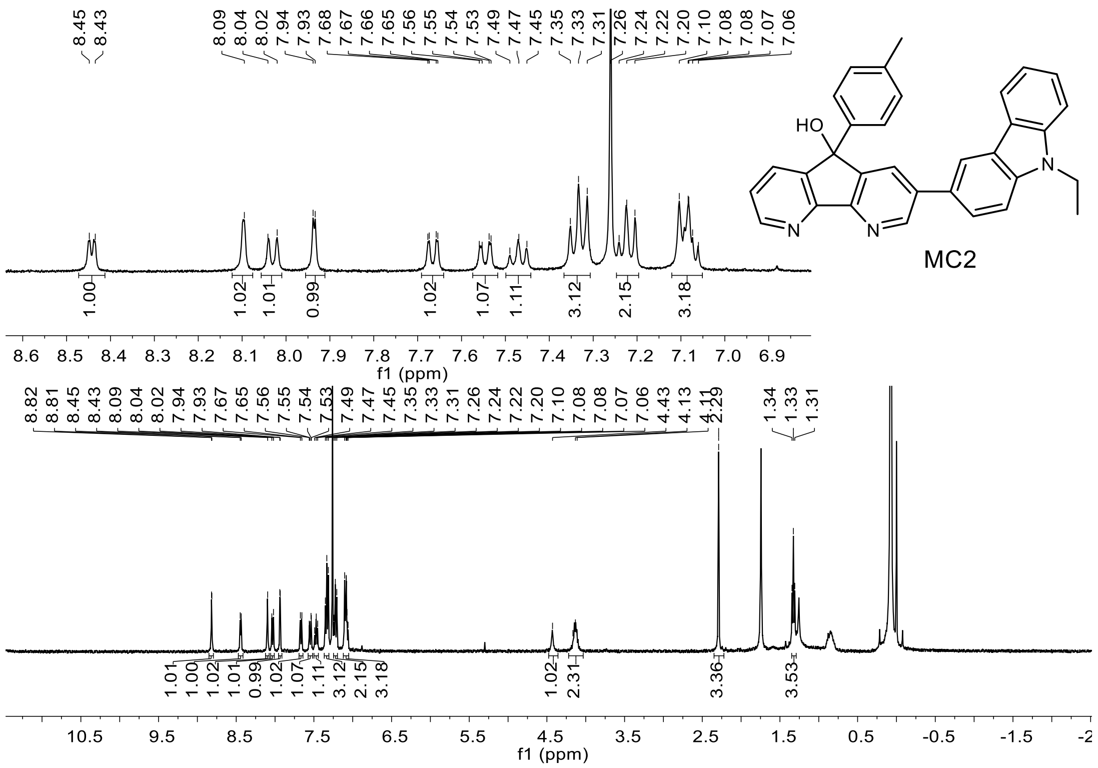

Supplementary Figure 56. ^{1}H NMR spectra for **MC2** in $CDCl_3$

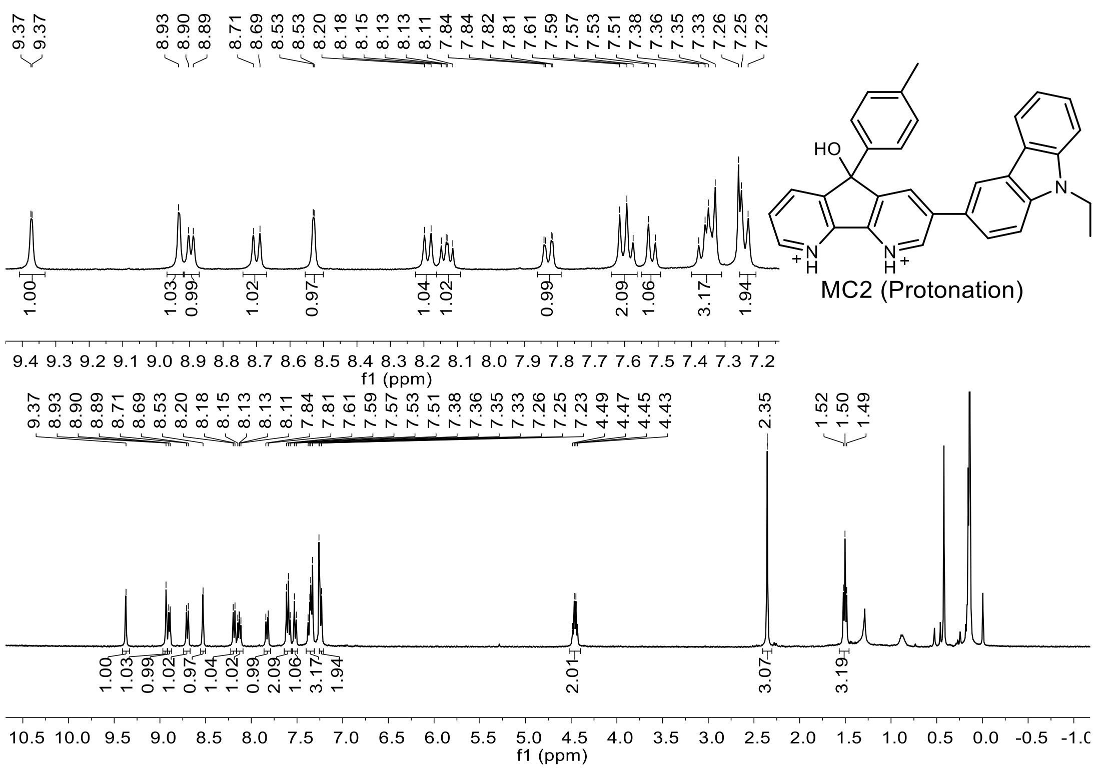

Supplementary Figure 57. ^{1}H NMR spectra for protonated **MC2** in $CDCl_3$ and CF_3COOH mixed solvents. The signal of CF_3COOH is located at 11.8 ppm.

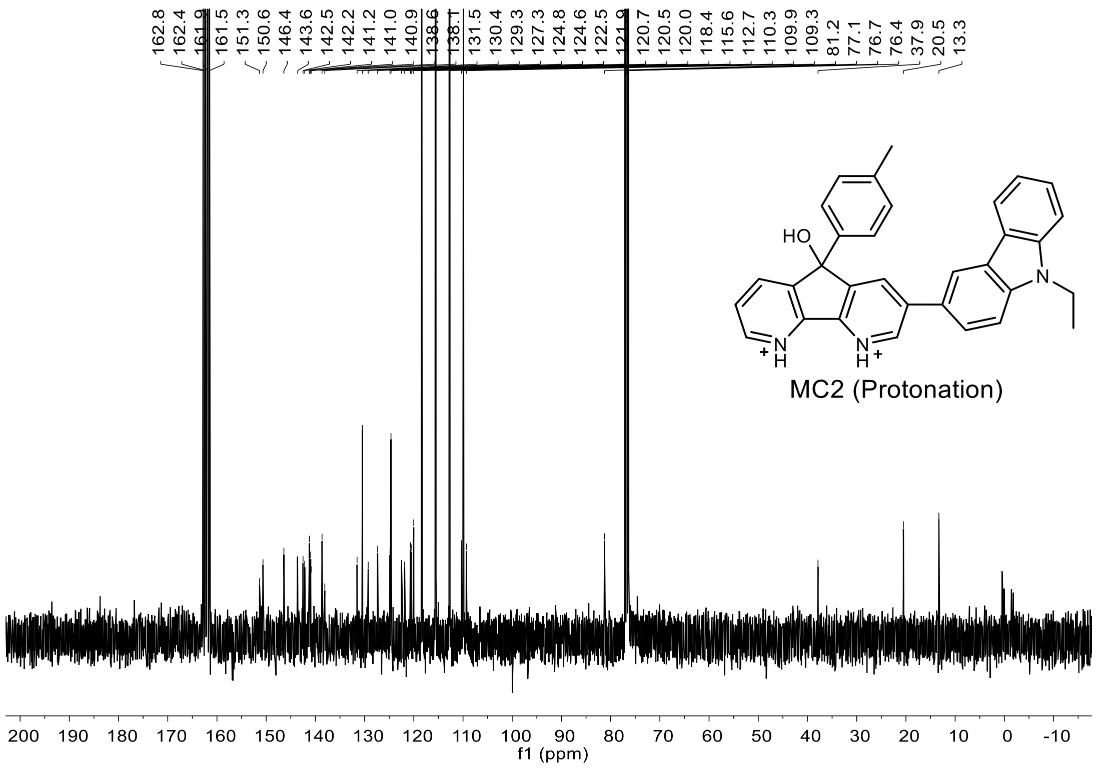

Supplementary Figure 58. ^{13}C NMR spectra for protonated **MC2** in $CDCl_3$ and CF_3COOH mixed solvents. The signals at 162.8, 162.4, 161.9, 161.5, 118.4, 115.6, 112.7 and 109.9 ppm are assigned to additional CF_3COOH.

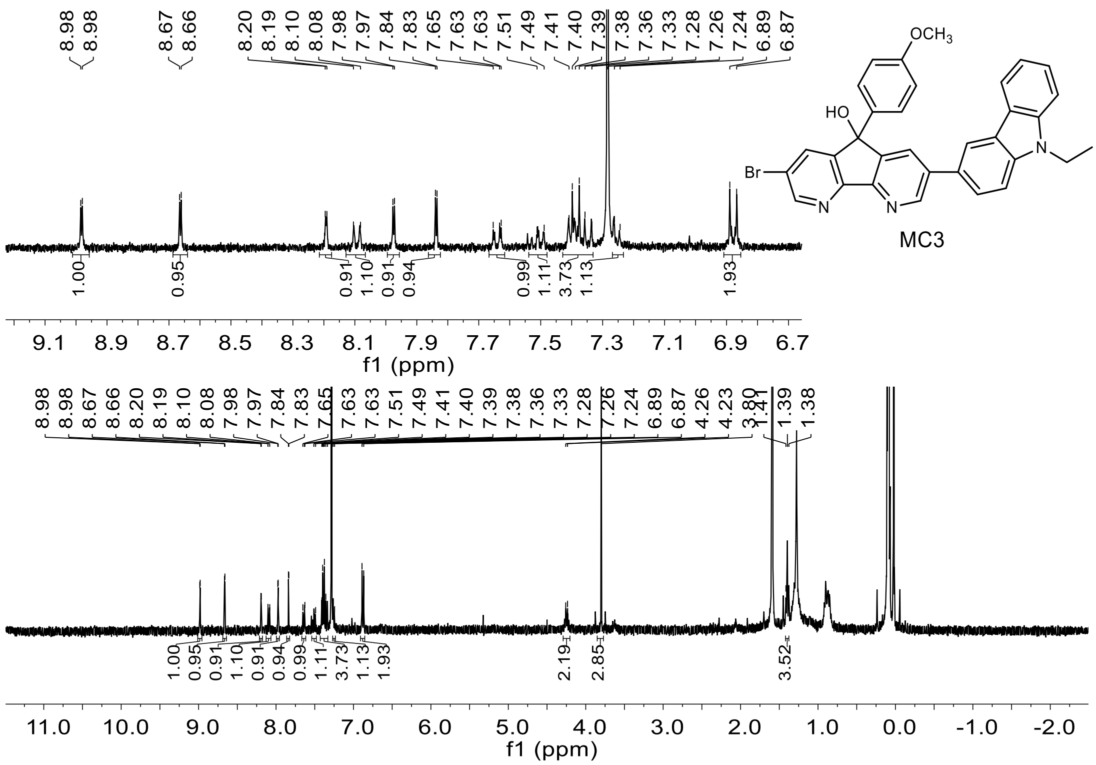

Supplementary Figure 59. ^{1}H NMR spectra for **MC3** in $CDCl_3$

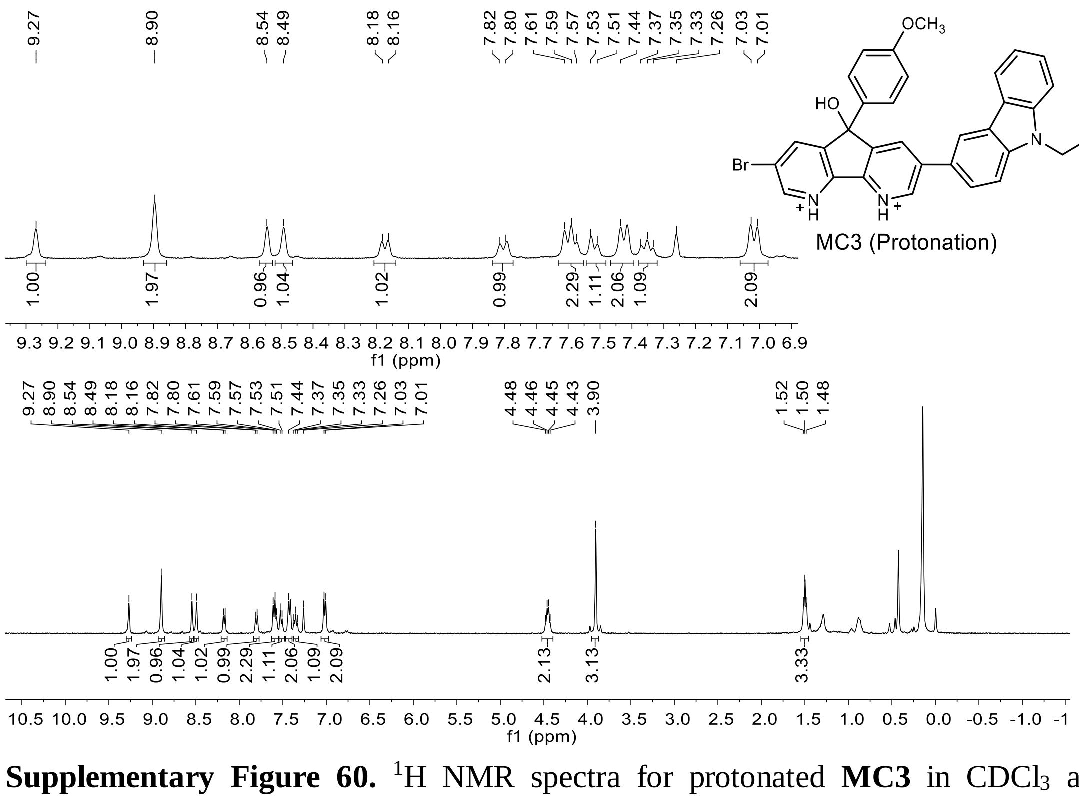

Supplementary Figure 60. ^{1}H NMR spectra for protonated **MC3** in $CDCl_3$ and CF_3COOH mixed solvents. The signal of CF_3COOH is located at 11.8 ppm.

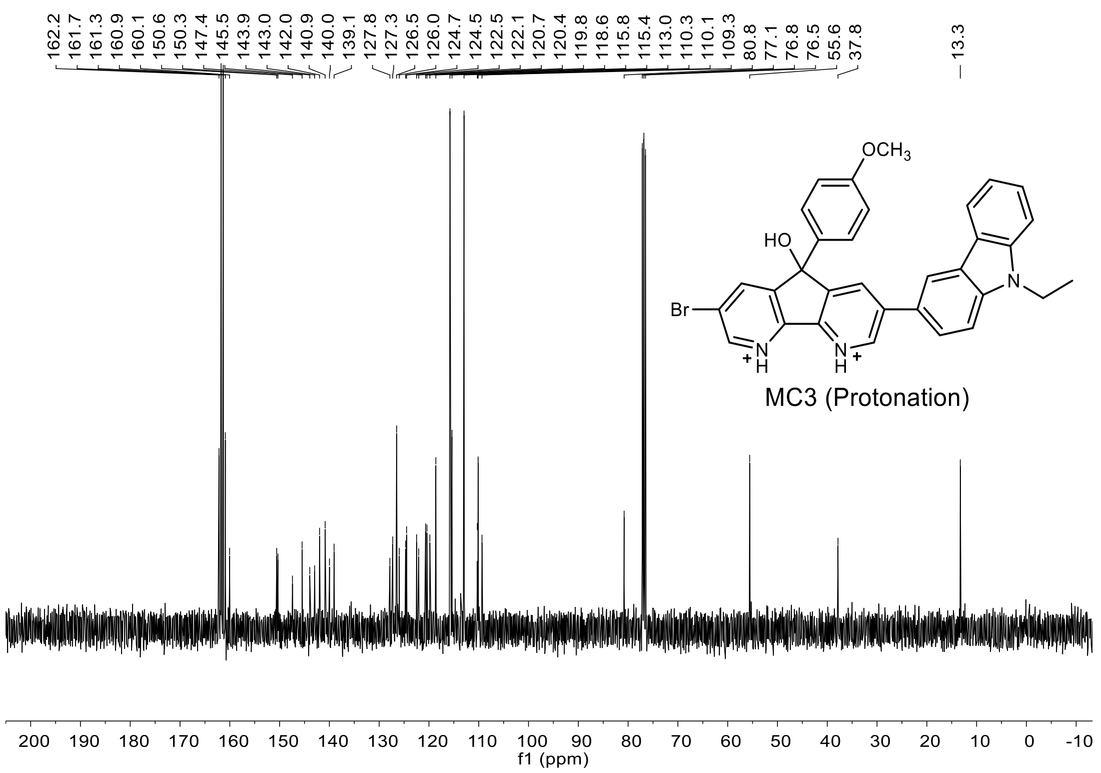

Supplementary Figure 61. ^{13}C NMR spectra for protonated **MC3** in $CDCl_3$ and CF_3COOH mixed solvents. The signals at 162.2, 161.7, 161.3, 160.9, 118.6, 115.8, 113.0 and 110.1 ppm are assigned to additional CF_3COOH.

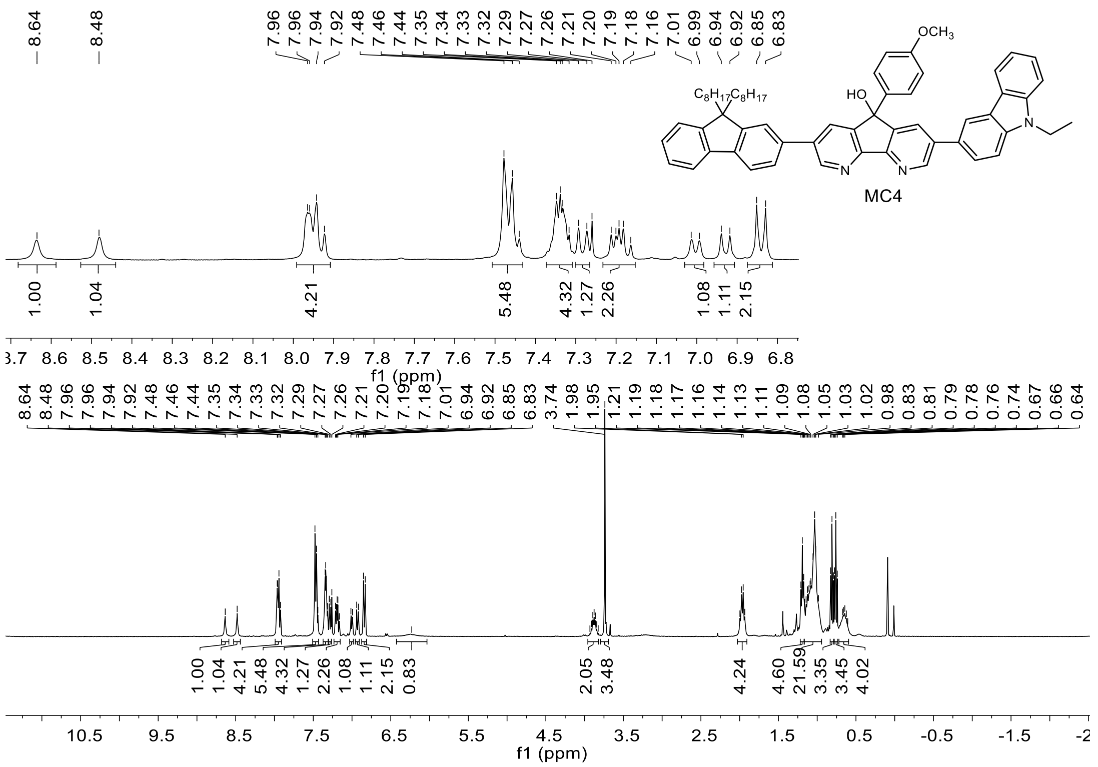

Supplementary Figure 62. ^{1}H NMR spectra for **MC4** in $CDCl_3$

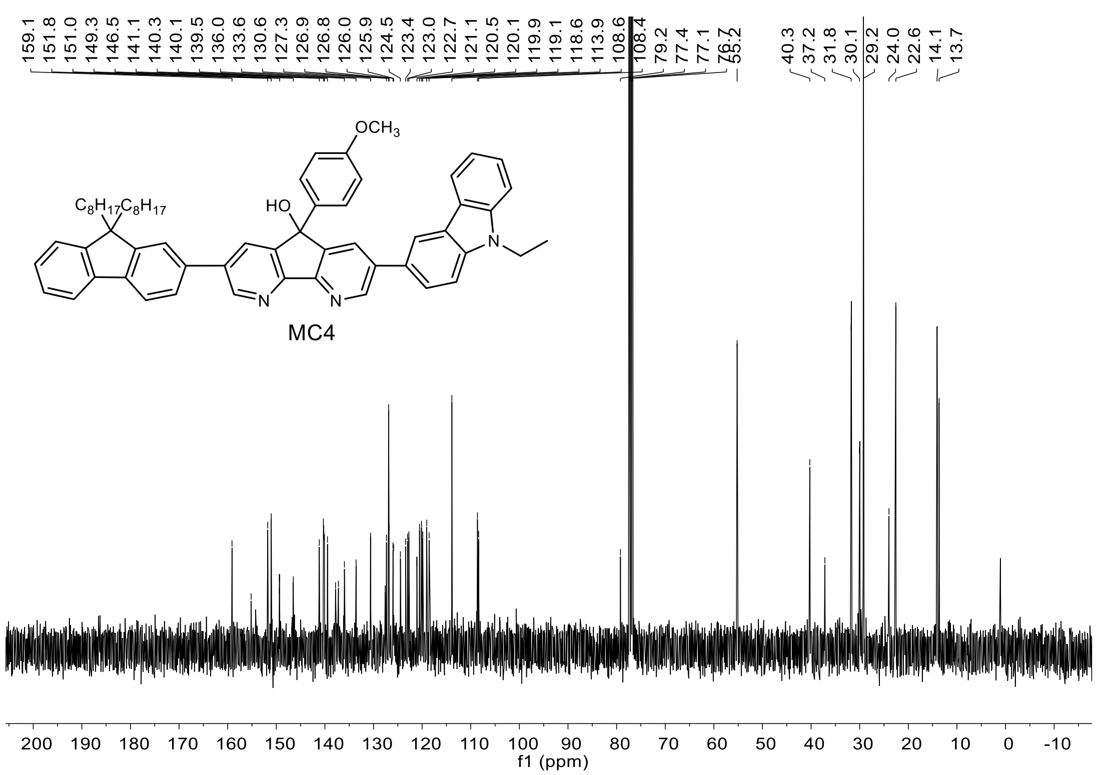

Supplementary Figure 63. ^{13}C NMR spectra for **MC4** in $CDCl_3$

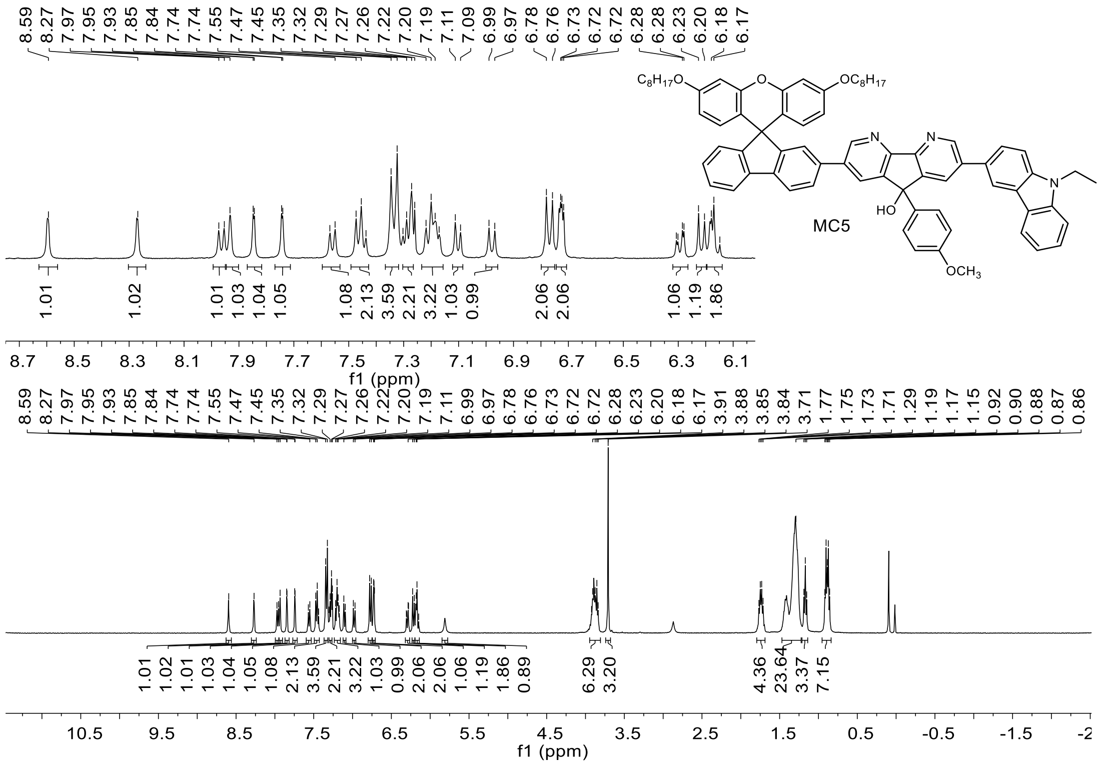

Supplementary Figure 64. ^{1}H NMR spectra for **MC5** in $CDCl_3$

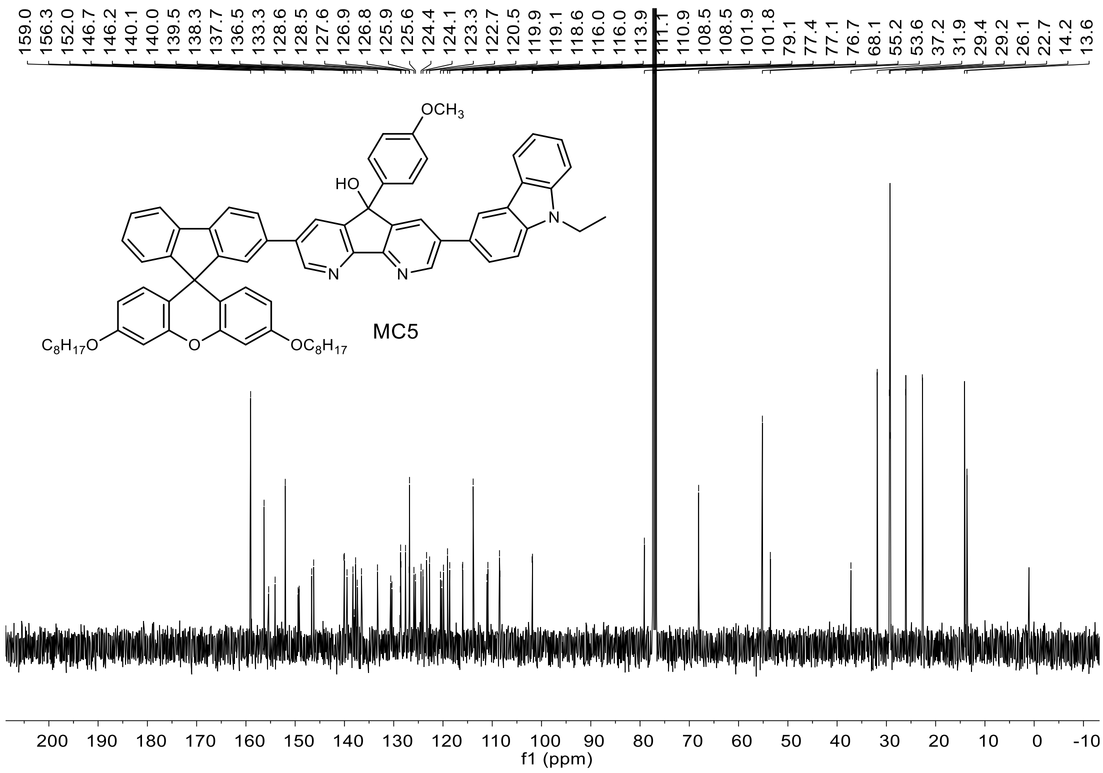

Supplementary Figure 65. ^{13}C NMR spectra for **MC5** in $CDCl_3$

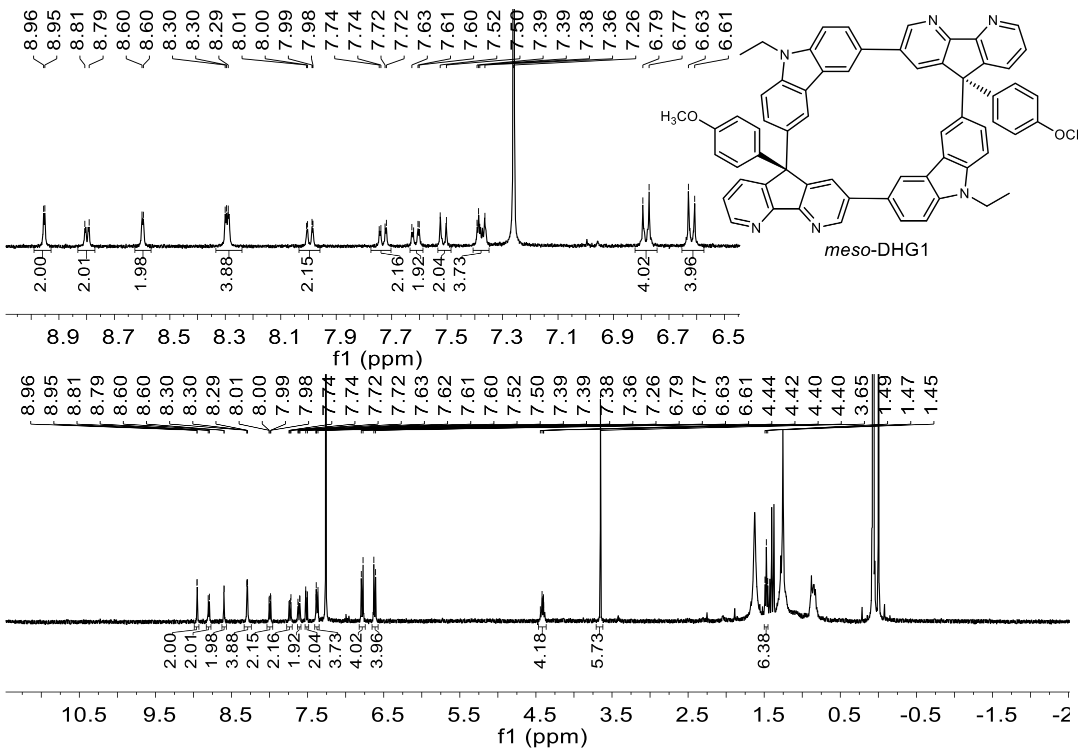

Supplementary Figure 66. ^{1}H NMR spectra for ***meso*-DHG1** in $CDCl_3$

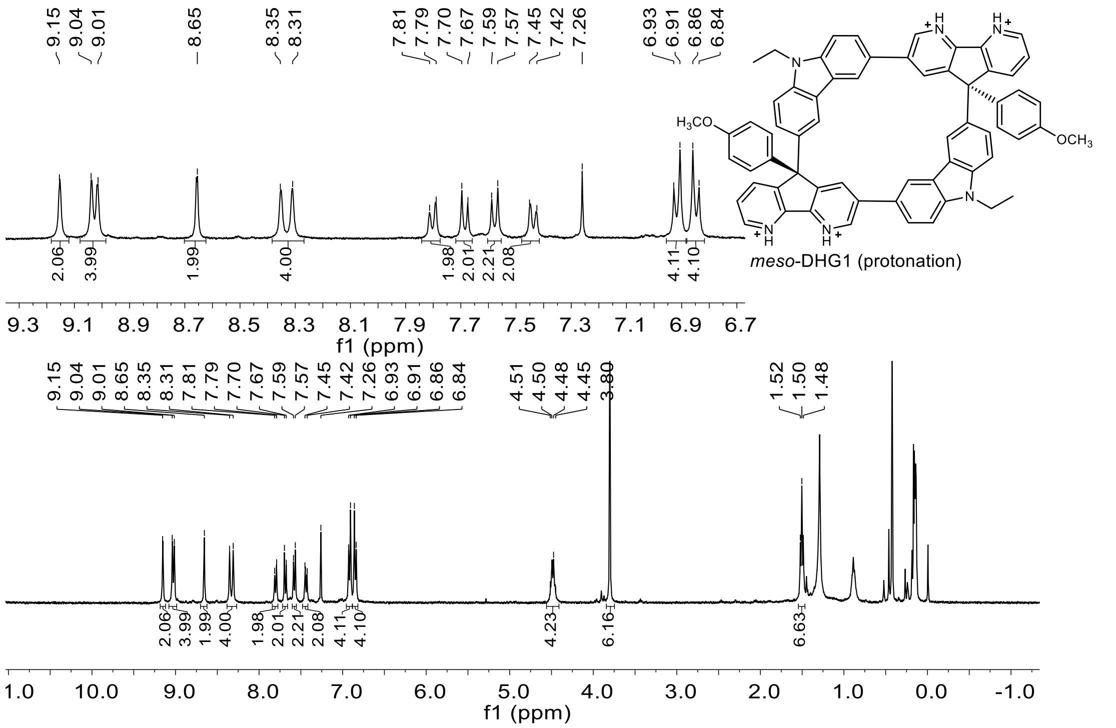

Supplementary Figure 67. ^{1}H NMR spectra for protonated ***meso*-DHG1** in $CDCl_3$ and CF_3COOH mixed solvents. The signal of CF_3COOH is located at 11.8 ppm.

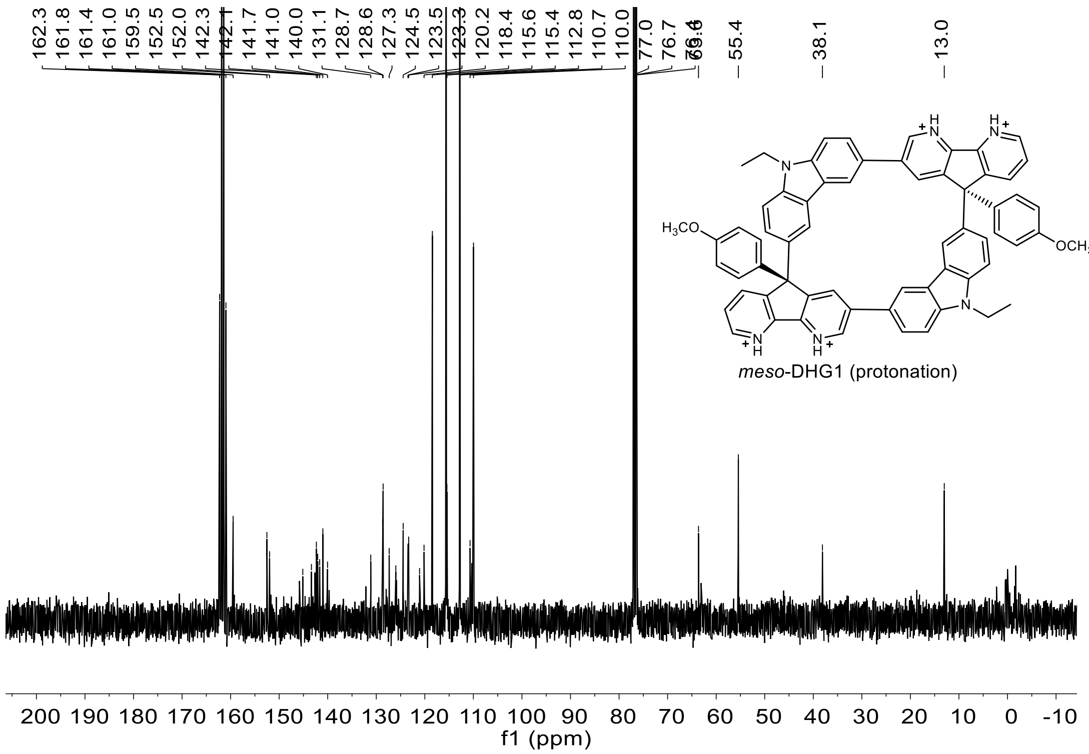

Supplementary Figure 68. ^{13}C NMR spectra for protonated ***meso*-DHG1** in $CDCl_3$ and CF_3COOH mixed solvents. The signals at 162.2, 161.8, 161.4, 160.9, 118.8, 115.9, 113.1 and 110.2 ppm are assigned to additional CF_3COOH.

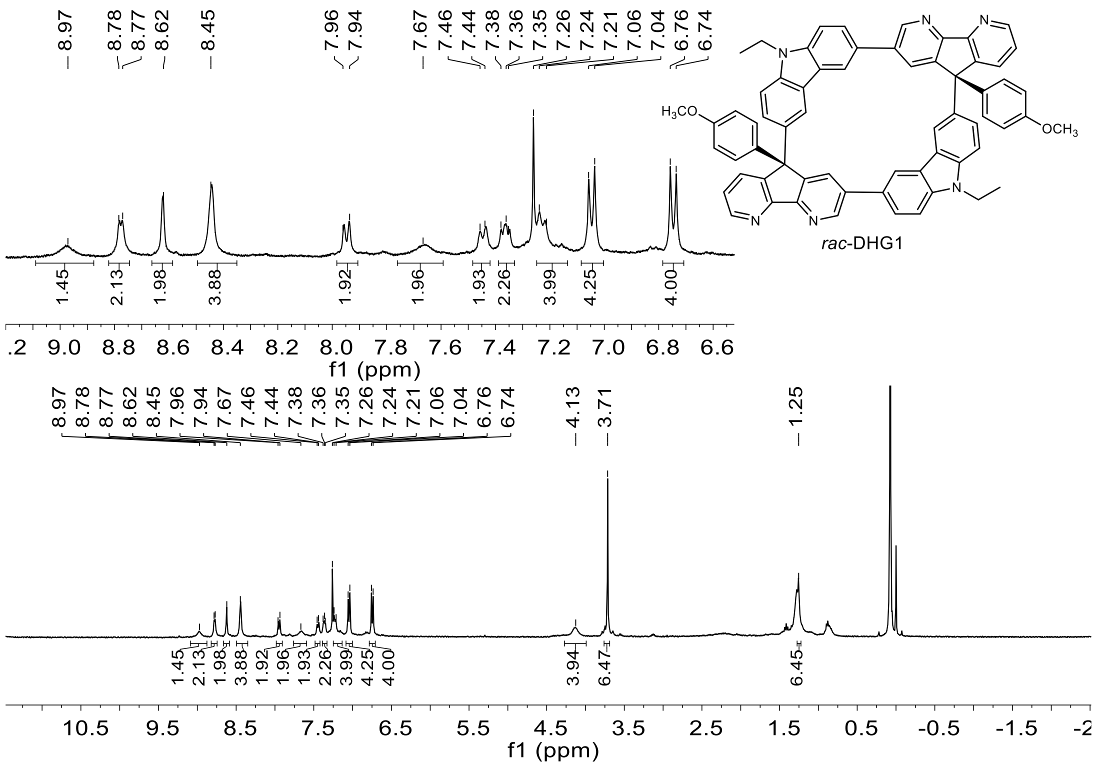

Supplementary Figure 69. ^{1}H NMR spectra for ***rac*-DHG1** in $CDCl_3$

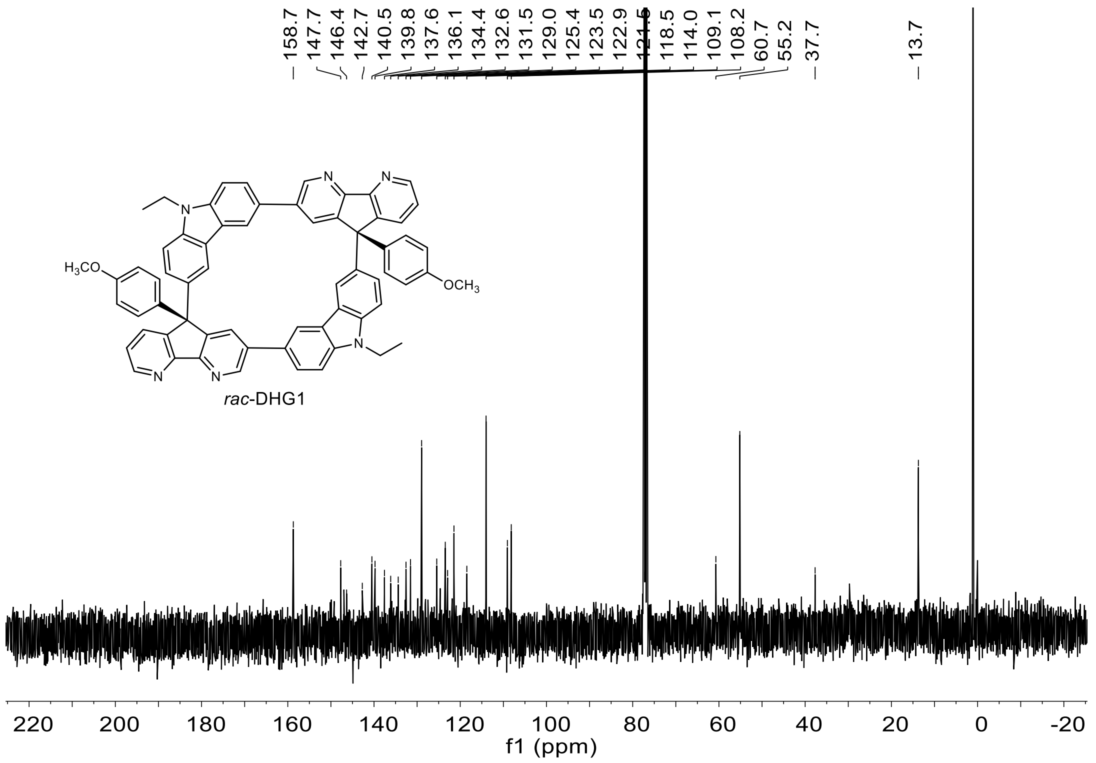

Supplementary Figure 70. ^{13}C NMR spectra for ***rac*-DHG1** in $CDCl_3$

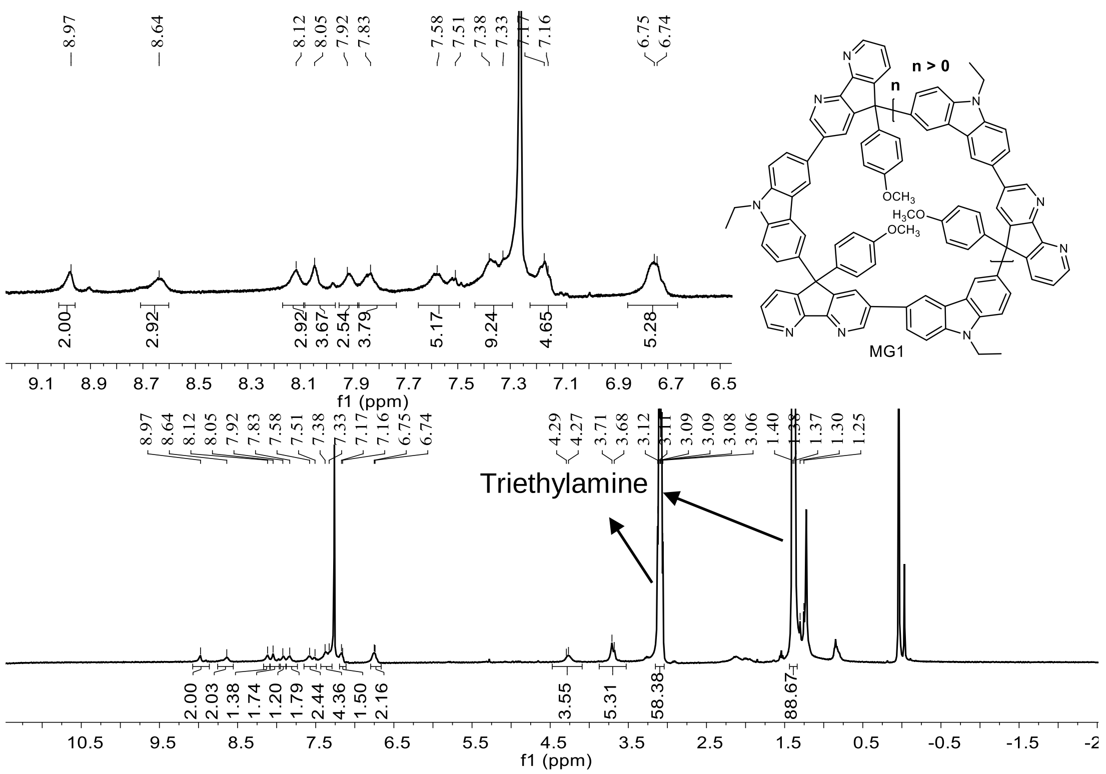

Supplementary Figure 71. ^{1}H NMR spectra for **MG1** in $CDCl_3$. In this spectra, the triethylamine (3.11 ppm and 1.38 ppm) were complexed and difficult to remove.

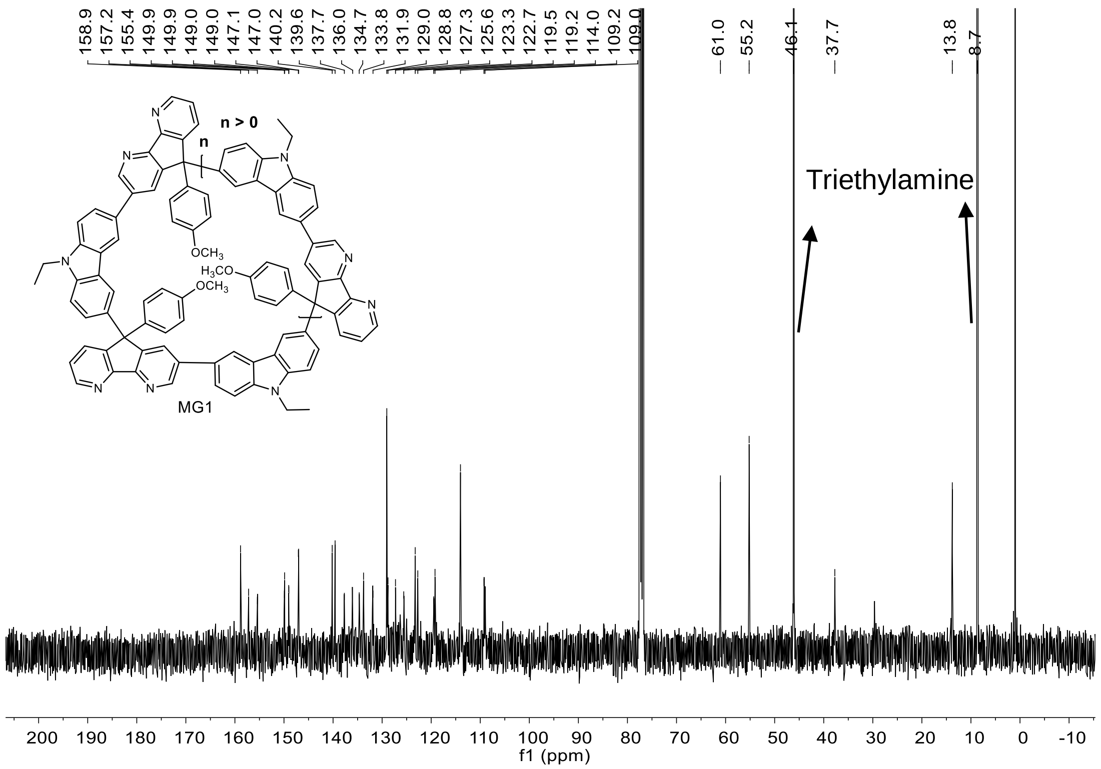

Supplementary Figure 72. ^{13}C NMR spectra for **MG1** in $CDCl_3$. In this spectra, the triethylamine (46.1 and 8.7 ppm) were complexed and difficult to remove.

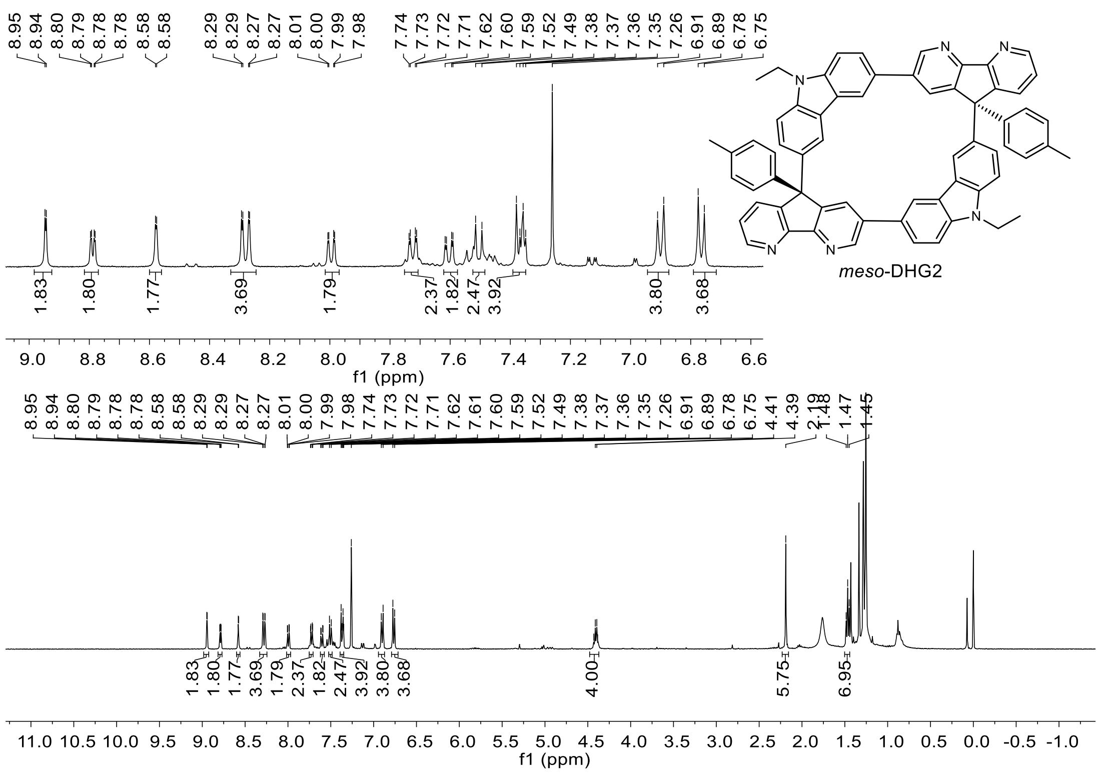

Supplementary Figure 73. ^{1}H NMR spectra for ***meso*-DHG2** in $CDCl_3$

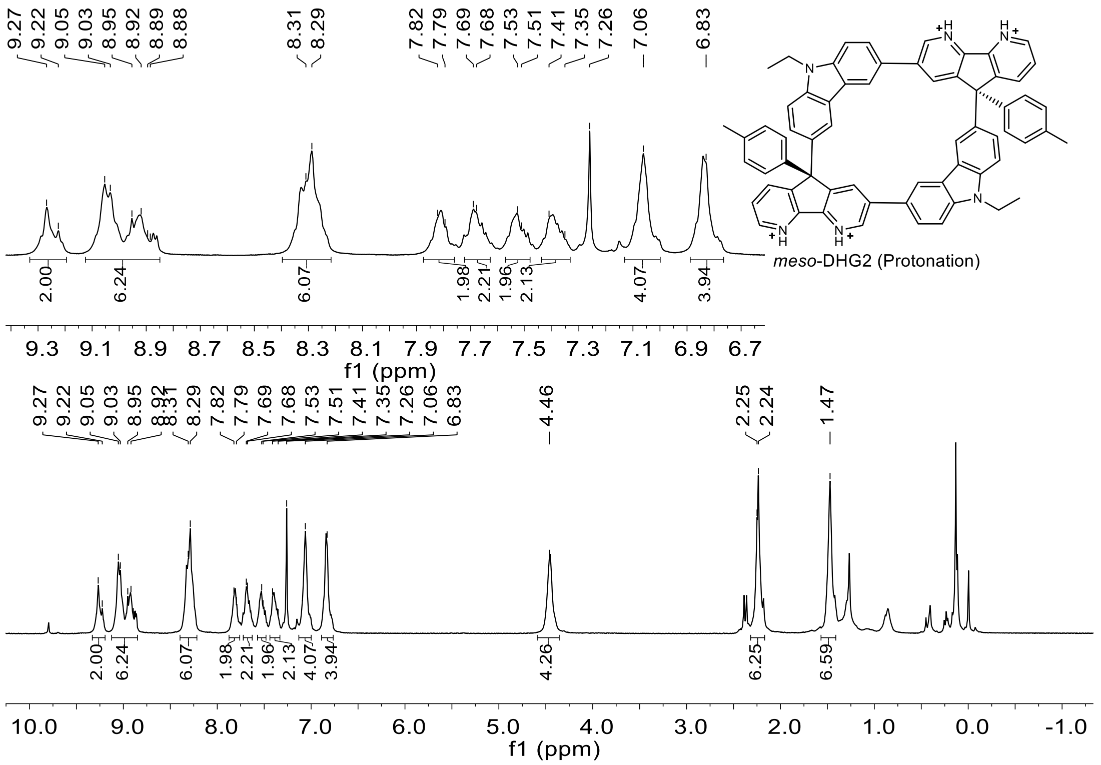

Supplementary Figure 74. ^{1}H NMR spectra for protonated ***meso*-DHG2** in $CDCl_3$ and CF_3COOH mixed solvents. The signal of CF_3COOH is located at 11.8 ppm.

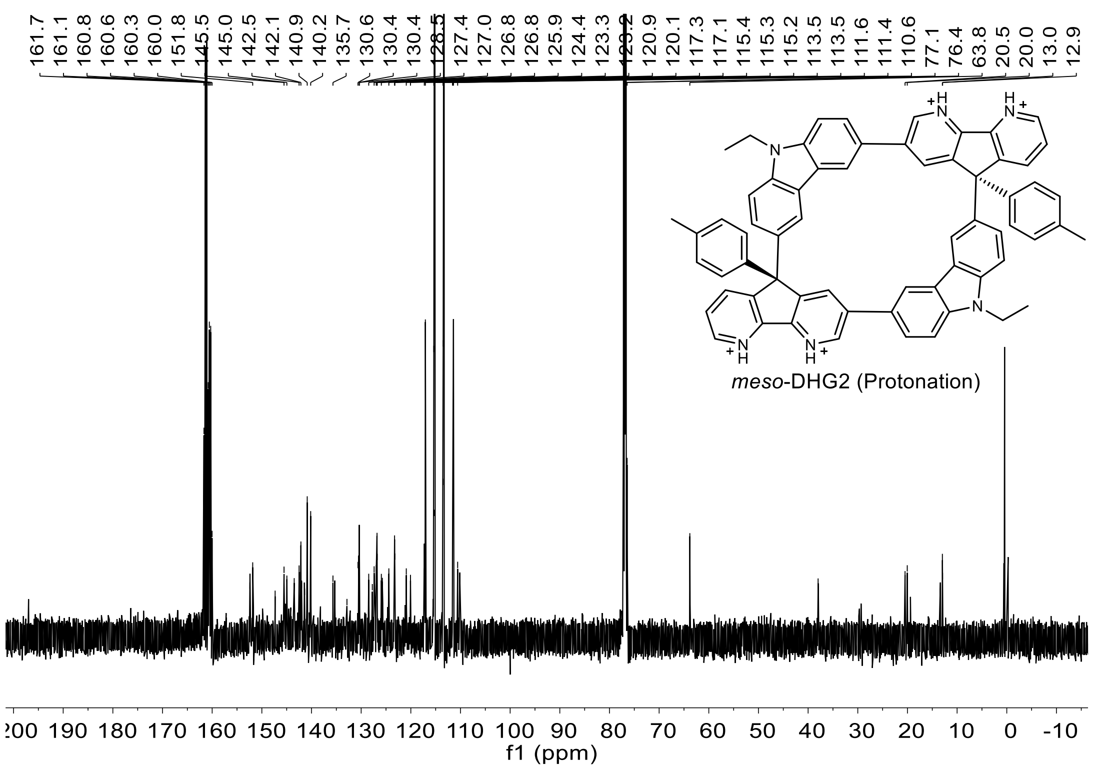

Supplementary Figure 75. ^{13}C NMR spectra for protonated ***meso*-DHG2** in $CDCl_3$ and CF_3COOH mixed solvents. The signals at 161.4, 161.0, 160.5, 160.1, 118.7, 115.8, 113.0, 110.2 ppm are assigned to additional CF_3COOH.

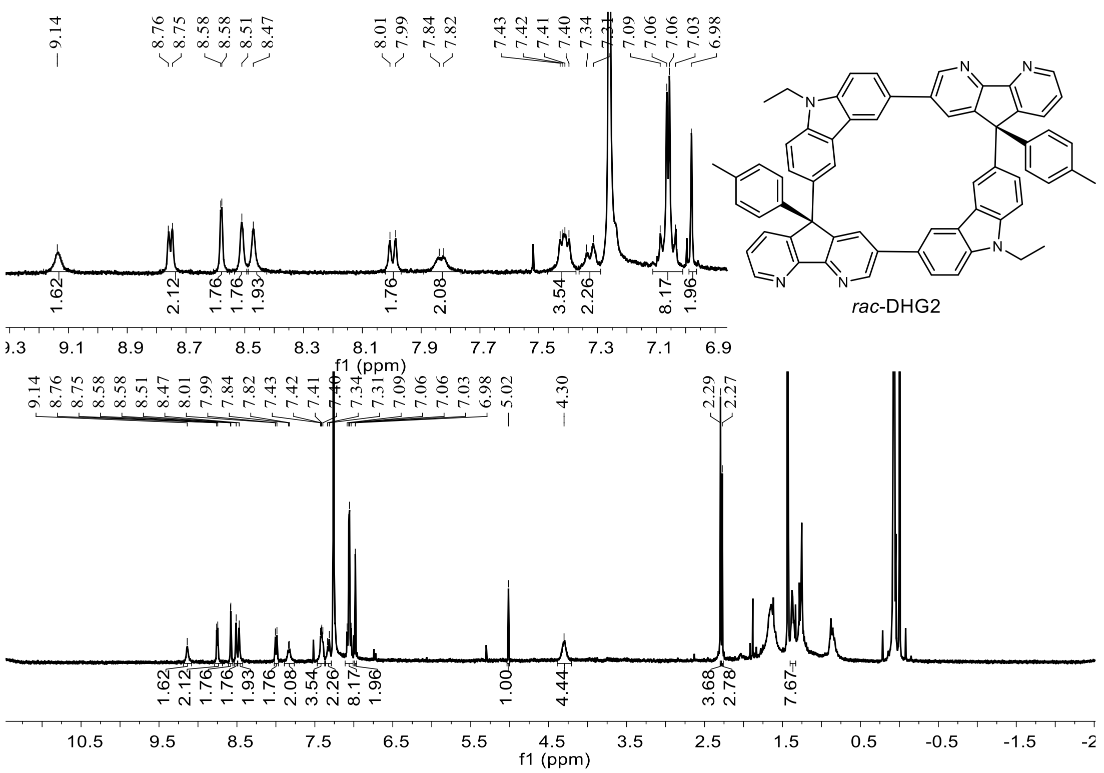

Supplementary Figure 76. ^{1}H NMR spectra for ***rac*-DHG2** in $CDCl_3$. In this spectra, the DCM and triethylamine were complexed and difficult to remove.

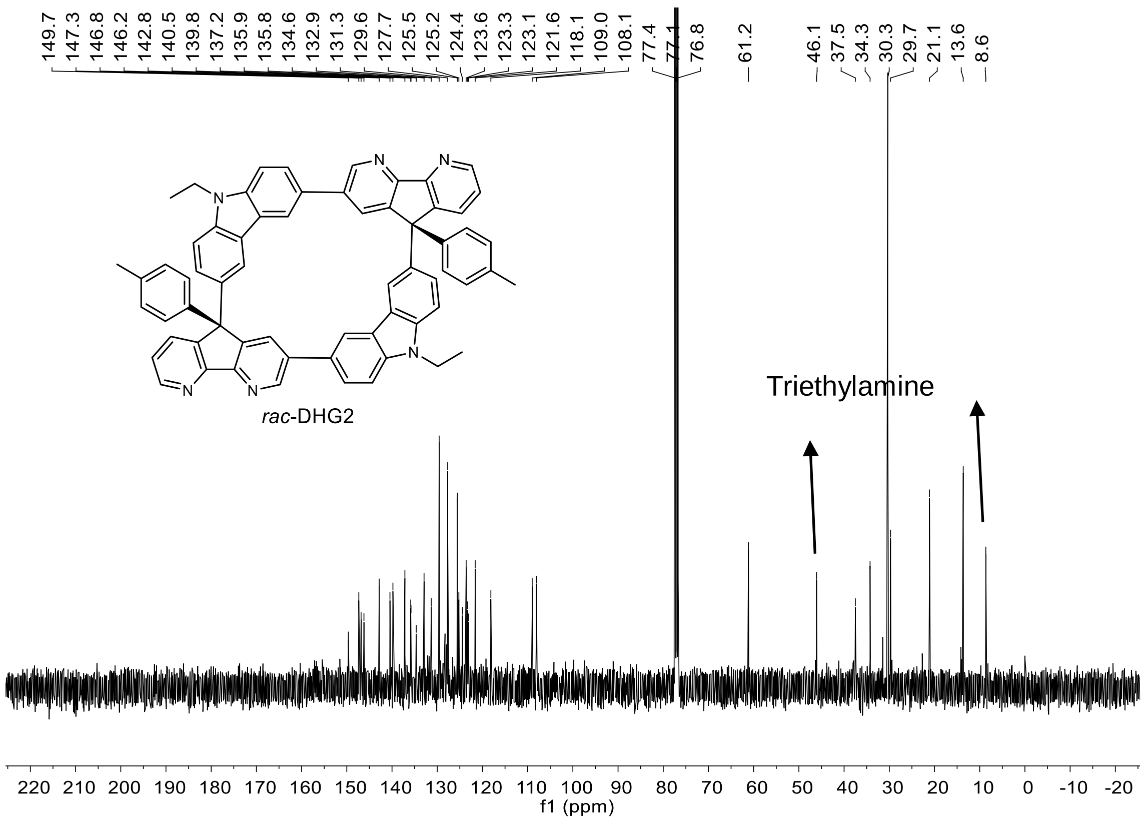

Supplementary Figure 77. ^{13}C NMR spectra for ***rac*-DHG2** in $CDCl_3$. In this spectra, the triethylamine (46.1 and 8.6 ppm) were complexed and difficult to remove.

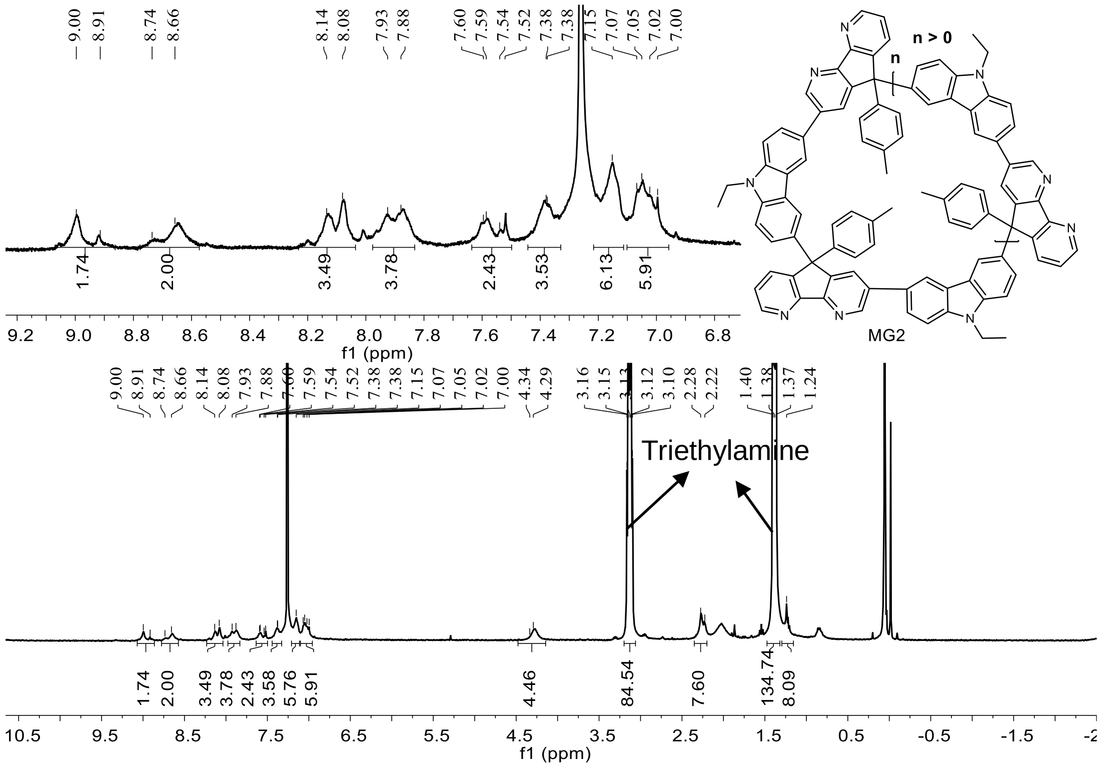

Supplementary Figure 78. ^{1}H NMR spectra for **MG2** in $CDCl_3$. In this spectra, the triethylamine (3.11 ppm and 1.38 ppm) were complexed and difficult to remove.

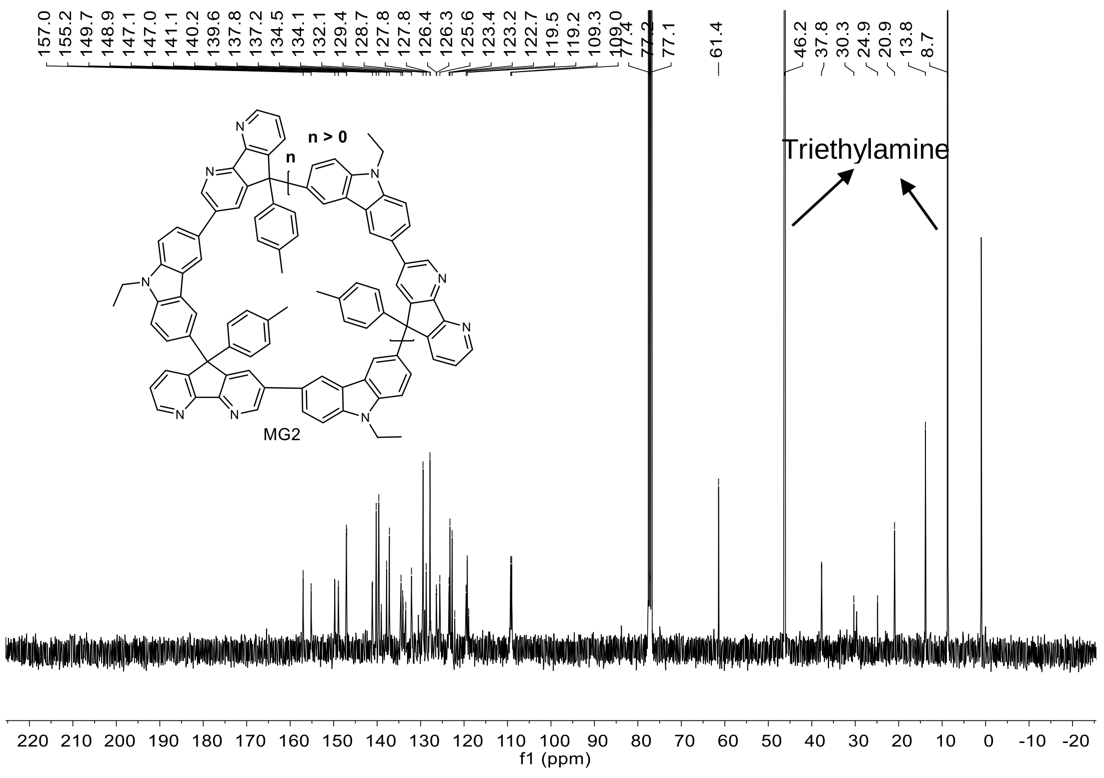

Supplementary Figure 79. ^{13}C NMR spectra for **MG2** in $CDCl_3$. In this spectra, the triethylamine (46.2 and 8.7 ppm) were complexed and difficult to remove.

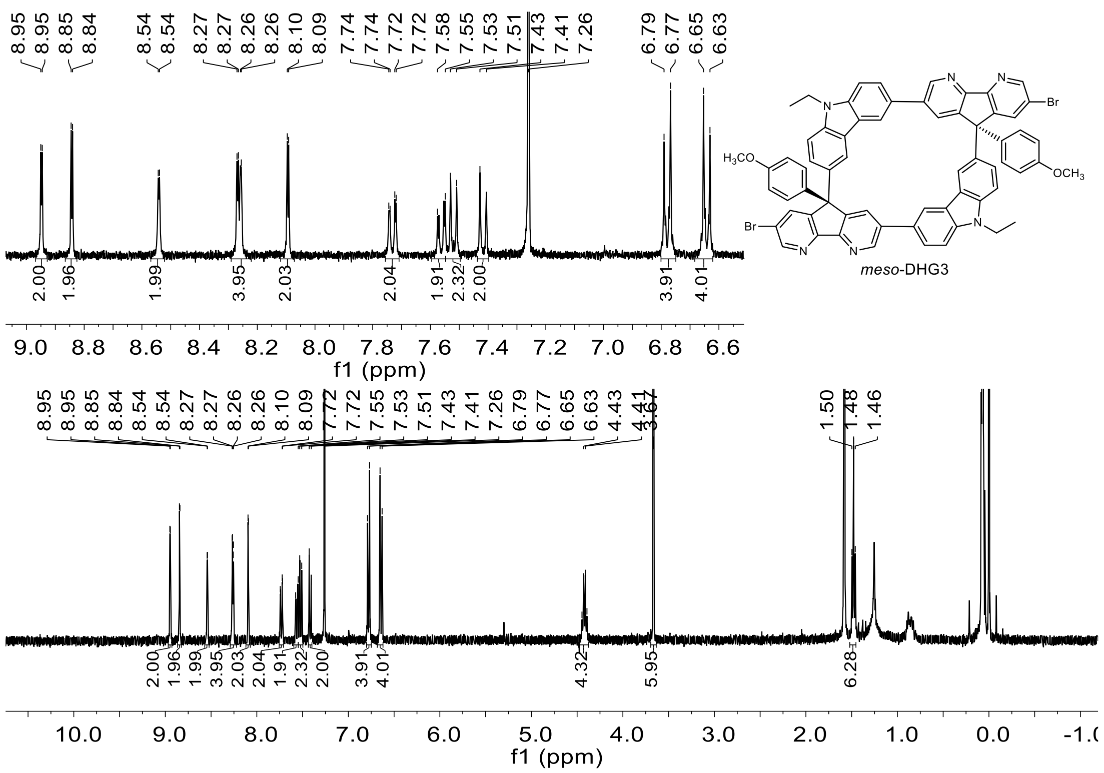

Supplementary Figure 80. ^{1}H NMR spectra for ***meso*-DHG3** in $CDCl_3$

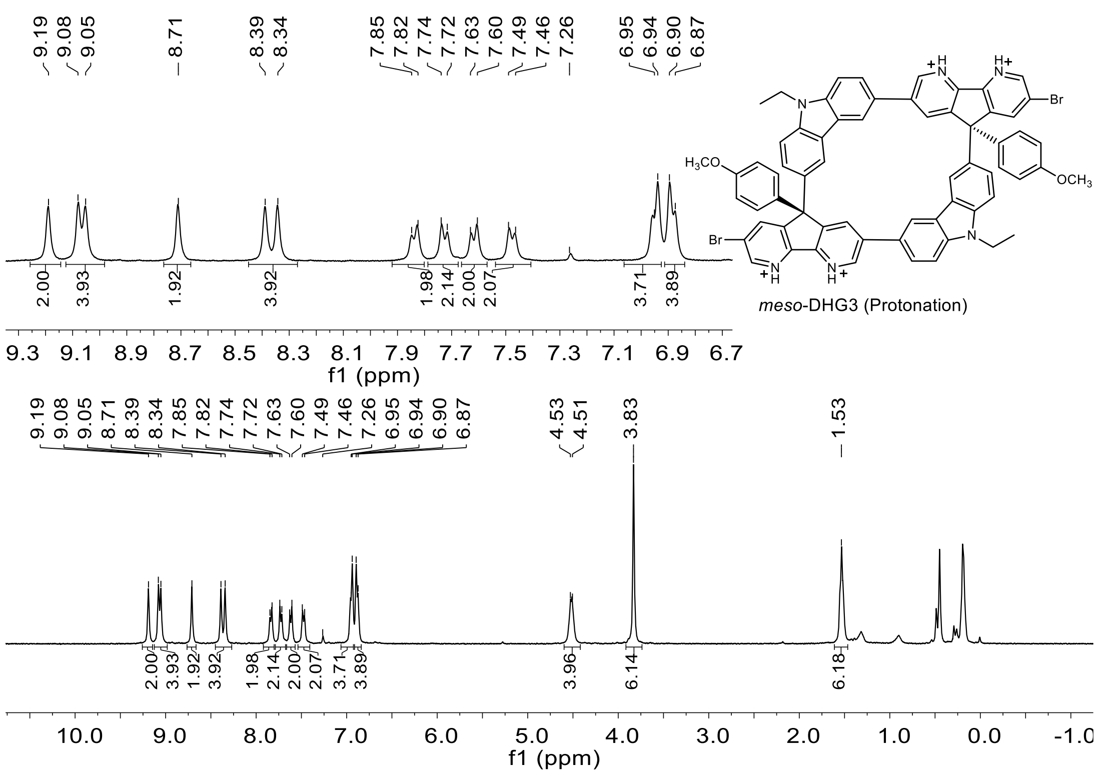

Supplementary Figure 81. ^{1}H NMR spectra for protonated ***meso*-DHG3** in $CDCl_3$ and CF_3COOH mixed solvents. The signal of CF_3COOH is located at 11.8 ppm.

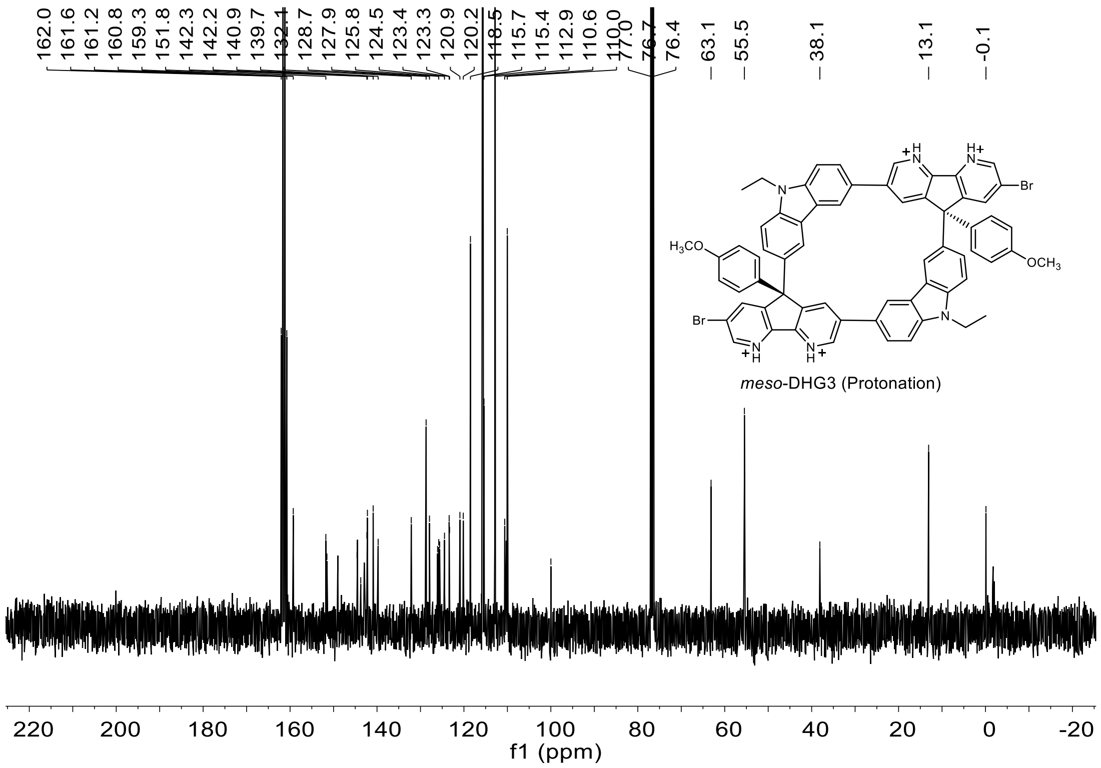

Supplementary Figure 82. ^{13}C NMR spectra for protonated ***meso*-DHG3** in $CDCl_3$ and CF_3COOH mixed solvents. The signals at 162.5, 162.0, 161.6, 161.2, 118.5, 115.7, 112.8 and 110.0 ppm are assigned to additional CF_3COOH.

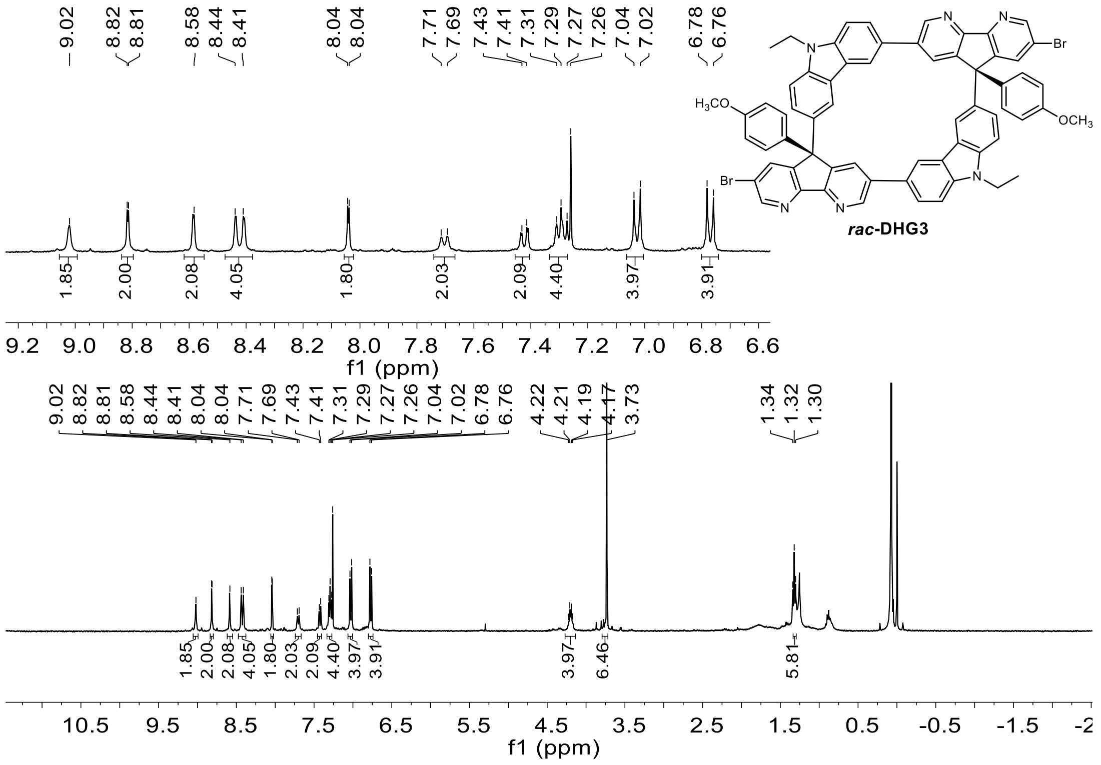

Supplementary Figure 83. ^{1}H NMR spectra for ***rac*-DHG3** in $CDCl_3$.

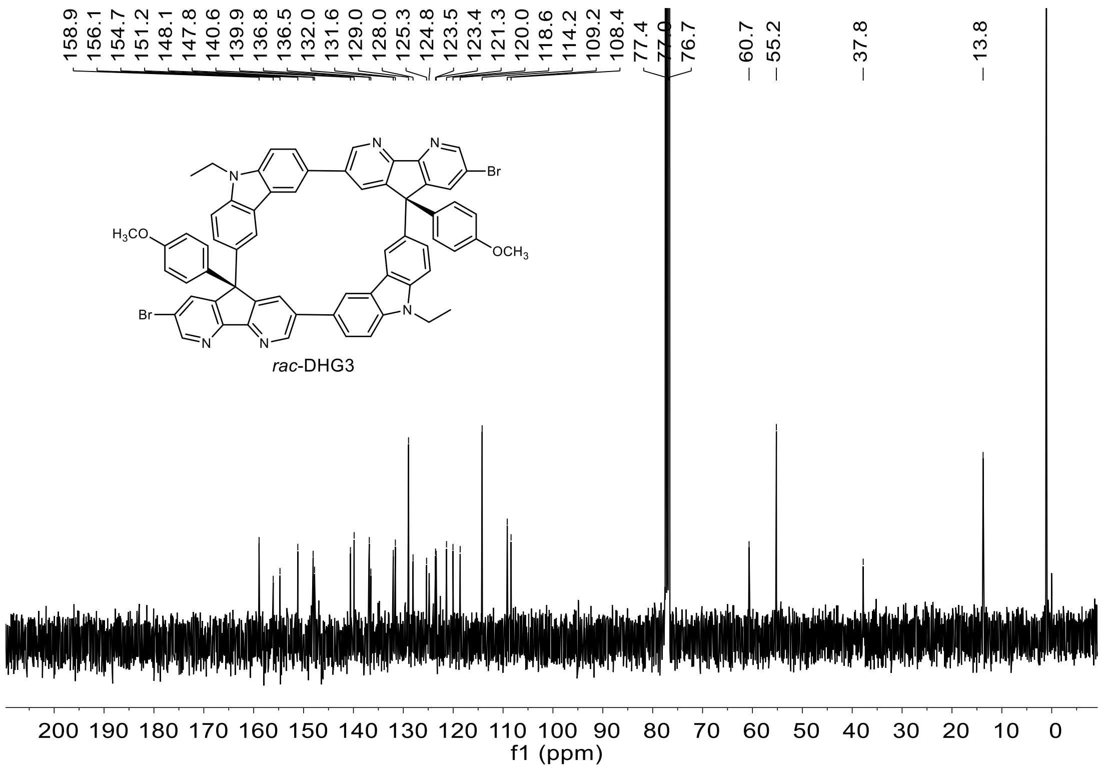

Supplementary Figure 84. ^{13}C NMR spectra for ***rac*-DHG3** in $CDCl_3$.

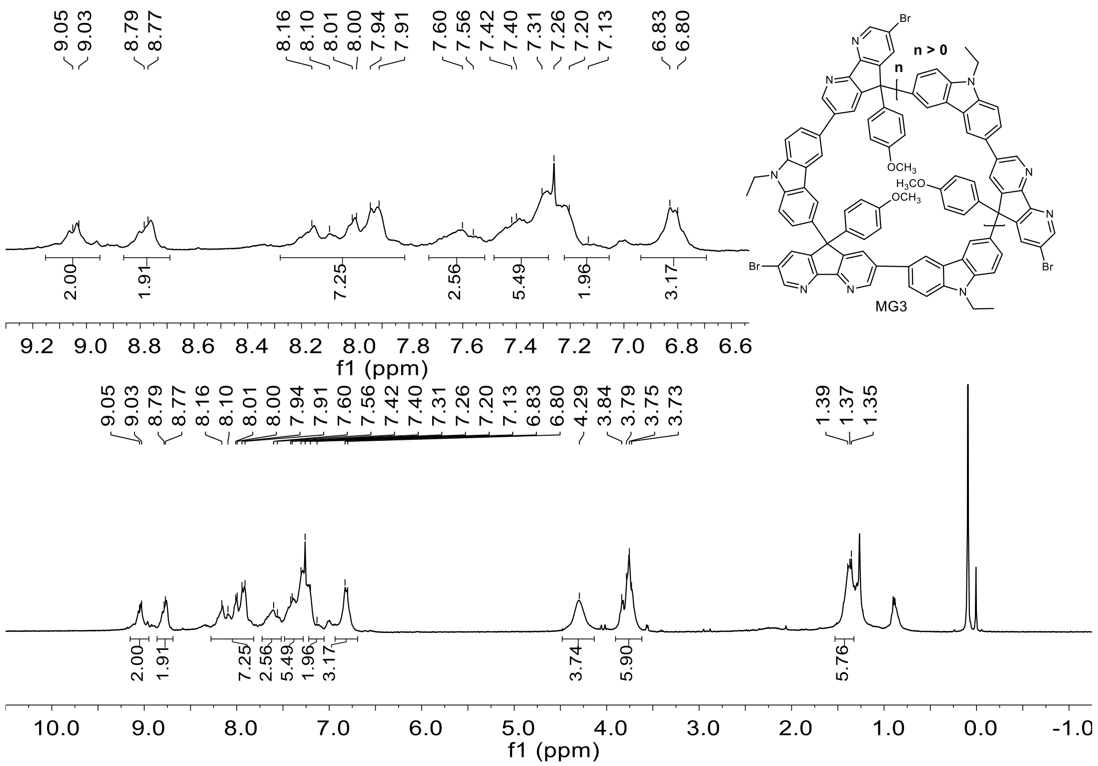

Supplementary Figure 85. ^{1}H NMR spectra for **MG3** in $CDCl_3$.

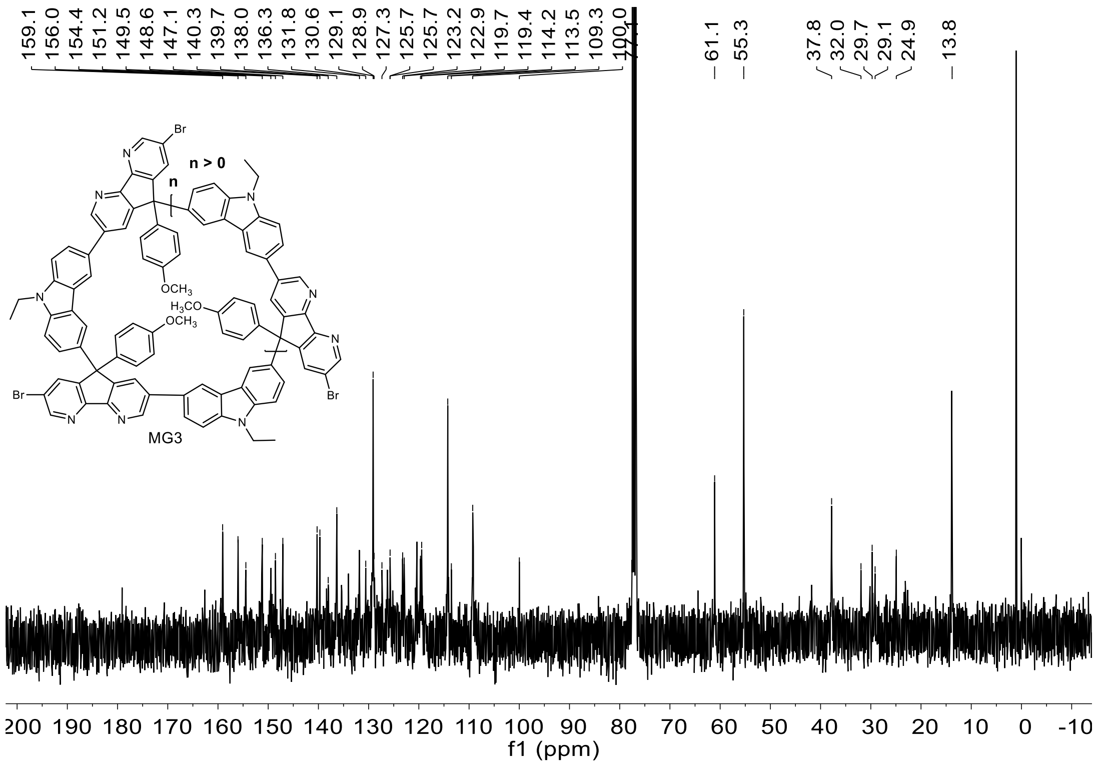

Supplementary Figure 86. ^{13}C NMR spectra for **MG3** in $CDCl_3$.

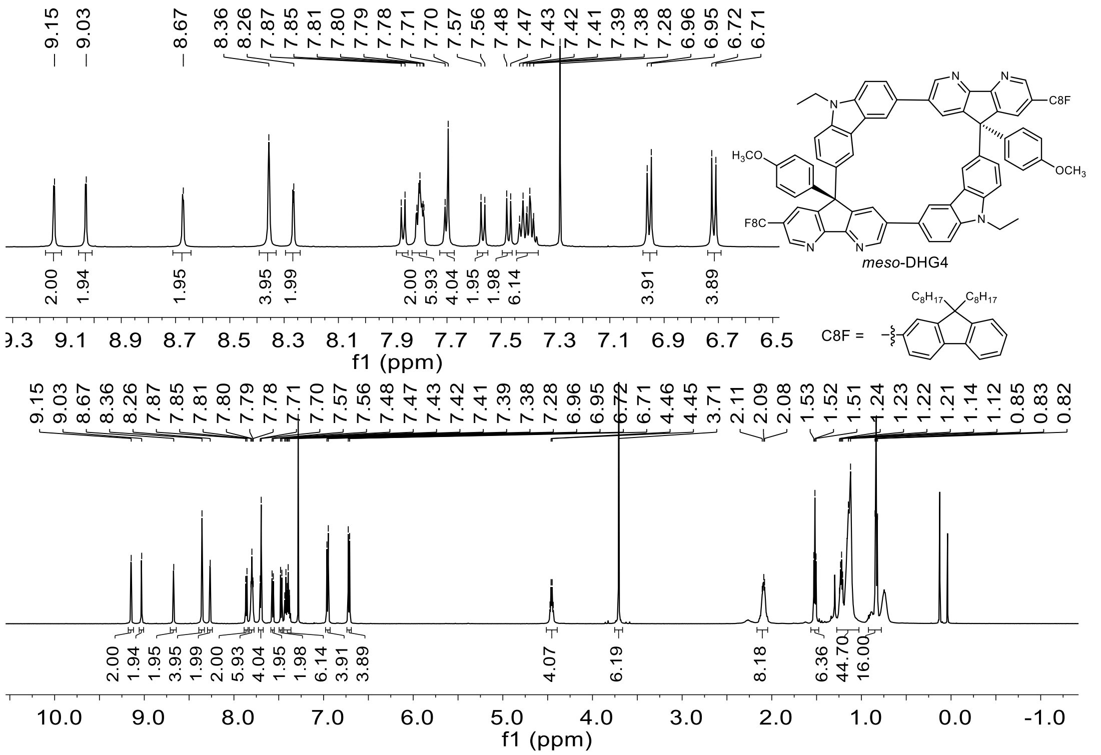

Supplementary Figure 87. ^{1}H NMR spectra for ***meso*-DHG4** in $CDCl_3$

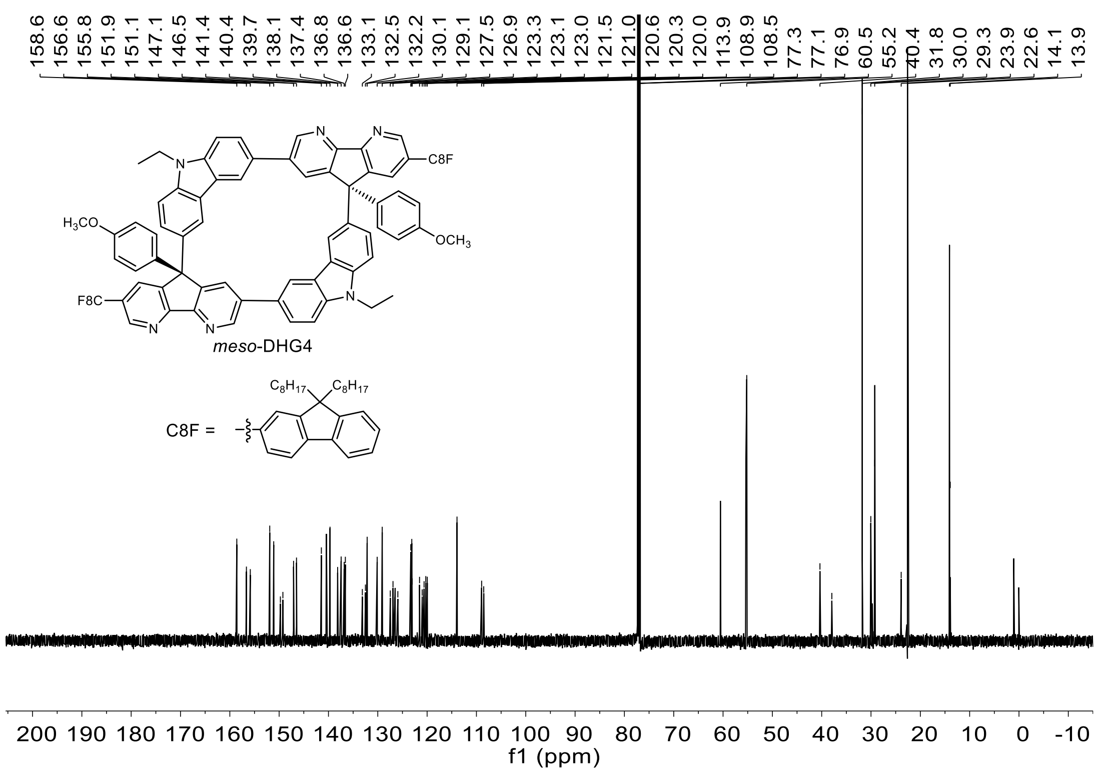

Supplementary Figure 88. ^{13}C NMR spectra for ***meso*-DHG4** in $CDCl_3$

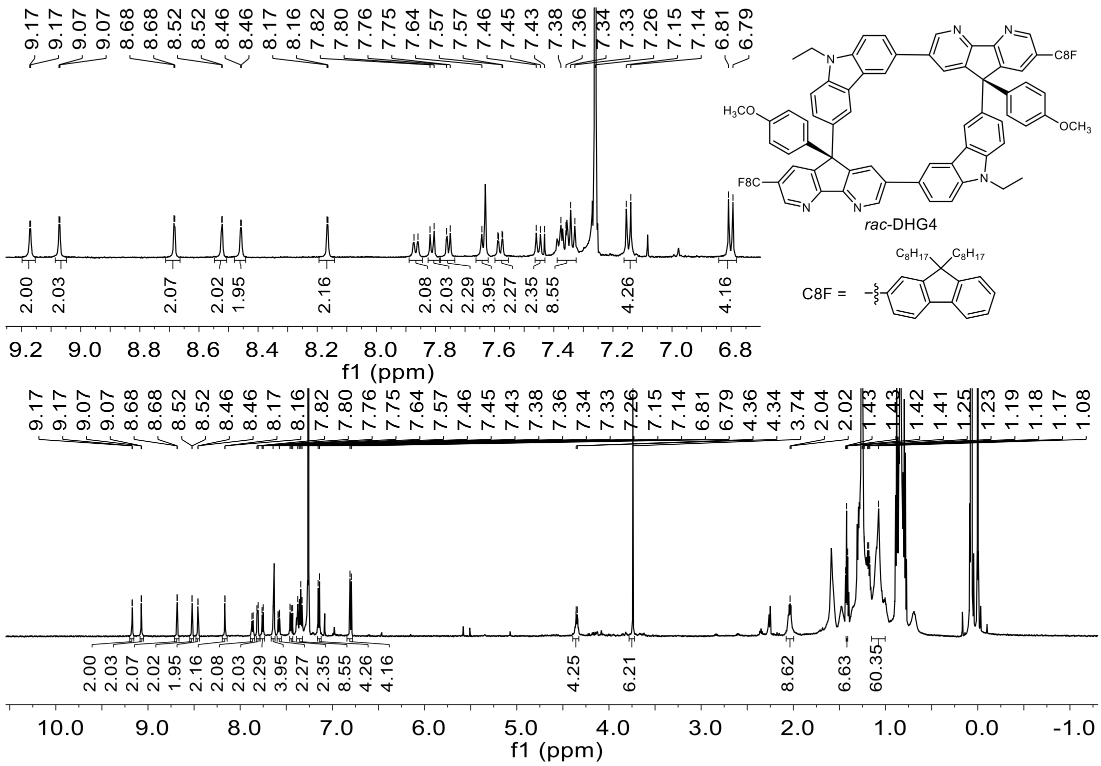

Supplementary Figure 89. ^{1}H NMR spectra for ***rac*-DHG4** in $CDCl_3$.

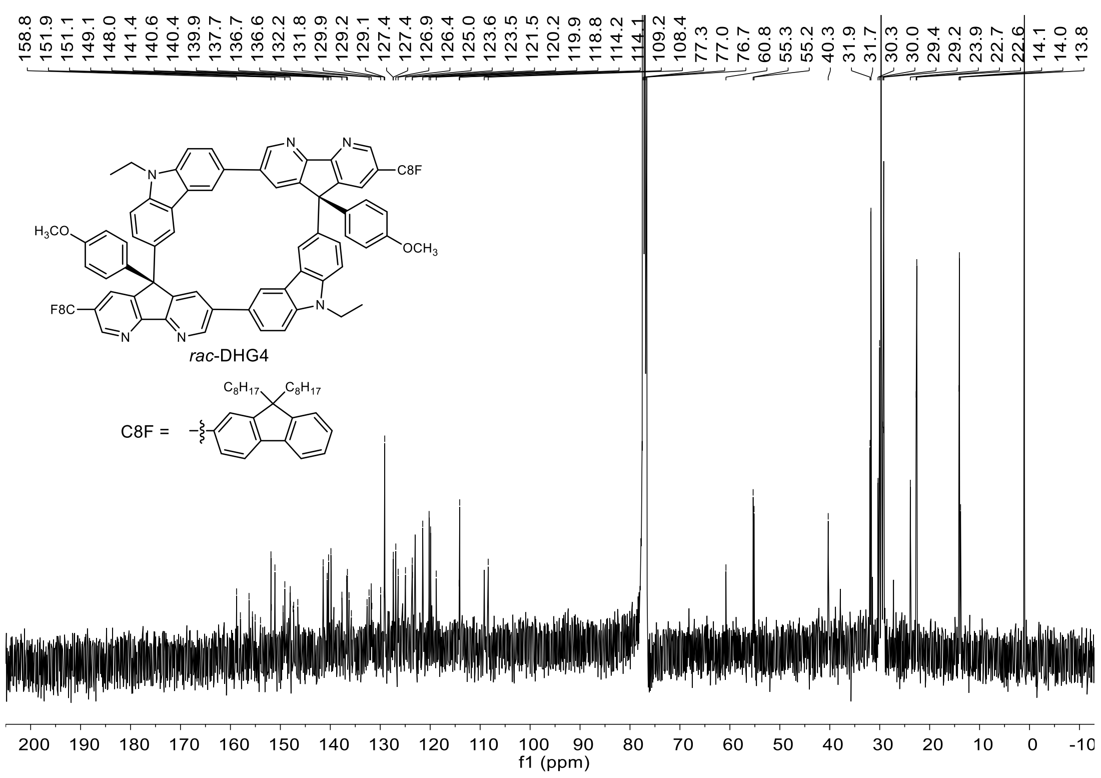

Supplementary Figure 90. ^{13}C NMR spectra for ***rac*-DHG4** in $CDCl_3$.

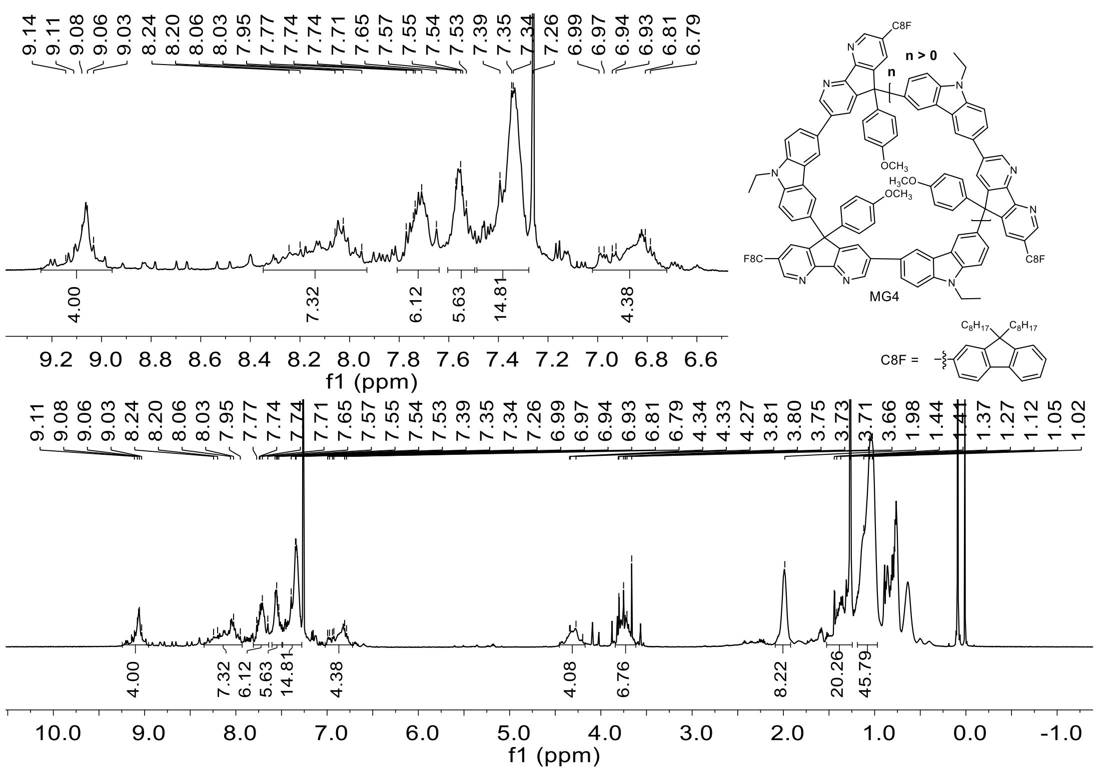

Supplementary Figure 91. ^{1}H NMR spectra for **MG4** in $CDCl_3$.

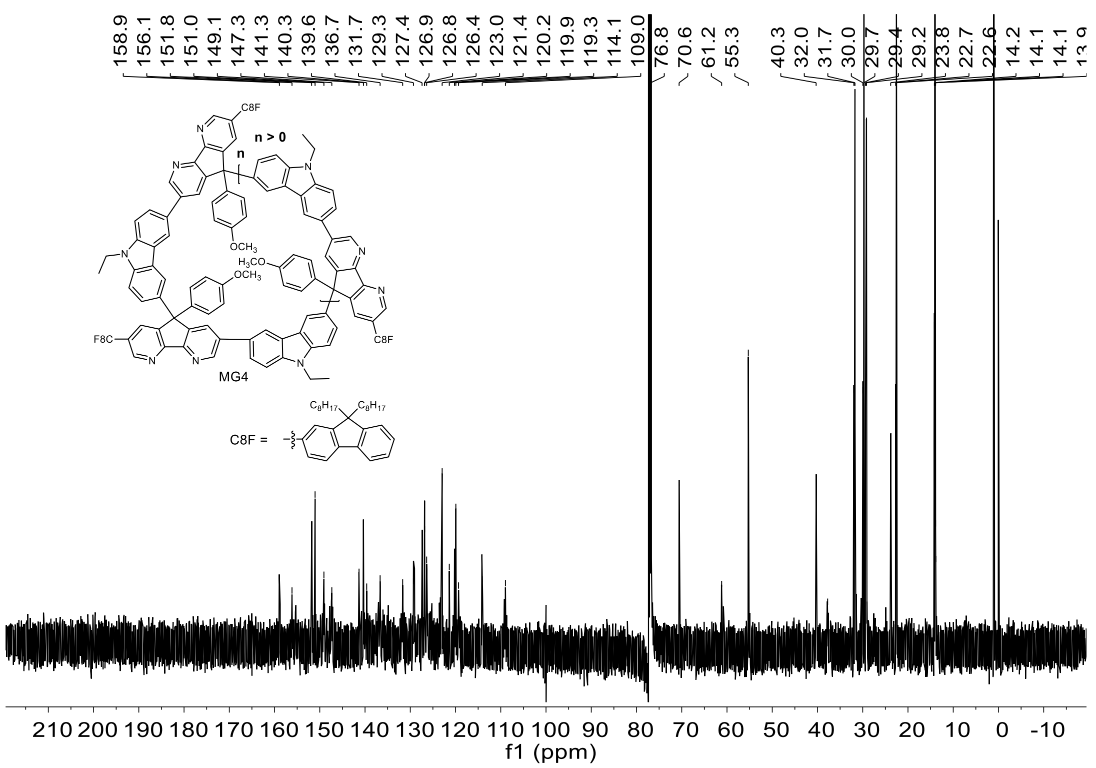

Supplementary Figure 92. ^{13}C NMR spectra for **MG4** in $CDCl_3$.

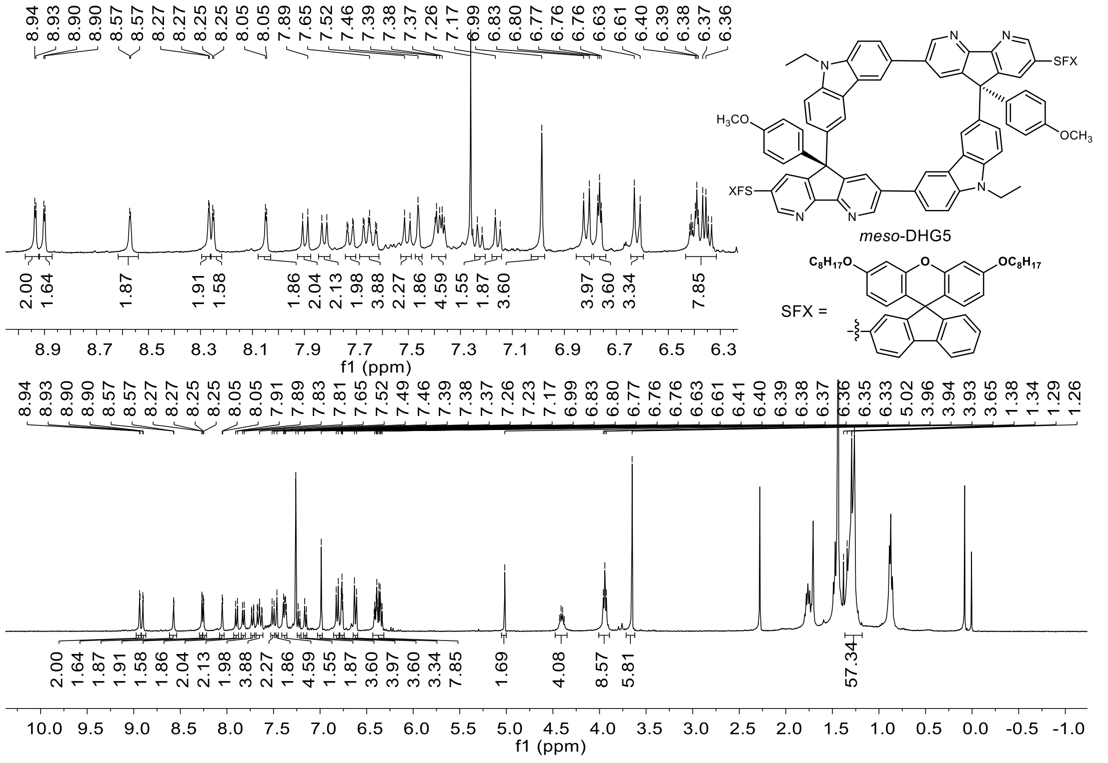

Supplementary Figure 93. ^{1}H NMR spectra for ***meso*-DHG5** in $CDCl_3$

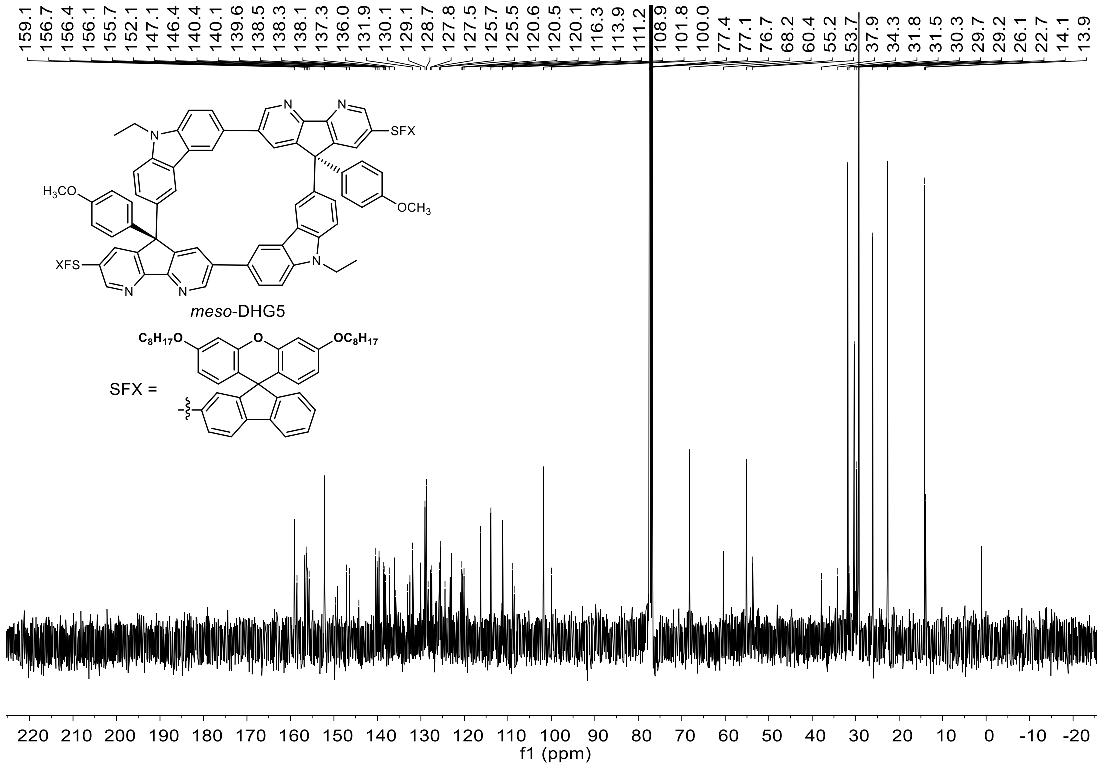

Supplementary Figure 94. ^{13}C NMR spectra for ***meso*-DHG5** in $CDCl_3$

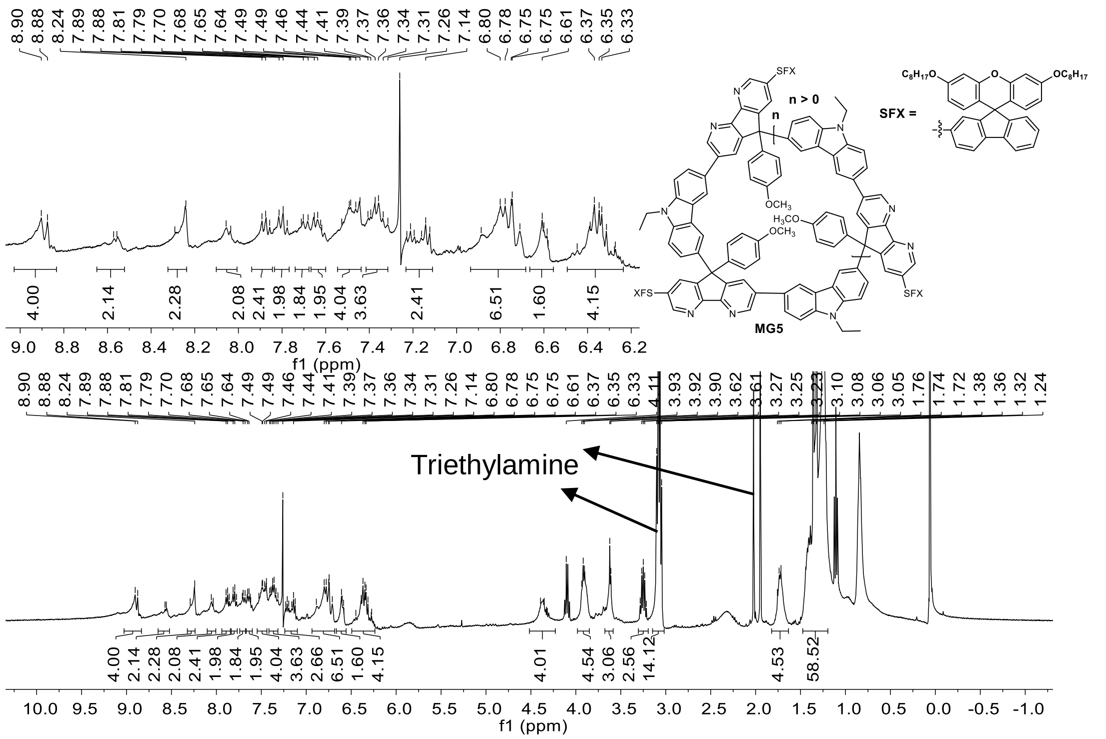

Supplementary Figure 95. ^{1}H NMR spectra for **MG5** in $CDCl_3$. In this spectra, the triethylamine (3.11 ppm and 1.38 ppm) were complexed and difficult to remove.

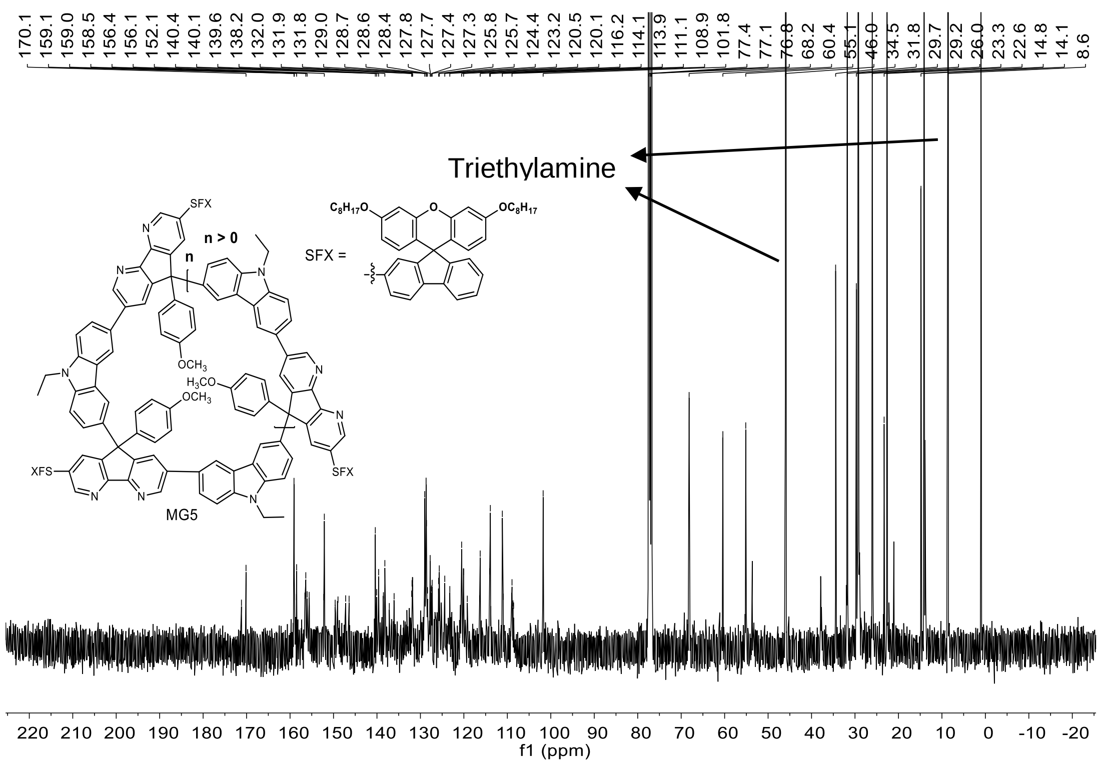

Supplementary Figure 96. ^{13}C NMR spectra for **MG5** in $CDCl_3$. In this spectra, the triethylamine (46.0 and 8.6 ppm) were complexed and difficult to remove.

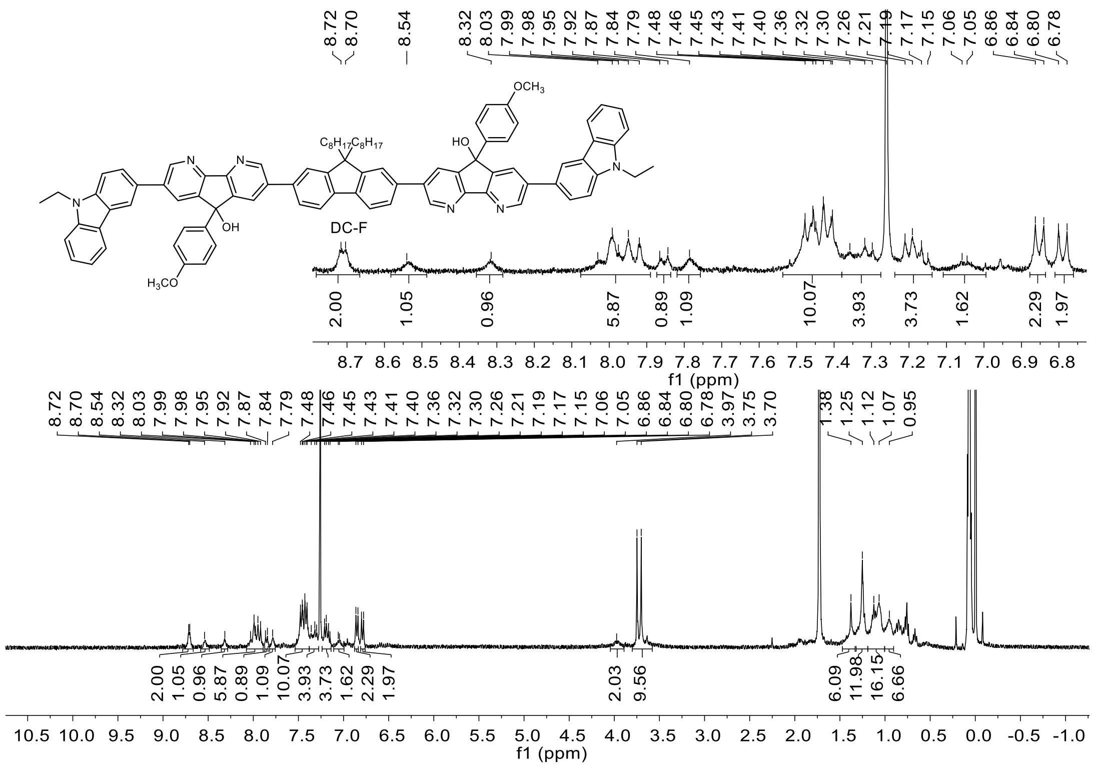

Supplementary Figure 97. ^{1}H NMR spectra for **DC-F** in $CDCl_3$

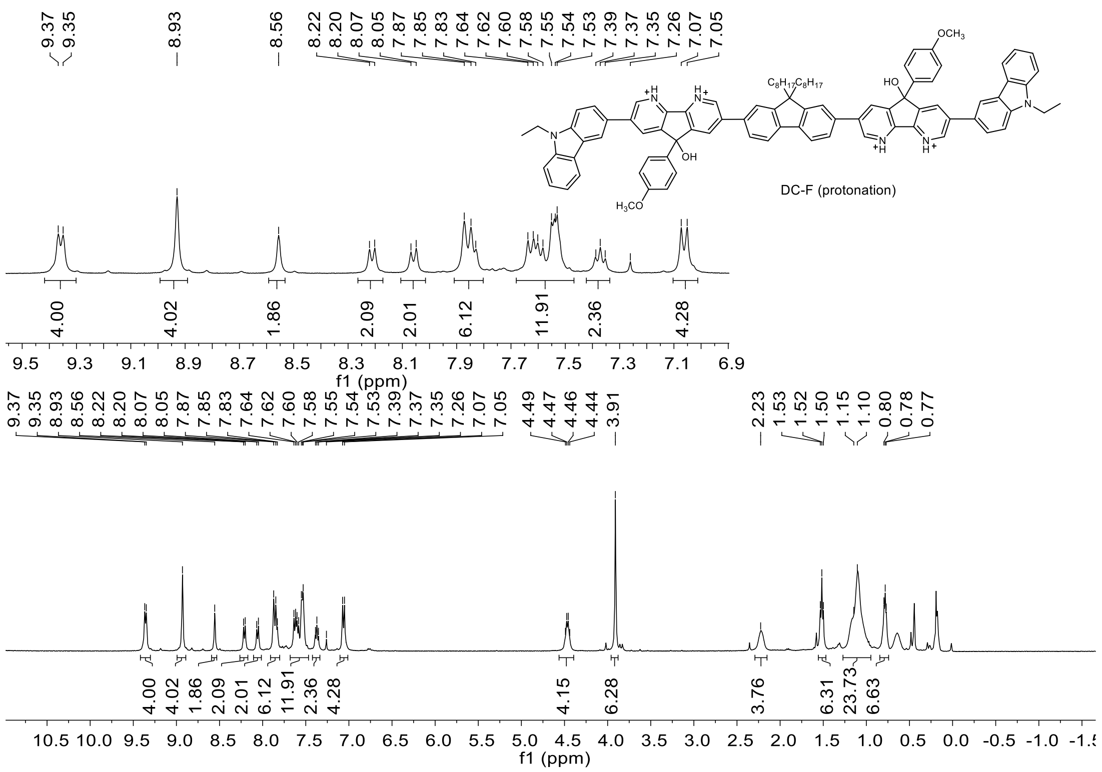

Supplementary Figure 98. ^{1}H NMR spectra for protonated **DC-F** in $CDCl_3$ and CF_3COOH mixed solvents. The signal of CF_3COOH is located at 11.8 ppm.

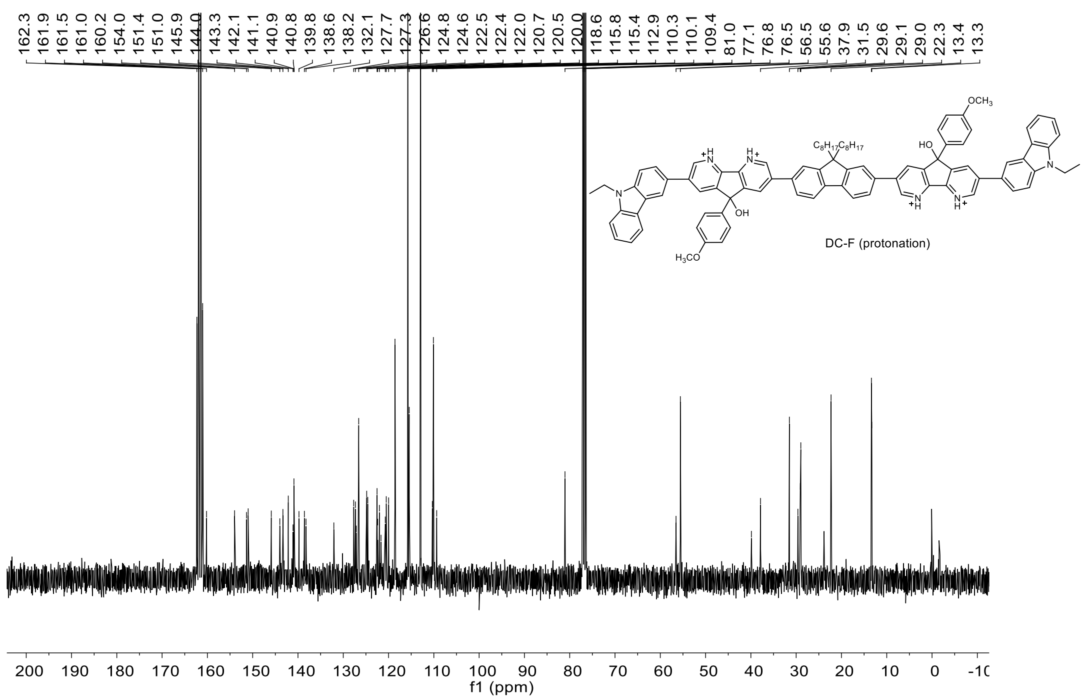

Supplementary Figure 99. ^{13}C NMR spectra for protonated **DC-F** in $CDCl_3$ and CF_3COOH mixed solvents. The signals at 162.3, 161.9, 161.5, 161.0, 118.6, 115.8, 112.9 and 110.1 ppm are assigned to additional CF_3COOH.

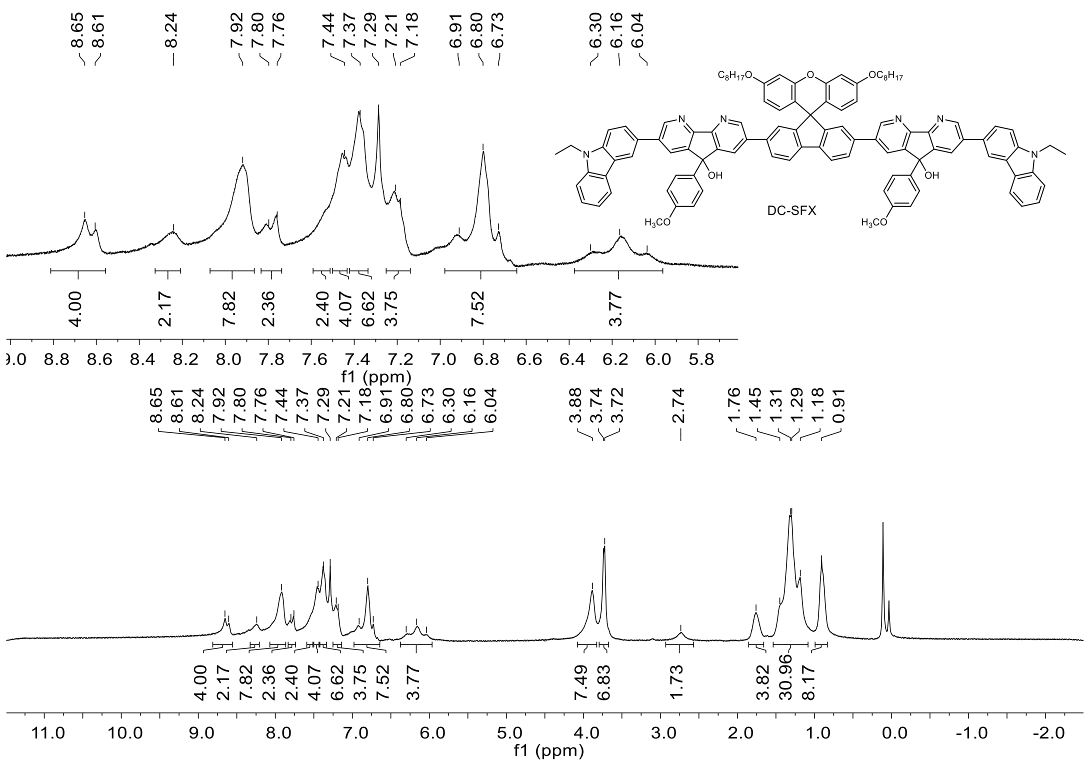

Supplementary Figure 100. ^{1}H NMR spectra for **DC-SFX** in $CDCl_3$

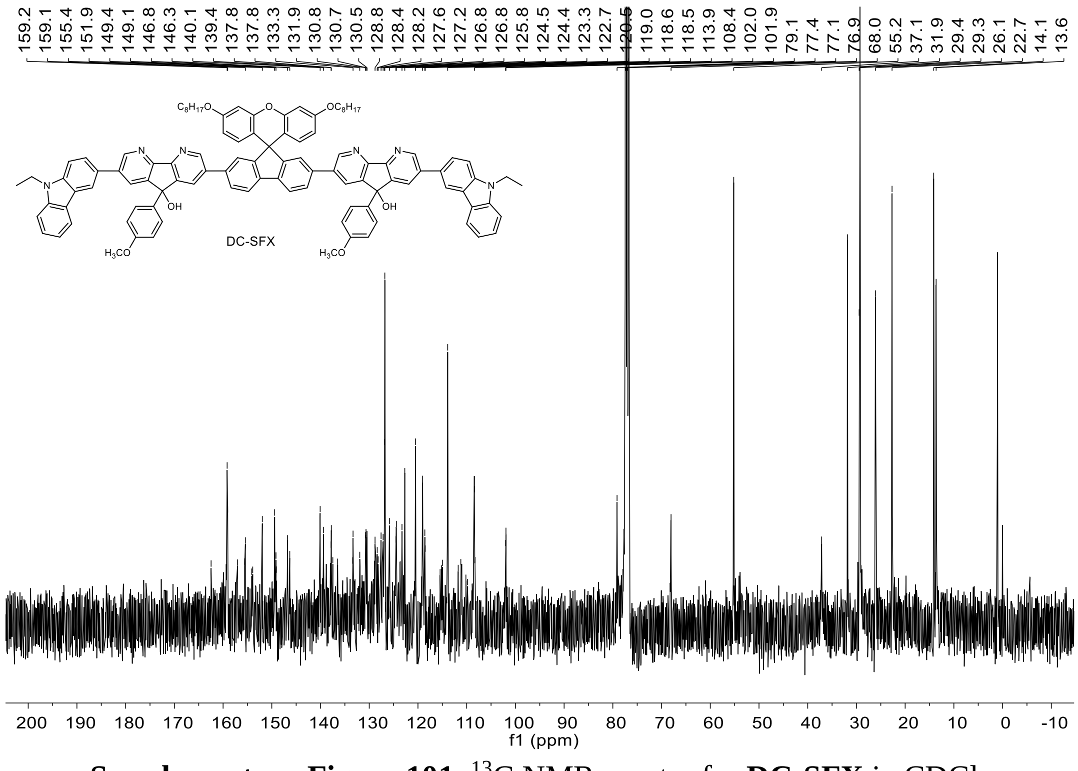

Supplementary Figure 101. ^{13}C NMR spectra for **DC-SFX** in $CDCl_3$

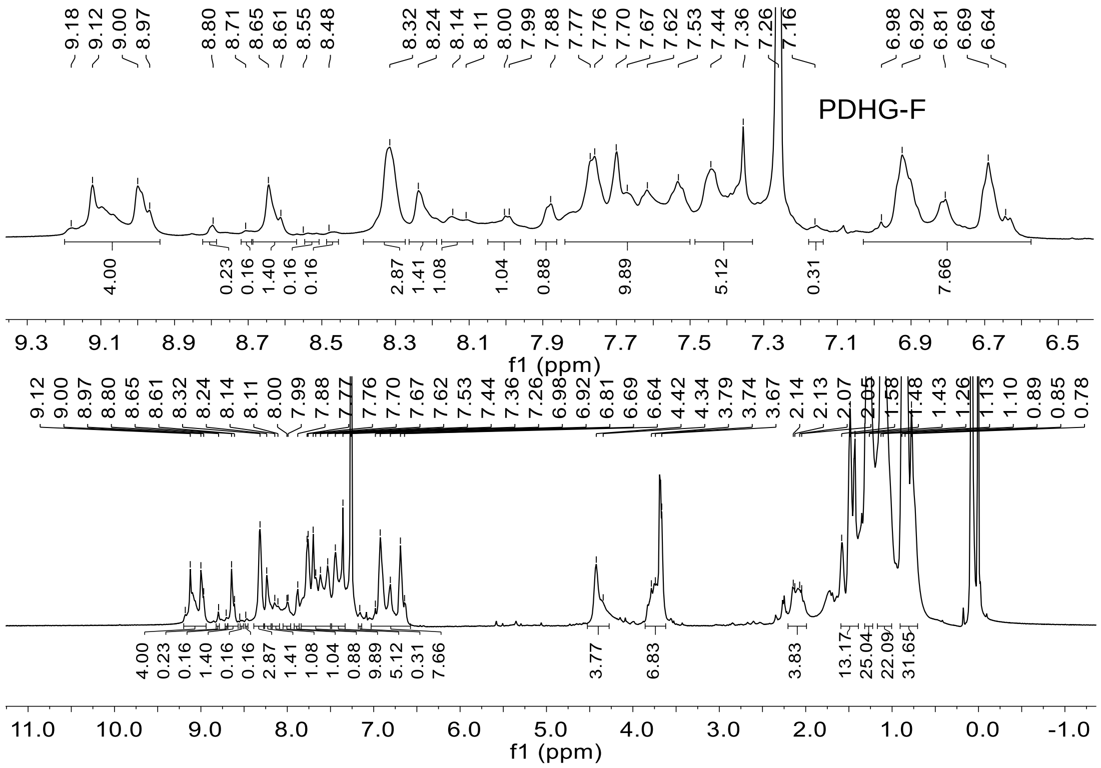

Supplementary Figure 102. ^{1}H NMR spectra for **PDHG-F** in $CDCl_3$

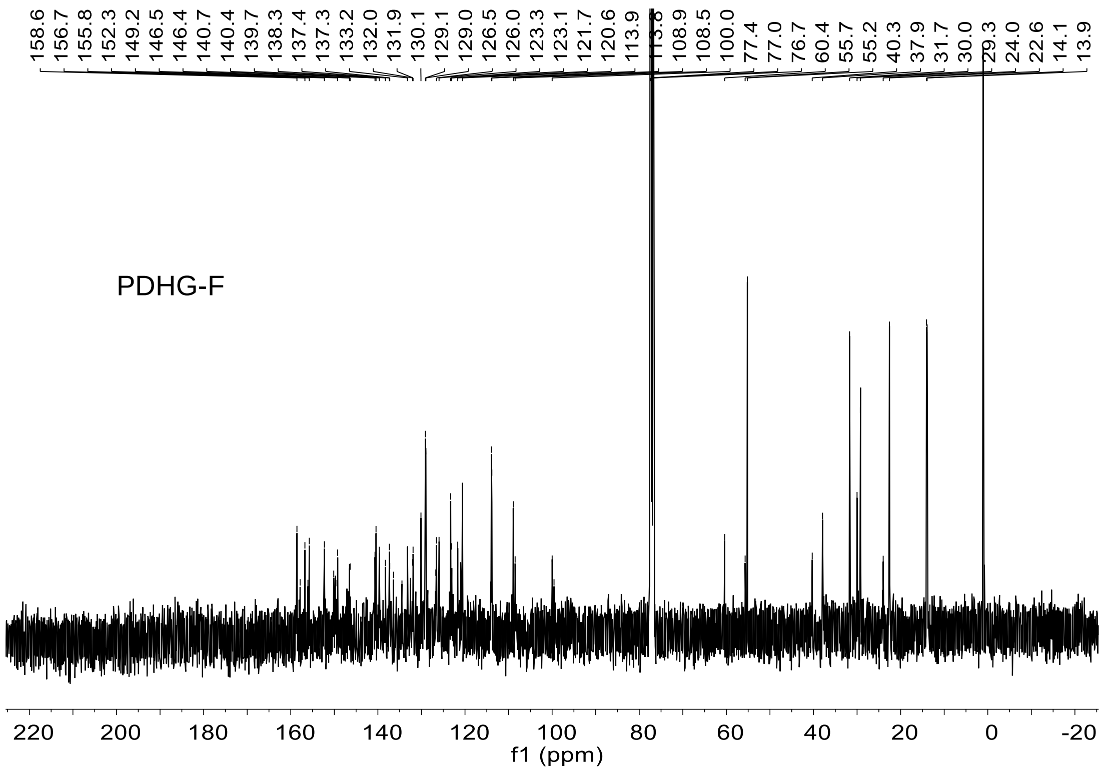

Supplementary Figure 103. ^{13}C NMR spectra for **PDHG-F** in $CDCl_3$

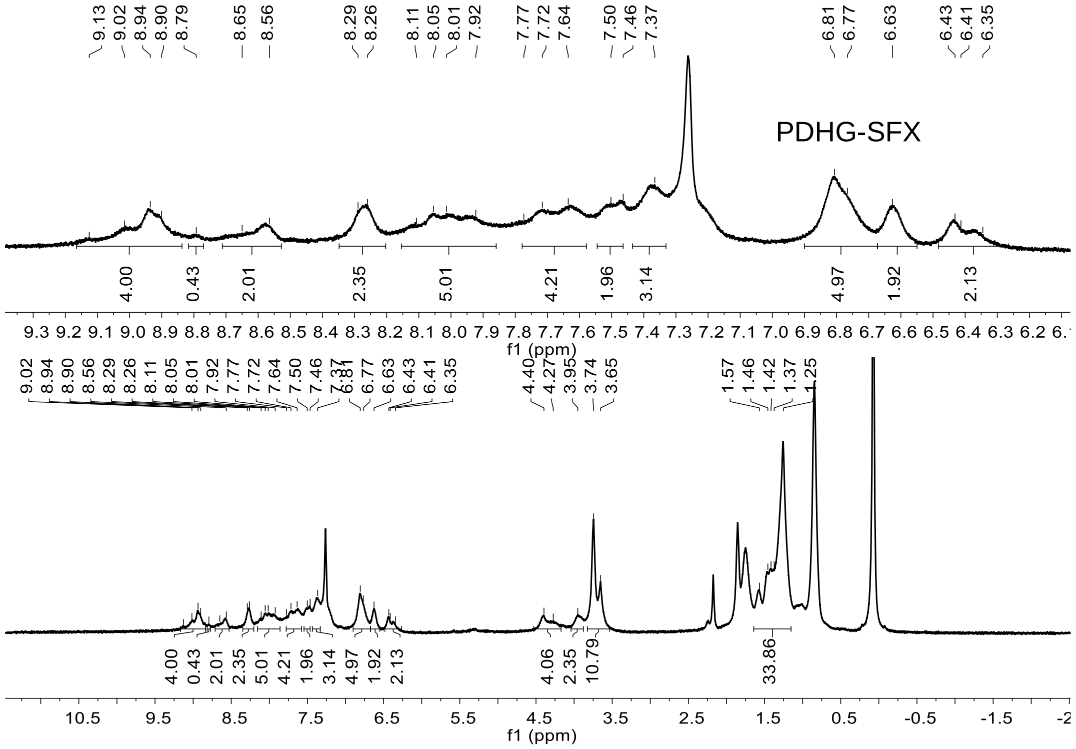

Supplementary Figure 104. ^{1}H NMR spectra for **PDHG-SFX** in $CDCl_3$

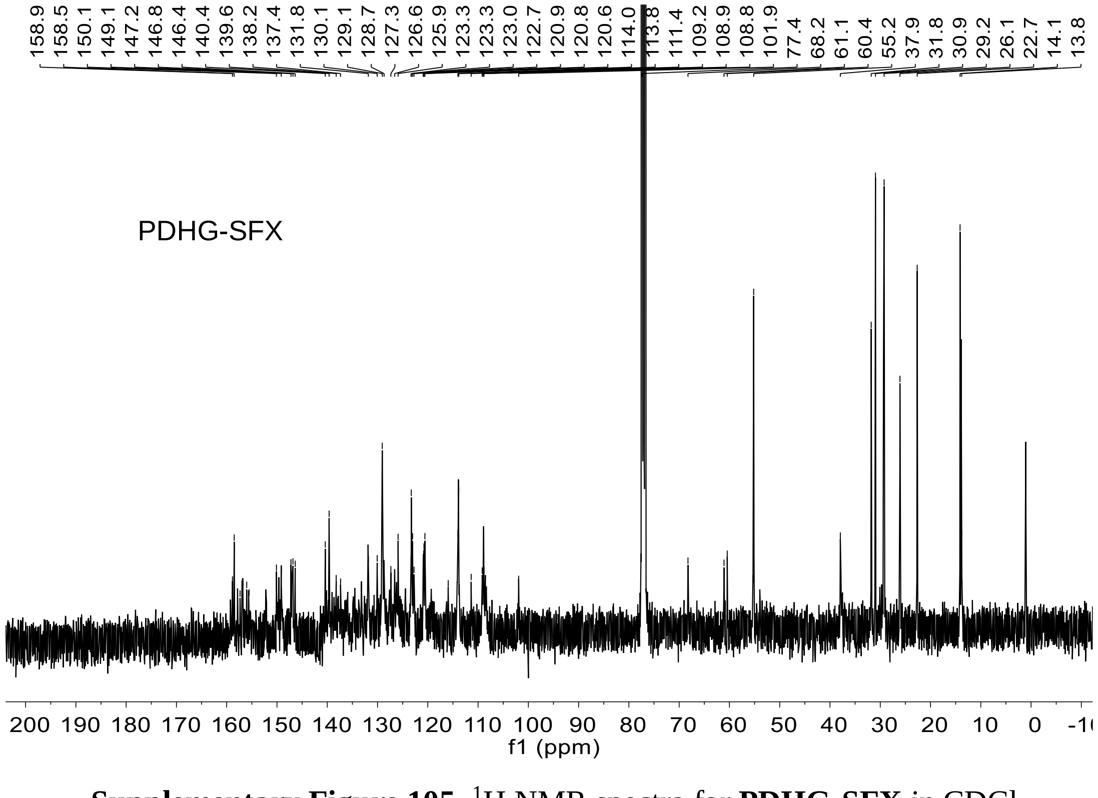

Supplementary Figure 105. ^{1}H NMR spectra for **PDHG-SFX** in $CDCl_3$

Supplementary Note 11. Single crystal crystallography

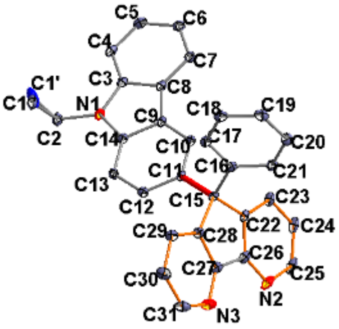

Supplementary Figure 106. Single crystal crystallography of **2a**

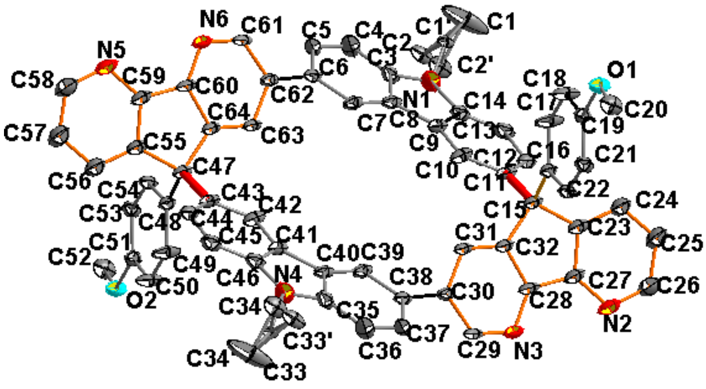

Supplementary Figure 107. Single crystal crystallography of ***meso*-DHG1**

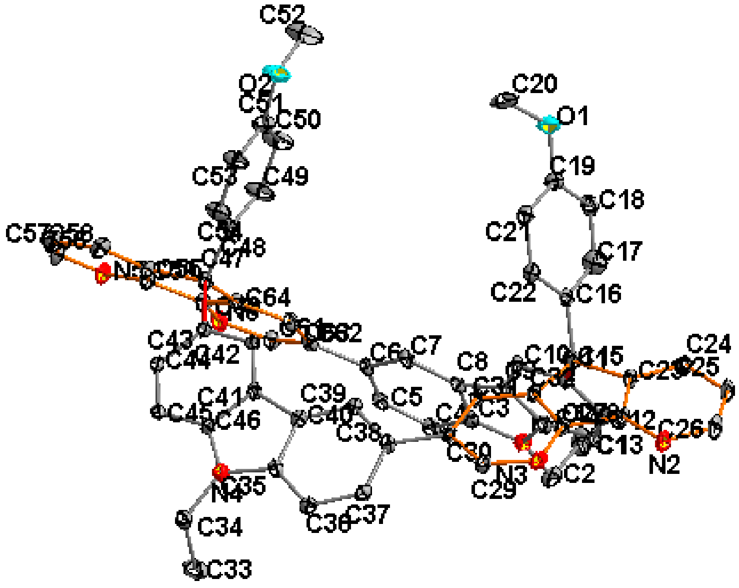

Supplementary Figure 108. Single crystal crystallography of ***rac*-DHG1**

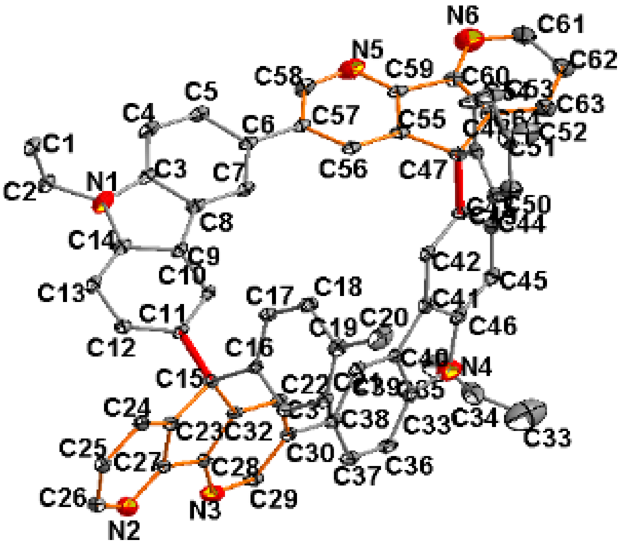

Supplementary Figure 109. Single crystal crystallography of ***rac*-DHG2**

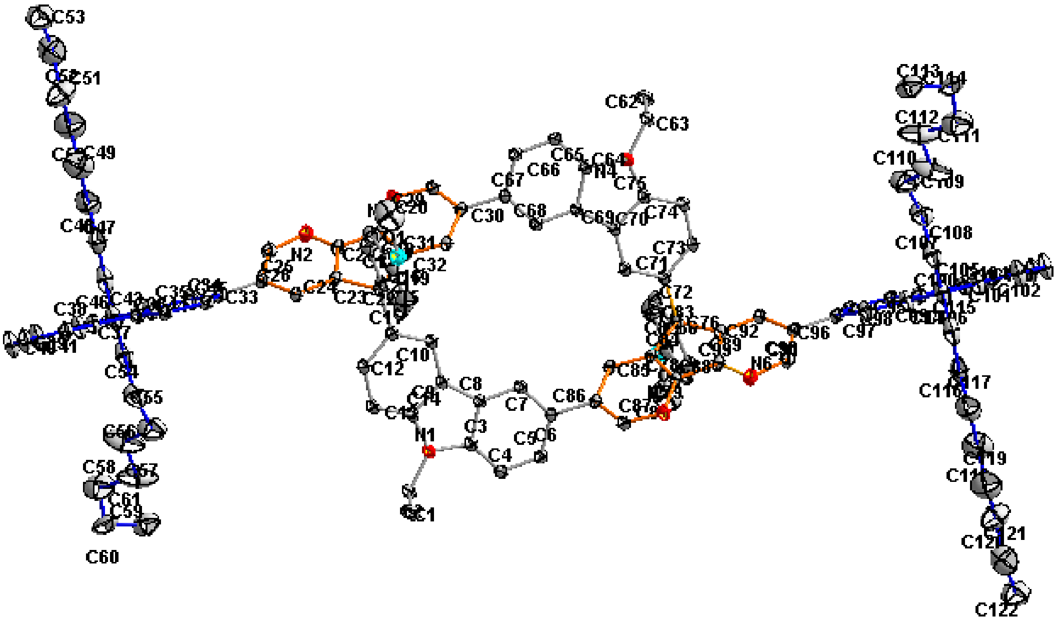

Supplementary Figure 110. Single crystal crystallography of ***meso*-DHG4**

Supplementary Table 4. The single crystal results.

Compound	**2a**	***meso*-DHG1**	***rac*-DHG1**[a]	***rac*-DHG2**	***meso*-DHG4**
CCDC	1886054	1886046	1886053	1886052	1938823
Formula	$C_{31} H_{23} N_3$	$C_{64}H_{46}N_6O_2$	$C_{64}H_{46}N_6O_2$	$C_{64} H_{46} N_6$	$C_{122} H_{126} N_6 O_2$, 2($H_2 O_2$)
Space group	P-1	P-1	C12/c1	P-1	P-1
Cell parameters	a = 9.0367 Å<br>b = 11.1446 Å<br>c = 12.5461 Å<br>α = 68.775°<br>β = 82.042°<br>γ = 80.769°	a = 10.053 Å<br>b = 10.698 Å<br>c = 14.764 Å<br>α = 91.431°<br>β = 94.150°<br>γ = 112.829°	a = 13.1939 Å<br>b = 31.701 Å<br>c = 27.112 Å<br>α = 90°<br>β = 90°<br>γ = 90°	a = 11.854 Å<br>b = 12.194 Å<br>c = 19.889 Å<br>α = 89.105°<br>β = 77.085°<br>γ = 79.327°	a = 13.6680 Å<br>b = 14.1611 Å<br>c = 15.3244 Å<br>α = 93.561°<br>β = 104.376°<br>γ = 116.019°
Cell volume (Å^3)	1158.1	1457.17	11340	2752.8	2531.69
D_{Cal} (g/cm^3)	1.255	1.06095	1.326	1.085	1.165
M_{μ} (mm^{-1})	0.074	0.065	0.219	0.064	0.550
N_{ref}	8728	4887	14132	9488	8619
F(000)	460.0	488.0	4744.0	944.0	952.0
Z	2	1	8	2	1
R (reflections)	0.0631 (7696)	0.0785 (4639)	0.1277 (8662)	0.1111 (8506)	0.0880 (6315)
wR_2 (reflections)	0.1770(10361)	0.2155 (7123)	0.4045(14132)	0.2742 (12934)	0.2903 (8619)
N_{par}	319	347	751	646	616

[a] It is noted that the solvents of DCM and methoxyl-alcohol were participated in the single crystallography. The A-typed errors were only focused on the solvent molecule. There is no A-typed errors for ***rac*-DHG1** backbones.

For single crystal crystallography of **2a**, the cif file and check.cif file are provided as the **Supplementary Data 12** and **Supplementary Data 13** respectively. For single crystal crystallography of ***meso*-DHG1**, the cif file and check.cif file are provided as the **Supplementary Data 14** and **Supplementary Data 15** respectively. For single crystal crystallography of ***rac*-DHG1**, the cif file and check.cif file are provided as the **Supplementary Data 16** and **Supplementary Data 17** respectively. For single crystal crystallography of ***rac*-DHG2**, the cif file and check.cif file are provided as the **Supplementary Data 18** and **Supplementary Data 19** respectively. For single crystal crystallography of ***meso*-DHG4**, the cif file and check.cif file are provided as the **Supplementary Data 20** and **Supplementary Data 21** respectively.

Supplementary References

1. Li, W.-J. et al. Synthesis and characterization of diazafluorene-based oligofluorenes and polyfluorene. *Polym. Chem.* **4**, 1796-1802 (2013).

2. Yu, Y. et al. 4,5-Diazafluorene-Based Donor–Acceptor Small Molecules as Charge Trapping Elements for Tunable Nonvolatile Organic Transistor Memory. *Adv. Sci.* **5**, 1800747 (2018).
3. Qian, Y. et al. Spiro[fluorene-9,9’-xanthene]-based universal hosts for understanding structure − property relationships in RGB and white PhOLEDs. *RSC Adv.* **5**, 29828-29836 (2015).
4. Scott, A.P. & Radom, L. Harmonic vibrational frequencies: an evaluation of Hartree− Fock, Møller− Plesset, quadratic configuration interaction, density functional theory, and semiempirical scale factors. *J. Phys. Chem.* **100**, 16502-16513 (1996).
5. Sun, H. COMPASS: an ab initio force-field optimized for condensed-phase applications overview with details on alkane and benzene compounds. *J. Phys. Chem. B* **102**, 7338-7364 (1998).
6. Xie, L.-H. et al. Facile Synthesis of Complicated 9,9-Diarylfluorenes Based on BF3·Et2O-Mediated Friedel−Crafts Reaction. *Org. Lett.* **8**, 3701-3704 (2006).
7. Saito, S., Ohwada, T. & Shudo, K. Friedel-Crafts-type reaction of benzaldehyde with benzene. Diprotonated benzaldehyde as the reactive intermediate. *J. Am. Chem. Soc.* **117**, 11081-11084 (1995).
8. Flory, P.J. The Configuration of Real Polymer Chains. *J. Chem. Phys.* **17**, 303-310 (1949).
9. Yu, J., Wang, Z. & Chu, B. Kinetic study of coil-to-globule transition. *Macromolecules* **25**, 1618-1620 (1992).
10. Tanaka, G. & Mattice, W.L. Chain collapse by atomistic simulation. *Macromolecules* **28**, 1049-1059 (1995).
11. Lindvig, T., Michelsen, M.L. & Kontogeorgis, G.M. A Flory–Huggins model based on the Hansen solubility parameters. *Fluid Phase Equilib.* **203**, 247-260 (2002).
12. Zellers, E.T. Three-dimensional solubility parameters and chemical protective clothing permeation. I. Modeling the solubility of organic solvents in Viton® golves. *J. Appl. Polym. Sci.* **50**, 513-530 (1993).
13. Luo, Z. & Jiang, J. Molecular dynamics and dissipative particle dynamics simulations for the miscibility of poly(ethylene oxide)/poly(vinyl chloride) blends. *Polymer* **51**, 291-299 (2010).
14. Scholte, T.G., Meijerink, N.L.J., Schoffeleers, H.M. & Brands, A.M.G. Mark–Houwink equation and GPC calibration for linear short-chain branched polyolefines, including polypropylene and ethylene–propylene copolymers. *J. Appl. Polym. Sci.* **29**, 3763-3782 (1984).
15. Grubisic, Z., Rempp, P. & Benoit, H. A universal calibration for gel permeation chromatography. *J. Polym. Sci. Part B: Polym. Lett.* **5**, 753-759 (1967).
16. Kondratuk, D.V. et al. Supramolecular nesting of cyclic polymers. *Nat. Chem.* **7**, 317-322 (2015).
17 Vanhee, S. *et al.* Synthesis and characterization of rigid rod poly(p-phenylenes). *Macromolecules* **29**, 5136-5142 (1996).

18 Naredla, R. R., Zheng, C., Lill, S. O. N. & Klumpp, D. A. Charge Delocalization and Enhanced Acidity in Tricationic Superelectrophiles. *J. Am. Chem. Soc.* **133**, 13169-13175 (2011).

19 Klumpp, D. A. Superelectrophiles: Charge–Charge Repulsive Effects. *Chem. Eur. J.* **14**, 2004-2015 (2008).

20. Flory, P.J. Principles of polymer chemistry (Cornell University Press, 1953).

21. Chatterjee, S. et al. Fiaud's Acid: A Brønsted Acid Catalyst for Enantioselective Friedel–Crafts Alkylation of Indoles with 2-Alkene-1,4-diones. *Org. Lett.* **19**, 3426-3429 (2017).

22. Zou, L. et al. Friedel–Crafts A2 + B4 Polycondensation toward Regioselective Linear Polymer with Rigid Triphenylmethane Backbone and Its Property as Gas Separation Membrane. *Macromolecules* **51**, 6580-6586 (2018).
